# DFT Modeling of the Alternating Radical Copolymerization and Alder-Ene Reaction between Maleic Anhydride and Olefins

**DOI:** 10.3390/polym12040744

**Published:** 2020-03-27

**Authors:** Ilya Nifant’ev, Alexander Vinogradov, Alexey Vinogradov, Pavel Ivchenko

**Affiliations:** 1 A.V. Topchiev Institute of Petrochemical Synthesis RAS, 29 Leninsky Pr., Moscow 119991, Russia; amvvin@mail.ru (A.V.); ,; 2 Chemistry Department, M.V. Lomonosov Moscow State University, 1 Leninskie Gory Str., Building 3, Moscow 119991, Russia

**Keywords:** Alder-ene reaction, alkenes, alternating copolymerization, density functional theory, free radical polymerization, maleic anhydride

## Abstract

The free radical copolymerization of electron-acceptor and electron-donor vinyl monomers represents a particular case of sequence-controlled polymerization. The reactions of maleic anhydride (MA) or related compounds (acceptor comonomers) with α-olefins (donor comonomers) result in the formation of the alternating copolymers that have clear prospects for petrochemical and biomedical applications. However, in contrast to the well-established polymerization of acrylate monomers, these processes have not been studied theoretically using the density functional theory (DFT) calculations. In our research, we performed a comprehensive theoretical analysis of the free radical copolymerization of MA and closely related maleimide with different structural types of olefins at mpw1pw91/6-311g(d) level of the DFT. The results of our calculations clearly indicated the preference of the alternating reaction mode for the copolymerization of MA with α-olefins, isobutylene and prospective unsaturated monomers, as well as methylenealkanes. The DFT modeling of the thermally induced Alder-ene reaction between MA and olefins allowed to exclude this reaction from the scope of possible side processes at moderately high temperatures. Comparative analysis of MA and N-methylmaleimide (MMI) reactivity shown that the use of MMI instead of MA makes no sense in terms of the reaction rate and selectivity.

## 1. Introduction

**Scheme 1:**
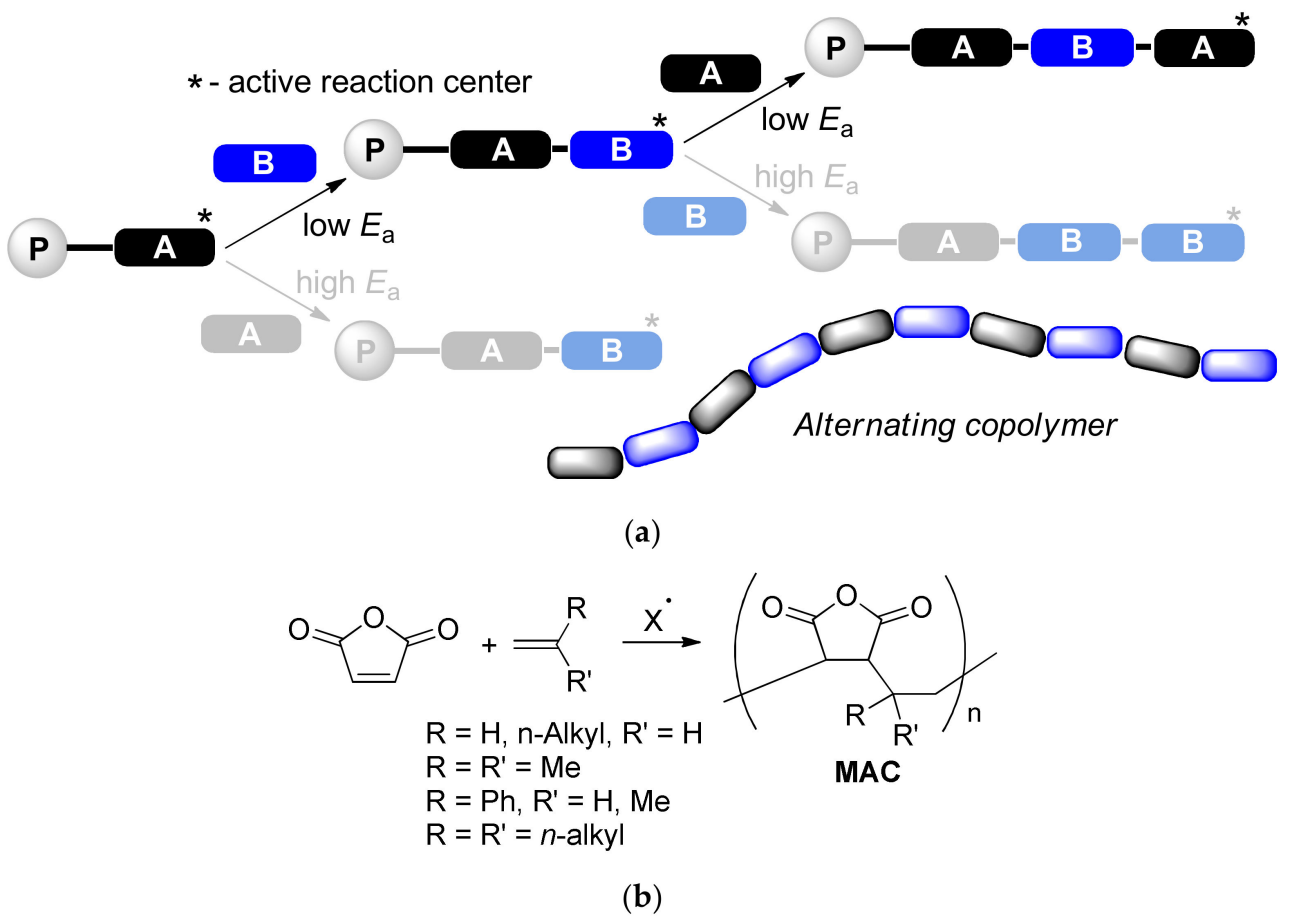
(**a**) Sequence-controlled polymerization and formation of the alternating copolymers; (**b**) Maleic anhydride copolymers (MAC) with olefins.

Sequence-controlled polymerization (SCP, Scheme 1a) represents an efficient approach to macromolecules with a determined structure and desired characteristics [1]. Free radical alternating copolymerization of donor and acceptor vinyl monomers is a special case of SCP, which attracts the attention of researchers owing to its experimental simplicity and wide selection of the monomers. Different derivatives of the unsaturated acids such as acrylates, acrylamides, maleic anhydride (MA) or maleimide can be used as an acceptor comonomers; donor comonomers are represented by vinyl ethers and olefins including substituted styrenes. Maleic anhydride copolymers (MAC) with donor monomers are highly attractive due to low cost, structural variability and high reactivity of succinic anhydride subunits (Scheme 1b). To date, MAC with ethylene [2,3], isobutylene [4,5,6] and styrenes [7,8] are synthesized and structurally characterized, alternating microstructure of these copolymers was confirmed by spectral methods [3,9,10]. MA copolymers with linear α-olefins are usually attributed to alternating copolymers [1,11,12,13,14,15,16] (Scheme 1), however these macromolecules are regarded as statistic in the few publications [17,18]. The wide spectra of the functional derivatives of MAC are considered as prospective membranes and films for power cells [19,20,21], pigments [22,23,24], polymers for biomedical applications [16,25,26,27,28,29,30,31,32], and highly efficient depressor additives and flow improvers for fuels and oils [15,18,33,34,35,36,37,38,39,40,41,42,43,44,45].

**Scheme 2:**
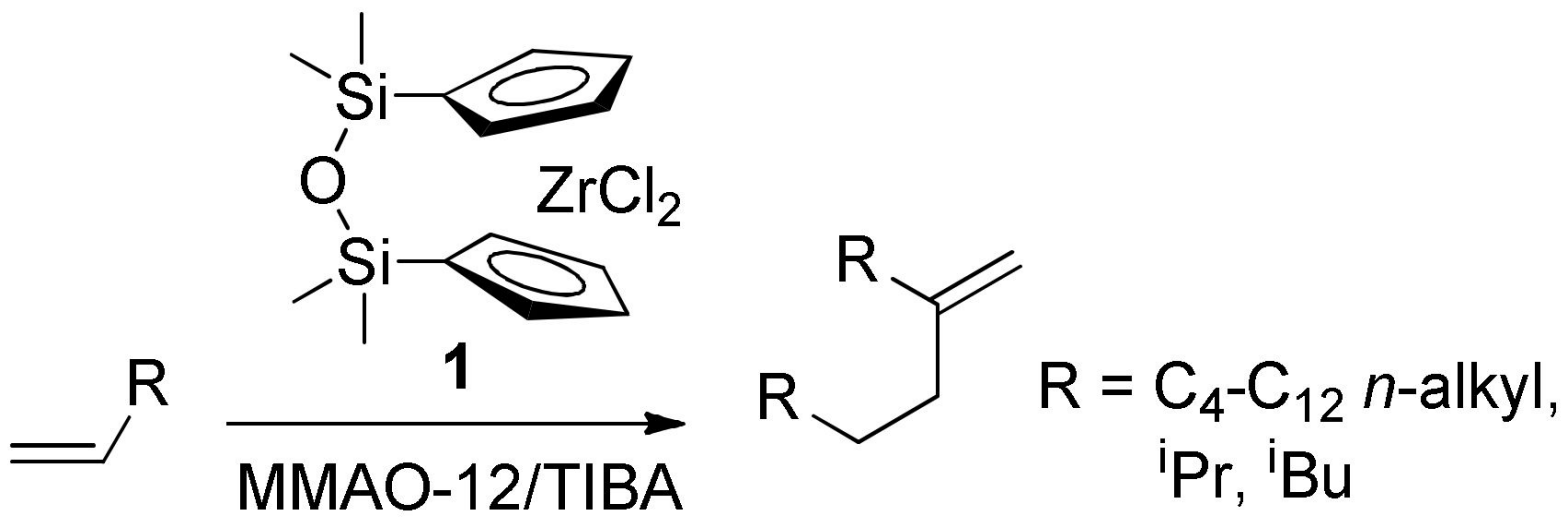
Selective dimerization of α-olefins.

Recently we performed copolymerization of MA with methylenealkanes RC(=CH_2_)CH_2_CH_2_R that are close structural analogs of isobutylene [46]. The ratio of comonomers in the products of copolymerization was ~1:1 (by ^1^H NMR and elemental analysis). The high synthetic availability of the methylenealkanes that can be obtained by zirconocene-catalyzed dimerization of α-olefins (Scheme 2) [47,48,49] makes it possible to consider methylenealkane/MA copolymers as low-cost materials that contain amphiphilic subunits and branched alkyl substituents with regulated length. The perceived potential of the practical application of these materials required a better understanding of the reaction mechanism and limitations.

The reasons of the formation of alternate polymers by MA and olefins are still unclear in detail. The methods of quantum chemistry based on the density functional theory were successfully used for the analysis of different radical polymerization processes [50,51,52], including homo- and copolymerization of acrylates [53,54,55,56,57,58,59,60,61,62,63,64,65,66,67,68,69,70,71], acrylamides [70,72,73], and acrylonitrile [74]. However, as far as we know, DFT-based methods have not been extensively used for visualization and comprehensive analysis of the free radical alternating copolymerization of donor and cyclic acceptor monomers, except the publications of Matsumoto et al., devoted to the copolymerization of *N*-methylmaleimide (MMI) with olefins [75], and MA with 2,4-dimethylpenta-1,3-diene [76]. 

**Scheme 3:**
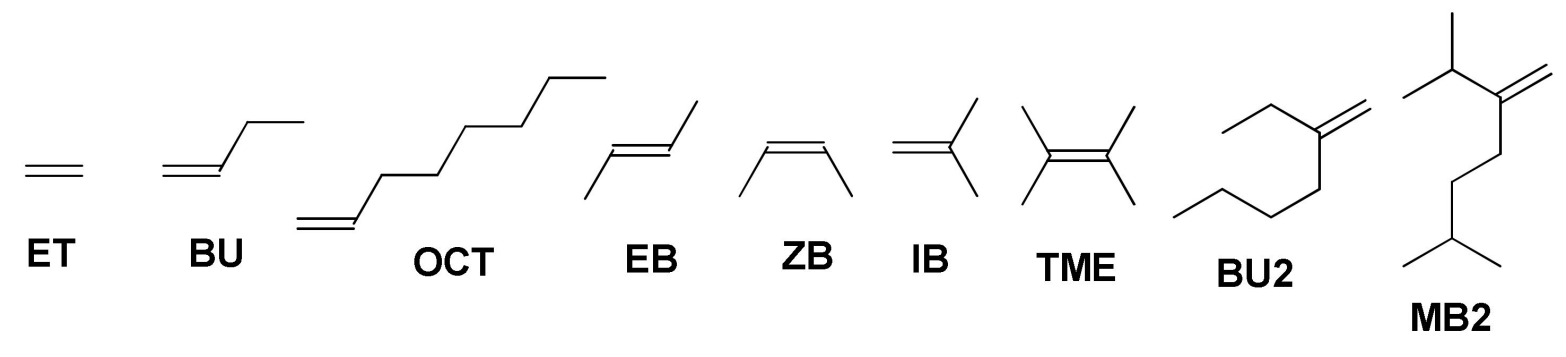
Molecular structures of the olefins studied in the reactions with MA.

In our study, we present the results of the DFT modeling of copolymerization of MA and closely related MMI with a number of olefins, namely, ethylene (ET), but-1-ene (BU), oct-1-ene (OCT) (*E*)-but-2-ene (EB), (*Z*)-but-2-ene (ZB), 2-methylprop-1-ene (isobutylene IB), 2,3-dimethylbut-2-ene (tetramethylethylene TME), 3-methyleneheptane (vinylidene dimer of but-1-ene BU2) and 2,6-dimethyl-3-methyleneheptane (vinylidene dimer of 3-methylbut-1-ene MB2) presented in Scheme 3. The results of our calculations allowed to describe the reaction mechanism in some detail, and predicted the limitations of the reaction by the structure of the olefin comonomer. The following material illustrates some fundamental principles of free radical polymerization mechanism, and therefore may be of interest for the chemists who work in the fields of both polymer science and higher education.

## 2. Materials and Methods 

### 2.1. DFT Calculations

The initial cartesian coordinates of the stationary points had been found by PRIRODA program (version 4.0, M.V. Lomonosov Moscow University, Moscow, Russia) [77] using the 3ζ basis. The final optimization and determination of the thermodynamic parameters for stationary points and transition states were carried out using Gaussian 09 program [78] for gas phase at 298.15 K, the root mean square (RMS) force criterion was 3 × 10^−4^. The mpw1pw91 functional [79] and 6-311g(d) basis set [80] were used in the optimizations. Transition states were found by energy scanning with sequential changing of key geometric parameters with a step of 0.01 Å followed by Berny optimization, and confirmed by intrinsic reaction coordinate (IRC) simulations.

For comparison of the free activation energies for Alder-ene reaction and alternating copolymerization of MA and BU2 we optimized the structures of the starting compounds, intermediates and transition states at 80 °C.

Cartesian coordinates, molecular structure plots, and key energy parameters of the optimized structures are presented in the Supporting Information.

### 2.2. General Experimentsl Remarks

TIBA (1 M solution in hexane, Merck, NJ, USA), MMAO-12 (1.52 M solution in toluene, Merck, NJ, USA), azobisisobutyronitrile (AIBN, Merck, NJ, USA), benzoyl peroxide (Merck, NJ, USA), mesitylene (Merck, NJ, USA) and CDCl_3_ (99.8% ^2^H, Cambridge Isotope Laboratories, Inc., MS, USA) were used as purchased. Toluene (Merck, NJ, USA) was refluxed over sodium and stored under argon atmosphere. Maleic anhydride (MA, Merck, NJ, USA) was recrystallized from toluene before use. Hex-1-ene (Merck, NJ, USA) was stored over Na wire and distilled under argon. The ^1^H NMR spectra were recorded on a Bruker AVANCE 400 spectrometer (400 MHz, Bruker, MS, USA) at 20 °C. The chemical shifts are reported in ppm relative to the solvent residual peaks.

Zirconium complex **1** was prepared in accordance with published procedure [81]. Dimerization of hex-1-ene was conducted in liquid olefin media in the presence of **1**, activated by TIBA and MMAO-12 using the protocol described previously [47,49], 5-methyleneundecane was separated by the vacuum distillation, b. p. 80 °C at 7 Torr. 

### 2.3. The Alder-Ene Reaction of MA with 5-Methyleneundecane

5-Methyleneundecane (4.03 g, 24 mmol) and MA (2.45 g, 25 mmol) were placed in round-bottom flask, prefilled with argon. Mesitylene (10 mL) was added, and the mixture was heated (130 °C) with stirring within 8 h. The formation of the product of Alder-ene reaction was controlled by GC. The product was separated by distillation (B. p. 133–138 °C/0.1 Torr). The yield was 5.37 g (84%). The control experiments were performed with the same reagent and solvent loading at 80 °C. After 4 h of stirring, the sample of the reaction mixture was analyzed by ^1^H NMR spectroscopy (see Supplementary Figure S1). The products of Alder-ene reaction were not detected. 

### 2.4. Copolymerization of MA and 5-Methyleneundecane

Briefly, 5-methyleneundecane (4.03 g, 24 mmol), MA (2.35 g, 24 mmol), and toluene (10 mL) were placed in a round-bottom flask, prefilled with argon (note that freshly recrystallized MA should be used, the presence of the traces of the maleic acid leads to the isomerization of 5-methyleneundecane). The mixture was heated to 80 °C with stirring, and 0.24 mL of 1M solution of AIBN in toluene (0.24 mmol) was added. After 4 h of stirring, the mixture was cooled, the solvent was removed under reduced pressure. The residue was washed by methanol, and dried in vacuo. The yield of the product was 5.0 g (78%), white powder. For C_16_H_26_O_3_ calculated, %: C 72.14, H 9.84, O 18.02; found, %: C 72.21, H 10.03, O 17.76. For NMR spectra, see Supplementary Figures S2–S4.

In parallel, we performed the controlled experiments on homopolymerization of MA and 5-methyleneundecane, initiated at 80 °C by AIBN. After 2 h of heating, no sign of the reaction appeared. In the presence of benzoyl peroxide at 100 °C, we detected the formation of the traces of MA homopolymer, while 5-methyleneundecane was inert. 

## 3. Results

### 3.1. MA–Olefin Association

**Figure 1:**
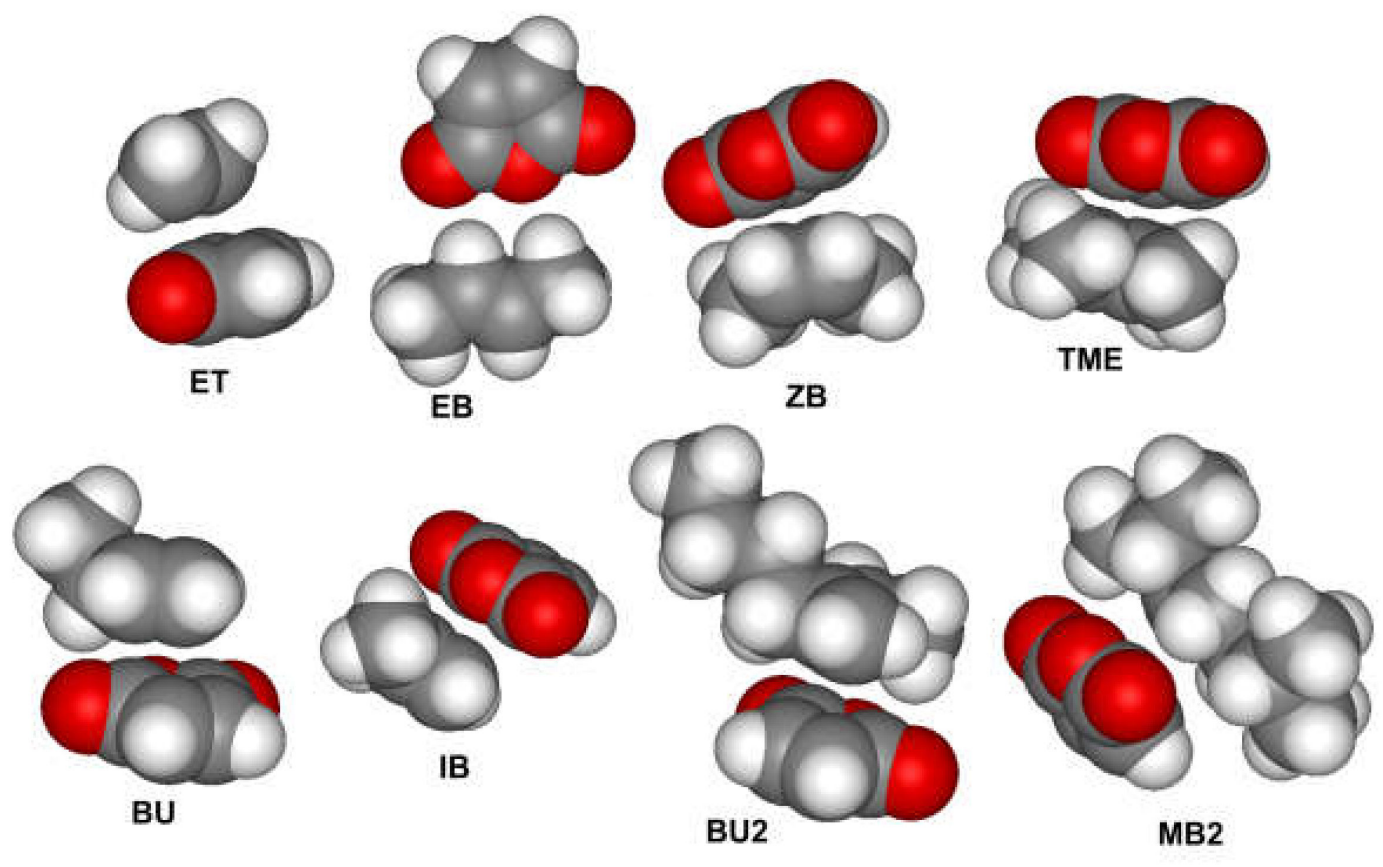
Optimized geometries of the adducts of MA and olefins.

The charge-transfer interaction between donor and acceptor monomers, called “templating”, was proposed as a factor that facilitates the alternating polymerization [1,8]. To confirm or refute this assumption for MA–olefin copolymerization [82], we analyzed the interactions between MA and olefins presented in Scheme 3. The complexes formed are substantially different by geometry as interatomic distances between MA and olefin molecules exceeded the sum of the Van-der-Vaals radii (Figure 1). Even in gas phase, the values of the calculated free energies and free enthalpies of the formation of these complexes (ΔG^f^ and ΔH^f^, see Table 1) suggest that such an association can hardly be expected to carry much weight for the interaction of MA with olefins.

### 3.2. The Alder-Ene Reaction between MA and Methylenealkanes

**Figure 2:**
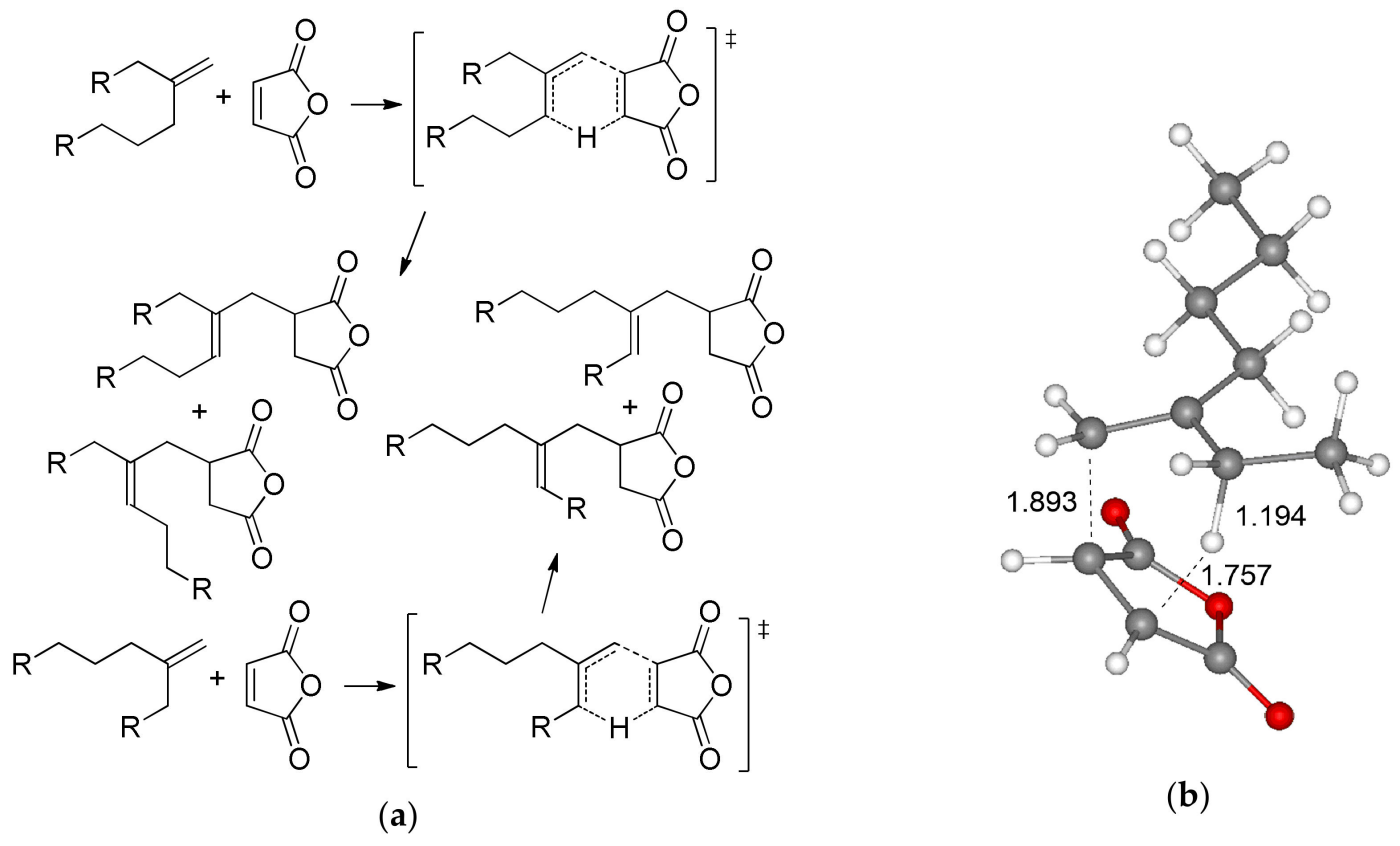
(**a**) The Alder-ene reaction between MA and methylenealkanes; (**b**) Optimized geometry of the transition state of the Alder-ene reaction between MA and 3-methyleneheptane (BU2).

Before the detailed analysis of radical copolymerization of MA and olefins, the possible side process, namely, thermally induced Alder-ene reaction with a formation of isomeric alkenyl-substituted succinic anhydrides (Figure 2a) should have been taken into account. We optimized four possible transition states (TS) of the ene reaction between MA and BU2, found the TS with minimal free energy (Figure 2b), and calculated the differences between the free energies and free enthalpies of this transition state and MA–BU2 complex. The value of ΔG^≠^ (33.9 kcal/mol, respectively) suggested that this reaction may proceed only at elevated temperatures.

**Figure 3:**
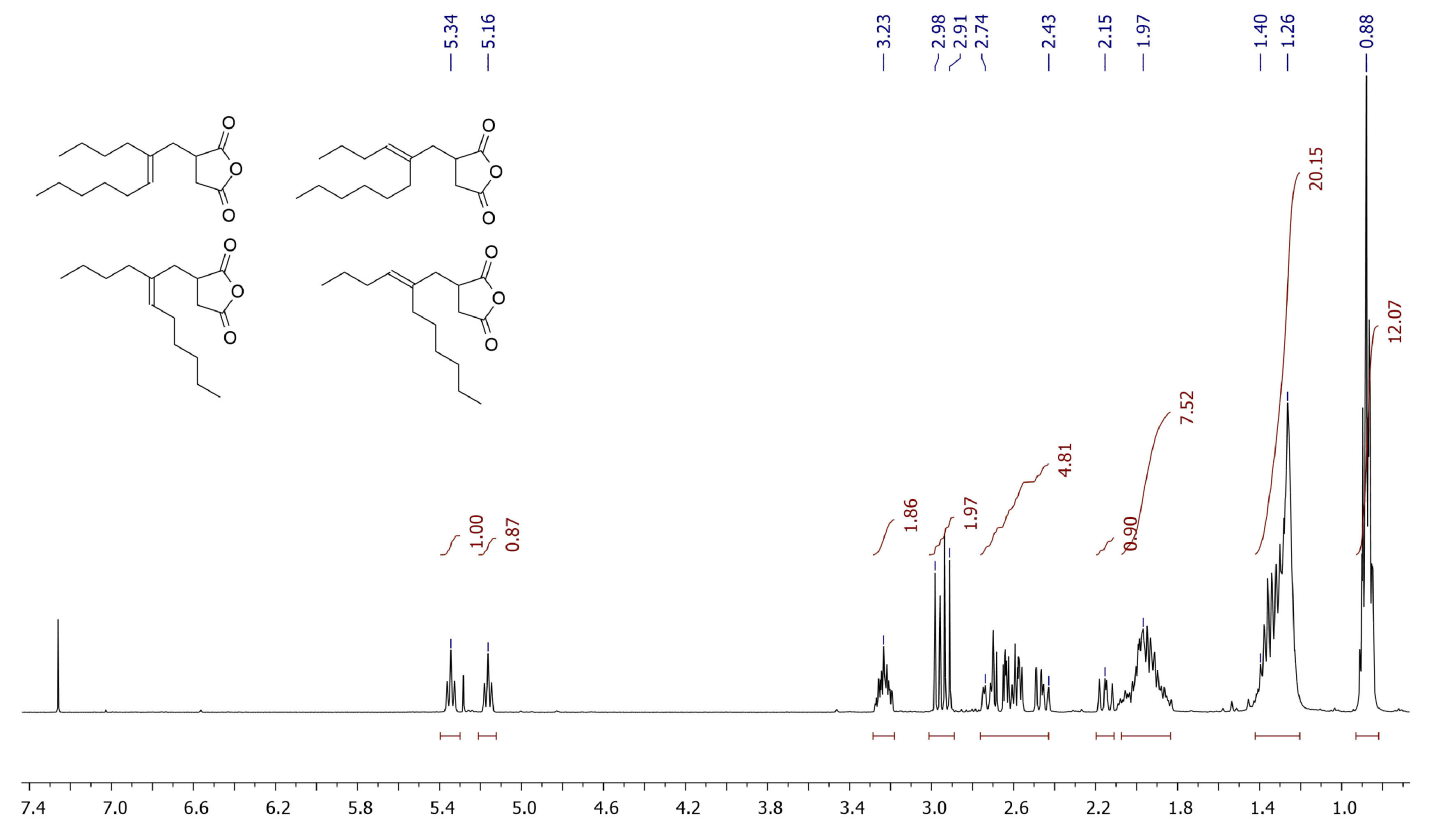
^1^H NMR spectrum (CDCl_3_, 20 °C, 400 MHz) of the products of the Alder-ene reaction between MA and 5-methyleneundecane. Volatile product of the isomerization of the starting olefin and MA were preliminarily eliminated at 0.01 Torr and 100 °C.

Our previous studies of free-radical copolymerization of MA with methylenealkanes [46] and comparative experiment (see Section 2.4) were conducted at 80 °C in order to provide high efficiency of the free-radical initiator AIBN. Our experiments with MA and 5-methyleneundecane (dimer of 1-hexene) in the absence of AIBN demonstrated that the temperature of 80 °C was not high enough to drive Alder-ene reaction (Section 2.4, Supplementary Figure S1). In fact, ~95% conversion of the reagents was achieved after 8 h heating at 130 °C, and the reaction resulted in the formation of the mixture of isomeric adducts (see Figure 3 for ^1^H NMR spectrum).

**Figure 4:**
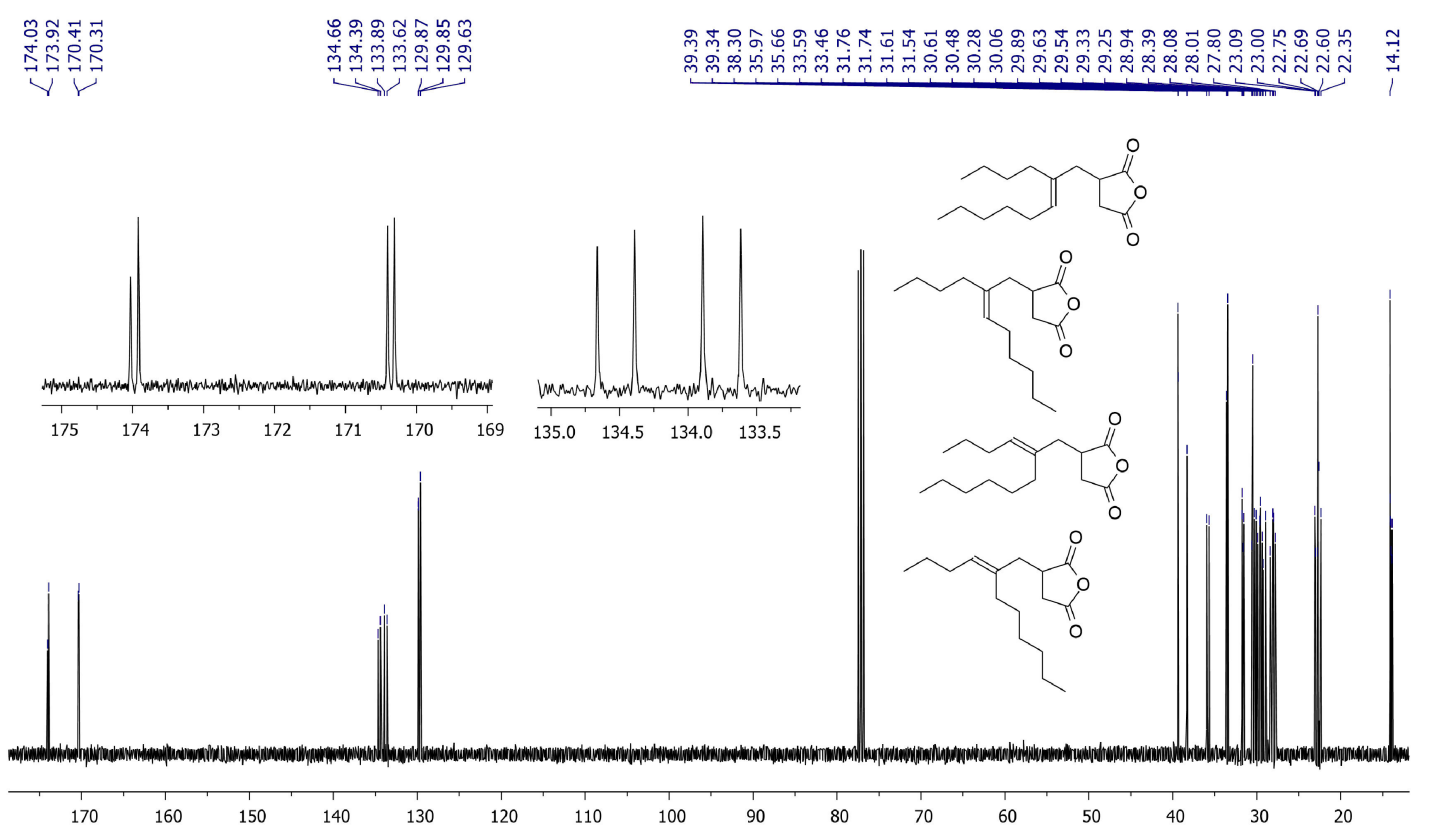
^13^C NMR spectrum (CDCl_3_, 20 °C, 400 MHz) of the products of the Alder-ene reaction between MA and 5-methyleneundecane.

For non-symmetrical methylenealkanes, eight isomeric addicts can be formed in Alder-ene reaction. ^13^C NMR spectrum of the reaction mixture (Figure 4) indicated the presence of the four groups of signals that reflect the formation of all possible isomers. We optimized all configurations of the TS (Supplementary Section S3) and found that the gap of the free energies for these transition states did not exceed 1.8 kcal/mol.

### 3.3. DFT Modeling of the Copolymerization of MA and Olefins

**Scheme 4:**
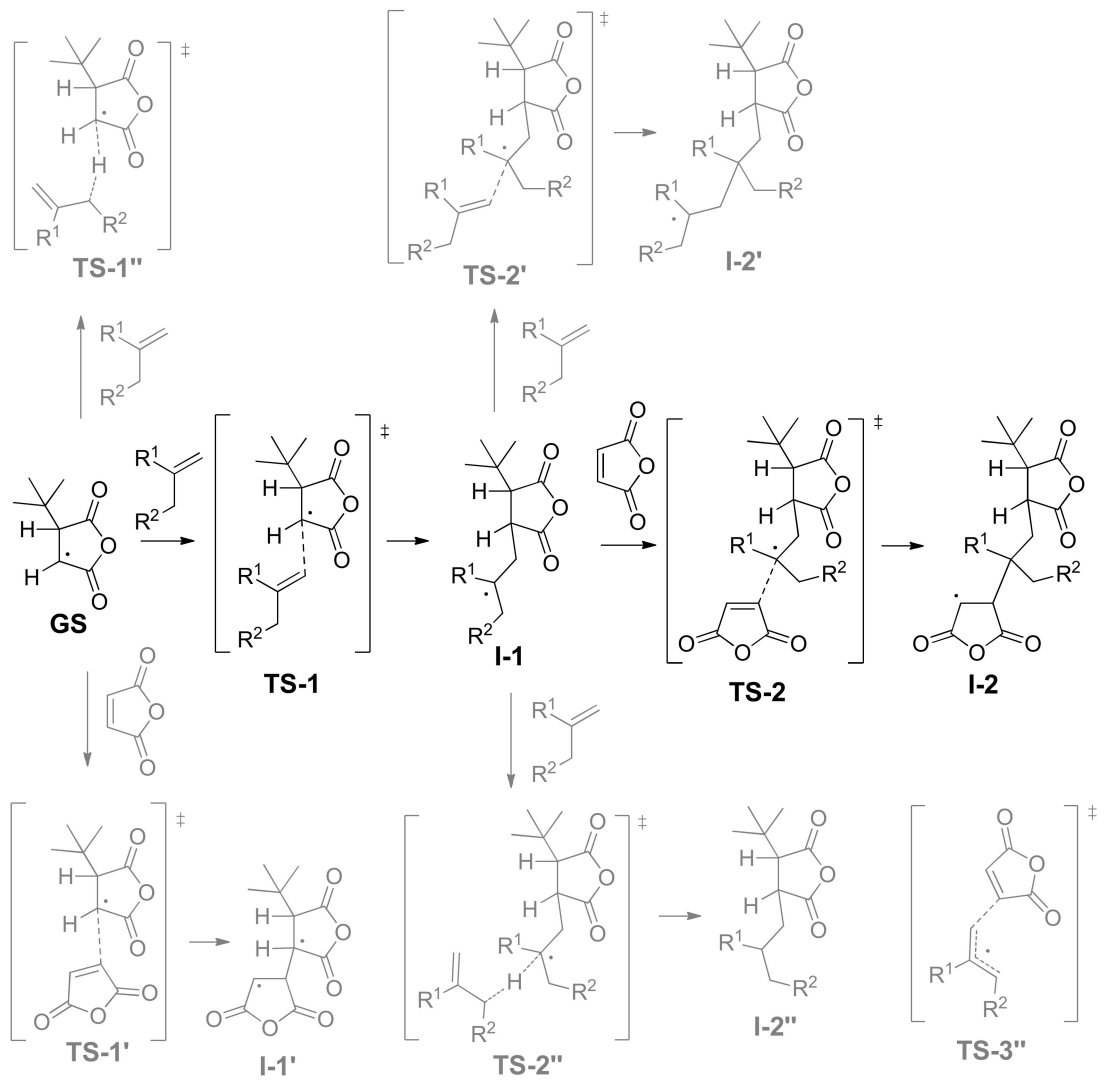
The main (marked in black) and side (marked in gray) model reactions for radical copolymerization of MA with olefins.

We used 3-(*tert*-butyl)dihydrofuran-2,5-dione radical (*^t^*Bu-MA·), the product of the reaction of MA with *tert*-butyl radical, as a ground state (GS) for the complex analysis of the reaction profiles of the free-radical copolymerization of MA with olefins (Scheme 4). There are four possible reaction pathways for *^t^*Bu-MA·:

1. The addition to olefin molecule via TS-1 with a formation of *^t^*Bu-MA-S· species (I-1, Scheme 4). This reaction pathway represents the first step of alternating polymerization.
2. The addition to MA molecule via TS-1’ with a formation of *^t^*Bu-MA-MA· species (I-1’, Scheme 4). This reaction pathway mimics homopolymerization of MA. The calculated ΔG^≠^ and ΔH^≠^ for the formation of MA homopolymer were found to be 17.7 and 5.2 kcal/mol, respectively.
3. The hydrogen transfer from *^t^*Bu-MA· to MA molecule with a formation of 3-(*tert*-butyl)dihydrofuran-2,5-dione and MA-H·. The values of ΔG^≠^ and ΔH^≠^ for this reaction were found to be 32.9 and 20.8 kcal/mol, respectively.
4. The transfer of the allyl hydrogen atom of the olefin molecule to *^t^*Bu-MA· with a formation of allyl radical and *^t^*Bu-MA-H. The energies of the corresponding TS-1’’ (Scheme 4) depend on the nature of the olefin (see Table 2). Evidently, the possibility of the further reactions of the allyl radicals should also be taken into account (see below).

*^t^*Bu-MA-S· species, formed at the first stage, can be involved into three different processes:

1. The addition to MA molecule via TS-2 with a formation of *^t^*Bu-MA-S-MA· species that are structurally similar to *^t^*Bu-MA·. This model reaction mimics the second step of the polymerization with a formation of the product with alternating microstructure. Therefore, the sequence *^t^*Bu-MA· – *^t^*Bu-MA-S· – *^t^*Bu-MA-S-MA· represents an adequate model of the alternating polymerization of MA with olefins.
2. The addition to olefin molecule via TS-2’ with a formation of *^t^*Bu-MA-S-S· species (I-2’, Scheme 4). This is the side reaction that reflects the possibility of the formation of oligoolefin blocks.
3. The transfer of the allyl hydrogen atom of the olefin molecule to *^t^*Bu-MA-S· (TS-2’’) with a formation of allyl radical and *^t^*Bu-MA-SH.

The detailed analysis of the transition states and stationary points presented in Scheme 4 needed consideration of all possible orientations of the molecules of reagents. We optimized the structures of these TS and intermediates (the results of this analysis are given in the Supporting Information), and found the optimal orientations for all olefins studied. The values of the relative free energies and relative free enthalpies for such optimized species are presented in Table 2.

**Figure 5:**
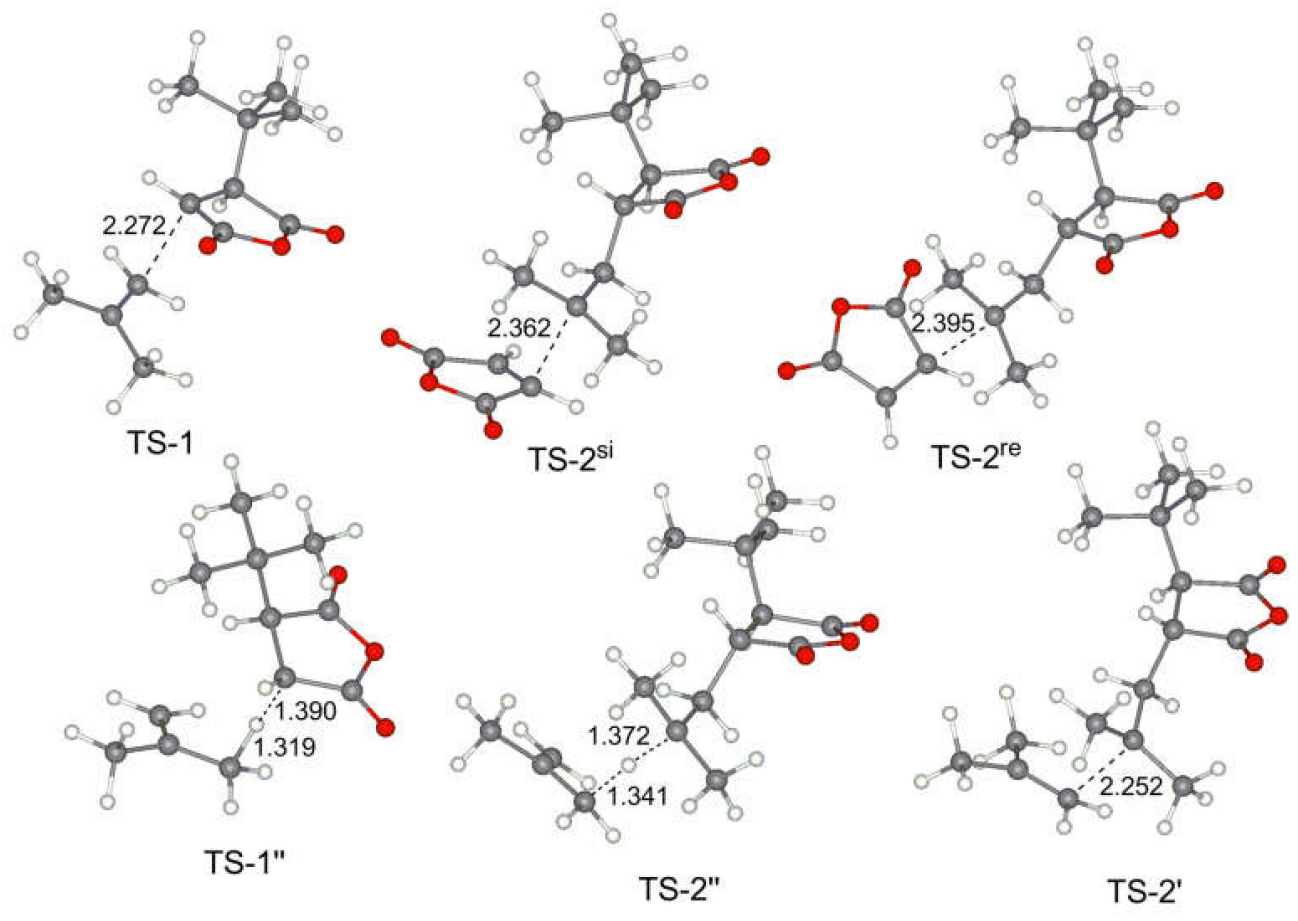
Calculated geometries of the key transition states for MA–IB copolymerization.

The geometries of the key transition states and stationary points for the reaction of MA with isobutylene IB are presented in Figure 5. We note that the interatomic distances in these TS correlate with relative energies, and the steric hindrance in the early transition states TS-1 with the participance of *tert*-alkyl radical are not significant.

The relative values of the transition states of hydrogen transfer from olefin to *^t^*Bu-MA· (TS-1’’) and to *^t^*Bu-MA-S· (TS-2’’) were only slightly higher than the values of TS-1 (Table 2), therefore, such transfer processes seem to be plausible pathways of the chain release. The calculated relative free energies of TS-3’’ for IB, OCT, and BU2 (16.4, 17.3 and 18.1 kcal/mol, respectively) indicate that allyl radical species also can initiate copolymerization.

### 3.4. DFT Modeling of the Copolymerization of MMI and Olefins

**Figure 6:**
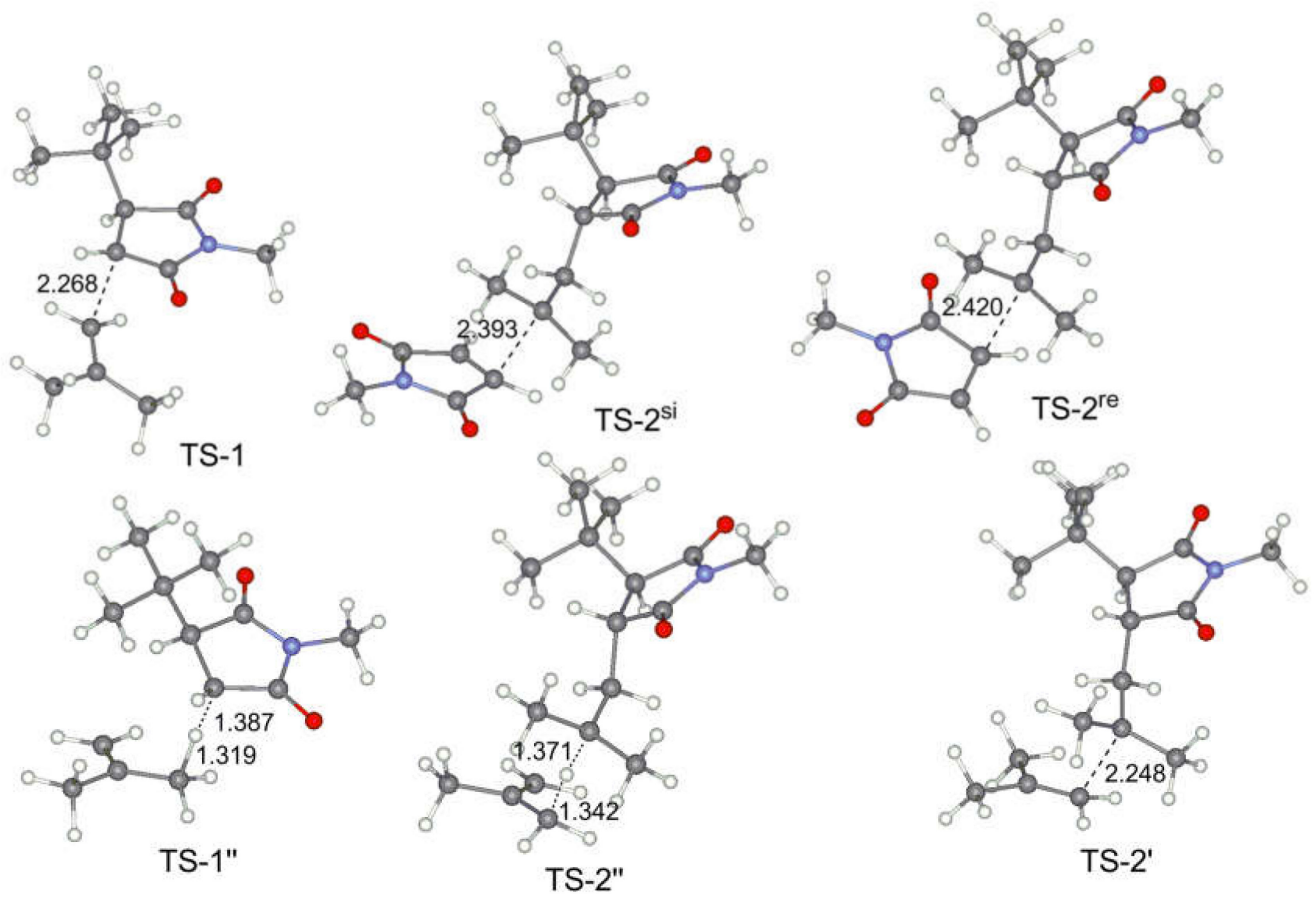
Calculated geometries of the key transition states for MMI–IB copolymerization.

To compare the reaction ability of the close analog of maleic anhydride, *N*-methylmaleimide, we have chosen two olefins, namely, ethylene ET and isobutylene IB. *tert*-Butyl substituted radical (*^t^*Bu-MMI·), closely related to *^t^*Bu-MA·, was used as a model of the active polymer chain. The results of calculations are presented in Table 3 and in Figure 6.

## 4. Discussion

**Figure 7:**
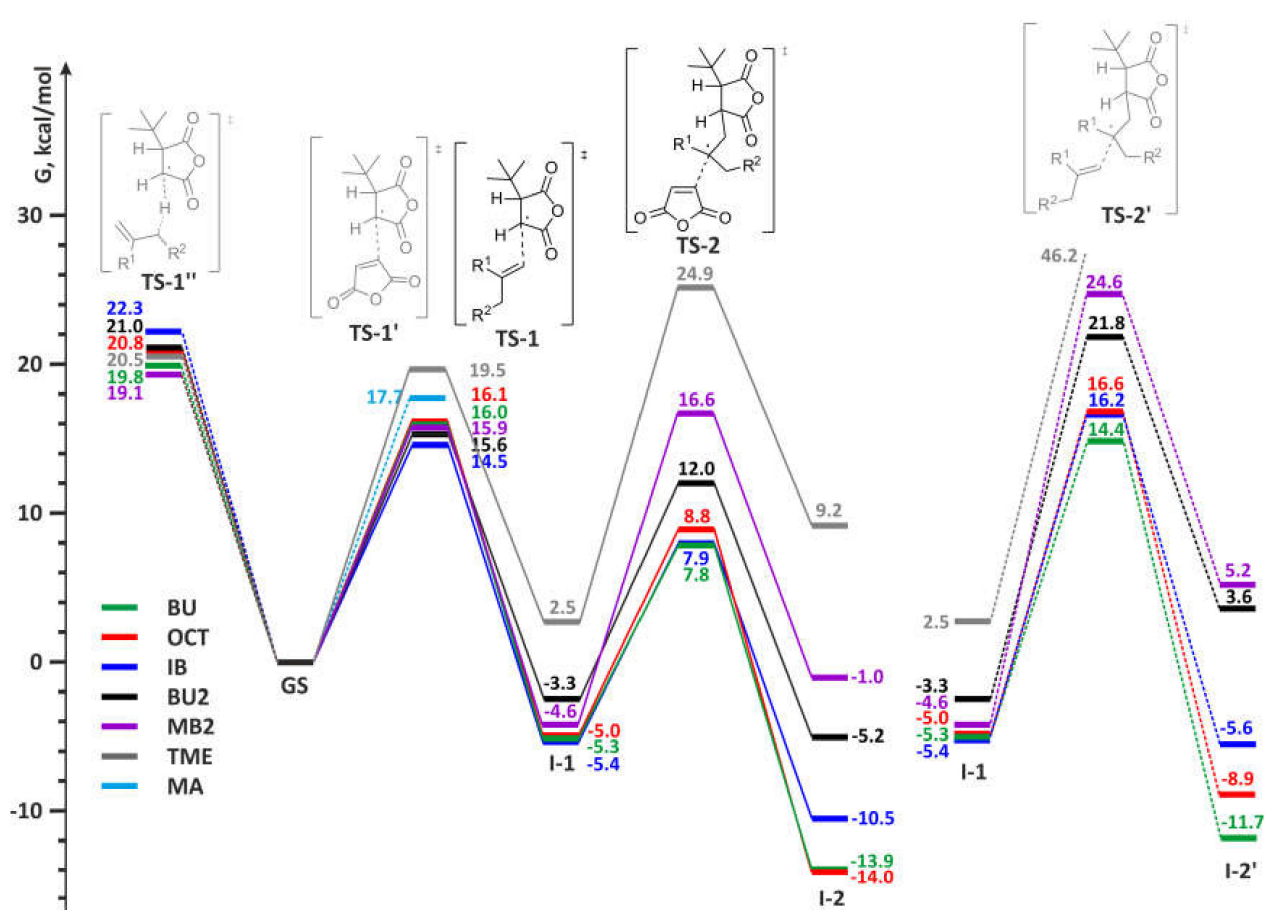
Free energy profiles for main (left) and side (right) reactions during radical copolymerization of MA and olefins.

Some examples of the energy profiles for the main and side reactions during MA–olefin copolymerization are presented in Figure 7.

The free activation energies for the first step of alternating copolymerization, the reaction of *^t^*Bu-MA· with olefins, substantially depend on the type of the olefin. This barrier is lower for isobutylene and methylenealkanes (14.5–15.9 kcal/mol), a little higher for α-olefins (16.0 kcal/mol) but considerably higher for internal olefins. For 2-butenes and tetramethylethylene the values of ΔG of TS-1 (~18.5 and 19.5 kcal/mol, respectively) exceeded the activation barrier of MA homopolymerization, therefore, the formation of copolymers for these olefins should be complicated by the formation of poly(MA) blocks. At the second stage of MA-olefin copolymerization, both MA and olefin molecule can react with alkyl radical formed at the first stage. For all olefins, the reaction with MA at the second stage is preferable, but the difference in free energies in chain propagation by MA and olefin is minimal for ethylene. The formation of 1,4-butylene fragments can therefore complicate MA-ethylene copolymerization under the high ethylene pressure. The result of the calculations are in agreement with early published data on the copolymerization of MA with different olefins [3,4,9,13,83,84], in particular, with low reactivity of 2-butenes and full inertness of TME towards MA in the presence of free-radical initiators [85].

In addition, calculated values of the activation barriers at 353 K for Alder-ene reaction (36.5 kcal/mol) and alternating polymerization for MA–BU2 pair (17.4 and 16.9 kcal/mol for BU2 and MA insertions, respectively), correspond the results of our experiments: at 80 °C, the reaction between MA and 5-methyleneundecane, close structural analog of BU2, was not observed. However, in the presence of AIBN, a copolymer with 1:1 MA/olefin ratio was formed with satisfactory yield. 

From the data reported in Table 2 and Table 3 and Figure 5 and Figure 6, it can be concluded that there is no marked difference between MA and MMI in reactions with olefin molecules. However, a substantial difference was detected when the activation barriers of MA and MMI homopolymerization were compared. AS was mentioned above, for MA the addition of the olefin molecule to *^t^*Bu-MA· is preferable for α-olefins and methylenealkanes, including isobutylene, and slightly preferable for ethylene and sterically hindered methylenealkane MB2. However, for MMI and IB the activation barriers of homopolymerization and copolymerization are close. Omayu and Matsumoto [75] estimated the activation barrier of the reaction of model radical Me-MMI· with IB by the value of 14.6 kcal/mol. Our estimation of the activation barrier of this stage for *^t^*Bu-MMI· was 15.9 kcal/mol, but at the same time, the ΔG^≠^ for MMI homopolymerization was 15.8 kcal/mol.

Note that the calculated activation barrier for the reaction of *^t^*Bu-MA· with IB was 14.5 kcal/mol, the activation barrier of the second step of the alternating copolymer formation was also substantially lower for MA–IB in comparison with MMI–IB (13.3 vs. 14.7 kcal/mol). Therefore, MMI fell behind MA as a comonomer in all respects.

## 5. Conclusions

The results of the comparative DFT calculations of the reaction profiles for maleic anhydride (MA) and different alkenes predicted the preference of the formation of alternating copolymer when MA was chosen in combination with α-olefins and methylenealkanes. For internal olefins, the formation of the alternating copolymer was found to be energetically unfavorable.

Bearing in mind the clear prospects of application for maleimide-based alternating copolymers with olefins, we analyzed the reaction profiles of the copolymerization of N-methylmaleimide (MMI) with ethylene and isobutylene. The results of our calculations predicted a higher propensity of maleimide to homopolymerization. Therefore, a more efficient and secure synthetic approach to such copolymers is, in our view, post-modification (imidization) of the alternating copolymers based on maleic anhydride.

The results of our calculations, performed for a wide range of olefins, might be used as a basis for the thorough theoretical analysis of other combinations of donor and acceptor monomers in the design of prospective macromolecules with alternating microstructures.

## Figures and Tables

**Table 1:** Calculated free energies and free enthalpies of the formation of MA-olefin complexes.

Olefin	ΔG^f^	ΔH^f^
ET	5.80	–0.22
Bu	6.97	–0.61
EB	6.79	–0.81
ZB	7.02	–0.42
IB	7.09	–1.05
TME	8.10	–0.46
BU2	7.67	–1.05
MB2	6.52	–0.82

**Table 2:** Calculated free energies and free enthalpies of the key intermediated and transition states for MA–olefin copolymerization.

Olefin		TS-1	I-1	TS-2	I-2	TS-2’	I-2’	TS-1’’	TS-2’’
**ET**	ΔG	17.21	–5.14	7.21	–22.84	10.77	–19.78	–	–
	ΔH	5.77	–16.98	–16.27	–47.70	–11.71	–42.64	–	–
**BU**	ΔG	16.05	–5.34	7.82	–13.95	14.49	–11.69	19.84	16.79
	ΔH	4.49	–16.97	–16.40	–39.87	-9.48	–35.86	8.51	–6.99
**OCT**	ΔG	16.10	–4.96	8.75	–13.98	16.58	–8.94	20.76	19.59
	ΔH	4.35	–16.94	–15.99	--40.05	–8.43	–36.57	8.64	–5.06
**ZB**	ΔG	18.70	–0.52	11.72	–11.11	20.12	–1.89	20.80	–
	ΔH	5.83	–13.86	–14.36	–37.71	–3.11	–28.33	9.08	–
**EB**	ΔG	18.47	–1.24	12.15	–12.15	21.82	–0.40	20.90	–
	ΔH	6.15	–13.70	–13.38	–38.45	–3.49	–26.98	9.56	–
**IB**	ΔG	14.50	–5.41	7.86	–10.50	16.20	–5.57	22.25	20.39
	ΔH	2.96	–17.26	–17.95	–37.20	–9.24	–31.30	11.13	–3.92
**BU2**	ΔG	15.57	–3.33	11.98	–5.15	21.76	3.56	21.03	24.85
	ΔH	3.03	–16.30	–14.86	–32.71	–4.31	–24.61	8.90	–1.49
**MB2**	ΔG	15.93	–4.60	16.41	–1.00	24.57	5.19	19.06	–
	ΔH	3.22	–16.76	–10.45	–28.30	–2.53	–22.73	7.58	–
**TME**	ΔG	19.47	2.49	23.94	9.17	46.60	34.28	20.47	–
	ΔH	6.80	–11.81	–4.52	–20.80	16.33	3.01	9.86	–
**MA**	ΔG	17.71 ^1^	–5.70 ^1^	–	–	32.88 ^2^	–	–	–
	ΔH	5.54 ^1^	–18.97 ^1^	–	–	20.78 ^2^	–	–	–

^1^ For MA, the energy values of TS-1’ and I-1’ are presented. ^2^ ΔG and ΔH of the transition state of the hydrogen transfer from *^t^*Bu-MA· to MA.

**Table 3:** Calculated free energies and free enthalpies of the key intermediated and transition states for MMI–olefin copolymerization.

Olefin		TS-1	I-1	TS-2	I-2	TS-1’’	TS-2’	I-2’
**ET**	ΔG	18.00	–5.22	7.57	–25.98	–	11.19	–19.50
	ΔH	6.68	–17.53	–16.42	–51.07	–	–12.05	–42.99
**IB**	ΔG	15.92	–5.48	9.21	–12.95	23.39	15.54	–6.30
	ΔH	4.52	–17.48	–17.41	-40.15	11.99	–9.46	–31.42
**MMI**	ΔG	15.82 ^1^	–10.27 ^1^	–	–	–	32.76 ^2^	–
	ΔH	4.34 ^1^	–23.50 ^1^	–	–	–	20.72 ^2^	–

^1^ For MMI, the energy values of TS-1’ and I-1’ are presented. ^2^ The relative energies pf the transition state of the hydrogen transfer from–*^t^*Bu-MMI· to MMI.

## Supplementary Materials

The following are available online at https://www.mdpi.com/2073-4360/12/4/744/s1, Figure S1: 1H NMR spectrum (CDCl_3_, 20 °C, 400 MHz) of the mixture of MA and 5-methylene-undecane after 2 h at 80 °C, Figure S2: 1H NMR spectrum (CDCl_3_, 20 °C, 400 MHz) of the copolymer of MA and 5-methyleneundecane, Figure S3: 1H NMR spectrum (CDCl_3_, 20 °C, 101 MHz) of the copolymer of MA and 5-methyleneundecane, Figure S4: 1H-^13^C correlation NMR spectrum (CDCl_3_, 20 °C) of the copolymer of MA and 5-methyleneundecane. Optimized geometries, energy parameters and cartesian coordinates for all stationary points and transition states.
