# Supplementary material for: DFT Modeling of the Alternating Radical Copolymerization and Alder-Ene Reaction between Maleic Anhydride and Olefins
Source: Polymers (Basel). 2020 Mar 27;12(4):744. doi: 10.3390/polym12040744 (PMC7240384; doi:10.3390/polym12040744)
Supplement: Supplementary file 1 [file polymers-12-00744-s001.pdf]

DFT modeling of the alternating radical copolymerization and Alder-ene reaction between maleic anhydride and olefins

Ilya Nifant'ev [1,2,*], Alexander Vinogradov [1], Alexey Vinogradov [1] and Pavel Ivchenko [1,2]

[1] A.V. Topchiev Institute of Petrochemical Synthesis RAS, 29 Leninsky Pr., Moscow, Russia 119991; ilnif@yahoo.com (I.N.), amvvin@mail.ru (Alexander V.), vinasora@gmail.com (Alexey V.), phpasha1@yandex.ru (P.I.)

[2] Chemistry Department, M.V. Lomonosov Moscow State University, 1 Leninskie Gory Str., Building 3, Moscow, Russia 119991; inif@org.chem.msu.ru (I.N.), inpv@org.chem.msu.ru (P.I.)

* Correspondence: ilnif@yahoo.com; Tel.: +7-495-939-4098 (I.N.)

SUPPORTING INFORMATION

S1. Supplementary NMR spectra 2
S2. Ground state and monomers 4
S3. MA – olefin complexes 16
S4. Alder-ene reaction 25
S5. MA – MA reaction profile 29
S6. MA – ET reaction profile 34
S7. MA – BU reaction profile 43
S8. MA – EB reaction profile 55
S9. MA – ZB reaction profile 79
S10. MA – IB reaction profile 103
S11. MA – OCT reaction profile 116
S12. MA – BU2 reaction profile 131
S13. MA – MB2 reaction profile 145
S14. MMI – MMI reaction profile 161
S15. MMI – ET reaction profile 167
S16. MMI – IB reaction profile 177

S1. Additional NMR spectra

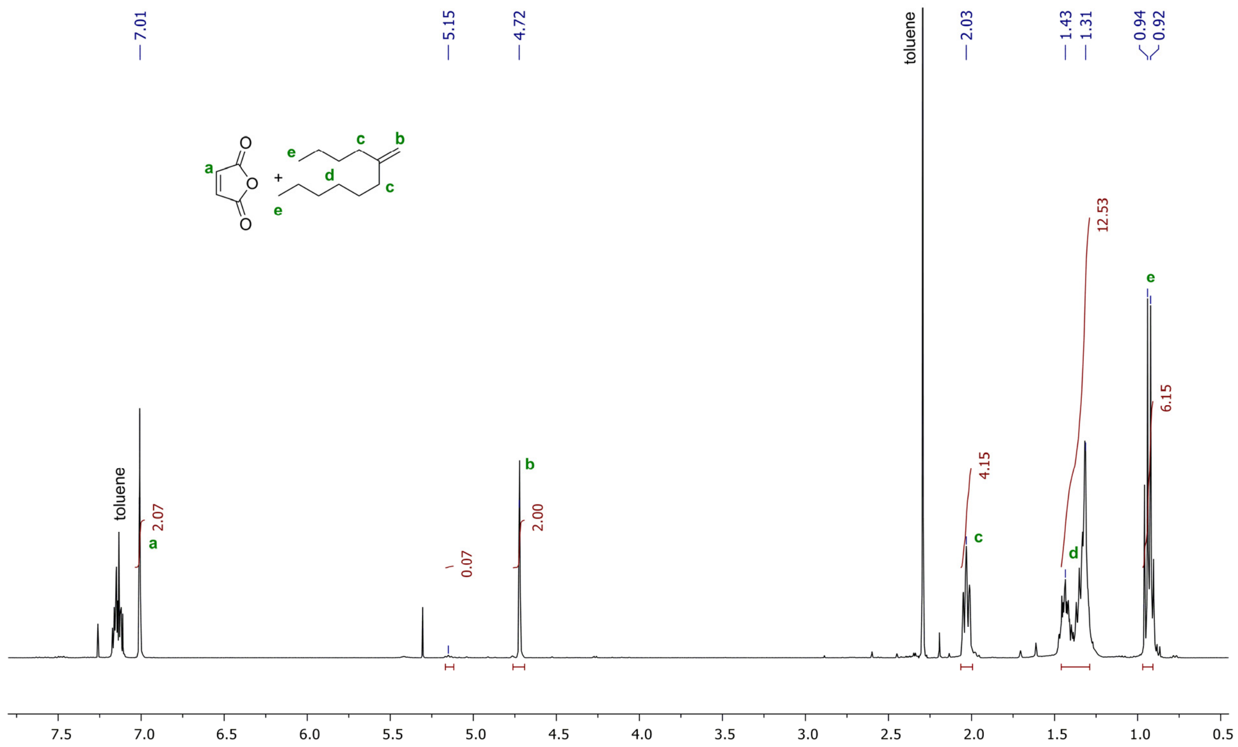

Figure S1. ^{1}H NMR spectrum ($CDCl_3$, 20 °C, 400 MHz) of the mixture of MA and 5-methylene-undecane after 2 h at 80 °C.

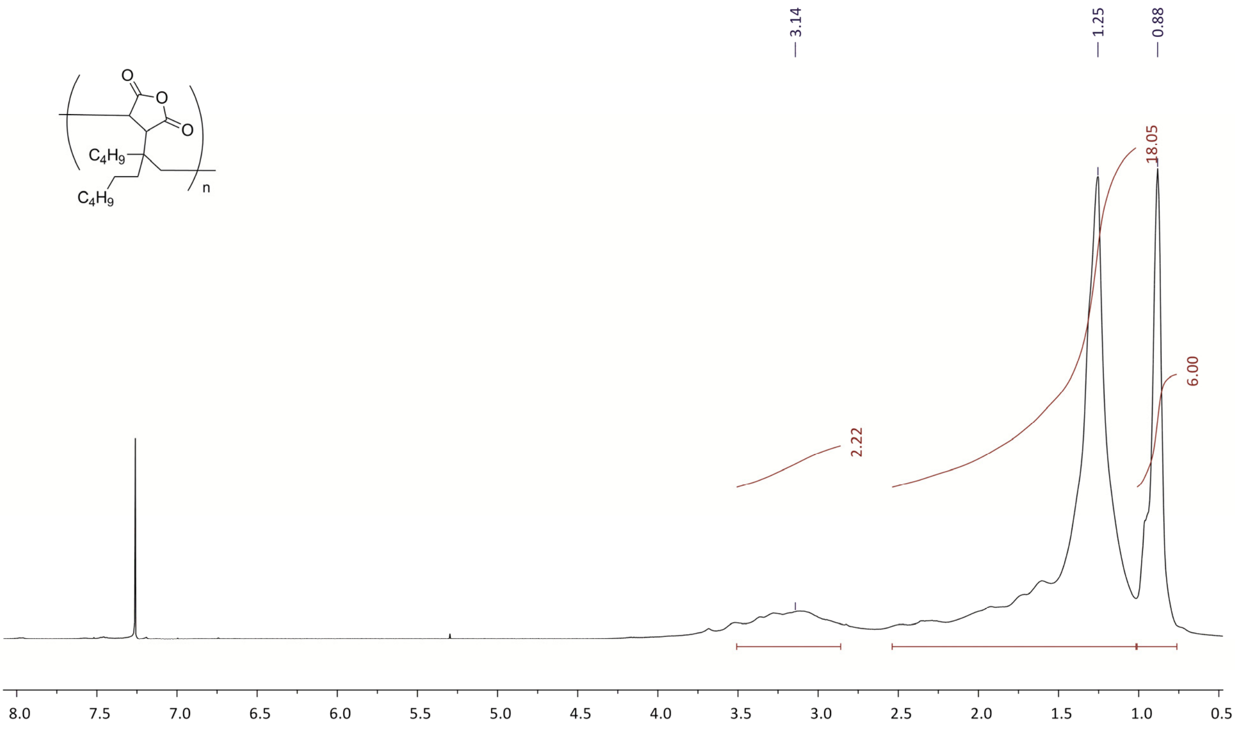

Figure S2. ^{1}H NMR spectrum ($CDCl_3$, 20 °C, 400 MHz) of the copolymer of MA and 5-methyleneundecane.

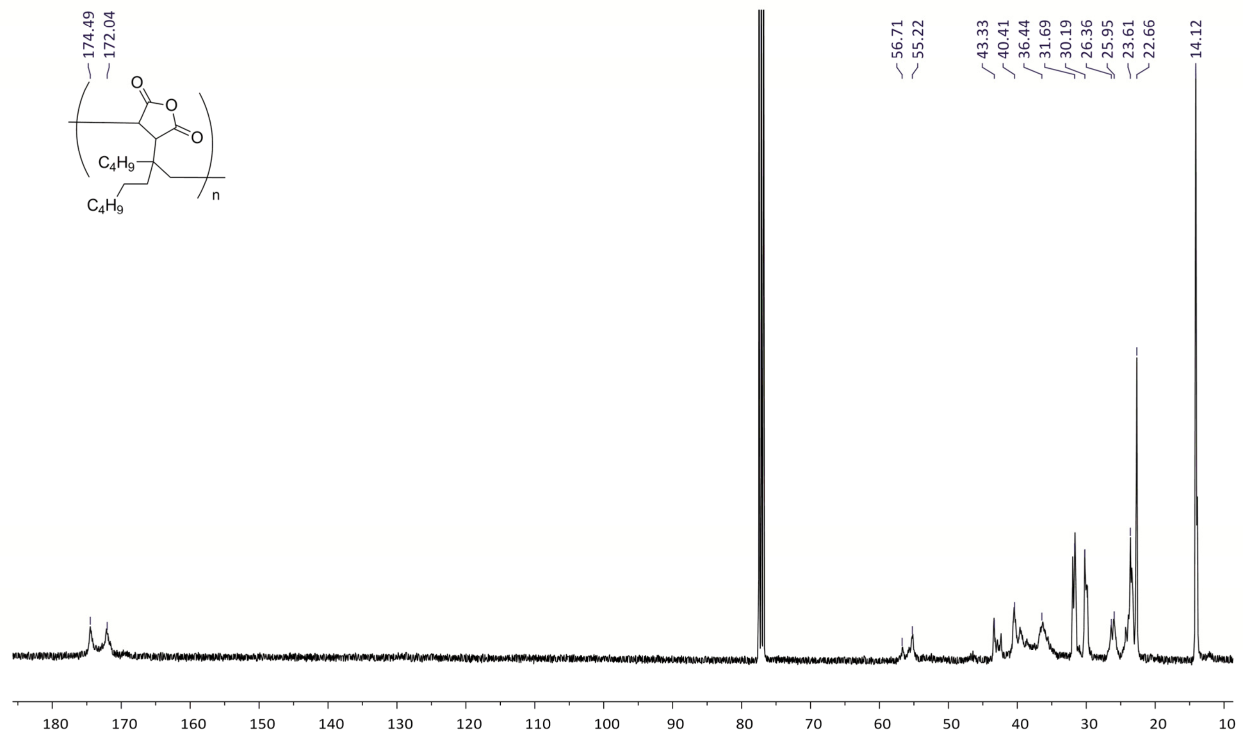

Figure S3. ^{1}H NMR spectrum ($CDCl_3$, 20 °C, 101 MHz) of the copolymer of MA and 5-methyleneundecane.

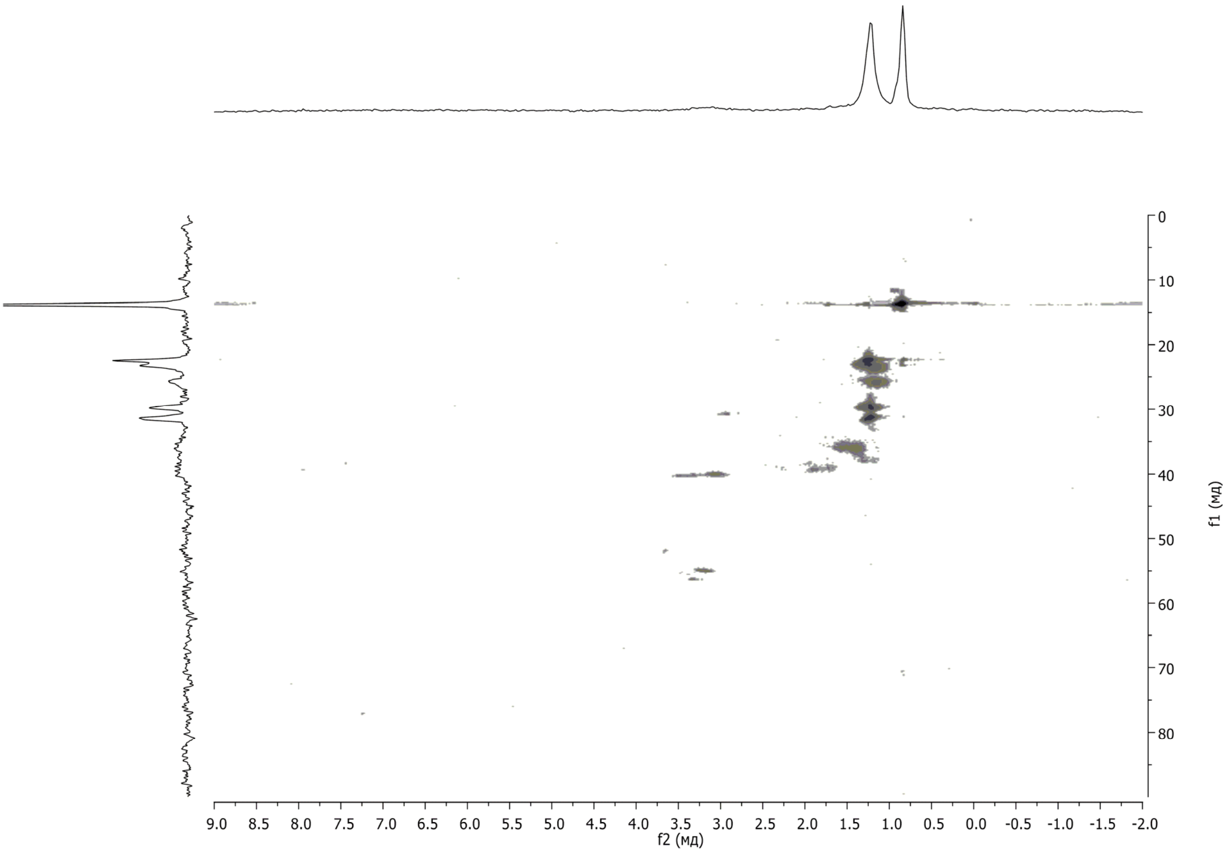

Figure S4. ^{1}H-^{13}C correlation NMR spectrum ($CDCl_3$, 20 °C) of the copolymer of MA and 5-methyleneundecane.

S2. Ground state and monomers

ET ethylene

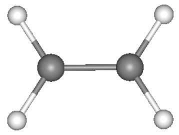

Zero-point vibrational energy	134509.6 (Joules/Mol)
	32.14857 (Kcal/Mol)
Zero-point correction=	0.051232 (Hartree/Particle)
Thermal correction to Energy=	0.054272
Thermal correction to Enthalpy=	0.055217
Thermal correction to Gibbs Free Energy=	0.029058
Sum of electronic and zero-point Energies=	-78.527592
Sum of electronic and thermal Energies=	-78.524552
Sum of electronic and thermal Enthalpies=	-78.523608
Sum of electronic and thermal Free Energies=	-78.549766 X

cartesian

6	-0.66229999	0.00000000	0.00000000	6	0.66229999	0.00000000	0.00000000
1	-1.23329997	-0.92250001	0.00000000	1	1.23329997	0.92250001	0.00000000
1	-1.23329997	0.92250001	0.00000000	1	1.23329997	-0.92250001	0.00000000

BU but-1-ene

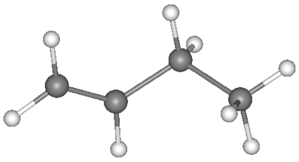

Zero-point vibrational energy	286481.1 (Joules/Mol)
	68.47063 (Kcal/Mol)
Zero-point correction=	0.109115 (Hartree/Particle)
Thermal correction to Energy=	0.114367
Thermal correction to Enthalpy=	0.115312
Thermal correction to Gibbs Free Energy=	0.081631
Sum of electronic and zero-point Energies=	-157.099983
Sum of electronic and thermal Energies=	-157.094730
Sum of electronic and thermal Enthalpies=	-157.093786
Sum of electronic and thermal Free Energies=	-157.127466

cartesian

6	0.53839999	0.51929998	0.30590001	6	1.71700001	-0.24620000	-0.29300001
6	-1.85090005	0.01700000	-0.27849999	1	1.51419997	-0.53049999	-1.32780004
1	0.36469999	1.44099998	-0.25749999	1	2.62770009	0.35699999	-0.28090000
1	0.79549998	0.82510000	1.32760000	1	1.92170000	-1.16289997	0.26629999
1	-1.94860005	0.92490000	-0.86690003	6	-0.71780002	-0.29290000	0.33950001
1	-2.72630000	-0.62029999	-0.21960001	1	-0.66949999	-1.21710002	0.91530001

EB (*E*)-but-2-ene

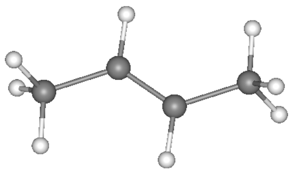

Zero-point vibrational energy	285232.3 (Joules/Mol)
	68.17215 (Kcal/Mol)
Zero-point correction=	0.108639 (Hartree/Particle)
Thermal correction to Energy=	0.114110
Thermal correction to Enthalpy=	0.115054
Thermal correction to Gibbs Free Energy=	0.081247
Sum of electronic and zero-point Energies=	-157.105889
Sum of electronic and thermal Energies=	-157.100418
Sum of electronic and thermal Enthalpies=	-157.099474
Sum of electronic and thermal Free Energies=	-157.133281

cartesian

6	-1.95280004	-0.07870000	0.00000000	6	-0.53560001	0.39340001	0.00000000
6	0.53560001	-0.39340001	0.00000000	1	-0.38830000	1.47340000	0.00000000
1	-2.01379991	-1.16869998	-0.00010000	1	-2.49510002	0.28860000	-0.87699997
1	-2.49499989	0.28839999	0.87720001	1	2.01379991	1.16869998	-0.00020000
6	1.95280004	0.07870000	0.00000000	1	2.49499989	-0.28830001	0.87720001
1	0.38830000	-1.47340000	0.00000000	1	2.49510002	-0.28870001	-0.87699997

ZB (*Z*)-but-2-ene

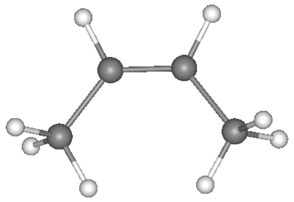

Zero-point vibrational energy	285408.2 (Joules/Mol)
	68.21419 (Kcal/Mol)
Zero-point correction=	0.108706 (Hartree/Particle)

Thermal correction to Energy=	0.114252
Thermal correction to Enthalpy=	0.115196
Thermal correction to Gibbs Free Energy=	0.080760
Sum of electronic and zero-point Energies=	-157.103807
Sum of electronic and thermal Energies=	-157.098261
Sum of electronic and thermal Enthalpies=	-157.097316
Sum of electronic and thermal Free Energies=	-157.131753

cartesian

6	1.58420002	-0.51899999	0.00010000	6	0.66600001	0.66039997	0.00000000
6	-0.66610003	0.66039997	-0.00010000	1	1.16209996	1.62909997	-0.00010000
1	1.05690002	-1.47270000	-0.00080000	1	2.23889995	-0.50269997	0.87709999
1	2.24040008	-0.50180000	-0.87570000	1	-1.05669999	-1.47259998	0.00020000
6	-1.58420002	-0.51899999	0.00000000	1	-2.23970008	-0.50220001	0.87629998
1	-1.16209996	1.62919998	-0.00020000	1	-2.23950005	-0.50250000	-0.87650001

IB Isobutylene, 2-methylprop-1-ene

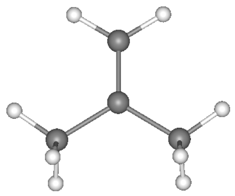

Zero-point vibrational energy	284807.5 (Joules/Mol)
	68.07062 (Kcal/Mol)
Zero-point correction=	0.108477 (Hartree/Particle)
Thermal correction to Energy=	0.113809
Thermal correction to Enthalpy=	0.114753
Thermal correction to Gibbs Free Energy=	0.081125
Sum of electronic and zero-point Energies=	-157.106278
Sum of electronic and thermal Energies=	-157.100947
Sum of electronic and thermal Enthalpies=	-157.100002
Sum of electronic and thermal Free Energies=	-157.133631

cartesian

6	0.00000000	0.12260000	0.00000000	1	-1.32249999	-1.32920003	0.87709999
6	0.00000000	1.45319998	-0.00010000	1	-2.15359998	-0.03690000	-0.00010000
1	0.92360002	2.02259994	-0.00010000	6	1.26979995	-0.67570001	0.00000000
1	-0.92360002	2.02259994	-0.00010000	1	1.32249999	-1.32920003	0.87709999
6	-1.26979995	-0.67570001	0.00000000	1	1.32239997	-1.32930005	-0.87699997
1	-1.32239997	-1.32930005	-0.87699997	1	2.15359998	-0.03690000	0.00000000

IB_rad 2-methylprop-1-en-3-yl radical

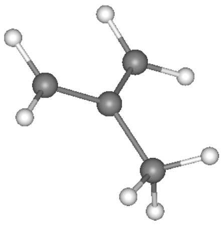

Zero-point vibrational energy	247435.4 (Joules/Mol)
	59.13847 (Kcal/Mol)
Zero-point correction=	0.094243 (Hartree/Particle)
Thermal correction to Energy=	0.098694
Thermal correction to Enthalpy=	0.099638
Thermal correction to Gibbs Free Energy=	0.067272
Sum of electronic and zero-point Energies=	-156.471143
Sum of electronic and thermal Energies=	-156.466692
Sum of electronic and thermal Enthalpies=	-156.465748
Sum of electronic and thermal Free Energies=	-156.498114

cartesian

6	-0.73930001	1.22370005	-0.00020000	1	-0.29960001	-2.15059996	-0.00210000
6	-0.09010000	-0.00410000	-0.00110000	1	-1.88269997	-1.19280005	0.00260000
6	1.41919994	-0.03080000	0.00040000	1	-1.82110000	1.28470004	0.00290000
6	-0.79879999	-1.18929994	-0.00020000	1	-0.18850000	2.15669990	-0.00210000
1	1.82330000	0.51990002	-0.85219997	1	1.80429995	-1.04960001	-0.04760000
1	1.81830001	0.43439999	0.90499997				

TME 2,3-dimethylbut-2-ene

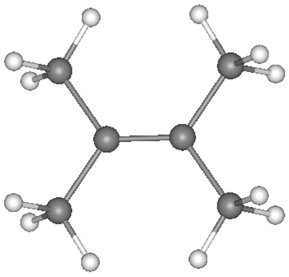

Zero-point vibrational energy	432255.4 (Joules/Mol)
	432255.4 (Kcal/Mol)
Zero-point correction=	0.164637 (Hartree/Particle)
Thermal correction to Energy=	0.172544
Thermal correction to Enthalpy=	0.173488
Thermal correction to Gibbs Free Energy=	0.132554
Sum of electronic and zero-point Energies=	-235.675991
Sum of electronic and thermal Energies=	-235.668084

Sum of electronic and thermal Enthalpies=	-235.667140
Sum of electronic and thermal Free Energies=	-235.708074

cartesian

6	1.52020001	-1.24230003	0.00000000	1	-2.17849994	-1.25559998	-0.87570000
6	0.67140001	0.00000000	0.00000000	1	-0.95859998	-2.17319989	0.00010000
6	-0.67140001	0.00000000	0.00000000	1	2.17820001	-1.25580001	-0.87589997
1	0.95859998	-2.17319989	0.00060000	1	-2.17840004	1.25559998	0.87580001
6	-1.52020001	1.24230003	0.00000000	1	-0.95859998	2.17319989	-0.00010000
6	-1.52020001	-1.24230003	0.00000000	1	-2.17869997	1.25549996	-0.87550002
1	2.17890000	-1.25520003	0.87540001	1	0.95859998	2.17319989	-0.00070000
6	1.52020001	1.24230003	0.00000000	1	2.17810011	1.25580001	0.87589997
1	-2.17860007	-1.25549996	0.87559998	1	2.17899990	1.25520003	-0.87540001

BU2 3-methyleneheptane

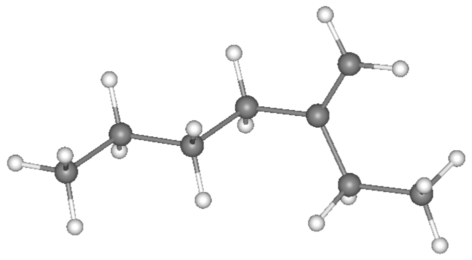

Zero-point vibrational energy	587755.4 (Joules/Mol)
	140.47691 (Kcal/Mol)
Zero-point correction=	0.223864 (Hartree/Particle)
Thermal correction to Energy=	0.234314
Thermal correction to Enthalpy=	0.235258
Thermal correction to Gibbs Free Energy=	0.187303
Sum of electronic and zero-point Energies=	-314.237640
Sum of electronic and thermal Energies=	-314.227190
Sum of electronic and thermal Enthalpies=	-314.226246
Sum of electronic and thermal Free Energies=	-314.274202

cartesian

6	1.84189999	-0.84350002	0.31060001	1	-0.43779999	1.74230003	0.65200001
6	1.22189999	0.51520002	0.10980000	1	-1.18850005	0.25720000	-1.22459996
6	-0.18300000	0.67820001	0.63169998	1	-1.02810001	-1.14359999	-0.18600000
6	-1.24460006	-0.06950000	-0.17990001	1	-2.88039994	1.22179997	0.35339999
6	-2.65720010	0.14850000	0.34940001	6	-3.71690011	-0.58910000	-0.45649999
6	1.85430002	1.52559996	-0.48400000	1	-4.71869993	-0.41720000	-0.05630000
6	3.26580000	-1.02030003	-0.18920000	1	-3.54130006	-1.66849995	-0.45050001

1	1.19939995	-1.59609997	-0.16210000	1	-3.71950006	-0.26460001	-1.50059998
1	3.33699989	-0.85879999	-1.26730001	1	1.37919998	2.49460006	-0.59880000
1	3.62179995	-2.03209996	0.01540000	1	2.86039996	1.43470001	-0.87650001
1	3.95250010	-0.32449999	0.29830000	1	-2.70460010	-0.17060000	1.39719999
1	-0.22600000	0.33210000	1.67279994	1	1.79990005	-1.07690001	1.38269997

BU2_rad 3-methyleneheptan-2-yl radical

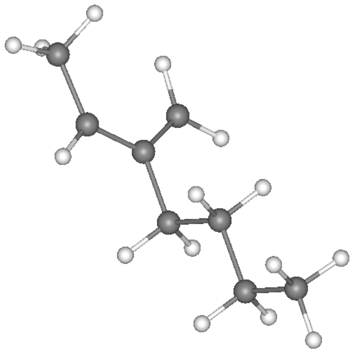

Zero-point vibrational energy	549463.5 (Joules/Mol)
	131.32494 (Kcal/Mol)
Zero-point correction=	0.209280 (Hartree/Particle)
Thermal correction to Energy=	0.219955
Thermal correction to Enthalpy=	0.220899
Thermal correction to Gibbs Free Energy=	0.171948
Sum of electronic and zero-point Energies=	-313.609461
Sum of electronic and thermal Energies=	-313.598786
Sum of electronic and thermal Enthalpies=	-313.597842
Sum of electronic and thermal Free Energies=	-313.646793

cartesian

6	-1.72800004	1.54040003	-0.45879999	1	0.97649997	-1.04489994	-0.50650001
6	-1.24500000	0.42530000	0.19380000	1	1.18799996	0.58920002	-1.09150004
6	0.14590000	0.46309999	0.78820002	1	-3.71810007	-1.96319997	-0.04740000
6	-1.97770000	-0.75739998	0.29730001	1	-4.06110001	-0.24190000	0.10630000
1	0.38190001	1.48450005	1.10409999	1	-3.33960009	-0.86170000	-1.36979997
1	0.17410000	-0.15019999	1.69529998	6	3.68980002	-0.48199999	-0.56889999
1	-1.52639997	-1.58440006	0.83780003	1	2.86179996	1.02219999	0.72979999
6	-3.33690000	-0.96840000	-0.27900001	1	2.64980006	-0.61330003	1.31190002
1	-2.70190001	1.55299997	-0.93269998	1	4.68650007	-0.45400000	-0.12240000
1	-1.14470005	2.45190001	-0.51789999	1	3.50009990	-1.51119995	-0.88590002
6	1.22000003	-0.02560000	-0.18449999	1	3.71530008	0.13730000	-1.46969998
6	2.62479997	0.00230000	0.40500000				

MB2 2,6-dimethyl-3-methyleneheptane

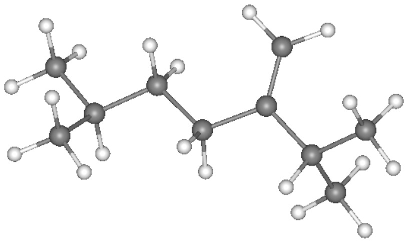

Zero-point vibrational energy	736671.7 (Joules/Mol)
	176.06877 (Kcal/Mol)
Zero-point correction=	0.280583 (Hartree/Particle)
Thermal correction to Energy=	0.293542
Thermal correction to Enthalpy=	0.294486
Thermal correction to Gibbs Free Energy=	0.241430
Sum of electronic and zero-point Energies=	-392.803590
Sum of electronic and thermal Energies=	-392.790632
Sum of electronic and thermal Enthalpies=	-392.789688
Sum of electronic and thermal Free Energies=	-392.842744

cartesian

6	2.73539996	-1.02719998	1.24450004	1	-0.19850001	-0.87489998	-1.04929996
6	2.41939998	-0.49100000	-0.15700001	1	-0.07050000	-1.11070001	0.66759998
6	1.19790006	0.40950000	-0.12340000	1	-1.24020004	1.10039997	1.03559995
6	-0.11800000	-0.32800001	-0.10120000	1	-1.48490000	1.23080003	-0.69450003
6	-1.36950004	0.50800002	0.12290000	1	-2.52399993	-1.03299999	1.05690002
6	-2.66339993	-0.30260000	0.24789999	6	-2.99970007	-1.07130003	-1.02690005
6	1.28369999	1.73819995	-0.11570000	6	-3.82119989	0.61080003	0.63950002
6	3.65960002	0.15510000	-0.76389998	1	-3.94280005	-1.61339998	-0.92110002
1	2.15720010	-1.35440004	-0.78410000	1	-2.23300004	-1.80439997	-1.28550005
1	3.55710006	-1.74740005	1.20940006	1	-3.10710001	-0.38909999	-1.87660003
1	1.87880003	-1.52909994	1.69900000	1	-4.74739981	0.04640000	0.77550000
1	3.03099990	-0.21010000	1.90750003	1	-4.00559998	1.36280000	-0.13420001
1	3.45510006	0.56680000	-1.75430000	1	-3.61579990	1.14189994	1.57260001
1	4.46229982	-0.57980001	-0.86210001	1	0.40590000	2.37129998	-0.06670000
1	4.04150009	0.96439999	-0.13609999	1	2.23639989	2.25259995	-0.16010000

OCT oct-1-ene

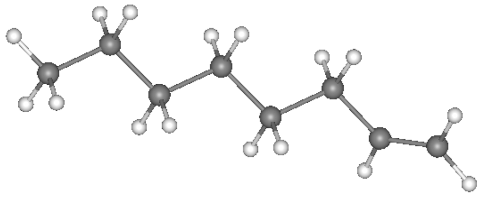

Zero-point vibrational energy	587824.9 (Joules/Mol)
	140.49352 (Kcal/Mol)
Zero-point correction=	0.223891 (Hartree/Particle)
Thermal correction to Energy=	0.234432
Thermal correction to Enthalpy=	0.235376
Thermal correction to Gibbs Free Energy=	0.187310
Sum of electronic and zero-point Energies=	-314.234535
Sum of electronic and thermal Energies=	-314.223994
Sum of electronic and thermal Enthalpies=	-314.223050
Sum of electronic and thermal Free Energies=	-314.271115

cartesian

6	3.31489992	-0.22090000	0.34940001	1	-1.77960002	-0.96469998	0.92799997
6	2.05990005	0.57760000	0.19360000	1	-1.67840004	-1.04869998	-0.81669998
6	0.80159998	-0.29229999	0.16599999	1	4.29269981	0.31750000	-1.44169998
6	-0.48480001	0.51660001	0.05420000	1	5.19689989	-0.86870003	-0.35210001
6	4.31519985	-0.26080000	-0.52240002	1	-0.54640001	1.21899998	0.89490002
1	1.97839999	1.28779995	1.02690005	1	3.38549995	-0.82130003	1.25619996
1	2.11549997	1.17900002	-0.71990001	6	-4.27869987	-0.41000000	-0.12150000
1	0.87199998	-0.99440002	-0.67229998	1	-3.09299994	1.16240001	0.74839997
1	0.76670003	-0.90890002	1.07239997	1	-2.99140000	1.07790005	-0.99540001
1	-0.44720000	1.13610005	-0.85049999	1	-5.18680000	0.19130000	-0.20580000
6	-1.74090004	-0.34540001	0.02310000	1	-4.26140022	-1.10020006	-0.96950001
6	-3.02999997	0.45989999	-0.09080000	1	-4.36619997	-1.01189995	0.78729999

OCT_rad oct-1-en-3-yl radical

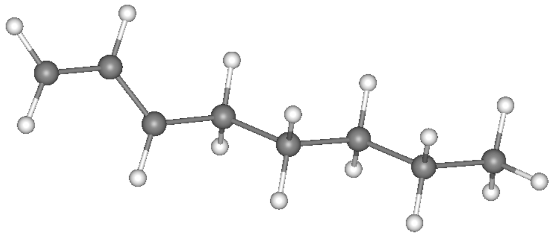

Zero-point vibrational energy	550938.8 (Joules/Mol)
	131.67753 (Kcal/Mol)
Z Zero-point correction=	0.209841 (Hartree/Particle)

Thermal correction to Energy=	0.220336
Thermal correction to Enthalpy=	0.221280
Thermal correction to Gibbs Free Energy=	0.172675
Sum of electronic and zero-point Energies=	-313.608136
Sum of electronic and thermal Energies=	-313.597641
Sum of electronic and thermal Enthalpies=	-313.596697
Sum of electronic and thermal Free Energies=	-313.645303

cartesian

6	-4.40250015	-0.42480001	-0.25020000	6	2.89159989	-0.61760002	-0.08710000
6	-3.17100000	0.18189999	-0.39410001	1	1.73269999	1.03439999	-0.83490002
6	-2.10500002	0.04650000	0.47639999	1	1.91050005	1.02030003	0.90619999
1	-5.19509983	-0.27370000	-0.97170001	1	-3.02399993	0.82239997	-1.26320004
1	-4.61520004	-1.07330000	0.59259999	1	-2.23070002	-0.59230000	1.34940004
6	-0.77730000	0.69970000	0.30289999	6	4.24790001	0.06010000	-0.22270000
6	0.37700000	-0.30080000	0.17290001	1	2.89759994	-1.27909994	0.78689998
1	-0.79619998	1.35339999	-0.57590002	1	2.71810007	-1.26740003	-0.95260000
1	-0.57220000	1.35189998	1.16369998	1	5.05579996	-0.67000002	-0.30960000
6	1.73800004	0.37059999	0.03880000	1	4.28579998	0.69959998	-1.10880005
1	0.38049999	-0.96329999	1.04670000	1	4.46589994	0.69010001	0.64420003
1	0.19430000	-0.94610000	-0.69349998				

MA maleic anhydride

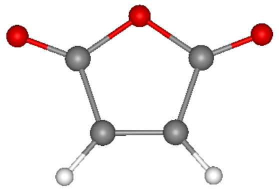

Zero-point vibrational energy	148175.2 (Joules/Mol)
	35.41473 (Kcal/Mol)
Zero-point correction=	0.056437 (Hartree/Particle)
Thermal correction to Energy=	0.061578
Thermal correction to Enthalpy=	0.062522
Thermal correction to Gibbs Free Energy=	0.027392
Sum of electronic and zero-point Energies=	-379.232910
Sum of electronic and thermal Energies=	-379.227769
Sum of electronic and thermal Enthalpies=	-379.226824
Sum of electronic and thermal Free Energies=	-379.261955

cartesian

6	-1.12310004	0.16010000	0.00000000	8	-2.22589993	0.59750003	-0.00010000

6	-0.66420001	-1.25259995	0.00010000	8	2.22589993	0.59750003	0.00000000
6	0.66420001	-1.25259995	0.00010000	1	-1.35609996	-2.08170009	0.00020000
6	1.12310004	0.16010000	0.00000000	1	1.35609996	-2.08170009	0.00020000
8	0.00000000	0.96410000	-0.00010000				

MMI N-methylmaleimide

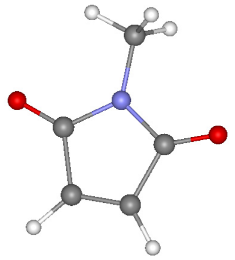

Zero-point vibrational energy	255407.9 (Joules/Mol)
	61.04395 (Kcal/Mol)
Zero-point correction=	0.097280 (Hartree/Particle)
Thermal correction to Energy=	0.104419
Thermal correction to Enthalpy=	0.105363
Thermal correction to Gibbs Free Energy=	0.065341
Sum of electronic and zero-point Energies=	-398.643424
Sum of electronic and thermal Energies=	-398.636285
Sum of electronic and thermal Enthalpies=	-398.635341
Sum of electronic and thermal Free Energies=	-398.675363

cartesian

6	1.14170003	-0.20950000	0.00010000	1	1.33050001	-2.46449995	-0.00040000
6	0.65109998	-1.62469995	-0.00020000	1	-1.37210000	-2.44079995	-0.00040000
6	-0.67799997	-1.61310005	-0.00020000	6	0.02130000	2.03069997	0.00020000
6	-1.14540005	-0.19050001	0.00000000	1	-1.01240003	2.36999989	0.00020000
7	0.00560000	0.58859998	0.00010000	1	0.53049999	2.40879989	0.88690001
8	2.27719998	0.18600000	0.00000000	1	0.53049999	2.40890002	-0.88650000
8	-2.27600002	0.21890000	0.00000000				

tBu-MA radical

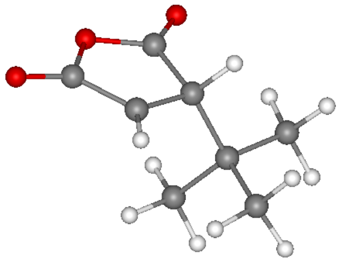

Zero-point vibrational energy	471263.0 (Joules/Mol)
	112.63456 (Kcal/Mol)
Zero-point correction=	0.179495 (Hartree/Particle)
Thermal correction to Energy=	0.190473
Thermal correction to Enthalpy=	0.191417
Thermal correction to Gibbs Free Energy=	0.142437
Sum of electronic and zero-point Energies=	-536.940787
Sum of electronic and thermal Energies=	-536.929808
Sum of electronic and thermal Enthalpies=	-536.928864
Sum of electronic and thermal Free Energies=	-536.977845

cartesian

6	-2.07850003	-0.52509999	-0.02790000	6	1.23889995	-0.36090001	1.57799995
6	-0.87220001	-1.07729995	-0.58690000	1	-0.79839998	-2.11150002	-0.88590002
6	0.16110000	-0.02900000	-0.69669998	1	2.16529989	-0.58770001	2.11019993
6	-0.59859997	1.20039999	-0.20150000	1	0.51630002	-1.14160001	1.83130002
8	-1.85819995	0.83639997	0.19650000	1	0.86519998	0.58899999	1.96780002
8	-3.12630010	-1.03840005	0.23320000	1	3.43050003	0.64740002	0.29449999
8	-0.22750001	2.32780004	-0.13600001	1	2.11129999	1.80139995	0.05640000
6	1.50010002	-0.29420000	0.07270000	1	2.72300005	0.86400002	-1.30729997
1	0.43220001	0.14749999	-1.74730003	1	3.05789995	-1.79219997	0.04170000
6	2.49329996	0.82529998	-0.23880000	1	2.21339989	-1.63419998	-1.49660003
6	2.07800007	-1.62500000	-0.41139999	1	1.44679999	-2.47399998	-0.13930000

tBu-MMI radical

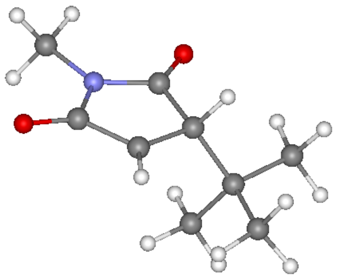

Zero-point vibrational energy	579168.6 (Joules/Mol)
	138.42462 (Kcal/Mol)
Zero-point correction=	0.220594 (Hartree/Particle)
Thermal correction to Energy=	0.233496
Thermal correction to Enthalpy=	0.234440
Thermal correction to Gibbs Free Energy=	0.180979
Sum of electronic and zero-point Energies=	-556.354618
Sum of electronic and thermal Energies=	-556.341716
Sum of electronic and thermal Enthalpies=	-556.340771

Sum of electronic and thermal Free Energies= -556.394233

cartesian

6	-1.66180003	-1.05470002	-0.11660000	1	2.44980001	-0.07880000	2.18919992
6	-0.31930000	-1.38960004	-0.53990000	1	0.92909998	-0.93180001	1.92890000
6	0.49840000	-0.16270000	-0.70520002	1	0.97700000	0.83880001	1.89189994
6	-0.50190002	0.94559997	-0.35730001	1	3.54660010	1.19319999	0.28929999
7	-1.69760001	0.34450001	-0.01830000	1	2.05069995	2.05290008	-0.11280000
8	-2.60619998	-1.77740002	0.11420000	1	2.88759995	1.10899997	-1.34590006
8	-0.31470001	2.13739991	-0.37000000	1	3.63360000	-1.28069997	0.28040001
6	1.82609999	-0.10460000	0.11640000	1	2.83389997	-1.44369996	-1.28199995
1	0.77560002	-0.03060000	-1.76150000	1	2.18230009	-2.26609993	0.14360000
6	2.61859989	1.14010000	-0.28610000	6	-2.88240004	1.07110000	0.36520001
6	2.65779996	-1.34730005	-0.20680000	1	-2.70029998	2.12750006	0.18240000
6	1.52059996	-0.06740000	1.61450005	1	-3.73370004	0.72570002	-0.22050001
1	-0.01420000	-2.40630007	-0.73519999	1	-3.10369992	0.91530001	1.42240000

S3. MA – olefin complexes

MA – ET

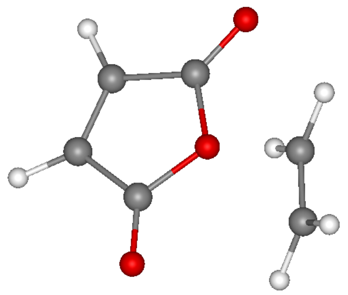

Zero-point vibrational energy	284729.9 (Joules/Mol)
	68.05208 (Kcal/Mol)
Zero-point correction=	0.108448 (Hartree/Particle)
Thermal correction to Energy=	0.118786
Thermal correction to Enthalpy=	0.119730
Thermal correction to Gibbs Free Energy=	0.068028
Sum of electronic and zero-point Energies=	-457.762058
Sum of electronic and thermal Energies=	-457.751720
Sum of electronic and thermal Enthalpies=	-457.750775
Sum of electronic and thermal Free Energies=	-457.802477

cartesian

6	2.43939996	-1.37140000	-0.14600000	8	0.38040000	2.04570007	-0.80980003
6	2.99189997	-0.30329999	0.41260001	1	3.45040011	-0.34590000	1.39540005
6	-1.43250000	-0.71439999	-0.10320000	1	3.01609993	0.65590000	-0.09350000
6	-1.39870000	-0.06320000	1.23010004	1	2.42109990	-2.33369994	0.35530001
6	-0.70319998	1.06280005	1.11930001	1	1.98899996	-1.33060002	-1.13189995
6	-0.26019999	1.19120002	-0.29150000	1	-1.87740004	-0.49860001	2.09450006
8	-0.73540002	0.09340000	-0.98070002	1	-0.45800000	1.80110002	1.86800003
8	-1.94009995	-1.73399997	-0.43640000				

MA – BU

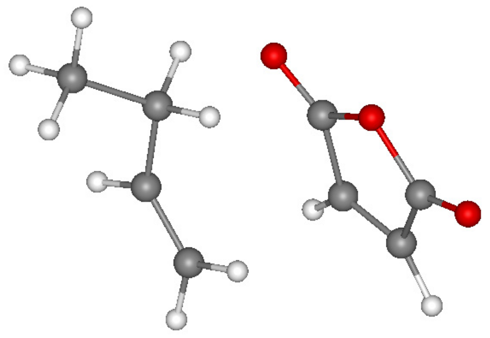

Zero-point vibrational energy	436628.1 (Joules/Mol)
	104.35663 (Kcal/Mol)
Zero-point correction=	0.166303 (Hartree/Particle)
Thermal correction to Energy=	0.178918
Thermal correction to Enthalpy=	0.179862
Thermal correction to Gibbs Free Energy=	0.123126
Sum of electronic and zero-point Energies=	-536.335143
Sum of electronic and thermal Energies=	-536.322528
Sum of electronic and thermal Enthalpies=	-536.321583
Sum of electronic and thermal Free Energies=	-536.378320

cartesian

6	-2.47270012	-0.12639999	-0.47819999	8	1.12129998	0.16260000	-1.01909995
6	-1.96430004	-0.22149999	0.92500001	8	0.32830000	2.17269993	-0.37520000
6	-1.24119997	-1.22119999	1.41919994	8	2.25189996	-1.78859997	-1.01660001
6	-3.99720001	-0.05060000	-0.54089999	1	-0.92030001	-1.23609996	2.45539999
1	-2.11269999	-0.98049998	-1.05900002	1	-0.95660001	-2.07299995	0.80760002
1	-4.45499992	-0.94669998	-0.11610000	1	-2.04949999	0.76840001	-0.94819999
1	-4.37599993	0.81040001	0.01630000	1	-4.34250021	0.04810000	-1.57229996
6	1.02390003	1.23520005	-0.15740000	1	-2.23429990	0.60479999	1.58260000
6	1.92060006	0.97049999	0.99540001	1	2.02550006	1.66729999	1.81350005
6	2.50009990	-0.20860000	0.80110002	1	3.20840001	-0.74190003	1.41729999
6	1.99769998	-0.75959998	-0.48249999				

MA – EB

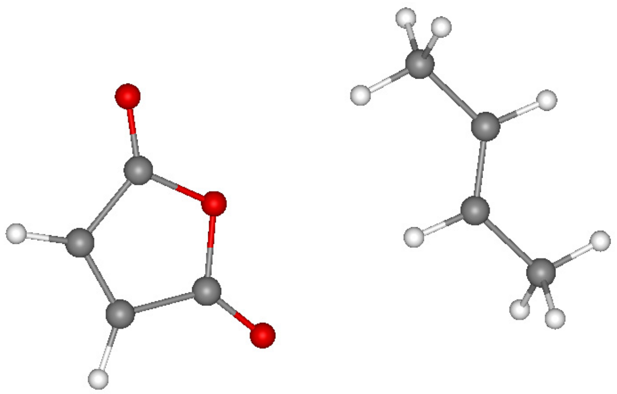

Zero-point vibrational energy	434569.2 (Joules/Mol)
	103.86452 (Kcal/Mol)
Zero-point correction=	0.165519 (Hartree/Particle)
Thermal correction to Energy=	0.177649
Thermal correction to Enthalpy=	0.178594
Thermal correction to Gibbs Free Energy=	0.121774
Sum of electronic and zero-point Energies=	-536.340665

Sum of electronic and thermal Energies=	-536.328535
Sum of electronic and thermal Enthalpies=	-536.327591
Sum of electronic and thermal Free Energies=	-536.384410

cartesian

6	3.38759995	0.95300001	-0.00180000	1	-3.35739994	-2.32559991	-0.00750000
6	3.10829997	-0.34689999	-0.00150000	1	4.02029991	-2.08979988	0.86460000
6	-2.42880011	0.88569999	-0.00320000	1	4.00530005	-2.08690000	-0.88859999
6	-3.49699998	-0.14600000	-0.01120000	1	5.14340019	-1.04879999	-0.02000000
6	-2.91529989	-1.34039998	-0.00480000	1	2.06529999	-0.66090000	0.00680000
6	-1.44490004	-1.13440001	0.00760000	6	2.37700009	2.05329990	0.00860000
8	-1.21379995	0.22589999	0.00810000	1	1.35679996	1.66670001	0.01560000
8	-2.51519990	2.06839991	-0.00530000	1	2.49930000	2.69720006	0.88590002
8	-0.56800002	-1.93429995	0.01590000	1	2.48559999	2.70129991	-0.86750001
6	4.12449980	-1.44200003	-0.01190000	1	4.43389988	1.26080000	-0.01010000
1	-4.54540014	0.11260000	-0.02060000				

MA – ZB

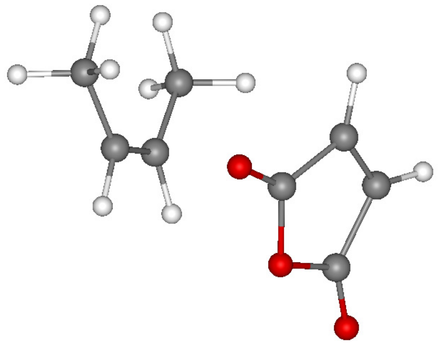

Zero-point vibrational energy	435599.8 (Joules/Mol)
	104.11086 (Kcal/Mol)
Zero-point correction=	0.165911 (Hartree/Particle)
Thermal correction to Energy=	0.178803
Thermal correction to Enthalpy=	0.179747
Thermal correction to Gibbs Free Energy=	0.122036
Sum of electronic and zero-point Energies=	-536.338651
Sum of electronic and thermal Energies=	-536.325759
Sum of electronic and thermal Enthalpies=	-536.324814
Sum of electronic and thermal Free Energies=	-536.382526

cartesian

6	-2.82010007	1.25209999	-0.15570000	6	-2.47729993	-1.78460002	0.68870002
6	-2.00880003	0.19599999	-0.83310002	1	-1.23230004	-1.70319998	-1.11189997

6	-1.86029994	-1.08060002	-0.47679999	1	-3.30150008	0.90630001	0.75940001
1	-2.19600010	2.11490011	0.09410000	1	-3.60540009	1.62210000	-0.82260001
6	1.07239997	1.26699996	0.18700001	1	0.84020001	0.87739998	2.39369988
6	1.16229999	0.48040000	1.44260001	1	1.89639997	-1.54680002	1.78859997
6	1.67990005	-0.70620000	1.14649999	1	-1.48500001	0.52429998	-1.72839999
6	1.93519998	-0.74229997	-0.31520000	1	-1.71159995	-2.24200010	1.32379997
8	1.56260002	0.48089999	-0.83560002	1	-3.08030009	-1.12820005	1.31610000
8	0.67600000	2.37280011	0.01320000	1	-3.12229991	-2.60190010	0.35060000
8	2.37380004	-1.61790001	-0.98600000				

MA – IB

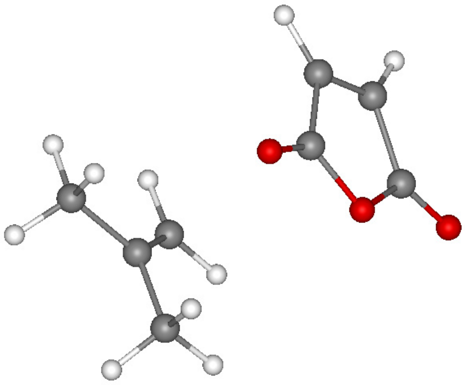

Zero-point vibrational energy	435226.1 (Joules/Mol)
	104.02153 (Kcal/Mol)
Zero-point correction=	0.165769 (Hartree/Particle)
Thermal correction to Energy=	0.178361
Thermal correction to Enthalpy=	0.179305
Thermal correction to Gibbs Free Energy=	0.123518
Sum of electronic and zero-point Energies=	-536.342039
Sum of electronic and thermal Energies=	-536.329447
Sum of electronic and thermal Enthalpies=	-536.328503
Sum of electronic and thermal Free Energies=	-536.384290

cartesian

6	-3.01819992	0.65380001	0.62620002	1	-1.43499994	-0.86360002	2.23819995
6	-2.20409989	-0.56010002	0.29170001	1	-0.88550001	-2.07430005	0.95370001
6	-1.46550000	-1.19159997	1.20379996	1	-2.94050002	0.92180002	1.68099999
1	-2.69549990	1.51080000	0.02730000	6	-2.26929998	-1.01160002	-1.13610005
6	0.71560001	1.10440004	-0.48480001	1	-4.07579994	0.49050000	0.39510000
6	1.31799996	1.32959998	0.85290003	1	-1.65590000	-1.89499998	-1.31459999
6	2.15400004	0.32760000	1.09930003	1	-1.92799997	-0.21840000	-1.80770004
6	2.11700010	-0.59549999	-0.06220000	1	-3.29990005	-1.24590003	-1.42190003

8	1.23989999	-0.06640000	-0.98930001	1	1.06780005	2.18750000	1.45879996
8	-0.06480000	1.76940000	-1.08529997	1	2.77290010	0.13900000	1.96379995
8	2.69880009	-1.61440003	-0.24030000				

MA – TME

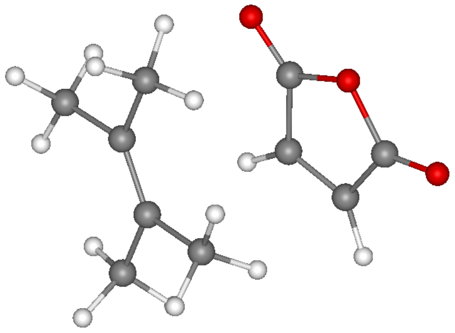

Zero-point vibrational energy	583453.2 (Joules/Mol)
	139.44867 (Kcal/Mol)
Zero-point correction=	0.222226 (Hartree/Particle)
Thermal correction to Energy=	0.237924
Thermal correction to Enthalpy=	0.238868
Thermal correction to Gibbs Free Energy=	0.176441
Sum of electronic and zero-point Energies=	-614.911337
Sum of electronic and thermal Energies=	-614.895638
Sum of electronic and thermal Enthalpies=	-614.894694
Sum of electronic and thermal Free Energies=	-614.957121

cartesian

6	-2.72900009	1.21459997	0.48590001	1	-0.96960002	0.32229999	-2.35339999
6	-1.98490000	0.26539999	-0.41190001	1	-1.17929995	1.87919998	-1.56550002
6	-1.68099999	-1.00390005	-0.08460000	1	-2.55669999	1.08980000	-2.31060004
1	-2.09509993	2.06909990	0.74409997	1	0.50870001	0.33460000	2.38120008
6	1.14069998	1.27900004	0.43779999	1	2.14450002	-1.68560004	1.60619998
6	1.09920001	0.22860000	1.48399997	6	-2.03480005	-1.64450002	1.23060000
6	1.90320003	-0.75999999	1.10520005	1	-2.83809996	-2.37849998	1.10029995
6	2.46779990	-0.40910000	-0.22020000	1	-1.17920005	-2.20239997	1.62629998
8	1.96710002	0.83109999	-0.57230002	1	-2.35339999	-0.94389999	1.99969995
8	0.61379999	2.34389997	0.40770000	6	-0.98879999	-1.96969998	-1.00460005
8	3.21180010	-1.01859999	-0.91570002	1	-1.57640004	-2.88930011	-1.09770000
1	-3.09480000	0.77069998	1.40929997	1	-0.81809998	-1.59119999	-2.00929999
6	-1.64569998	0.90369999	-1.73080003	1	-0.01840000	-2.27629995	-0.59410000
1	-3.59640002	1.62510002	-0.04170000				

MA – OCT

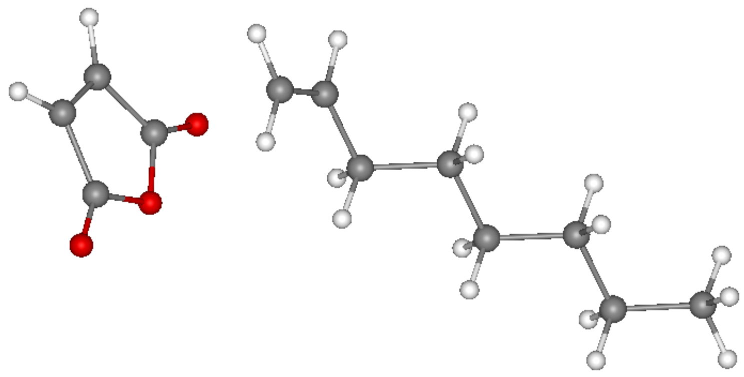

Zero-point vibrational energy	737679.8 (Joules/Mol)
	176.30971 (Kcal/Mol)
Zero-point correction=	0.280967 (Hartree/Particle)
Thermal correction to Energy=	0.298976
Thermal correction to Enthalpy=	0.299920
Thermal correction to Gibbs Free Energy=	0.228861
Sum of electronic and zero-point Energies=	-693.469820
Sum of electronic and thermal Energies=	-693.451811
Sum of electronic and thermal Enthalpies=	-693.450867
Sum of electronic and thermal Free Energies=	-693.521926

cartesian

6	0.52160001	0.57279998	-0.02850000	1	-0.36430001	1.97350001	1.41700006
6	-0.42559999	1.65600002	0.37610000	1	-4.39020014	0.41790000	2.11350012
6	-1.30350006	2.25370002	-0.42309999	1	-5.50000000	0.66949999	-0.34930000
6	1.98599994	0.95469999	0.19570000	6	2.95560002	-0.16000000	-0.17730001
1	0.35920000	0.31270000	-1.07939994	1	2.70989990	-1.06130004	0.39760000
1	2.21659994	1.85560000	-0.38429999	1	2.81100011	-0.42910001	-1.23090005
1	2.13290000	1.22730005	1.24800003	6	4.41650009	0.20530000	0.05480000
6	-2.83319998	-0.79629999	1.02929997	1	4.66090012	1.10990000	-0.51609999
6	-4.06309986	0.02380000	1.16299999	1	4.56199980	0.47000000	1.10969996
6	-4.60659981	0.14640000	-0.04250000	6	5.38920021	-0.90579998	-0.32269999
6	-3.74729991	-0.58310002	-1.00839996	1	5.14300013	-1.81029999	0.24540000
8	-2.70140004	-1.14030004	-0.29990000	1	5.24720001	-1.16770005	-1.37759995
8	-2.06019998	-1.14219999	1.86179996	6	6.84560013	-0.53450000	-0.08180000
8	-3.86750007	-0.71240002	-2.18210006	1	7.52029991	-1.34669995	-0.36250001
1	-1.95099998	3.04819989	-0.06740000	1	7.13259983	0.34670001	-0.66229999
1	-1.39610004	1.98029995	-1.47049999	1	7.02820015	-0.30489999	0.97170001
1	0.29780000	-0.33039999	0.55220002				

MA – BU2

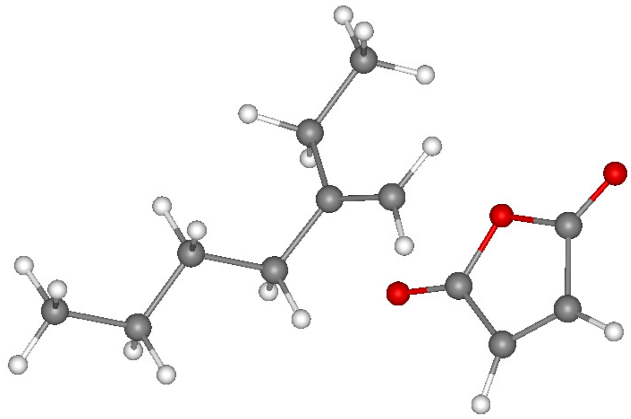

Zero-point vibrational energy	738237.7 (Joules/Mol)
	176.44305 (Kcal/Mol)
Zero-point correction=	0.281180 (Hartree/Particle)
Thermal correction to Energy=	0.298943
Thermal correction to Enthalpy=	0.299887
Thermal correction to Gibbs Free Energy=	0.230696
Sum of electronic and zero-point Energies=	-693.473456
Sum of electronic and thermal Energies=	-693.455693
Sum of electronic and thermal Enthalpies=	-693.454749
Sum of electronic and thermal Free Energies=	-693.523941

cartesian

6	-1.76580000	-0.33090001	-0.19620000	1	-0.39199999	1.26010001	1.60020006
6	-0.77240002	0.78030002	-0.42370000	1	-1.65919995	2.20650005	0.87800002
6	-0.04250000	0.83080000	-1.53859997	1	1.67040002	-2.96029997	-0.29879999
1	-1.56789994	-0.79380000	0.77829999	1	3.60689998	-1.81659997	-1.81560004
6	1.84609997	-1.07790005	0.92580003	1	0.30019999	3.62339997	1.31270003
6	2.16820002	-2.00909996	-0.18400000	1	1.33749998	2.58500004	0.34390000
6	3.11750007	-1.44990003	-0.92570001	1	0.05450000	3.52449989	-0.42890000
6	3.44109988	-0.12790000	-0.33430001	6	-4.20419979	-1.04410005	-0.05820000
8	2.65240002	0.03200000	0.78850001	1	-3.42179990	0.86860001	0.52079999
8	1.06439996	-1.19770002	1.81219995	1	-3.42219996	0.60200000	-1.21019995
8	4.21500015	0.69630003	-0.69499999	6	-5.66230011	-0.60970002	-0.10140000
1	-0.15090001	0.07890000	-2.31439996	1	-4.02419996	-1.80069995	-0.83109999
1	0.66909999	1.62530005	-1.73290002	1	-3.99650002	-1.53789997	0.89819998
1	-1.60940003	-1.11249995	-0.94739997	1	-6.33839989	-1.45669997	0.03650000
6	-0.66090000	1.79740000	0.68210000	1	-5.88339996	0.11840000	0.68379998
6	-3.23020005	0.11270000	-0.24840000	1	-5.91020012	-0.14320000	-1.05869997
6	0.31259999	2.94239998	0.45919999				

MA – MB2

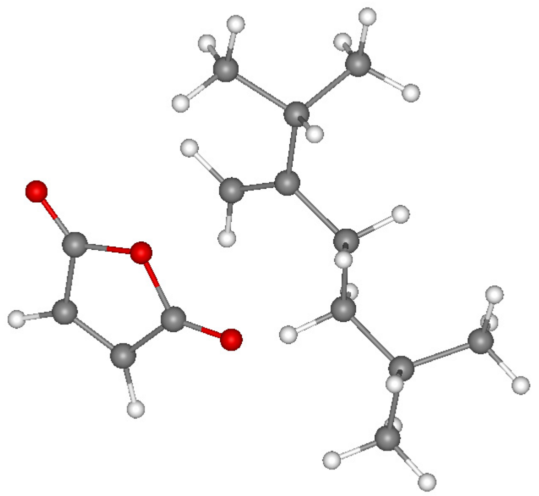

Zero-point vibrational energy	885716.4 (Joules/Mol)
	211.69129 (Kcal/Mol)
Zero-point correction=	0.337352 (Hartree/Particle)
Thermal correction to Energy=	0.357942
Thermal correction to Enthalpy=	0.358886
Thermal correction to Gibbs Free Energy=	0.282403
Sum of electronic and zero-point Energies=	-772.039359
Sum of electronic and thermal Energies=	-772.018769
Sum of electronic and thermal Enthalpies=	-772.017825
Sum of electronic and thermal Free Energies=	-772.094308

cartesian

6	1.52499998	0.75300002	-0.20530000	1	-3.28489995	-2.44359994	1.77119994
6	0.33070001	1.42340004	0.42670000	1	-2.00929999	3.74780011	-0.71829998
6	-0.19920000	1.02320004	1.58280003	1	-2.32960010	2.10339999	-0.16900000
1	2.27069998	1.52380002	-0.42850000	1	-1.77579999	3.33610010	0.97329998
6	-1.43350005	-1.64069998	-0.89179999	6	3.32040000	-1.07900000	-0.16470000
6	-1.68060005	-2.50370002	0.29040000	1	2.55229998	0.00110000	1.53680003
6	-2.78850007	-2.07629991	0.88550001	1	1.41999996	-1.12880003	0.82040000
6	-3.30049992	-0.91420001	0.11760000	6	3.78830004	-2.29999995	0.62199998
8	-2.44980001	-0.71109998	-0.95130002	1	2.92389989	-1.42990005	-1.12639999
8	-0.55400002	-1.68340003	-1.68900001	6	4.49749994	-0.14929999	-0.44679999
8	-4.25110006	-0.23250000	0.31770000	1	4.57649994	-2.83990002	0.09110000
1	0.22319999	0.20690000	2.15689993	1	4.19010019	-2.00819993	1.59759998
1	-1.06640005	1.51020002	2.01340008	1	2.96900010	-3.00209999	0.79939997
1	1.21039999	0.37180001	-1.18579996	1	0.32670000	4.66069984	-0.80140001
6	-0.21520001	2.60150003	-0.36059999	1	0.65880001	4.16919994	0.86339998
6	2.17869997	-0.37680000	0.57669997	1	1.71850002	3.64269996	-0.44580001

6	-1.66340005	2.95840001	-0.04670000	1	5.31029987	-0.68599999	-0.94279999
1	-0.16830000	2.31780005	-1.42060006	1	4.22249985	0.68690002	-1.09259999
6	0.67809999	3.83520007	-0.17730001	1	4.89860010	0.26629999	0.48350000
1	-1.02440000	-3.31960011	0.55449998				

S4. Alder-ene reaction

ene_TS_a

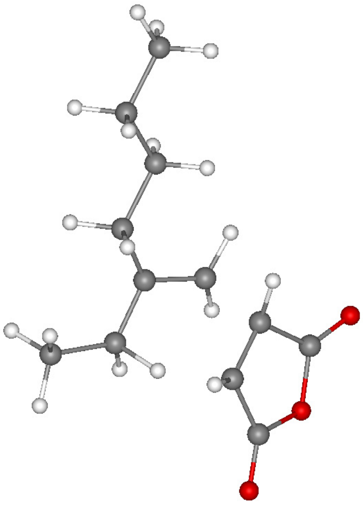

Zero-point vibrational energy	734185.8 (Joules/Mol)
	175.47461 (Kcal/Mol)
Zero-point correction=	0.279637 (Hartree/Particle)
Thermal correction to Energy=	0.295307
Thermal correction to Enthalpy=	0.296251
Thermal correction to Gibbs Free Energy=	0.235622
Sum of electronic and zero-point Energies=	-693.436575
Sum of electronic and thermal Energies=	-693.420905
Sum of electronic and thermal Enthalpies=	-693.419961
Sum of electronic and thermal Free Energies=	-693.480590

cartesian

6	2.04119992	-1.69480002	-0.16910000	1	-0.87400001	3.71359992	-1.04310000
6	0.99890000	-0.85339999	0.50580001	1	-0.74500000	3.28040004	0.66159999
6	1.70150006	0.24250001	1.04540002	1	-2.49130011	1.74430001	-0.39629999
6	3.08089995	0.17060000	0.58130002	6	-2.85719991	-0.36140001	-0.13650000
8	3.19899988	-0.97170001	-0.23810001	1	-1.76610005	1.08220005	1.03349996
8	1.93719995	-2.79390001	-0.61440003	1	-0.64069998	-1.32550001	-1.17240000
8	4.02299976	0.87080002	0.77810001	1	0.76590002	-0.38280001	-1.80320001
1	0.23210000	-1.38450003	1.05530000	6	-4.21099997	-0.14839999	0.53160000
1	1.46580005	0.76480001	1.96200001	1	-3.00819993	-0.61299998	-1.19239998
6	-0.04040000	-0.42870000	-1.07669997	1	-2.37019992	-1.23220003	0.31639999
6	-0.63559997	0.78869998	-0.73689997	6	-5.11700010	-1.36730003	0.43740001
6	0.26230001	1.90530002	-0.69110000	1	-4.05950022	0.11610000	1.58449996
6	-0.20870000	3.28550005	-0.28889999	1	-4.70790005	0.71480000	0.07410000
6	-1.95430005	0.86080003	-0.02950000	1	-6.07910013	-1.18820000	0.92170000
1	0.96100003	1.92390001	-1.53040004	1	-5.31750011	-1.63479996	-0.60339999

1	1.02040005	1.46089995	0.15380000	1	-4.66410017	-2.23850012	0.91820002
1	0.64069998	3.96099997	-0.18189999				

ene_TS_b

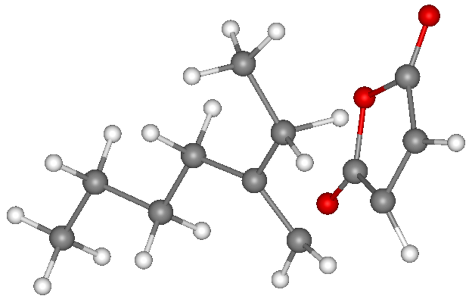

Zero-point vibrational energy	735574.4 (Joules/Mol)
	175.80650 (Kcal/Mol)
Zero-point correction=	0.280165 (Hartree/Particle)
Thermal correction to Energy=	0.295728
Thermal correction to Enthalpy=	0.296672
Thermal correction to Gibbs Free Energy=	0.236727
Sum of electronic and zero-point Energies=	-693.438759
Sum of electronic and thermal Energies=	-693.423197
Sum of electronic and thermal Enthalpies=	-693.422252
Sum of electronic and thermal Free Energies=	-693.482197

cartesian

6	-0.75889999	-1.68640006	0.19000000	1	-0.13030000	3.81940007	-0.53880000
6	-1.41970003	-0.87470001	1.26989996	1	-0.63360000	2.52920008	-1.63380003
6	-2.54239988	-0.28580001	0.65249997	1	1.58980000	1.97319996	-0.55479997
6	-2.57279992	-0.68379998	-0.73799998	6	2.44359994	0.15809999	0.23350000
8	-1.39209998	-1.44200003	-0.98680001	1	0.81519997	0.53219998	-1.12800002
8	0.18179999	-2.41560006	0.28130001	1	0.71359998	-0.35020000	2.15120006
8	-3.35080004	-0.49540001	-1.61950004	1	-0.63880002	0.73290002	2.68689990
1	-1.48920000	-1.36029994	2.23699999	6	3.53940010	0.10070000	-0.82459998
1	-3.43079996	0.08440000	1.14240003	1	2.85100007	0.58410001	1.15830004
6	-0.07710000	0.32760000	1.84990001	1	2.12599993	-0.86100000	0.46860000
6	0.15140000	1.20029998	0.77530003	6	4.73969984	-0.72530001	-0.38550001
6	-0.94480002	2.08299994	0.47119999	1	3.12529993	-0.31869999	-1.74820006
6	-0.87830001	3.03889990	-0.70090002	1	3.86420012	1.11810005	-1.07290006
6	1.24699998	0.98509997	-0.22280000	1	5.50860023	-0.75629997	-1.16040003
1	-1.36189997	2.54699993	1.36909997	1	5.19880009	-0.31270000	0.51719999
1	-1.79190004	1.26119995	0.29310000	1	4.45109987	-1.75629997	-0.16599999
1	-1.84029996	3.53220010	-0.84350002				

ene_TS_c

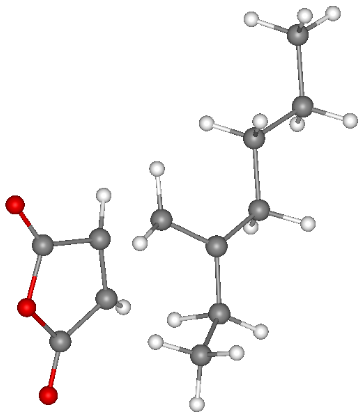

Zero-point vibrational energy	733505.2 (Joules/Mol)
	175.31195 (Kcal/Mol)
Zero-point correction=	0.279377 (Hartree/Particle)
Thermal correction to Energy=	0.295167
Thermal correction to Enthalpy=	0.296111
Thermal correction to Gibbs Free Energy=	0.234890
Sum of electronic and zero-point Energies=	-693.434721
Sum of electronic and thermal Energies=	-693.418932
Sum of electronic and thermal Enthalpies=	-693.417988
Sum of electronic and thermal Free Energies=	-693.479209

cartesian

6	1.88720000	-1.63909996	-0.36469999	6	-2.97370005	-0.11300000	-0.12850000
6	0.80180001	-0.94840002	0.40860000	1	-1.80470002	1.05239999	1.25549996
6	1.47220004	-0.01830000	1.23389995	1	-0.83029997	-0.99750000	-1.33930004
6	2.88610005	-0.03260000	0.87970001	1	0.65130001	-0.08750000	-1.79840004
8	3.05579996	-0.95630002	-0.17399999	6	-4.31619978	0.07380000	0.56989998
8	1.80739999	-2.60080004	-1.06169999	1	-3.13400006	-0.18290000	-1.21039999
8	3.82049990	0.56300002	1.31400001	1	-2.54460001	-1.07369995	0.17820001
1	0.00520000	-1.57900000	0.78299999	6	-5.29430008	-1.05789995	0.29080001
1	1.17379999	0.27129999	2.23189998	1	-4.15450001	0.16120000	1.65059996
6	-0.15940000	-0.18550000	-1.08529997	1	-4.75689983	1.02649999	0.25500000
6	-0.67519999	0.99299997	-0.53729999	1	-6.24569988	-0.89870000	0.80239999
6	0.26260000	2.02399993	-0.22280000	1	-5.50659990	-1.14670002	-0.77789998
1	-0.17790000	2.86849999	0.31079999	1	-4.89750004	-2.01959991	0.62690002
6	-1.99989998	1.01900005	0.17170000	1	2.07520008	3.09590006	-0.68070000
6	1.35029995	2.46070004	-1.19099998	1	1.90409994	1.62150002	-1.61160004
1	0.88090003	1.37500000	0.61390001	1	0.92500001	3.03340006	-2.01789999
1	-2.48070002	1.97969997	-0.04830000				

ene_TS_d

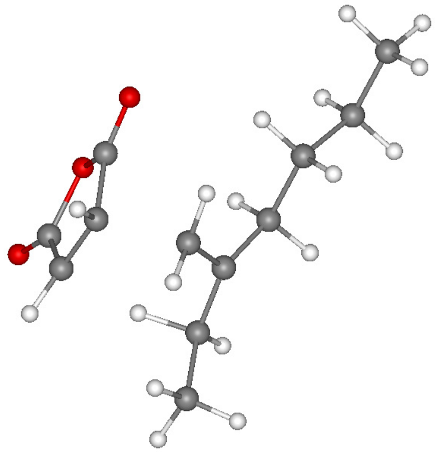

Zero-point vibrational energy	735545.6 (Joules/Mol)
	175.79961 (Kcal/Mol)
Zero-point correction=	0.280155 (Hartree/Particle)
Thermal correction to Energy=	0.295864
Thermal correction to Enthalpy=	0.296808
Thermal correction to Gibbs Free Energy=	0.236339
Sum of electronic and zero-point Energies=	-693.436470
Sum of electronic and thermal Energies=	-693.420760
Sum of electronic and thermal Enthalpies=	-693.419816
Sum of electronic and thermal Free Energies=	-693.480285

cartesian

6	-0.61559999	-1.57990003	0.67680001	6	2.47909999	0.36690000	0.07040000
6	-1.38329995	-0.43259999	1.28240001	1	0.88290000	0.02530000	-1.33710003
6	-2.47749996	-0.22690000	0.41020000	1	0.66530001	0.48649999	1.97290003
6	-2.40019989	-1.18390000	-0.67089999	1	-0.74180001	1.61570001	2.04130006
8	-1.17910004	-1.90450001	-0.51340002	6	3.62339997	-0.03100000	-0.85470003
8	0.34990001	-2.13120008	1.11160004	1	2.81599998	1.16209996	0.74650002
8	-3.12470007	-1.44309998	-1.57930005	1	2.21690011	-0.48960000	0.69700003
1	-1.52890003	-0.50260001	2.35540009	6	4.85260010	-0.51279998	-0.09790000
1	-3.41479993	0.25080001	0.65520000	1	3.27970004	-0.81959999	-1.53320003
6	-0.14250000	0.95639998	1.42250001	1	3.89310002	0.81980002	-1.49160004
6	0.10590000	1.37720001	0.10440000	1	5.65609980	-0.79699999	-0.78079998
6	-0.99339998	1.98500001	-0.59170002	1	5.24340010	0.26460001	0.56459999
1	-0.78369999	2.17090011	-1.64649999	1	4.62039995	-1.38460004	0.51889998
6	1.25419998	0.84390002	-0.70230001	1	-2.68249989	3.32550001	-0.56959999
6	-1.79550004	3.12010002	0.03160000	1	-2.13709998	2.88750005	1.04059994
1	-1.74360001	1.05760002	-0.53680003	1	-1.20290005	4.03599977	0.07770000
1	1.54960001	1.62720001	-1.41030002				

S4. MA – MA reaction profile

TS__tBuMA-rad_MA__re

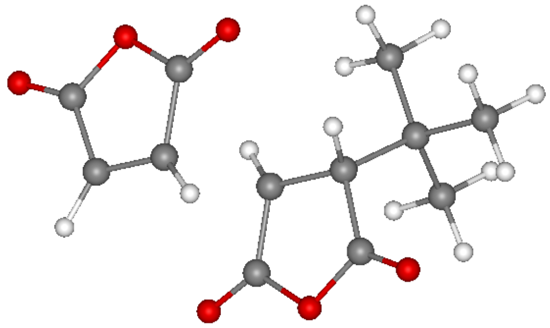

Zero-point vibrational energy	623301.4 (Joules/Mol)
	148.97260 (Kcal/Mol)
Zero-point correction=	0.237403 (Hartree/Particle)
Thermal correction to Energy=	0.254314
Thermal correction to Enthalpy=	0.255258
Thermal correction to Gibbs Free Energy=	0.191130
Sum of electronic and zero-point Energies=	-916.165292
Sum of electronic and thermal Energies=	-916.148380
Sum of electronic and thermal Enthalpies=	-916.147436
Sum of electronic and thermal Free Energies=	-916.211565

cartesian

6	-4.08935833	0.16157414	-0.12999353	1	0.30434191	-0.49722588	1.30930650
6	-3.34995794	1.26457417	0.49740648	6	2.69754171	-1.30422580	0.53940648
6	-2.29125810	0.73177415	1.16800642	6	0.83094192	-1.87152588	-1.00389349
6	-2.44245815	-0.74972582	1.11350656	6	2.26764202	0.12937415	-1.47159350
8	-3.47925806	-1.03362584	0.26650646	1	-1.03485811	0.24177414	-1.06329358
8	-5.03855801	0.17067415	-0.84269357	1	-1.68975806	1.20817423	1.92900646
8	-1.80355811	-1.59192586	1.66220641	1	1.51624191	-2.55602598	-1.50909352
1	-3.62625790	2.29947424	0.37040645	1	0.28624189	-2.44412565	-0.24969354
6	-0.34705812	2.18607426	-0.29909351	1	0.11394191	-1.53022587	-1.75419354
6	-0.50655812	0.73357415	-0.25949353	1	2.92724180	-0.48762584	-2.08559346
6	0.63604188	0.14597414	0.48580647	1	1.52484190	0.57317412	-2.14079356
6	1.28814185	1.38837409	1.07580650	1	2.87614202	0.93707412	-1.05629349
8	0.69544184	2.52047396	0.54770648	1	3.38424206	-1.91992581	-0.04659353
8	-0.97315812	3.01207399	-0.88959348	1	3.27794170	-0.52922589	1.03840649
8	2.16834211	1.47367418	1.86640644	1	2.25544167	-1.94112575	1.30980647
6	1.62134182	-0.72132587	-0.37779355				

TS__tBuMA-rad_MA__si

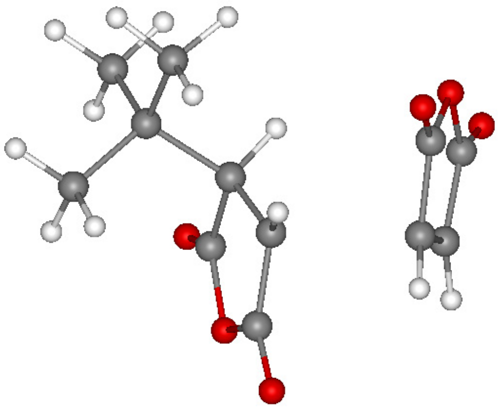

Zero-point vibrational energy	622897.1 (Joules/Mol)
	148.87598 (Kcal/Mol)
Zero-point correction=	0.237249 (Hartree/Particle)
Thermal correction to Energy=	0.254244
Thermal correction to Enthalpy=	0.255188
Thermal correction to Gibbs Free Energy=	0.190473
Sum of electronic and zero-point Energies=	-916.164796
Sum of electronic and thermal Energies=	-916.147801
Sum of electronic and thermal Enthalpies=	-916.146857
Sum of electronic and thermal Free Energies=	-916.211572

cartesian

6	3.25968385	0.05001935	-1.20550966	1	0.47408381	-0.51628065	-0.82980967
6	2.91108370	1.31681931	-0.55090964	6	-1.92461634	-1.50048065	-1.33670974
6	2.51538372	1.04351938	0.72379035	6	-1.07241607	-1.87288058	0.97169030
6	2.74918365	-0.41468063	0.94669032	6	-2.66771626	0.00691935	0.52119035
8	3.12048364	-0.96578062	-0.25430965	1	0.35768381	0.41951934	1.83799028
8	3.61578369	-0.17728063	-2.31510973	1	2.43368387	1.73751938	1.54809034
8	2.63088369	-1.05298066	1.94209027	1	-1.88551617	-2.58678079	1.12179029
1	2.97478366	2.26881933	-1.05600965	1	-0.20221621	-2.43398070	0.62229031
6	-0.02351618	2.26631927	0.69259030	1	-0.83001620	-1.45358062	1.95029020
6	0.26518381	0.84171939	0.84779030	1	-3.51301646	-0.65008068	0.73639029
6	-0.26901621	0.11921935	-0.33580965	1	-2.40611625	0.51251936	1.45509028
6	-0.60051614	1.27211928	-1.27400970	1	-3.01951623	0.76161933	-0.18710965
8	-0.48471624	2.46981931	-0.59420967	1	-2.76981616	-2.16398072	-1.13860965
8	0.12338382	3.16531920	1.46349037	1	-2.22441626	-0.78918064	-2.10520959
8	-0.92261618	1.24451935	-2.41480970	1	-1.11321616	-2.10958076	-1.74340963
6	-1.50231624	-0.80878067	-0.03970966				

tBuMA--MA-rad__re

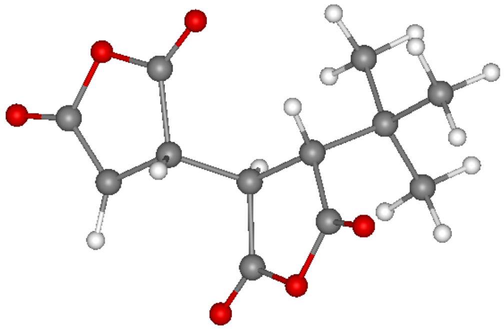

Zero-point vibrational energy	631790.7 (Joules/Mol)
	151.00161 (Kcal/Mol)
Zero-point correction=	0.240636 (Hartree/Particle)
Thermal correction to Energy=	0.257280
Thermal correction to Enthalpy=	0.258224
Thermal correction to Gibbs Free Energy=	0.195259
Sum of electronic and zero-point Energies=	-916.203502
Sum of electronic and thermal Energies=	-916.186859
Sum of electronic and thermal Enthalpies=	-916.185915
Sum of electronic and thermal Free Energies=	-916.248880

cartesian

6	-4.12842274	0.30385160	0.02854836	1	0.34857732	-0.54284835	1.44054830
6	-3.09032273	1.28755164	0.19224837	6	2.65367746	-1.37424839	0.43384838
6	-1.90362263	0.65535164	0.80794835	6	0.66497737	-1.84774840	-0.97165167
6	-2.36952257	-0.79184842	0.98324835	6	2.09677744	0.14625160	-1.47245169
8	-3.63932252	-0.92224848	0.51964837	1	-0.80472267	0.41585159	-1.03345168
8	-5.22932243	0.39015159	-0.42135167	1	-1.70932269	1.05885160	1.81214833
8	-1.76152277	-1.70044839	1.45264840	1	1.30177736	-2.50724840	-1.56575167
1	-3.18762255	2.31425166	-0.12365164	1	0.18227732	-2.45184827	-0.20165165
6	-0.22742268	2.24135160	-0.11255164	1	-0.11212268	-1.47344840	-1.64285159
6	-0.59902269	0.77185160	-0.02375163	1	2.67257738	-0.46754840	-2.16825175
6	0.62747735	0.11125159	0.61324835	1	1.32307720	0.64885163	-2.06045175
6	1.38957739	1.29345155	1.17574835	1	2.77357745	0.91045165	-1.08135164
8	0.88967735	2.47405171	0.63334835	1	3.27427745	-1.97664845	-0.23375164
8	-0.80042267	3.10965180	-0.68845165	1	3.29367733	-0.63464844	0.91374832
8	2.29117727	1.32375157	1.94344831	1	2.26467729	-2.03574824	1.21214843
6	1.51487732	-0.73024845	-0.36115164				

tBuMA--MA-rad__si

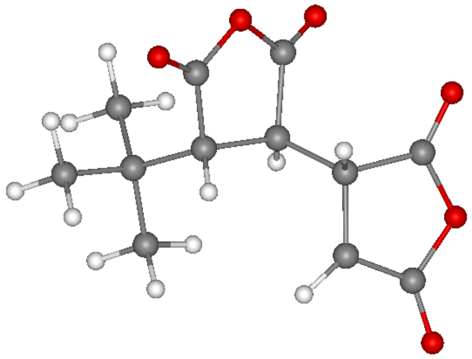

Zero-point vibrational energy	630480.3 (Joules/Mol)
	150.68840 (Kcal/Mol)
Zero-point correction=	0.240137 (Hartree/Particle)
Thermal correction to Energy=	0.256947
Thermal correction to Enthalpy=	0.257892
Thermal correction to Gibbs Free Energy=	0.194076
Sum of electronic and zero-point Energies=	-916.194882
Sum of electronic and thermal Energies=	-916.178072
Sum of electronic and thermal Enthalpies=	-916.177128
Sum of electronic and thermal Free Energies=	-916.240943

cartesian

6	3.77880001	0.77913225	0.31207421	1	-0.28370005	0.39953226	1.45347416
6	2.45810008	0.69723225	0.88877422	6	-2.72919989	1.08203232	0.58817422
6	1.93840003	-0.67876774	0.74227422	6	-0.80680007	1.92233229	-0.73802578
6	3.15670013	-1.41016769	0.17117423	6	-2.04270005	-0.12206772	-1.49242580
8	4.13969994	-0.49816772	-0.11612578	1	0.89300001	-0.21646774	-1.09582579
8	4.50780010	1.71583223	0.18197422	1	1.69429994	-1.14416766	1.70557415
8	3.29629993	-2.57406783	0.00997422	1	-1.48959994	2.56333232	-1.29992580
1	1.95729995	1.55333233	1.31477416	1	-0.44139999	2.50483227	0.11237422
6	0.26989996	-2.16026783	-0.50232577	1	0.04119998	1.71113229	-1.39352584
6	0.67499995	-0.73396772	-0.16232578	1	-2.71670008	0.50243223	-2.08232570
6	-0.55559999	-0.19776773	0.57957423	1	-1.23340011	-0.43316773	-2.15852571
6	-1.19919991	-1.47636771	1.09017420	1	-2.60229993	-1.01506782	-1.20302582
8	-0.72100002	-2.55196762	0.37237424	1	-3.39260006	1.72913218	0.00977422
8	0.66560000	-2.86206770	-1.36722577	1	-3.31080008	0.22953224	0.93737423
8	-2.00449991	-1.61706769	1.94987416	1	-2.40380001	1.64243221	1.46857417
6	-1.53880000	0.65933228	-0.27672577				

TS__tBuMA-rad_to_MA_transfer

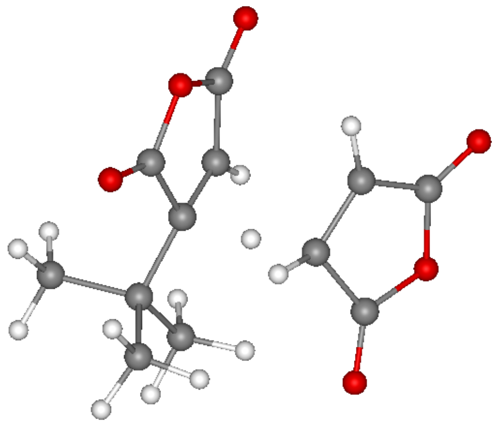

Zero-point vibrational energy	608891.8 (Joules/Mol)
	145.52864 (Kcal/Mol)
Zero-point correction=	0.231915 (Hartree/Particle)
Thermal correction to Energy=	0.249175
Thermal correction to Enthalpy=	0.250120
Thermal correction to Gibbs Free Energy=	0.185289
Sum of electronic and zero-point Energies=	-916.140774
Sum of electronic and thermal Energies=	-916.123513
Sum of electronic and thermal Enthalpies=	-916.122569
Sum of electronic and thermal Free Energies=	-916.187399

cartesian

6	3.59811306	0.47189677	-0.05378066	1	0.63771290	0.06589678	-0.48258066
6	2.66821289	0.75429678	-1.14338064	6	-1.42738700	-1.58550322	-0.85598063
6	1.77241278	-0.30180320	-1.23948073	6	-1.01748705	-1.35410321	1.59821928
6	2.31541300	-1.37220323	-0.33788067	6	-2.85908699	-0.05510321	0.51361930
8	3.31561303	-0.81960320	0.41271934	1	0.25741288	1.13739681	2.11361933
8	4.47571278	1.12459683	0.41191936	1	1.22961283	-0.57420325	-2.13808060
8	1.96751285	-2.50320315	-0.22108066	1	-1.66698694	-2.21910310	1.74561930
1	2.70991302	1.66329682	-1.72428071	1	0.00641286	-1.72790313	1.53631926
6	0.33811289	2.70479679	0.54071939	1	-1.10768723	-0.72820318	2.48921943
6	0.02671289	1.39729679	1.09181929	1	-3.54668689	-0.87480319	0.73421931
6	-0.51428717	0.58489680	0.08971934	1	-2.90338707	0.65359676	1.34421933
6	-0.74338716	1.53649676	-1.06998062	1	-3.21588707	0.44449678	-0.38818064
8	-0.11918712	2.71909690	-0.78498065	1	-2.18138695	-2.35900331	-0.69498062
8	0.85711288	3.65869689	1.02891934	1	-1.66238713	-1.08080316	-1.79278064
8	-1.30628705	1.34599674	-2.10038066	1	-0.46588713	-2.09350324	-0.95108068
6	-1.43858695	-0.61370325	0.32801935				

S6. MA – ET reaction profile

TS__tBuMA-rad_ET

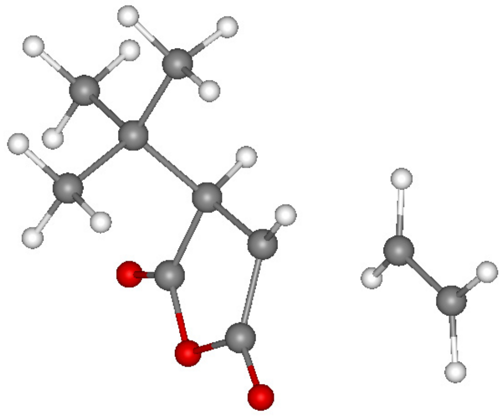

Zero-point vibrational energy	611266.2 (Joules/Mol)
	146.09612 (Kcal/Mol)
Zero-point correction=	0.232819 (Hartree/Particle)
Thermal correction to Energy=	0.247037
Thermal correction to Enthalpy=	0.247981
Thermal correction to Gibbs Free Energy=	0.191066
Sum of electronic and zero-point Energies=	-615.458433
Sum of electronic and thermal Energies=	-615.444216
Sum of electronic and thermal Enthalpies=	-615.443271
Sum of electronic and thermal Free Energies=	-615.500186

cartesian

6	3.81212139	0.83583570	0.52849638	1	1.18232131	0.97603571	-0.93970358
6	2.63582134	0.65053570	1.17889643	1	2.01852131	1.49973571	1.45079648
6	1.65922141	-1.15666437	-0.84530360	1	2.46062136	-0.26046434	1.74179649
6	1.06922138	0.07513568	-0.35720354	1	-2.52897882	1.92473578	-0.78580362
6	-0.13437864	-0.26296434	0.45619643	1	-1.35447860	2.17783570	0.50339639
6	0.03972137	-1.76656425	0.64039642	1	-0.81357861	1.94823575	-1.16650355
8	1.04642129	-2.21966434	-0.16930357	1	-2.65387869	-0.31896433	-1.97320354
8	2.52892160	-1.35346437	-1.63790357	1	-0.91327858	-0.39136434	-2.23000360
8	-0.56527865	-2.50396442	1.35209644	1	-1.74327862	-1.70296431	-1.38140357
6	-1.51257861	0.11363569	-0.18690357	1	-3.60397863	0.01553565	0.34649640
1	-0.11887863	0.19623569	1.45149648	1	-2.65167856	-1.32076430	1.00609648
6	-2.63827848	-0.25516433	0.78099644	1	-2.53817844	0.28343567	1.72759652
6	-1.54347861	1.62523580	-0.42130357	1	4.50022125	0.01753566	0.35729641
6	-1.70857871	-0.61876434	-1.51520348	1	4.06962156	1.79203558	0.08769643

TS__tBuMA-rad_to_ET_transfer

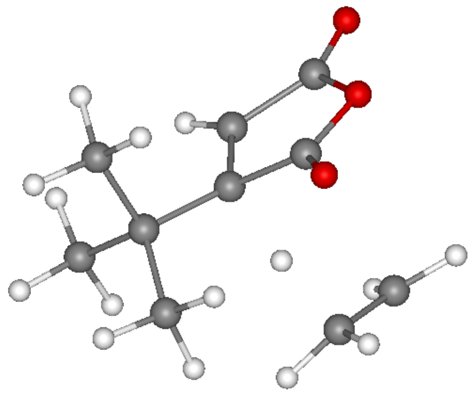

Zero-point vibrational energy	597009.5 (Joules/Mol)
	142.68871 (Kcal/Mol)
Zero-point correction=	0.227389 (Hartree/Particle)
Thermal correction to Energy=	0.241924
Thermal correction to Enthalpy=	0.242869
Thermal correction to Gibbs Free Energy=	0.185534
Sum of electronic and zero-point Energies=	-615.436718
Sum of electronic and thermal Energies=	-615.422183
Sum of electronic and thermal Enthalpies=	-615.421239
Sum of electronic and thermal Free Energies=	-615.478574

cartesian

6	-2.44815016	2.14842844	0.04092498	6	1.77494991	-1.86617136	-0.15187502
6	-1.12884998	2.15382838	-0.37677503	1	-1.04684997	-0.95837146	2.00922489
1	-3.25075006	1.85912848	-0.62667501	1	-0.92285001	2.18702841	-1.44407499
6	-2.41455007	-1.32567143	0.31022495	1	2.69745016	0.05922854	1.61962497
6	-1.17105007	-0.94827145	0.93762499	1	1.19075000	0.96282858	1.76982498
6	-0.28005001	-0.48687148	-0.03857501	1	1.26824999	-0.73157144	2.27312493
6	-0.93765002	-0.86137146	-1.34177494	1	2.86044979	-1.88717151	-0.02507502
8	-2.22874999	-1.22607148	-1.08077502	1	1.33885002	-2.58457160	0.54622495
8	-3.45945024	-1.68617153	0.76472497	1	1.55085003	-2.19557142	-1.16787505
8	-0.50045002	-0.82857144	-2.45157504	1	2.96945000	0.45492852	-0.82577503
6	1.24175000	-0.44817147	0.10402499	1	1.59744990	0.26892853	-1.92427492
1	-0.67774999	0.87142855	-0.14957501	1	1.59944999	1.55112863	-0.70537502
6	1.88114989	0.51412857	-0.90187502	1	-0.39865002	2.67102838	0.24172498
6	1.61195004	-0.01557145	1.52332509	1	-2.71694994	2.34812856	1.07132506

tBuMA--ET-rad

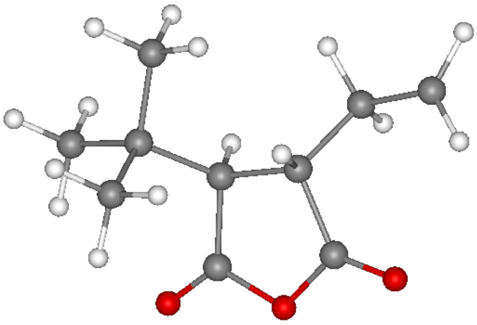

Zero-point vibrational energy	618240.7 (Joules/Mol)
	147.76306 (Kcal/Mol)
Zero-point correction=	0.235475 (Hartree/Particle)
Thermal correction to Energy=	0.249489
Thermal correction to Enthalpy=	0.250433
Thermal correction to Gibbs Free Energy=	0.194170
Sum of electronic and zero-point Energies=	-615.494491
Sum of electronic and thermal Energies=	-615.480478
Sum of electronic and thermal Enthalpies=	-615.479534
Sum of electronic and thermal Free Energies=	-615.535796

cartesian

6	3.35090351	1.22619653	0.23496072	1	1.14060366	0.32749641	-1.04203928
6	2.08500361	0.74149638	0.84076077	1	1.46100354	1.59089637	1.13536072
6	1.91430354	-1.49210358	-0.34293929	1	2.28670359	0.19529641	1.77786076
6	1.24040365	-0.16480356	-0.07023928	1	-3.49979639	0.25959641	0.18716072
6	-0.12889636	-0.54030359	0.50496072	1	-2.81329632	-1.29340363	0.68266076
6	-0.14149636	-2.05780363	0.45916072	1	-2.54519653	0.14599645	1.66616070
8	1.05780363	-2.52320361	-0.03403929	1	-2.39479637	0.09609640	-2.07533932
8	3.00530362	-1.70740366	-0.76093924	1	-0.66669637	-0.18330356	-2.22653937
8	-0.99389642	-2.81820369	0.78596073	1	-1.72649646	-1.47010350	-1.63313925
6	-1.37069631	0.11009645	-0.16843928	1	-2.07269645	2.10949636	-0.60113925
1	-0.16369638	-0.27560356	1.56926072	1	-1.06589627	2.02049637	0.84086072
6	-2.62879634	-0.22140357	0.63936073	1	-0.32929635	1.95289636	-0.76553923
6	-1.18909645	1.63019633	-0.17323929	1	3.93740368	0.58129638	-0.40403929
6	-1.54019642	-0.39400357	-1.60343921	1	3.79150367	2.15409636	0.57656074

TS__tBuMA--ET-rad_MA__re

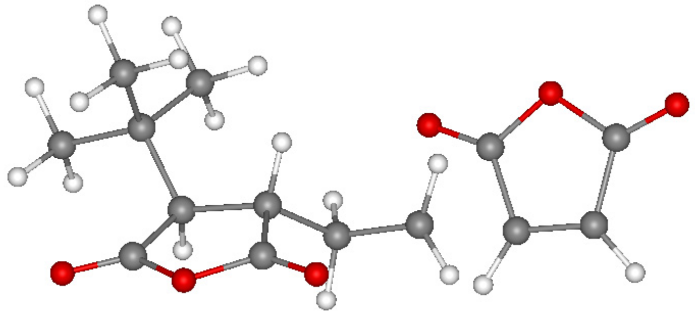

Zero-point vibrational energy	771662.5 (Joules/Mol)
	184.43176 (Kcal/Mol)
Zero-point correction=	0.293911 (Hartree/Particle)
Thermal correction to Energy=	0.313691
Thermal correction to Enthalpy=	0.314636
Thermal correction to Gibbs Free Energy=	0.241780
Sum of electronic and zero-point Energies=	-994.725949
Sum of electronic and thermal Energies=	-994.706169
Sum of electronic and thermal Enthalpies=	-994.705224
Sum of electronic and thermal Free Energies=	-994.778080

cartesian

6	-3.57126474	-0.70104325	0.44645947	1	1.71023500	-0.01204324	1.45595944
6	-3.77166510	0.57595676	1.17815948	6	3.39093494	-1.82444310	0.55615950
6	-4.88756514	1.15255678	0.68635947	6	1.07193506	-2.07984328	-0.29384053
6	-5.35556507	0.34495676	-0.44704053	6	2.79363489	-0.92904317	-1.69974053
8	-4.51016521	-0.76214325	-0.55154055	1	0.43193507	0.53065675	-1.19114053
8	-2.77056503	-1.56574321	0.62905949	1	-0.82186490	-0.20144323	0.85065949
8	-6.26656485	0.50695676	-1.19254053	1	-0.40466490	1.31805670	1.63885951
1	-3.23546505	0.80675673	2.08565927	1	3.65493488	-2.78764319	0.11265945
1	-5.38616514	2.05805683	0.99625945	1	4.29053497	-1.21364319	0.60915947
6	-1.78036487	1.49675679	0.01625946	1	3.05193520	-2.01524329	1.57845950
6	-0.60856491	0.85695672	0.66775948	1	3.03943491	-1.88584328	-2.16534066
6	1.12973511	2.34195685	-0.39124054	1	2.04493523	-0.44944325	-2.33664060
6	0.67013514	0.91555679	-0.19454055	1	3.69843483	-0.31694326	-1.71854055
6	1.88583505	0.19245675	0.39225948	1	1.35043514	-3.05044317	-0.71044052
6	2.98083496	1.24545670	0.35125947	1	0.68123507	-2.25784326	0.71135944
8	2.46633530	2.44465685	-0.10504054	1	0.25273508	-1.69394326	-0.90504050
8	0.49073511	3.28535676	-0.72984052	1	-2.08036494	1.14845669	-0.96694052
8	4.12473488	1.16315675	0.65625948	1	-2.05176497	2.51645684	0.25235948
6	2.29093504	-1.15444326	-0.27194053				

TS__tBuMA--ET-rad_MA__si

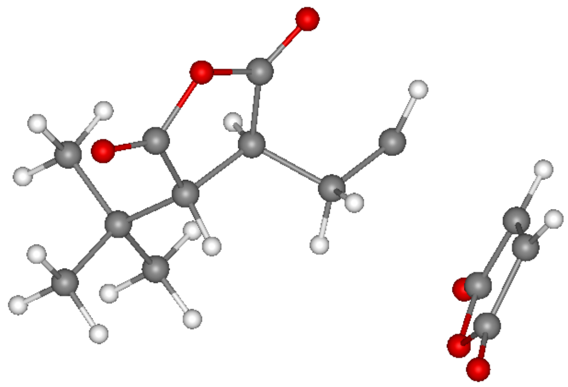

Zero-point vibrational energy	771335.4 (Joules/Mol)
	184.35358 (Kcal/Mol)
Zero-point correction=	0.293786 (Hartree/Particle)
Thermal correction to Energy=	0.313639
Thermal correction to Enthalpy=	0.314583
Thermal correction to Gibbs Free Energy=	0.241856
Sum of electronic and zero-point Energies=	-994.725824
Sum of electronic and thermal Energies=	-994.705971
Sum of electronic and thermal Enthalpies=	-994.705027
Sum of electronic and thermal Free Energies=	-994.777754

cartesian

6	3.94073534	0.05355948	-1.64965951	1	-0.89236486	-0.24504054	-1.43075943
6	4.16273499	1.31165957	-0.92495948	6	-2.90386486	-1.91034055	-1.13945949
6	4.10803509	1.04905951	0.39644051	6	-1.19706488	-2.07414055	0.65594053
6	3.97673535	-0.42364049	0.55504054	6	-3.27276468	-0.73034054	1.03394055
8	3.82523513	-0.96314055	-0.70275944	1	-0.81436491	0.61305952	1.43224049
8	3.85863519	-0.16564050	-2.81565928	1	1.11633515	-0.40774053	0.13754053
8	3.98503518	-1.08994055	1.54024053	1	1.18093514	1.05945945	-0.83135951
1	4.31133509	2.25285959	-1.43185949	1	-3.40526485	-2.79774046	-0.74525946
1	4.35623503	1.69225955	1.22624052	1	-3.64386463	-1.29924059	-1.65325952
6	1.78103518	1.31455946	1.19744051	1	-2.17156482	-2.24684048	-1.87875950
6	0.93293512	0.66895950	0.16274054	1	-3.75176477	-1.61494040	1.45924056
6	-0.98436487	2.31775951	0.21644054	1	-2.83986473	-0.17124051	1.86844051
6	-0.58266485	0.86985952	0.39414054	1	-4.06006479	-0.11874050	0.58744049
6	-1.48526490	0.09635949	-0.57275945	1	-1.69246495	-2.97744060	1.01884055
6	-2.42196465	1.16055942	-1.11745942	1	-0.42696485	-2.38934064	-0.05315946
8	-2.06426477	2.40075946	-0.62305945	1	-0.69996488	-1.61614060	1.51494050
8	-0.49066487	3.29105949	0.68854052	1	1.98393512	0.78685951	2.12264061
8	-3.33716464	1.05555952	-1.86525953	1	1.84223509	2.39425945	1.22684050
6	-2.22356486	-1.14684057	0.00014053				

tBuMA--ET-rad--MA__re

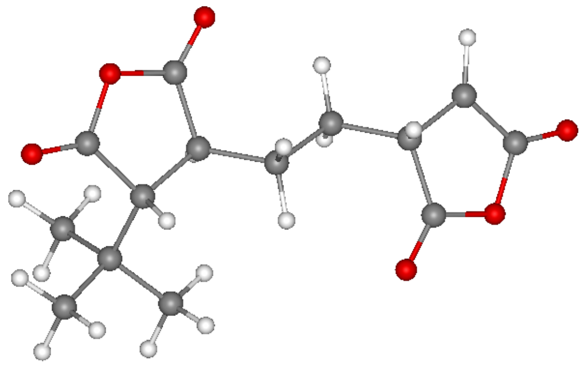

Zero-point vibrational energy	782586.3 (Joules/Mol)
	187.04260 (Kcal/Mol)
Zero-point correction=	0.298071 (Hartree/Particle)
Thermal correction to Energy=	0.317402
Thermal correction to Enthalpy=	0.318346
Thermal correction to Gibbs Free Energy=	0.247701
Sum of electronic and zero-point Energies=	-994.775591
Sum of electronic and thermal Energies=	-994.756260
Sum of electronic and thermal Enthalpies=	-994.755316
Sum of electronic and thermal Free Energies=	-994.825962

cartesian

6	-3.65754867	-0.66955131	0.69181621	1	1.57265115	-0.16155133	1.51591611
6	-3.28884864	0.81004870	0.65081620	6	3.41745114	-1.77865124	0.57521623
6	-4.49514866	1.40504861	0.04281623	6	1.18375134	-2.08785129	-0.46328378
6	-5.46634865	0.37624866	-0.22408378	6	2.91475129	-0.70755130	-1.62718368
8	-4.91464853	-0.84615129	0.18801622	1	0.44275135	0.56344867	-1.15668368
8	-2.99704885	-1.58205128	1.07561624	1	-0.83724868	-0.49265134	0.80431622
8	-6.56014872	0.43314865	-0.70038378	1	-0.69534868	1.04354858	1.63041615
1	-3.16514874	1.15144873	1.68911624	1	3.77815127	-2.67395139	0.06281622
1	-4.66484833	2.44654870	-0.18628377	1	4.26665163	-1.12135124	0.75461620
6	-1.97504878	1.08994865	-0.10458377	1	3.02185130	-2.09205127	1.54561615
6	-0.74414867	0.58104867	0.63771623	1	3.25465131	-1.59955144	-2.15778375
6	0.93355137	2.31594872	-0.11468378	1	2.17935133	-0.21655136	-2.27098370
6	0.56865138	0.84714866	-0.10578378	1	3.77565122	-0.04295135	-1.52488375
6	1.79875135	0.15264866	0.48891622	1	1.55495119	-2.99335122	-0.94878381
6	2.81795120	1.27014863	0.61821622	1	0.73945135	-2.38775134	0.48921624
8	2.24005127	2.47554874	0.26561624	1	0.38625133	-1.69015121	-1.09558368
8	0.25305134	3.25234866	-0.39068377	1	-2.03014874	0.62964869	-1.09678388
8	3.95005107	1.23124862	0.97221625	1	-1.89264870	2.16574860	-0.26498377
6	2.33395123	-1.09725142	-0.26558378				

tBuMA--ET-rad--MA__si

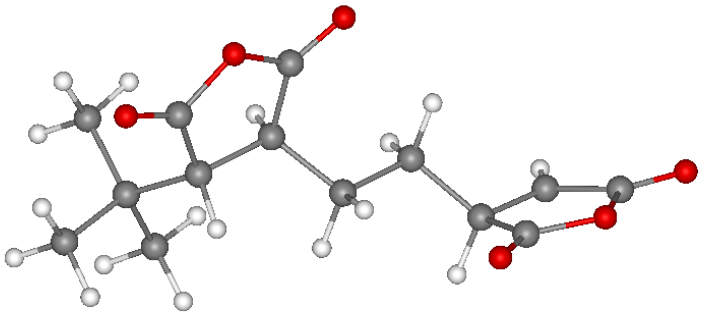

Zero-point vibrational energy	782250.9 (Joules/Mol)
	186.96246 (Kcal/Mol)
Zero-point correction=	0.297944 (Hartree/Particle)
Thermal correction to Energy=	0.317336
Thermal correction to Enthalpy=	0.318280
Thermal correction to Gibbs Free Energy=	0.246976
Sum of electronic and zero-point Energies=	-994.773995
Sum of electronic and thermal Energies=	-994.754602
Sum of electronic and thermal Enthalpies=	-994.753658
Sum of electronic and thermal Free Energies=	-994.824963

cartesian

6	5.66526222	0.30315942	0.21770811	1	-1.36363792	0.24065942	-1.55519187
6	4.57176208	-0.43554062	0.79460812	6	-3.72363806	-0.84204054	-1.13239193
6	3.39286208	-0.36064059	-0.09089188	6	-1.86713791	-1.97384048	0.06390812
6	3.91656208	0.50795943	-1.23209190	6	-3.28863788	-0.30504060	1.26970816
8	5.21976185	0.84345937	-0.99579191	1	-0.49673790	0.15455940	1.29960811
8	6.77966213	0.48445940	0.60740811	1	0.73126215	-0.88014054	-0.66119188
8	3.33556199	0.87305939	-2.20309186	1	1.09106207	0.72715938	-1.24869192
1	4.64796209	-0.94814062	1.74200809	1	-4.40403795	-1.64364052	-0.83409190
1	3.15576220	-1.34804058	-0.51449192	1	-4.30673790	0.06525940	-1.27899194
6	2.12086225	0.20025942	0.57390809	1	-3.28723788	-1.12134063	-2.09569192
6	0.90066212	0.15245941	-0.33939189	1	-3.94723797	-1.11484063	1.59130812
6	-0.34933788	2.13305950	0.62300813	1	-2.55503798	-0.15514058	2.06710815
6	-0.38703781	0.64785939	0.32750812	1	-3.89793777	0.59835941	1.19530809
6	-1.65073800	0.45865941	-0.51899189	1	-2.55493784	-2.78124070	0.32550812
6	-2.27773786	1.84135938	-0.55039191	1	-1.38183784	-2.25134063	-0.87599188
8	-1.47023797	2.73935938	0.11770812	1	-1.10233784	-1.93884063	0.84420812
8	0.48086214	2.75665951	1.20240808	1	1.92796206	-0.39464056	1.47310817
8	-3.29083800	2.20295954	-1.05219197	1	2.30196214	1.22095942	0.91310811
6	-2.63673782	-0.65724063	-0.06959188				

TS__tBuMA--ET-rad_ET

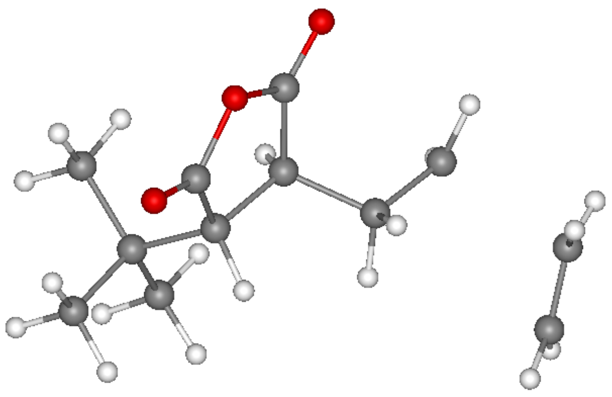

Zero-point vibrational energy	759114.5 (Joules/Mol)
	181.43273 (Kcal/Mol)
Zero-point correction=	0.289131 (Hartree/Particle)
Thermal correction to Energy=	0.306272
Thermal correction to Enthalpy=	0.307217
Thermal correction to Gibbs Free Energy=	0.241758
Sum of electronic and zero-point Energies=	-694.012833
Sum of electronic and thermal Energies=	-693.995692
Sum of electronic and thermal Enthalpies=	-693.994748
Sum of electronic and thermal Free Energies=	-694.060206

cartesian

1	4.66230583	-1.92041755	-0.18560591	1	-0.71139407	-0.31801766	-1.38870597
6	4.37940598	-1.12311769	-0.86390591	6	-3.15199399	-1.19981766	-0.81570590
6	4.50700617	0.17658234	-0.52100593	6	-1.37359416	-1.86911750	0.77989411
1	5.05850601	0.46668231	0.36559409	6	-2.84979391	0.08668232	1.30539405
1	3.94590616	-1.42181778	-1.81200600	1	0.01250592	0.41958234	1.34969401
1	4.34020615	0.96118236	-1.25050592	1	1.49590588	-0.61161762	-0.39970589
1	2.84890604	1.96498227	0.61509413	1	1.51990592	0.91738236	-1.25740600
6	2.58450603	0.91458237	0.59479409	1	-3.85429406	-1.87051749	-0.31420591
6	1.44860590	0.47298232	-0.25790590	1	-3.72099400	-0.39661765	-1.28130591
6	-0.19149408	2.27818227	0.37599409	1	-2.66519403	-1.76761770	-1.61360598
6	0.04660593	0.78758234	0.32279411	1	-3.53989410	-0.57971770	1.82769406
6	-1.10099411	0.24758232	-0.53570592	1	-2.16159415	0.48648232	2.05519414
6	-1.73889410	1.50218225	-1.10280597	1	-3.43959403	0.91828233	0.91129404
8	-1.20229411	2.61758232	-0.49740592	1	-2.07869411	-2.56241751	1.24459410
8	0.37010592	3.10488248	1.01779401	1	-0.83459407	-2.42041755	0.00449409
8	-2.57719398	1.61058235	-1.93810594	1	-0.65349406	-1.58081770	1.54939401
6	-2.12629414	-0.67281765	0.19129410	1	2.75190592	0.38088232	1.52459407

tBuMA--ET-rad—ET

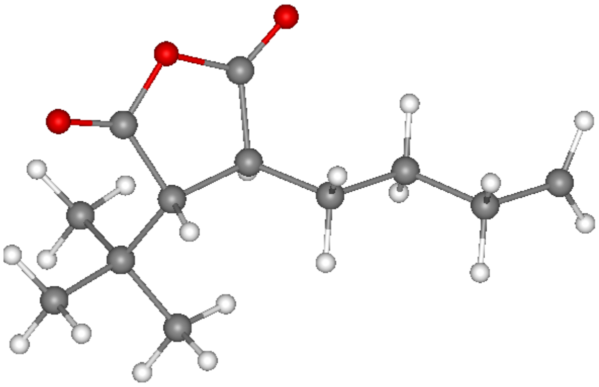

Zero-point vibrational energy	767698.6 (Joules/Mol)
	183.48438 (Kcal/Mol)
Zero-point correction=	0.292401 (Hartree/Particle)
Thermal correction to Energy=	0.309532
Thermal correction to Enthalpy=	0.310477
Thermal correction to Gibbs Free Energy=	0.245611
Sum of electronic and zero-point Energies=	-694.062104
Sum of electronic and thermal Energies=	-694.044972
Sum of electronic and thermal Enthalpies=	-694.044028
Sum of electronic and thermal Free Energies=	-694.108894

cartesian

1	-5.60569096	0.80683827	-0.21467647	1	1.09990895	0.16813827	1.53972363
6	-5.09909105	-0.13576174	-0.04527646	6	3.34620881	-0.94966173	0.74602354
6	-3.80629110	-0.17696172	0.68512356	6	1.31000900	-1.92916167	-0.28177646
1	-3.86869097	0.44453827	1.58772361	6	2.63800883	-0.21826172	-1.53587639
1	-5.49239111	-1.01166177	-0.54587644	1	-0.11919108	0.32033825	-1.17827642
1	-3.59989119	-1.19706178	1.02732360	1	-1.11109102	-0.77646172	0.91392356
1	-2.80739117	1.34253824	-0.49027646	1	-1.38819110	0.83243823	1.54382360
6	-2.61869121	0.32063827	-0.15637647	1	3.95010877	-1.74916184	0.30942354
6	-1.30519104	0.25823826	0.61332351	1	3.98000884	-0.07466173	0.87882352
6	-0.09069109	2.25143814	-0.36337647	1	3.02270889	-1.28376174	1.73622358
6	-0.08219108	0.75003827	-0.17157647	1	3.22040892	-1.02016175	-1.99497640
6	1.26710892	0.45793825	0.49482355	1	1.81740892	0.01293826	-2.22077656
6	1.95000899	1.81253827	0.54942352	1	3.28570890	0.65913826	-1.47337639
8	1.11110890	2.78203821	0.04602354	1	1.92320895	-2.73366165	-0.69437647
8	-0.95169103	2.94973850	-0.79087645	1	0.94200897	-2.26586151	0.69142354
8	3.02830887	2.10193825	0.95622355	1	0.44990891	-1.80776179	-0.94467646
6	2.14330888	-0.65146172	-0.15377647	1	-2.53909111	-0.28996176	-1.06317639

S7. MA – BU reaction profile

TS__tBuMA-rad_BU

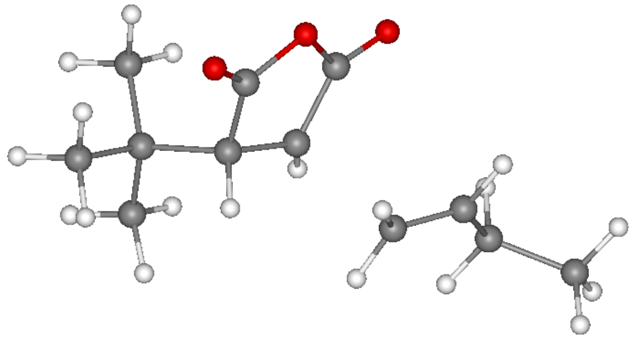

Zero-point vibrational energy	762664.9 (Joules/Mol)
	182.28129 (Kcal/Mol)
Zero-point correction=	0.290484 (Hartree/Particle)
Thermal correction to Energy=	0.307411
Thermal correction to Enthalpy=	0.308355
Thermal correction to Gibbs Free Energy=	0.244114
Sum of electronic and zero-point Energies=	-694.033360
Sum of electronic and thermal Energies=	-694.016433
Sum of electronic and thermal Enthalpies=	-694.015489
Sum of electronic and thermal Free Energies=	-694.079730

cartesian

6	3.68988824	0.32767951	-0.21853530	6	-2.01851177	1.94587958	-0.36493531
6	2.83058834	-0.59212053	0.57696474	6	-2.84511161	-0.12352049	-1.50223529
6	1.67948830	-0.23592049	1.20636463	1	0.39198828	0.51697946	-0.90543526
6	5.16668797	0.24177951	0.16596469	1	1.43818831	0.81157947	1.35846460
1	3.58378839	0.06177950	-1.27873540	1	1.17878830	-0.93832052	1.86386466
1	5.54798794	-0.77462053	0.04356469	1	3.33268833	1.35737956	-0.11753531
1	5.32188797	0.53187954	1.20716465	1	-2.86771178	2.53477955	-0.71963531
6	0.21278830	-1.65932047	-0.84423530	1	-1.67431164	2.39757967	0.56986469
6	0.01118828	-0.31862050	-0.33873531	1	-1.22311163	2.04987955	-1.10593534
6	-1.24221170	-0.29442048	0.46916467	1	-3.65641165	0.45297951	-1.95273530
6	-1.52741170	-1.78482056	0.62296468	1	-2.01491165	-0.13052049	-2.21343517
8	-0.69711173	-2.50352049	-0.19353530	1	-3.20141172	-1.15052056	-1.38883531
8	0.99938828	-2.09632063	-1.62983537	1	-4.46661186	1.01927948	0.38266468
8	-2.33131170	-2.32042050	1.31886470	1	-3.96691155	-0.56082052	1.00056469
6	-2.44261169	0.48997951	-0.16063531	1	-3.37271166	0.91257954	1.76356471
1	-1.09281170	0.11877948	1.47366464	1	5.77188826	0.89917946	-0.46113533
6	-3.62971163	0.45597953	0.80366468	1	3.11368823	-1.64212048	0.56876469

TS__tBuMA-rad_to_BU_transfer

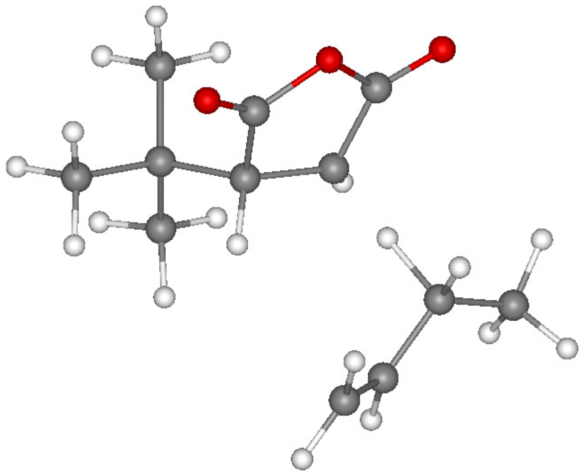

Zero-point vibrational energy	749999.6 (Joules/Mol)
	179.25421 (Kcal/Mol)
Zero-point correction=	0.285660 (Hartree/Particle)
Thermal correction to Energy=	0.302774
Thermal correction to Enthalpy=	0.303719
Thermal correction to Gibbs Free Energy=	0.239118
Sum of electronic and zero-point Energies=	-694.027145
Sum of electronic and thermal Energies=	-694.010031
Sum of electronic and thermal Enthalpies=	-694.009086
Sum of electronic and thermal Free Energies=	-694.073687

cartesian

6	3.54728818	0.82002354	0.84685290	6	-2.80751181	-1.28207648	-0.58564711
6	2.72048831	0.23652354	-0.27344707	6	-1.59871161	-1.14557648	1.58095288
6	2.70338821	-1.21247649	-0.43454707	6	-2.87261176	0.83842349	0.74385285
6	2.56318831	-1.85487652	-1.60334706	1	0.28958833	0.94442356	1.46455288
1	1.51148844	0.57762355	0.03675294	1	2.53948832	-2.93677640	-1.65664709
1	3.32898831	1.87982357	0.98765290	1	2.47978830	-1.31667638	-2.54234695
1	3.35868835	0.30592352	1.79265296	1	-2.48361182	-1.56467640	2.06585312
1	4.61578846	0.72592354	0.63055289	1	-0.93751168	-1.97827649	1.32595289
6	0.13278836	2.34712338	-0.18684706	1	-1.09091163	-0.52427649	2.32195306
6	0.20308836	1.00592351	0.38635293	1	-3.75291181	0.49812353	1.29405296
6	-0.71681166	0.11912352	-0.40024707	1	-2.33061171	1.53202355	1.39235294
6	-1.01091158	0.98872352	-1.61604714	1	-3.23101163	1.39732349	-0.12454706
8	-0.53661162	2.25932360	-1.40744710	1	-3.70531178	-1.63537645	-0.07184707
8	0.57598835	3.38582349	0.19905293	1	-3.11371160	-0.78067648	-1.50284708
8	-1.56101155	0.69462353	-2.62924695	1	-2.21931171	-2.15967655	-0.86814702
6	-2.01021171	-0.35677645	0.33565292	1	2.82428837	0.77442354	-1.21854711
1	-0.20491163	-0.78177643	-0.76104707	1	2.78988838	-1.80127645	0.47775295

tBuMA--BU-rad

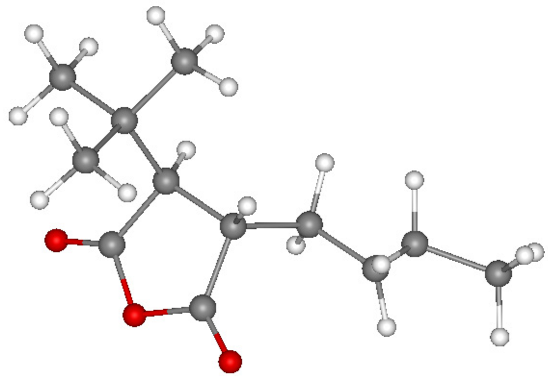

Zero-point vibrational energy	769682.1 (Joules/Mol)
	183.95844 (Kcal/Mol)
Zero-point correction=	0.293156 (Hartree/Particle)
Thermal correction to Energy=	0.310006
Thermal correction to Enthalpy=	0.310951
Thermal correction to Gibbs Free Energy=	0.246822
Sum of electronic and zero-point Energies=	-694.067484
Sum of electronic and thermal Energies=	-694.050634
Sum of electronic and thermal Enthalpies=	-694.049690
Sum of electronic and thermal Free Energies=	-694.113818

cartesian

6	2.62458539	-0.69595885	0.72733235	6	-3.51641464	0.73564112	0.49943236
6	3.30368543	0.28844118	-0.15636766	6	-1.45831466	1.97934127	-0.11336765
6	4.82478523	0.25274116	-0.04686765	6	-2.35271454	0.21354115	-1.65036774
6	1.22138536	-0.50635886	1.17573237	1	0.41588533	0.10384119	-0.73326761
1	2.93728542	1.30004120	0.06223235	1	1.09718537	0.49254119	1.61383235
1	3.02348542	0.09714115	-1.20556772	1	0.96818531	-1.22395885	1.96273232
1	5.21138525	-0.73745883	-0.29966766	1	-4.12581444	1.48634124	-0.01006765
1	5.15298510	0.48724115	0.96813238	1	-4.06171465	-0.20705885	0.49393234
6	0.15978535	-1.98365879	-0.56746763	1	-3.40681458	1.04944122	1.54123235
6	0.15258537	-0.60785884	0.05003235	1	-2.92641449	0.97894120	-2.17796779
6	-1.27771461	-0.42895883	0.56463236	1	-1.40561461	0.09774119	-2.18426776
6	-1.85211468	-1.83175886	0.48413235	1	-2.90721464	-0.72415882	-1.73976767
8	-0.99961466	-2.64815879	-0.22746766	1	-2.09381461	2.74734116	-0.56046760
8	0.99808538	-2.50225878	-1.23016763	1	-1.26891470	2.27294111	0.92283237
8	-2.87031460	-2.25575876	0.92633235	1	-0.50631464	2.00004125	-0.64866763
6	-2.15371466	0.61704111	-0.18786766	1	3.08208537	-1.67185879	0.85023236
1	-1.27861464	-0.14635885	1.62243235	1	5.28878546	0.97124112	-0.72556764

TS__tBuMA--BU-rad_MA__re

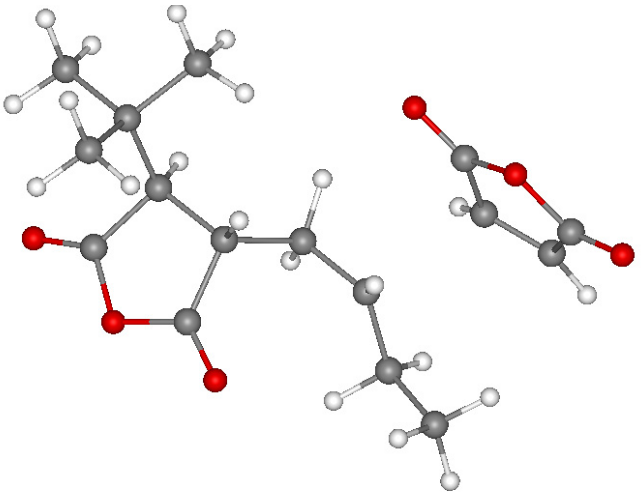

Zero-point vibrational energy	922178.3 (Joules/Mol)
	220.40590 (Kcal/Mol)
Zero-point correction=	0.351239 (Hartree/Particle)
Thermal correction to Energy=	0.373835
Thermal correction to Enthalpy=	0.374779
Thermal correction to Gibbs Free Energy=	0.295579
Sum of electronic and zero-point Energies=	-1073.299150
Sum of electronic and thermal Energies=	-1073.276554
Sum of electronic and thermal Enthalpies=	-1073.275610
Sum of electronic and thermal Free Energies=	-1073.354810

cartesian

6	-2.84317684	-1.52212799	0.23166049	8	4.23722315	1.16877210	0.90656048
6	-3.19147682	-0.43402794	1.17756045	6	2.81902361	-1.38252795	-0.28293952
6	-4.44477654	-0.02112792	0.86826050	1	2.05972338	-0.40802792	1.47836053
6	-4.85667658	-0.70052791	-0.36093953	6	4.04842329	-1.87162793	0.48766047
8	-3.83557653	-1.59302795	-0.70963955	6	1.77212346	-2.49892783	-0.27643952
8	-1.89257658	-2.24512792	0.21896046	6	3.22102356	-1.04782796	-1.72113955
8	-5.84157658	-0.59602797	-1.01953948	1	0.62592340	-0.12752791	-1.07303953
1	-2.64827681	-0.27972791	2.09726048	1	-0.42297661	-0.59702790	1.08206046
1	-5.07407665	0.69567204	1.37266052	1	-0.01067662	1.00617206	1.69276047
6	-2.03567648	2.34707212	0.55076045	1	4.44662333	-2.77312803	0.01546048
6	-1.52157664	0.99597204	0.17956048	1	4.84082317	-1.12532794	0.51856047
6	-0.27287662	0.44737208	0.78776050	1	3.79092312	-2.12672806	1.51946044
6	-3.03777647	2.92077208	-0.43933952	1	3.61552334	-1.93782783	-2.21593952
1	-2.46107674	2.32227206	1.56376052	1	2.37782335	-0.69822788	-2.32293963
1	-3.91157675	2.27597213	-0.55713952	1	4.00302315	-0.28462791	-1.75703955
1	-2.57567644	3.04147196	-1.42083955	1	2.18532324	-3.39612794	-0.74313951
6	1.30032337	1.81817210	-0.64173955	1	1.47972333	-2.76582789	0.74236047

6	0.92912340	0.42747208	-0.18243951	1	0.86102343	-2.24422789	-0.82093954
6	2.21612358	-0.13422792	0.42956048	1	-1.17677665	3.02667212	0.62726045
6	3.15752316	1.05567205	0.42776048	1	-1.75397658	0.64437211	-0.82303953
8	2.58052349	2.11347198	-0.25423953	1	-3.39207649	3.90117216	-0.11643951
8	0.63302338	2.60297203	-1.23713958				

TS__tBuMA--BU-rad_MA__si

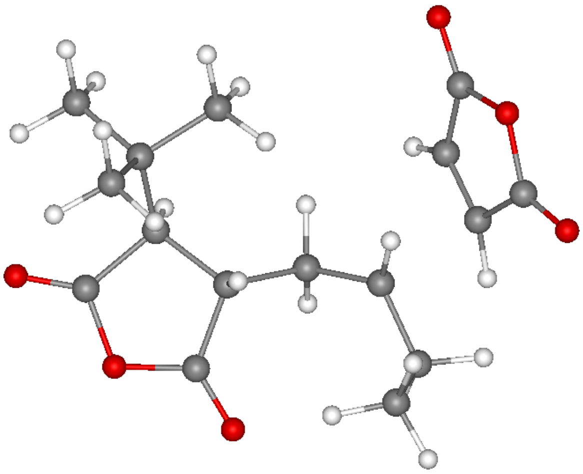

Zero-point vibrational energy	923788.9 (Joules/Mol)
	220.79085 (Kcal/Mol)
Zero-point correction=	0.351853 (Hartree/Particle)
Thermal correction to Energy=	0.374222
Thermal correction to Enthalpy=	0.375166
Thermal correction to Gibbs Free Energy=	0.297397
Sum of electronic and zero-point Energies=	-1073.295142
Sum of electronic and thermal Energies=	-1073.272772
Sum of electronic and thermal Enthalpies=	-1073.271828
Sum of electronic and thermal Free Energies=	-1073.349597

cartesian

6	-3.45575595	-1.89913714	0.69739997	8	1.34734416	3.05266285	0.82040000
6	-3.08505583	-0.92463714	1.72580004	8	4.41164398	-0.14113709	0.94669998
6	-3.36015582	0.31526291	1.24979997	6	2.10444403	-1.59863710	-0.46290001
6	-4.09445572	0.13956290	-0.02799998	1	1.72254419	-0.86293709	1.52759993
8	-4.05545568	-1.19933712	-0.34860000	6	2.93314409	-2.73583722	0.14300001
8	-3.31115603	-3.08083701	0.65840006	6	0.70134413	-2.13213706	-0.76300001
8	-4.64135599	0.94556290	-0.71319997	6	2.75824404	-1.13073707	-1.76550007
1	-2.64395595	-1.21493709	2.66709995	1	0.92604423	0.78856289	-0.86039996
1	-3.37045598	1.25056291	1.78830004	1	-0.57765579	-0.43873709	1.06060004
6	-1.82775581	2.61806297	-0.04239999	1	-0.35155582	1.11596286	1.84749997
6	-1.47785580	1.17096293	0.03440002	1	2.98514414	-3.57273722	-0.55760002

6	-0.37455583	0.61846286	0.87349999	1	3.94924402	-2.41993713	0.37330002
6	-1.25085580	3.30026293	-1.28980005	1	2.47564411	-3.10813713	1.06430006
1	-2.91795588	2.71616292	-0.09329998	1	2.78754401	-1.95273709	-2.48379993
1	-1.58265579	2.79746294	-2.20110011	1	2.20694399	-0.31473708	-2.24210000
1	-1.59405577	4.33536291	-1.34070003	1	3.78614402	-0.80043709	-1.60500002
1	-0.16135582	3.31666279	-1.27050006	1	0.76644421	-2.97553706	-1.45370007
6	1.76944423	1.95256293	0.66360003	1	0.19904417	-2.49483705	0.13790001
6	1.03024423	0.70776290	0.22750002	1	0.05804417	-1.38433707	-1.23390007
6	2.00314403	-0.43173710	0.55680001	1	-1.48515582	3.13906288	0.85479999
6	3.31594419	0.29116291	0.80620003	1	-1.70555580	0.58966291	-0.85940003
8	3.08704400	1.65246284	0.88999999				

tBuMA--BU-rad--MA__re

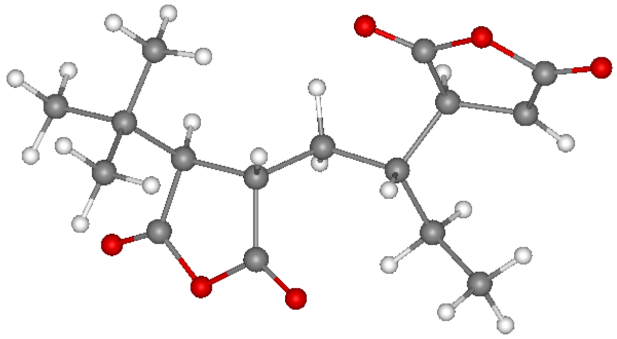

Zero-point vibrational energy	932871.3 (Joules/Mol)
	222.96159 (Kcal/Mol)
Zero-point correction=	0.355312 (Hartree/Particle)
Thermal correction to Energy=	0.377343
Thermal correction to Enthalpy=	0.378287
Thermal correction to Gibbs Free Energy=	0.301793
Sum of electronic and zero-point Energies=	-1073.335985
Sum of electronic and thermal Energies=	-1073.313954
Sum of electronic and thermal Enthalpies=	-1073.313010
Sum of electronic and thermal Free Energies=	-1073.389504

cartesian

6	-2.83272314	-1.50571871	0.07186279	8	3.89057684	1.43748128	0.94026279
6	-2.88012314	-0.15121862	0.77666277	6	2.85807705	-1.33861864	-0.22023720
6	-4.27632332	0.25998136	0.53416276	1	1.97507679	-0.41691864	1.51056278
6	-4.95962334	-0.74801862	-0.23223722	6	4.16347694	-1.60701871	0.53296280
8	-4.04422331	-1.78091872	-0.48843721	6	1.98397696	-2.59221864	-0.12933721
8	-1.90632308	-2.24691868	-0.03603721	6	3.17157698	-1.02301872	-1.68433726
8	-6.08512306	-0.80681860	-0.62713724	1	0.44707692	-0.47761860	-0.96363723

1	-2.71892309	-0.31381863	1.85266280	1	-0.49682310	-0.73311859	1.23116279
1	-4.78142309	1.13148129	0.91996276	1	-0.09932311	0.91058141	1.69336283
6	-2.00852299	2.25498128	0.63086277	1	4.67117691	-2.46911860	0.09346279
6	-1.73852313	0.78348142	0.27876279	1	4.84557676	-0.75841862	0.49856281
6	-0.39422309	0.28198138	0.84526277	1	3.97317696	-1.83671868	1.58486283
6	-2.81942320	3.01088142	-0.41283721	1	3.68347692	-1.86911869	-2.14783716
1	-2.48522329	2.31288123	1.61766279	1	2.27097702	-0.83421862	-2.27463698
1	-3.78112316	2.53878140	-0.62863719	1	3.82937670	-0.15511861	-1.78383720
1	-2.26332307	3.06608129	-1.35003722	1	2.50977707	-3.44341874	-0.56843722
6	1.01457691	1.55158138	-0.83733720	1	1.76317680	-2.84561872	0.91106278
6	0.74997687	0.21648139	-0.17793721	1	1.03127694	-2.49481869	-0.65313721
6	2.09457684	-0.15801862	0.45406276	1	-1.04942310	2.76678133	0.74546278
6	2.87287712	1.14198136	0.40596280	1	-1.70722306	0.70638138	-0.81233722
8	2.21907711	2.04918122	-0.41053721	1	-3.02342319	4.03248167	-0.08573721
8	0.33247691	2.15308142	-1.60493720				

tBuMA--BU-rad--MA__si

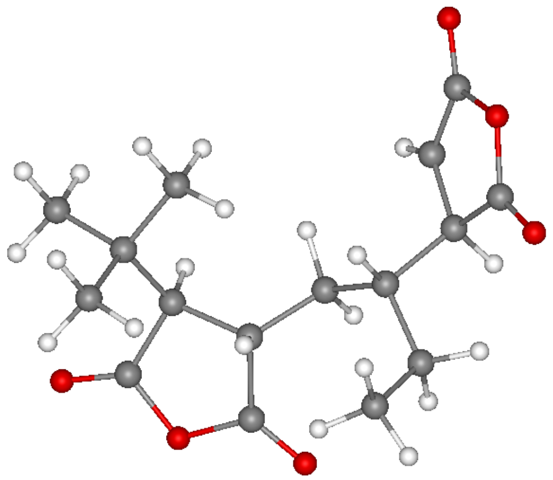

Zero-point vibrational energy	932748.5 (Joules/Mol)
	222.93225 (Kcal/Mol)
Zero-point correction=	0.355265 (Hartree/Particle)
Thermal correction to Energy=	0.377253
Thermal correction to Enthalpy=	0.378197
Thermal correction to Gibbs Free Energy=	0.301572
Sum of electronic and zero-point Energies=	-1073.330848
Sum of electronic and thermal Energies=	-1073.308860
Sum of electronic and thermal Enthalpies=	-1073.307916
Sum of electronic and thermal Free Energies=	-1073.384542

cartesian

6	4.05577660	1.58646274	0.87771165	8	-1.21452332	-2.97493720	1.20251167
6	3.01847672	0.80646276	1.50201154	8	-4.38292313	0.10786276	0.99811167

6	2.95437670	-0.53573728	0.88981164	6	-2.10372329	1.53496277	-0.46788836
6	4.13557673	-0.47973725	-0.08088838	1	-1.72702336	0.95266283	1.57281160
8	4.68127680	0.77716279	-0.07238837	6	-2.97642326	2.68336272	0.04931164
8	4.40127659	2.71556282	1.06321156	6	-0.71542329	2.09386277	-0.78748840
8	4.56357670	-1.33743715	-0.78268832	6	-2.72312331	0.95996284	-1.74458838
1	2.44637656	1.18246281	2.33601165	1	-0.86852330	-0.84843719	-0.68188834
1	3.14767671	-1.32013726	1.63431156	1	0.54147673	0.56956279	1.23931158
6	1.67637682	-2.42153716	-0.20268838	1	0.47967672	-1.01993728	1.97151160
6	1.62927675	-0.92383718	0.15121163	1	-3.04722333	3.46886277	-0.70708835
6	0.43487674	-0.49293724	1.01251161	1	-3.98412323	2.34956288	0.29041162
6	0.91457671	-2.82603717	-1.45898843	1	-2.54342341	3.13236284	0.94811165
1	2.71997666	-2.70023727	-0.35228837	1	-2.76812339	1.73086274	-2.51678824
1	1.22367668	-2.22823715	-2.32088828	1	-2.13822341	0.13286276	-2.15718818
1	1.12257671	-3.87013721	-1.70028841	1	-3.74212313	0.60726273	-1.57598841
1	-0.16772327	-2.74493718	-1.35368836	1	-0.79852325	2.88966274	-1.53088844
6	-1.66722322	-1.90903723	0.93631166	1	-0.23852324	2.52706289	0.09601164
6	-0.96212322	-0.68343723	0.39651161	1	-0.04422328	1.33766282	-1.20168841
6	-1.97732329	0.44546273	0.63061166	1	1.31247663	-2.99823713	0.65221161
6	-3.27122331	-0.29873723	0.91341168	1	1.60297680	-0.34043723	-0.77848834
8	-3.00052333	-1.63783717	1.11081159				

TS__tBuMA--BU-rad_BU

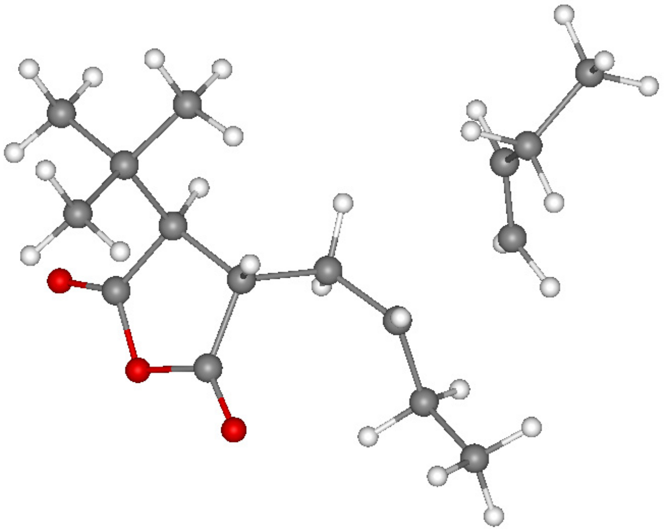

Zero-point vibrational energy	1061022.7 (Joules/Mol)
	253.59052 (Kcal/Mol)
Zero-point correction=	0.404122 (Hartree/Particle)
Thermal correction to Energy=	0.426614
Thermal correction to Enthalpy=	0.427558
Thermal correction to Gibbs Free Energy=	0.349416
Sum of electronic and zero-point Energies=	-851.154979

Sum of electronic and thermal Energies= -851.132487
Sum of electronic and thermal Enthalpies= -851.131543
Sum of electronic and thermal Free Energies= -851.209685

cartesian

6	-3.63024569	-1.43711519	-0.46926734	6	4.02855396	-2.00751519	0.61883271
6	-3.01854587	-0.85521519	0.76383269	6	1.80415428	-2.33961511	-0.42826733
6	-2.98804569	0.47018483	1.05933261	6	3.52195430	-0.92001522	-1.57386732
1	-3.62214565	1.16238475	0.51553267	1	0.90505421	0.16498484	-1.06696737
1	-2.52114582	-1.55651522	1.43253267	1	-0.31254578	-0.22601515	1.03273261
1	-2.65884566	0.80388486	2.03743267	1	0.26295424	1.29128480	1.69893265
6	-1.44284582	2.94518495	0.45793265	1	4.39975452	-2.89581513	0.10153267
6	-1.18284571	1.50408483	0.13493267	1	4.87215424	-1.34461522	0.80593270
6	-0.02424580	0.79688483	0.76333266	1	3.63745427	-2.32781506	1.58843267
6	-2.35474586	3.64508486	-0.53936732	1	3.88835430	-1.79821515	-2.11026740
1	-1.85854578	3.03058481	1.47213268	1	2.78145432	-0.43771517	-2.21766734
1	-3.32714581	3.15278482	-0.61906731	1	4.36715412	-0.23611516	-1.46026742
1	-2.53544569	4.68258476	-0.25086734	1	2.18745422	-3.23921514	-0.91586733
1	-1.90164566	3.65208483	-1.53316736	1	1.36525416	-2.64521527	0.52543265
6	1.82115424	1.97858489	-0.52376729	1	1.00135422	-1.95001507	-1.05826735
6	1.22365427	0.64598483	-0.14036733	1	-0.48854578	3.48598480	0.49703267
6	2.37395430	-0.09971517	0.54383266	1	-1.41484582	1.19378483	-0.88116729
6	3.41565418	0.98208481	0.75763267	6	-4.67474556	-2.51081514	-0.16556732
8	3.05445433	2.12298489	0.07273267	1	-2.84554577	-1.88051510	-1.09816742
8	1.36915421	2.83278489	-1.21526742	1	-4.08084583	-0.63791519	-1.06626737
8	4.41945457	0.94888484	1.39243257	1	-5.08514595	-2.93581510	-1.08456743
6	2.93985415	-1.33381522	-0.22046731	1	-5.50244570	-2.09851527	0.41543266
1	2.07345438	-0.44811517	1.53733265	1	-4.24274588	-3.33111525	0.41353264

TS__tBuMA--BU-rad_BU_transfer

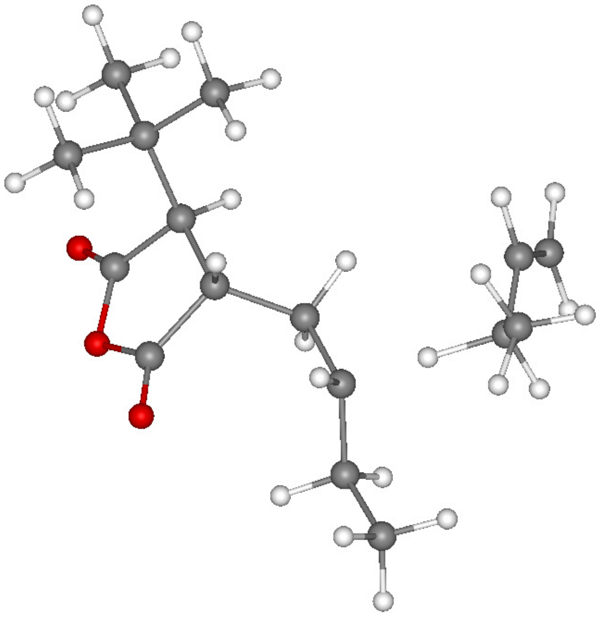

Zero-point vibrational energy	1048672.9 (Joules/Mol)
	250.63884 (Kcal/Mol)
Zero-point correction=	0.399418 (Hartree/Particle)
Thermal correction to Energy=	0.422218
Thermal correction to Enthalpy=	0.423162
Thermal correction to Gibbs Free Energy=	0.344719
Sum of electronic and zero-point Energies=	-851.151318
Sum of electronic and thermal Energies=	-851.128518
Sum of electronic and thermal Enthalpies=	-851.127574
Sum of electronic and thermal Free Energies=	-851.206017

cartesian

6	2.01179338	2.35370016	-0.64100224	1	-4.83780622	-2.26059985	0.20739777
6	1.46039343	1.13610005	0.05149779	1	-4.87810612	-0.90829998	-0.93020219
1	1.39549351	1.23909998	1.13659775	1	-3.78090668	-2.25810003	-1.20640218
6	0.27569348	0.44029999	-0.56740224	1	-4.40320635	-0.70489997	2.14599776
6	3.19619346	2.98029995	0.08039778	1	-3.07340646	0.44349998	2.10959768
1	2.29459333	2.08979988	-1.66790223	1	-4.47400618	0.67269999	1.05479777
1	4.03009367	2.27970004	0.17119779	1	-2.89850664	-2.68039989	1.65489781
1	2.91679335	3.29680014	1.08839774	1	-1.77310658	-2.62849998	0.29909778
6	-1.47990656	2.10940003	0.14219777	1	-1.51090646	-1.60349989	1.71479785
6	-1.07090652	0.65569997	0.15719779	1	1.21629357	3.10479999	-0.73650217
6	-2.23750639	-0.06600001	-0.52460223	6	3.92269349	-0.87690002	1.33099782
6	-3.00290656	1.06500006	-1.18490219	6	3.42889333	-0.74330002	-0.09220222

8	-2.55440664	2.27740002	-0.70260221	6	2.86339355	-1.94120002	-0.70840222
8	-0.99760652	3.03060007	0.71749783	6	2.91629338	-2.25129986	-2.01170230
8	-3.87230659	1.01819992	-1.99320221	1	2.48869348	0.14520000	-0.02910221
6	-3.14200664	-0.93349999	0.40169781	1	3.16049337	-1.32380009	1.97559786
1	-1.87970662	-0.71919996	-1.32650220	1	4.81149387	-1.51279998	1.38989782
6	-4.22350645	-1.62339997	-0.43380219	1	2.44919348	-3.14779997	-2.40240216
6	-2.27540660	-2.01519990	1.05229783	1	3.43689346	-1.62059999	-2.72570229
6	-3.80350661	-0.07660002	1.48349786	1	4.13959360	-0.23220003	-0.74620217
1	-0.94300652	0.36679998	1.20179784	1	2.33769345	-2.62189984	-0.03790221
1	0.44009352	-0.64490002	-0.58570224	1	3.56189346	3.86140013	-0.45060223
1	0.17359352	0.73389995	-1.61850214	1	4.18439388	0.09449999	1.75609779

tBuMA--BU-rad—BU

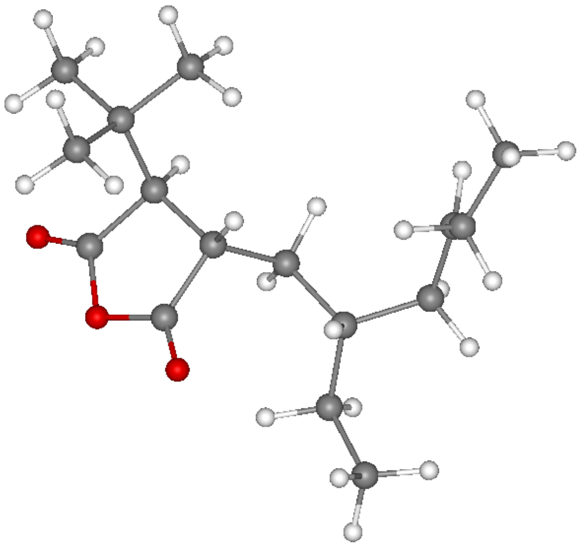

Zero-point vibrational energy	1069510.5 (Joules/Mol)
	255.61915 (Kcal/Mol)
Zero-point correction=	0.407355 (Hartree/Particle)
Thermal correction to Energy=	0.429767
Thermal correction to Enthalpy=	0.430711
Thermal correction to Gibbs Free Energy=	0.352875
Sum of electronic and zero-point Energies=	-851.196932
Sum of electronic and thermal Energies=	-851.174520
Sum of electronic and thermal Enthalpies=	-851.173575
Sum of electronic and thermal Free Energies=	-851.251412

cartesian

6	3.21488500	1.49860668	-0.59238482	6	-4.28641510	1.25410652	0.70511514
6	2.84798503	0.97790653	0.75341517	6	-2.13471508	2.30110645	0.05171516

6	2.77308488	-0.48289341	1.03421521	6	-3.32231498	0.78730661	-1.55288482
1	3.70788479	-0.95809340	0.70901519	1	-0.51561511	0.27820659	-0.82008481
1	2.58098507	1.69020653	1.52811515	1	0.39148489	0.23010659	1.41911519
1	2.70298505	-0.65749347	2.11391521	1	-0.10131514	-1.42889333	1.67431521
6	1.74498475	-2.74019361	0.48651516	1	-4.82351494	2.11740661	0.30441517
6	1.59988487	-1.21919346	0.34541517	1	-4.94851494	0.39040655	0.65561515
6	0.25618485	-0.72879344	0.90961516	1	-4.08021498	1.45230651	1.76041520
6	2.81328487	-3.35689354	-0.40498483	1	-3.84561515	1.64980650	-1.97168481
1	1.94858491	-2.97569346	1.53901517	1	-2.42861509	0.62850660	-2.16248488
1	3.80858517	-2.95179343	-0.20708483	1	-3.97581506	-0.07949343	-1.68148482
1	2.87048507	-4.43739367	-0.25638485	1	-2.69161510	3.16840649	-0.31088483
1	2.58398485	-3.18259358	-1.45888484	1	-1.86961520	2.49380660	1.09481514
6	-1.16051519	-1.72499347	-0.94528484	1	-1.20911515	2.24990654	-0.52598482
6	-0.85841513	-0.49139345	-0.12728484	1	0.78938484	-3.21849346	0.25381517
6	-2.20201516	-0.15969343	0.53071517	1	1.63458478	-0.98049343	-0.72378480
6	-2.96291494	-1.46669340	0.41411516	6	3.58128500	2.97760653	-0.59708482
8	-2.33981514	-2.28619337	-0.50588483	1	2.39278507	1.33210659	-1.30818486
8	-0.53891516	-2.21129346	-1.83388484	1	4.04808474	0.90680653	-0.99698484
8	-3.95211506	-1.81839347	0.96981514	1	3.83818483	3.32340646	-1.60038483
6	-2.99021506	1.03830647	-0.08048484	1	4.43718481	3.17290640	0.05311516
1	-2.07851505	0.04690656	1.59781516	1	2.74968505	3.59090662	-0.23968484

S8. MA – EB reaction profile

TS__tBuMA-rad_EB__re

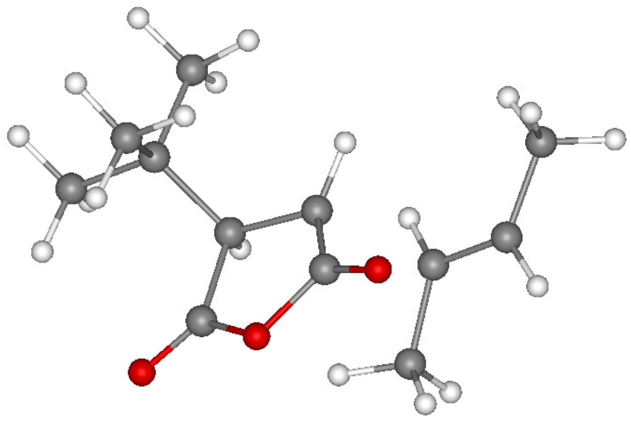

Zero-point vibrational energy	761323.2 (Joules/Mol)
	181.96062 (Kcal/Mol)
Zero-point correction=	0.289973 (Hartree/Particle)
Thermal correction to Energy=	0.306983
Thermal correction to Enthalpy=	0.307927
Thermal correction to Gibbs Free Energy=	0.244762
Sum of electronic and zero-point Energies=	-694.036487
Sum of electronic and thermal Energies=	-694.019477
Sum of electronic and thermal Enthalpies=	-694.018533
Sum of electronic and thermal Free Energies=	-694.081698

cartesian

6	3.22692060	0.05749413	0.09858528	1	-1.36757934	2.14969397	1.10208535
6	2.18582058	-0.23790586	0.93448526	1	-0.91757935	2.29819393	-0.60331470
6	0.72162062	-1.27760577	-1.33321476	1	-3.27257919	0.87039411	-1.86711478
6	0.44302061	-0.12570587	-0.50421470	1	-1.60537934	0.46349412	-2.26681471
6	-0.80447936	-0.38700587	0.27198529	1	-2.73237944	-0.80100584	-1.75931478
6	-1.01367939	-1.87650585	0.01898529	1	-4.09277964	0.73539412	0.52548528
8	-0.14107938	-2.31180573	-0.93871468	1	-3.50587940	-0.92400587	0.70088530
8	1.53192067	-1.45620584	-2.19341469	1	-2.98467922	0.32009417	1.83478522
8	-1.79287934	-2.61630583	0.53328526	6	3.54602051	1.41869414	-0.40311471
6	-2.04737949	0.47839415	-0.12551472	1	1.01102066	-1.80630589	1.87088513
1	-0.66907936	-0.25330585	1.35258532	1	2.64332056	-1.64120591	2.48058534
6	-3.22437954	0.12469417	0.78568530	1	2.37292051	-2.38710594	0.90598530
6	-1.69877934	1.95429409	0.07808529	1	2.94762039	2.19039392	0.08558530
6	-2.42947960	0.23479414	-1.58601475	1	3.37592077	1.48199415	-1.48421478
1	0.72182059	0.85029411	-0.87041473	1	4.60172033	1.66109407	-0.24671471
6	2.03762054	-1.58940589	1.57088518	1	1.71102059	0.59269416	1.45458531

1	-2.57977915	2.57449436	-0.10421471	1	3.80152082	-0.76520586	-0.31911471

TS__tBuMA-rad_EB__si

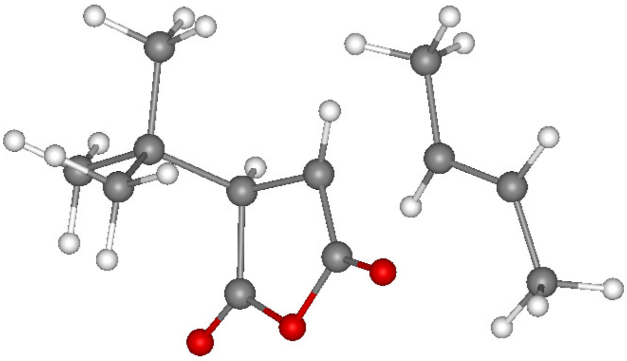

Zero-point vibrational energy	761476.7 (Joules/Mol)
	181.99730 (Kcal/Mol)
Zero-point correction=	0.290031 (Hartree/Particle)
Thermal correction to Energy=	0.306979
Thermal correction to Enthalpy=	0.307923
Thermal correction to Gibbs Free Energy=	0.245088
Sum of electronic and zero-point Energies=	-694.036010
Sum of electronic and thermal Energies=	-694.019062
Sum of electronic and thermal Enthalpies=	-694.018118
Sum of electronic and thermal Free Energies=	-694.080953

cartesian

6	3.90753222	-1.01763535	0.38535291	6	1.70883226	2.19826460	0.47645295
6	3.27193236	0.28166473	0.05015292	1	3.93113232	-1.67813528	-0.48644710
6	2.11703229	0.75836468	0.61345291	1	-3.19136763	1.67426479	-1.10234714
1	3.38203239	-1.53813529	1.18765295	1	-1.91746771	2.19096470	-0.00154708
6	0.89393228	-1.46513534	-0.85734707	1	-1.51136780	1.57576478	-1.61074710
6	0.42893225	-0.12663528	-0.56854707	1	-3.43826771	-0.77253532	-1.71594703
6	-0.70246774	-0.22893529	0.40275294	1	-1.72876775	-0.94223529	-2.10484719
6	-0.53216773	-1.65663528	0.90785289	1	-2.47936773	-2.00673532	-0.90854710
8	0.34833226	-2.32833529	0.10445292	1	-4.16656780	0.11706471	0.54015291
8	1.63623226	-1.88023531	-1.69724715	1	-3.17286777	-1.07253528	1.39105296
8	-1.05056775	-2.18363523	1.84145296	1	-2.97206759	0.64576471	1.72725296
6	-2.13266778	0.02706471	-0.18624708	1	4.94693232	-0.87083530	0.69865292
1	-0.59486771	0.44296473	1.26205289	1	0.63303226	2.34336472	0.58895290
6	-3.16726756	-0.08313528	0.93545294	1	2.00663233	2.60646486	-0.49144709
6	-2.18006778	1.44536471	-0.75734711	1	2.19433236	2.79956484	1.25075293
6	-2.45486760	-0.98233527	-1.28884709	1	3.73543239	0.86586475	-0.74254709
1	0.48753223	0.61436474	-1.35134709	1	1.76323223	0.24666470	1.50745296

TS__tBuMA-rad_to_EB_transfer

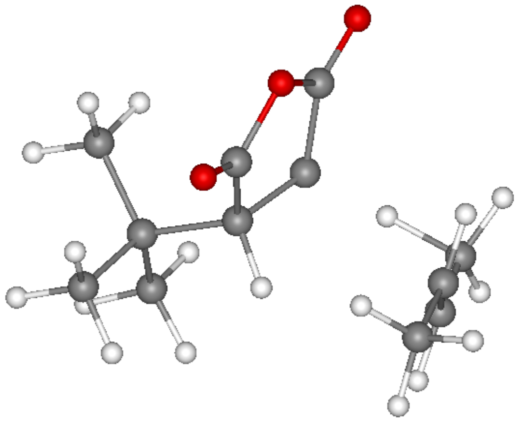

Zero-point vibrational energy	749468.2 (Joules/Mol)
	179.12721 (Kcal/Mol)
Zero-point correction=	0.285457 (Hartree/Particle)
Thermal correction to Energy=	0.302554
Thermal correction to Enthalpy=	0.303498
Thermal correction to Gibbs Free Energy=	0.238794
Sum of electronic and zero-point Energies=	-694.031152
Sum of electronic and thermal Energies=	-694.014055
Sum of electronic and thermal Enthalpies=	-694.013111
Sum of electronic and thermal Free Energies=	-694.077815

cartesian

6	-2.20285606	1.27969706	-1.38663244	6	-3.78785586	-1.44950294	0.84816754
6	-2.72185588	0.01649705	-0.90013242	1	-3.46185589	0.72539705	0.92836761
6	-3.31785607	-0.15040296	0.29696760	1	3.37844396	-0.98280299	-1.69723237
1	-0.97005594	1.38899708	-0.98633242	1	1.67454410	-1.09660292	-2.13473225
6	0.17444403	2.35589695	0.67526758	1	2.46084404	0.48299706	-2.00593233
6	0.34464404	1.45929706	-0.46503243	1	4.05314445	0.03549704	0.53786755
6	0.58754402	0.07999703	0.07126759	1	3.01944399	1.44309700	0.31086758
6	0.15674403	0.23979706	1.52356756	1	2.83814406	0.42859703	1.74836755
8	-0.01115596	1.56759703	1.81566751	1	3.10614395	-2.30440307	0.36176759
8	0.12774403	3.54549694	0.73896754	1	1.83064401	-1.94190288	1.53176761
8	-0.02705596	-0.60260296	2.34496760	1	1.41234398	-2.55990291	-0.06433243
6	2.04044414	-0.47430295	-0.07543242	1	-2.62525606	2.17609692	-0.93423247
1	-0.08655596	-0.65970296	-0.37893245	1	-2.09285593	1.37379706	-2.46513224
6	2.09704399	-1.90030289	0.47626758	1	-3.25015593	-1.69190288	1.77066755
6	2.40114403	-0.51310295	-1.56233239	1	-3.63935590	-2.26980305	0.14386758
6	3.03654408	0.41189706	0.67356759	1	-4.85005569	-1.40970290	1.10866761
1	0.87774402	1.85559702	-1.32043242	1	-2.57295585	-0.85930300	-1.53003240

tBuMA--EB-rad__re

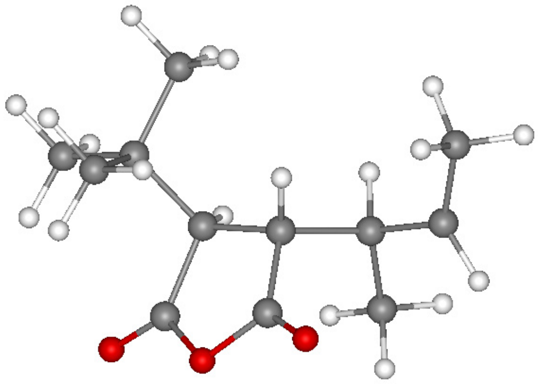

Zero-point vibrational energy	768142.8 (Joules/Mol)
	183.59053 (Kcal/Mol)
Zero-point correction=	0.292570 (Hartree/Particle)
Thermal correction to Energy=	0.309488
Thermal correction to Enthalpy=	0.310432
Thermal correction to Gibbs Free Energy=	0.247503
Sum of electronic and zero-point Energies=	-694.068036
Sum of electronic and thermal Energies=	-694.051118
Sum of electronic and thermal Enthalpies=	-694.050174
Sum of electronic and thermal Free Energies=	-694.113103

cartesian

6	-3.06035280	0.08485287	-0.16072060	1	-0.68005288	0.53245294	0.89797938
6	-3.29125285	1.37205291	0.54117942	1	-1.46745288	0.60885286	-1.44242060
6	-1.75635290	-0.24034710	-0.80502063	6	-1.85945284	-1.48954713	-1.67592061
1	-2.70975280	2.18935299	0.10317940	1	3.94374704	1.00775290	-0.23442058
1	-3.01815295	1.31455290	1.60557938	1	3.58544707	-0.71214712	-0.43012059
6	-0.75945288	-1.56474710	1.10227942	1	3.06564713	0.44095290	-1.65622056
6	-0.59925288	-0.33574709	0.24317941	1	2.83474708	1.05785286	2.03177953
6	0.79864717	-0.47814709	-0.36292058	1	1.19184709	0.47385287	2.25267935
6	1.15674710	-1.92324710	-0.07072060	1	2.49904704	-0.66344708	1.89987934
8	0.25734714	-2.45514727	0.82827938	1	2.17724705	2.65845299	0.17937940
8	-1.60905290	-1.82164705	1.89207935	1	1.20274711	2.09575272	-1.17702067
8	2.05354714	-2.57914686	-0.49262059	1	0.49874714	2.20995283	0.44037938
6	1.87634718	0.51625288	0.16807941	1	-3.76675296	-0.72314709	-0.00302060
1	0.77444714	-0.36634710	-1.45102060	1	-0.91965288	-1.73604715	-2.17522073
6	3.19304705	0.29265290	-0.58032060	1	-2.61205292	-1.34404707	-2.45252061
6	1.40284717	1.94505298	-0.11252059	1	-2.16375279	-2.35894704	-1.08692062
6	2.10544705	0.32985288	1.66937935	1	-4.34485292	1.66135299	0.51437938

tBuMA--EB-rad__si

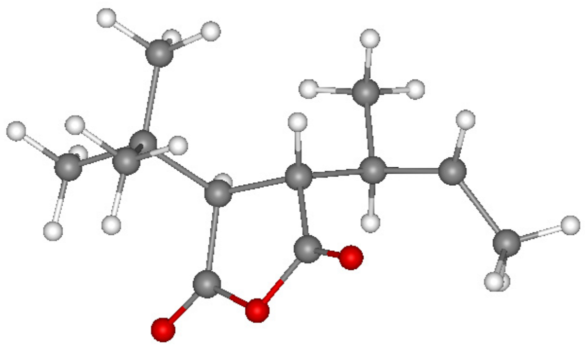

Zero-point vibrational energy	768953.3 (Joules/Mol)
	183.78425 (Kcal/Mol)
Zero-point correction=	0.292879 (Hartree/Particle)
Thermal correction to Energy=	0.309646
Thermal correction to Enthalpy=	0.310590
Thermal correction to Gibbs Free Energy=	0.248531
Sum of electronic and zero-point Energies=	-694.066200
Sum of electronic and thermal Energies=	-694.049433
Sum of electronic and thermal Enthalpies=	-694.048489
Sum of electronic and thermal Free Energies=	-694.110548

cartesian

6	3.07096767	0.30664995	-0.12991762	1	0.47406760	0.38434997	-1.20721769
6	4.10196733	-0.67585003	0.28688237	6	1.44816756	1.93394995	0.79868233
6	1.74506760	0.45344999	0.54068232	1	-4.15803242	0.21724996	0.14848237
1	3.99846745	-1.62725008	-0.25291762	1	-3.37293243	-1.11225009	1.00698233
1	4.03956747	-0.90535003	1.35458231	1	-3.14803243	0.54504991	1.55798233
6	0.89696759	-1.61105001	-0.64411765	1	-3.12843251	-0.50545001	-2.03621745
6	0.57486761	-0.18065003	-0.27921763	1	-1.39293242	-0.72585005	-2.19801760
6	-0.73443240	-0.30315003	0.50928235	1	-2.34653234	-1.85915005	-1.23231769
6	-0.73463237	-1.76115012	0.92748237	1	-2.88303232	1.84204984	-1.17791760
8	0.17326760	-2.46245003	0.16378236	1	-1.82083249	2.20274997	0.18078238
8	1.65266752	-2.02944994	-1.45911765	1	-1.14033246	1.73924994	-1.38191772
8	-1.38533247	-2.30815005	1.75768232	1	5.11166763	-0.31045002	0.08118236
6	-2.03923249	0.07704994	-0.25891763	1	0.48926759	2.09264994	1.29658234
1	-0.71543241	0.29094997	1.42658234	1	1.43296754	2.49175000	-0.14141762
6	-3.24593234	-0.08445004	0.66938233	1	2.22556758	2.36934996	1.42808235
6	-1.95563245	1.54565001	-0.68201768	1	3.21216750	0.84274995	-1.06451762
6	-2.22753239	-0.80495000	-1.49591768	1	1.78156757	-0.06785002	1.50658238

TS__tBuMA--EB-rad_MA__R_re

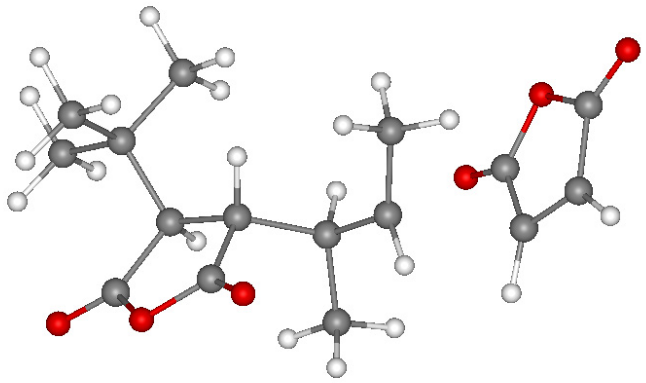

Zero-point vibrational energy	922037.2 (Joules/Mol)
	220.37218 (Kcal/Mol)
Zero-point correction=	0.351185 (Hartree/Particle)
Thermal correction to Energy=	0.373627
Thermal correction to Enthalpy=	0.374572
Thermal correction to Gibbs Free Energy=	0.297336
Sum of electronic and zero-point Energies=	-1073.299868
Sum of electronic and thermal Energies=	-1073.277426
Sum of electronic and thermal Enthalpies=	-1073.276482
Sum of electronic and thermal Free Energies=	-1073.353717

cartesian

6	-3.46581626	0.44663954	-1.16530931	6	2.53818369	1.35083961	0.24659070
6	-3.66981626	-1.00306046	-0.93630934	1	1.69548380	0.37633950	-1.46900928
6	-4.71051598	-1.12926042	-0.07340932	6	3.60018373	2.00393963	-0.64240932
6	-5.09081602	0.20823954	0.38169068	6	1.38868380	2.34533954	0.43079069
8	-4.28581619	1.12683952	-0.30000931	6	3.15318370	1.00713956	1.60509074
8	-2.74161625	1.00633955	-1.93170929	1	0.61148381	-0.15286046	1.21449077
8	-5.91351604	0.56373954	1.16519070	1	-0.72921622	0.35033953	-0.81300932
1	-3.27031636	-1.75026047	-1.60320926	6	-0.36941624	-1.49656045	-1.83950925
1	-5.20671606	-2.02246046	0.27419069	1	3.97058368	2.91643953	-0.16880932
1	-1.78471625	-2.27246046	0.31109068	1	4.44798374	1.34423959	-0.82090932
6	-1.61931622	-1.19946051	0.32149071	1	3.18318367	2.28243947	-1.61420929
6	-1.96061623	-0.50546044	1.58929074	1	3.51078367	1.91513968	2.09529066
6	-0.51091623	-0.69176048	-0.55070931	1	2.43548369	0.54033953	2.28539062
1	-1.28081620	-0.82736051	2.38889050	1	4.00978374	0.33593953	1.50389075
1	-2.96451640	-0.76316047	1.93359077	1	1.76728380	3.27573967	0.86019069
6	1.34278381	-2.01106048	0.56299067	1	0.91628385	2.59313965	-0.52340931
6	0.82298374	-0.62726045	0.25489068	1	0.61168373	1.97653961	1.10439074
6	1.98218381	0.07603954	-0.45630932	1	-1.89191616	0.58203954	1.50749075
6	3.02288365	-1.01816046	-0.60700935	1	0.51008379	-1.20156050	-2.41530943

8	2.59888363	-2.16646051	0.03869069	1	-1.22821629	-1.32846045	-2.48850942
8	0.79808378	-2.89026046	1.15139067	1	-0.29071623	-2.56806040	-1.63770926
8	4.06718397	-0.99836046	-1.17020929				

TS__tBuMA--EB-rad_MA__R_si

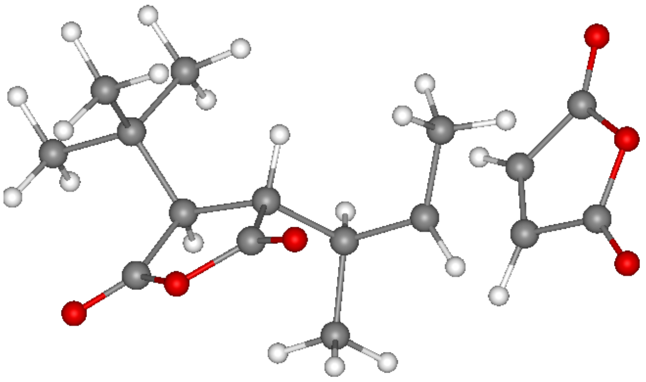

Zero-point vibrational energy	921612.8 (Joules/Mol)
	220.27074 (Kcal/Mol)
Zero-point correction=	0.351024 (Hartree/Particle)
Thermal correction to Energy=	0.373497
Thermal correction to Enthalpy=	0.374441
Thermal correction to Gibbs Free Energy=	0.296977
Sum of electronic and zero-point Energies=	-1073.296486
Sum of electronic and thermal Energies=	-1073.274013
Sum of electronic and thermal Enthalpies=	-1073.273069
Sum of electronic and thermal Free Energies=	-1073.350532

cartesian

6	4.69051170	1.15307450	-0.00604188	6	-3.14818859	0.96147436	-1.55994189
6	3.70191145	0.83907443	1.02865815	1	-0.44928843	0.06207439	-1.17184186
6	3.56461143	-0.50822562	1.08595812	1	0.76501155	0.16327441	0.96915817
6	4.58121157	-1.09412563	0.17555812	6	0.31531158	-1.83772564	1.63415813
8	5.18771172	-0.05622560	-0.49364185	1	-4.18038845	2.60567427	0.35745811
8	5.07921171	2.20027423	-0.41734189	1	-4.50238848	0.94327444	0.86225814
8	4.86971140	-2.23352551	-0.00994188	1	-3.35008860	1.92937422	1.75935817
1	3.20581150	1.60637450	1.60345817	1	-3.57678843	1.87407422	-1.97974181
1	3.09491158	-1.10132563	1.85395813	1	-2.38018847	0.61867440	-2.25874209
6	1.74341154	-1.19692564	-0.32524186	1	-3.94348860	0.21187437	-1.53924191
6	0.56071156	-0.82072562	0.52165812	1	-2.01108837	3.28047442	-0.56974185
6	-1.21298838	-1.88822556	-0.92344189	1	-1.10998845	2.55097437	0.75615811
6	-0.71848840	-0.58582556	-0.33804190	1	-0.72788841	2.13537431	-0.91984189
6	-1.91908836	-0.03812560	0.43675810	1	2.07651162	-2.22892570	-0.27104187
6	-2.87188840	-1.21852565	0.47995812	6	2.04311156	-0.46702561	-1.58324182

8	-2.43628860	-2.20362568	-0.38704187	1	3.03171158	-0.70892560	-1.97344184
8	-0.67178839	-2.60002565	-1.70684183	1	1.97441149	0.61877441	-1.46364188
8	-3.86328840	-1.36742568	1.11505818	1	1.32931161	-0.76262558	-2.36254191
6	-2.59018850	1.23737431	-0.16204187	1	-0.52848840	-1.56472564	2.27045798
1	-1.65598845	0.20127439	1.47115815	1	1.18351150	-1.93032551	2.28785801
6	-3.72368860	1.69747424	0.75815815	1	0.11801156	-2.83092570	1.22315812
6	-1.54468846	2.35377431	-0.22774188				

TS__tBuMA--EB-rad_MA__S_re

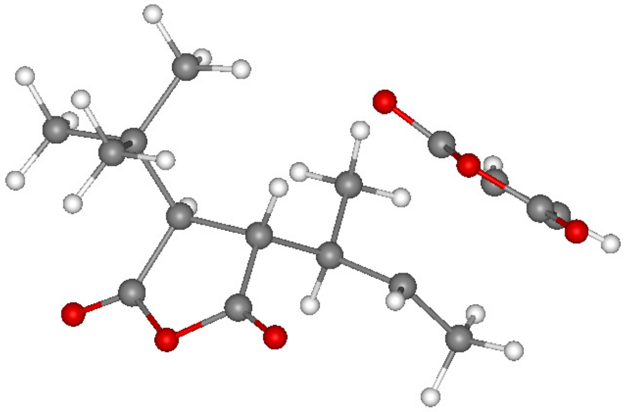

Zero-point vibrational energy	923235.9 (Joules/Mol)
	220.65867 (Kcal/Mol)
Zero-point correction=	0.351642 (Hartree/Particle)
Thermal correction to Energy=	0.373894
Thermal correction to Enthalpy=	0.374838
Thermal correction to Gibbs Free Energy=	0.298672
Sum of electronic and zero-point Energies=	-1073.294791
Sum of electronic and thermal Energies=	-1073.272539
Sum of electronic and thermal Enthalpies=	-1073.271595
Sum of electronic and thermal Free Energies=	-1073.347761

cartesian

6	2.76406741	-1.20456755	0.28046510	6	-4.06093264	-1.20336747	-0.13643491
6	3.33406734	-0.22196746	-0.67583489	6	-1.82663250	-2.22586751	-0.49013489
6	4.53136730	0.17083253	-0.16653490	6	-2.50643253	-1.48086751	1.80116510
6	4.67186737	-0.40436745	1.17086506	1	-0.02553263	-0.51516747	0.63596511
8	3.55386734	-1.22106755	1.39346504	6	0.36576736	0.31533253	-2.01373482
8	1.79696727	-1.89896750	0.17096509	1	-0.14353263	2.08553243	-0.94543493
8	5.52786732	-0.28876746	1.98746514	1	-4.47503281	-2.19896746	0.04056510
1	3.00516748	-0.17036745	-1.70143497	1	-4.67563295	-0.48176748	0.40046510
1	5.28906727	0.80143255	-0.60533488	1	-4.15743303	-0.99076742	-1.20473492
6	2.42766738	2.61433244	-0.89333487	1	-2.91553259	-2.47416759	1.99856508

6	1.72866726	1.48213243	-0.22583491	1	-1.47623253	-1.48406756	2.16646504
1	1.91066742	1.37763250	0.83796513	1	-3.07973266	-0.77136743	2.40396500
6	0.34476736	1.12383246	-0.71063495	1	-2.27693248	-3.20776749	-0.32593489
6	-0.58663261	1.27473247	1.62626505	1	-1.87143254	-2.02316761	-1.56383491
6	-0.51443261	0.42273253	0.37446511	1	-0.77693266	-2.30026746	-0.20273489
6	-1.98893261	0.23733252	-0.00883490	1	3.46546745	2.70163250	-0.57083488
6	-2.68593264	1.33573246	0.76736510	1	2.40526748	2.54713249	-1.98453486
8	-1.83973265	1.81953251	1.74686515	1	1.93286729	3.55813241	-0.62563491
8	0.26606739	1.51413250	2.42106533	1	-0.62153262	0.26693255	-2.47343493
8	-3.78103280	1.77643251	0.64096510	1	0.70246738	-0.70506746	-1.83183491
6	-2.59573245	-1.16816747	0.30596510	1	1.02846742	0.78013253	-2.74693489
1	-2.17053246	0.43523255	-1.06733489				

TS__tBuMA--EB-rad_MA__S_si

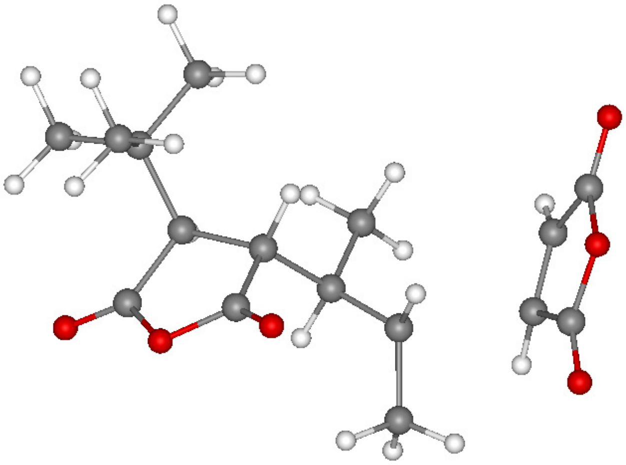

Zero-point vibrational energy	924032.5 (Joules/Mol)
	220.84906 (Kcal/Mol)
Zero-point correction=	0.351945 (Hartree/Particle)
Thermal correction to Energy=	0.374045
Thermal correction to Enthalpy=	0.374989
Thermal correction to Gibbs Free Energy=	0.298901
Sum of electronic and zero-point Energies=	-1073.288165
Sum of electronic and thermal Energies=	-1073.266065
Sum of electronic and thermal Enthalpies=	-1073.265121
Sum of electronic and thermal Free Energies=	-1073.341209

cartesian

6	4.47112131	1.31889772	-0.46359533	6	-2.03267908	2.35069752	-0.42899534
6	3.95272088	0.91399771	0.83950472	6	-3.07687902	0.60819769	-1.89079535
6	3.82422090	-0.44570231	0.85220468	1	-0.24577898	0.35429770	-1.04479527

6	4.49942112	-0.93380231	-0.38389534	6	0.84882104	0.82239765	1.35970461
8	4.77002096	0.15659767	-1.17479539	1	0.15152103	-1.18530226	1.55290461
8	4.64952087	2.40089774	-0.92939532	1	-4.70177889	2.01429772	-0.19989534
8	4.79442120	-2.04110241	-0.70719534	1	-4.73017883	0.32479769	0.31280467
1	3.71432090	1.63199759	1.60780466	1	-3.95787907	1.53959775	1.32760465
1	3.71982098	-1.08280230	1.71750462	1	-3.64807892	1.38769770	-2.39949512
6	1.74772096	-1.25350237	0.16130465	1	-2.16137910	0.45639771	-2.46919537
6	0.54772103	-0.54720235	0.75280464	1	-3.66697884	-0.31020230	-1.94649529
6	-0.84157902	-1.67680240	-1.05729532	1	-2.63597894	3.13049769	-0.89919531
6	-0.58277899	-0.38320231	-0.31459534	1	-1.81757903	2.67689753	0.59180468
6	-1.95057893	-0.05750231	0.29930466	1	-1.08797908	2.30589771	-0.97499532
6	-2.64217901	-1.40700221	0.29530466	6	1.95182109	-2.68610239	0.51740468
8	-1.99067903	-2.25720239	-0.57799530	1	2.90892100	-3.06590247	0.16290465
8	-0.19497895	-2.19070220	-1.91149533	1	1.17522097	-3.30140233	0.04520467
1	1.53982091	0.72899765	2.19700480	1	1.87622094	-2.84870243	1.59660470
8	-3.60007906	-1.76750231	0.89570463	1	1.97932100	-0.98190230	-0.86529535
6	-2.79687905	1.02479768	-0.44499534	1	-0.05807900	1.29399776	1.74020469
1	-1.86987901	0.25649768	1.34260464	1	1.29132104	1.49609780	0.62380469
6	-4.12317896	1.22909760	0.29240465				

tBuMA--EB-rad--MA__R_re

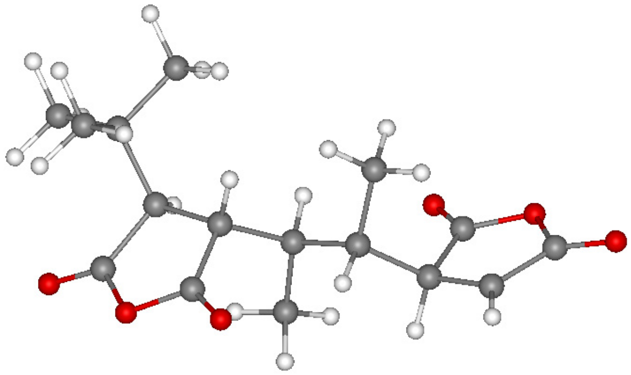

Zero-point vibrational energy 930973.1 (Joules/Mol)
222.50792 (Kcal/Mol)
Zero-point correction= 0.354589 (Hartree/Particle)
Thermal correction to Energy= 0.376705
Thermal correction to Enthalpy= 0.377649
Thermal correction to Gibbs Free Energy= 0.301634
Sum of electronic and zero-point Energies= -1073.339495
Sum of electronic and thermal Energies= -1073.317379
Sum of electronic and thermal Enthalpies= -1073.316435
Sum of electronic and thermal Free Energies= -1073.392450

cartesian

6	3.65713477	0.48510000	1.07975113	6	-2.74296522	1.06190002	-0.24604887
6	3.19243479	-0.82149994	0.44755113	1	-1.63326514	0.44350001	1.48485112
6	4.37113476	-1.20400000	-0.35444888	6	-3.88556528	1.56449997	0.64045107
6	5.41443491	-0.22640000	-0.18814889	6	-1.83096516	2.24869990	-0.56804889
8	4.93243456	0.76580000	0.67965108	6	-3.32026529	0.48139998	-1.53894889
8	3.05273485	1.20160007	1.81395113	1	-0.57426512	-0.09010001	-1.18554890
8	6.51483488	-0.16450000	-0.64874887	1	0.71283484	0.65989995	0.85695112
1	3.04493475	-1.54699993	1.25775111	6	0.63983488	-1.22650003	1.85345113
1	4.48183489	-2.07999992	-0.97634888	1	-4.44816542	2.33999991	0.11475112
1	1.69763494	-1.68949997	-0.78634888	1	-4.57696533	0.76849997	0.91265112
6	1.86203492	-0.70220000	-0.34324887	1	-3.50326514	2.00379992	1.56585109
6	2.01843476	0.31770000	-1.47034895	1	-3.86756516	1.25290000	-2.08504891
6	0.65323484	-0.39520001	0.57145113	1	-2.54566526	0.10370001	-2.21214890
1	1.16693485	0.31230000	-2.15114880	1	-4.02176523	-0.33320001	-1.34134889
1	2.89783478	0.09930000	-2.08074880	1	-2.40816522	3.04059982	-1.05124891
6	-1.00936508	-2.00889993	-0.45254889	1	-1.39086509	2.67079997	0.33935112
6	-0.67986512	-0.55320001	-0.20474887	1	-1.01536512	1.98820007	-1.24654889
6	-1.91046512	-0.00670001	0.52695113	1	2.12663484	1.33370006	-1.08044887
6	-2.71726513	-1.25090003	0.84365112	1	-0.29376516	-1.10189998	2.40605116
8	-2.17066526	-2.34290004	0.19405112	1	1.43303490	-0.91250002	2.53155112
8	-0.40016517	-2.81420016	-1.08344889	1	0.75663483	-2.29509997	1.64915109
8	-3.68036509	-1.37749994	1.52585113				

tBuMA--EB-rad--MA__R_si

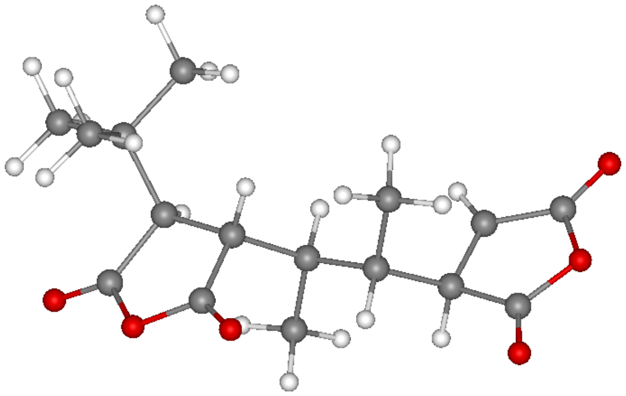

Zero-point vibrational energy	930735.3 (Joules/Mol)
	222.45108 (Kcal/Mol)
Zero-point correction=	0.354498 (Hartree/Particle)
Thermal correction to Energy=	0.376657
Thermal correction to Enthalpy=	0.377601
Thermal correction to Gibbs Free Energy=	0.301390

Sum of electronic and zero-point Energies= -1073.337104
Sum of electronic and thermal Energies= -1073.314945
Sum of electronic and thermal Enthalpies= -1073.314001
Sum of electronic and thermal Free Energies= -1073.390212

cartesian

6	4.96655130	0.94631857	0.40155116	6	-3.25644898	0.42191857	-1.60654891
6	3.63105106	0.72091860	0.89545119	1	-0.47614896	-0.04158142	-1.17594886
6	3.21605110	-0.66328144	0.59555119	1	0.77355111	0.60901856	0.85125118
6	4.43865108	-1.20508146	-0.13724884	6	0.56985104	-1.25738144	1.89675117
8	5.40475130	-0.23578142	-0.20314884	1	-4.46484900	2.26601863	0.00675115
8	5.66035128	1.91841865	0.44635114	1	-4.56774855	0.69981855	0.81745118
8	4.58895111	-2.28668141	-0.60224885	1	-3.54034877	1.96841860	1.47935116
1	3.07695103	1.48471856	1.42045116	1	-3.81734920	1.17191863	-2.16834879
1	3.12895107	-1.25428140	1.51655114	1	-2.45984888	0.06181857	-2.26344895
6	1.91825104	-0.85668141	-0.23024887	1	-3.93684888	-0.41178143	-1.41454887
6	0.67835104	-0.45558143	0.59835118	1	-2.42374897	3.00901866	-1.13194883
6	-0.98694891	-1.98368144	-0.53914881	1	-1.42764902	2.68501854	0.28425115
6	-0.63214892	-0.54698145	-0.22444886	1	-0.99854892	1.99501872	-1.28664887
6	-1.87304902	-0.00558141	0.49265113	1	1.86185098	-1.92838144	-0.44034886
6	-2.66264892	-1.25958145	0.81635118	6	2.02325106	-0.11678141	-1.56234884
8	-2.13764906	-2.33348131	0.12395115	1	2.97785091	-0.31718141	-2.05314898
8	-0.40424895	-2.76088142	-1.22444892	1	1.93595099	0.96701854	-1.43844891
8	-3.60034919	-1.39958143	1.53095114	1	1.25535107	-0.44918144	-2.26054883
6	-2.71954894	1.03101850	-0.30934885	1	-0.36134893	-1.05668139	2.43035102
1	-1.61574888	0.46781856	1.44435108	1	1.37055111	-1.01558137	2.59665108
6	-3.89184880	1.51101851	0.55055118	1	0.61295104	-2.33298135	1.70435107
6	-1.83494902	2.23941875	-0.62764883				

tBuMA--EB-rad--MA__S_re

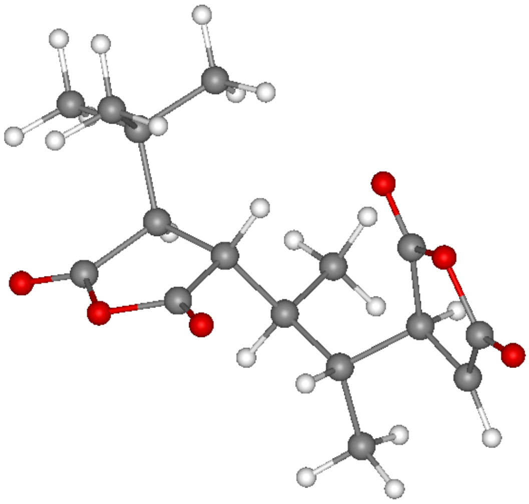

Zero-point vibrational energy	932343.8 (Joules/Mol)
	222.83553 (Kcal/Mol)
Zero-point correction=	0.355111 (Hartree/Particle)
Thermal correction to Energy=	0.377096
Thermal correction to Enthalpy=	0.378040
Thermal correction to Gibbs Free Energy=	0.302563
Sum of electronic and zero-point Energies=	-1073.334610
Sum of electronic and thermal Energies=	-1073.312625
Sum of electronic and thermal Enthalpies=	-1073.311681
Sum of electronic and thermal Free Energies=	-1073.387158

cartesian

6	2.69191384	-1.18541861	0.25059533	6	-4.13398647	-0.88351864	-0.16870463
6	2.97401381	0.16078140	-0.41830468	6	-2.05438614	-2.11011839	-0.74760461
6	4.31631374	0.46858138	0.12179536	6	-2.57608604	-1.60771859	1.64679539
6	4.71571350	-0.54131860	1.06439543	1	-0.01958612	-0.79441863	0.47569534
8	3.69781399	-1.50451863	1.10729527	6	0.43071389	0.30458140	-2.00520468
8	1.77381396	-1.92431867	0.06799534	1	0.01311389	1.97708142	-0.75310469
8	5.70221376	-0.65031862	1.72799540	1	-4.66748619	-1.83441865	-0.09560466
1	3.02171397	-0.02631860	-1.50020468	1	-4.63488626	-0.16461860	0.47869533
1	4.95111370	1.30618143	-0.12060463	1	-4.23248625	-0.52741861	-1.19790471
6	2.42401385	2.56908154	-0.87590468	1	-3.09058619	-2.56781840	1.72559536
6	1.93121397	1.29768133	-0.18090469	1	-1.54428613	-1.77271867	1.96769536
1	1.92211390	1.49188137	0.89229536	1	-3.04998612	-0.92941862	2.36139536
6	0.47971389	0.98938137	-0.64060462	1	-2.61648607	-3.04541850	-0.69120467
6	-0.33048609	0.85548139	1.75809538	1	-2.10168600	-1.76341867	-1.78330469
6	-0.39848611	0.22088140	0.38759536	1	-1.01388609	-2.34451842	-0.51620466
6	-1.89728618	0.26848140	0.05839536	1	3.39571381	2.89208150	-0.50070465
6	-2.42338610	1.31678140	1.01709533	1	2.51281381	2.44008160	-1.95800471
8	-1.49608612	1.53168142	2.01939535	1	1.72971392	3.39198160	-0.69680464

8	0.56241387	0.84308136	2.54599524	1	-0.57998610	0.29148138	-2.41420460
8	-3.45368600	1.90698135	1.00729537	1	0.77121389	-0.72951859	-1.94250464
6	-2.66478610	-1.08441865	0.21109535	1	1.05151391	0.83168137	-2.73330474
1	-2.08888602	0.63578141	-0.95230460				

tBuMA--EB-rad--MA__S_si

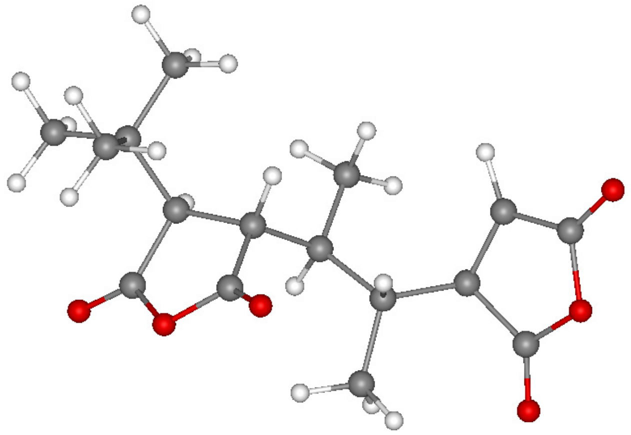

Zero-point vibrational energy	932720.8 (Joules/Mol)
	222.92562 (Kcal/Mol)
Zero-point correction=	0.355255 (Hartree/Particle)
Thermal correction to Energy=	0.377053
Thermal correction to Enthalpy=	0.377998
Thermal correction to Gibbs Free Energy=	0.303510
Sum of electronic and zero-point Energies=	-1073.324873
Sum of electronic and thermal Energies=	-1073.303074
Sum of electronic and thermal Enthalpies=	-1073.302130
Sum of electronic and thermal Free Energies=	-1073.376617

cartesian

6	4.89871168	1.00538146	-0.67872792	6	-3.18928862	0.52778137	-1.71002793
6	3.66861153	1.07228136	0.06787211	1	-0.30838853	0.72148144	-0.95552790
6	3.31981158	-0.26571861	0.59447211	6	0.83141148	1.08338141	1.34657204
6	4.57361126	-1.06151867	0.21477212	1	0.40701151	-0.99261856	1.43757212
8	5.39081144	-0.29491863	-0.57362789	1	-4.96588850	1.31628144	0.21347211
8	5.47851133	1.84788144	-1.29762793	1	-4.63738871	-0.40071863	0.46067208
8	4.87481165	-2.16271853	0.53987211	1	-4.06438828	0.78198147	1.63277209
1	3.12271142	1.99528134	0.17217211	1	-3.92558861	1.24508142	-2.07902789
1	3.26011157	-0.28981861	1.68957210	1	-2.29278851	0.64728141	-2.32432795
6	1.99791145	-0.86751866	0.01577210	1	-3.59318829	-0.47071862	-1.89482796
6	0.70881146	-0.27651861	0.66237211	1	-3.19428849	2.91368127	-0.35402790
6	-0.55318856	-1.33421862	-1.32552791	1	-2.22608852	2.41948128	1.03097212

6	-0.46798849	-0.17551862	-0.35452789	1	-1.52198863	2.43268132	-0.59142786
6	-1.84258842	-0.21231861	0.32957208	6	1.97231150	-2.38981867	0.17667212
6	-2.26058841	-1.65711856	0.13127212	1	2.69781160	-2.88891864	-0.46162790
8	-1.53098845	-2.20651865	-0.90702790	1	0.99151146	-2.79321861	-0.07662789
8	0.07541150	-1.53071856	-2.31432796	1	2.18791151	-2.67651868	1.20857215
8	-3.08148861	-2.29341865	0.70477211	1	1.99031150	-0.63401866	-1.05282784
6	-2.91968846	0.77458143	-0.22382790	1	-0.09548849	1.33458138	1.86437201
1	-1.76888847	-0.04111862	1.40617204	1	1.01961148	1.88698137	0.63187212
6	-4.21978855	0.60008132	0.56597209	1	1.61961150	1.10028136	2.10007215
6	-2.42738843	2.20978141	-0.02272789				

TS__tBuMA--EB-rad_EB__RRR

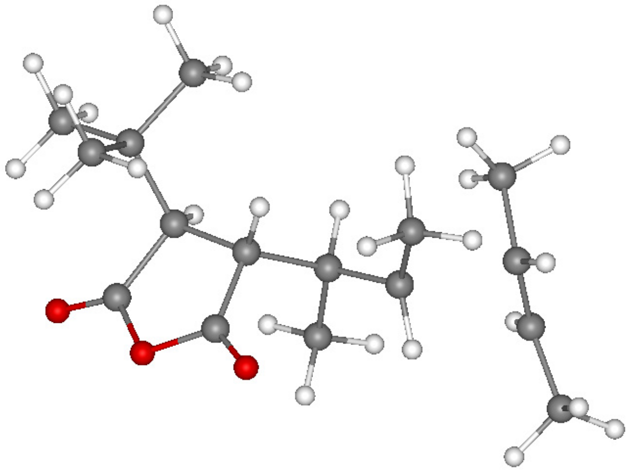

Zero-point vibrational energy	1058850.0 (Joules/Mol)
	253.07123 (Kcal/Mol)
Zero-point correction=	0.403295 (Hartree/Particle)
Thermal correction to Energy=	0.425846
Thermal correction to Enthalpy=	0.426790
Thermal correction to Gibbs Free Energy=	0.350518
Sum of electronic and zero-point Energies=	-851.156610
Sum of electronic and thermal Energies=	-851.134059
Sum of electronic and thermal Enthalpies=	-851.133114
Sum of electronic and thermal Free Energies=	-851.209386

cartesian

1	3.85671520	0.51743913	-0.95996302	1	-4.68878460	0.03133917	1.35573697
6	3.48991537	0.55293918	0.06313695	1	-3.69968462	1.30953908	2.05713701
6	4.05841541	-0.31926084	0.94973695	1	-4.50758457	1.03823912	-1.59076309
6	2.94481540	1.87953913	0.51123697	1	-3.07818460	0.13433918	-2.06886315
1	2.10461521	-1.41736090	-0.92376304	1	-4.33728456	-0.64226079	-1.10046303
6	1.65211535	-0.43366084	-0.82936305	1	-3.15498471	2.86243916	-0.47686306

6	0.54661524	-0.35146081	0.18813694	1	-1.90118480	2.45693922	0.69363695
6	-1.08538473	-1.93006086	-0.93486303	1	-1.68278480	2.06993937	-1.01716304
6	-0.87008476	-0.51876080	-0.44986305	6	1.50631523	0.29803917	-2.12566304
6	-2.03418469	-0.26776081	0.51183695	1	2.45331526	0.35073915	-2.66806293
6	-2.61938477	-1.65166092	0.72173697	1	1.14431524	1.32313919	-1.99436307
8	-2.07248473	-2.53346086	-0.18616304	1	0.80171531	-0.21696083	-2.79146314
8	-0.52288473	-2.51896071	-1.80116308	1	-0.00028473	-1.26196086	2.10723686
8	-3.42998457	-2.01846075	1.50973701	1	1.72971535	-1.15436089	1.80603695
6	-3.10428476	0.76133919	0.03443695	1	0.77341527	-2.36796069	0.96773696
1	-1.67688465	0.07923916	1.48603690	1	2.52351522	1.82023919	1.51773691
6	-4.15688467	0.95423913	1.12943697	1	2.17261529	2.26443934	-0.15766305
6	-2.41388464	2.10643935	-0.20636305	1	3.74141550	2.62953925	0.53853697
6	-3.78728461	0.29033914	-1.25136304	1	3.88071537	-0.17106083	2.01383686
1	-0.91988474	0.13243917	-1.32246304	6	4.83181524	-1.53166091	0.56063694
1	0.52771527	0.66763914	0.60023695	1	4.31331539	-2.45556068	0.84503698
6	0.76601523	-1.33676088	1.33223701	1	5.80651522	-1.56286085	1.05863690
1	-4.88848448	1.70223916	0.81333697	1	5.00711536	-1.57046092	-0.51656306

TS__tBuMA--EB-rad_EB__RRS

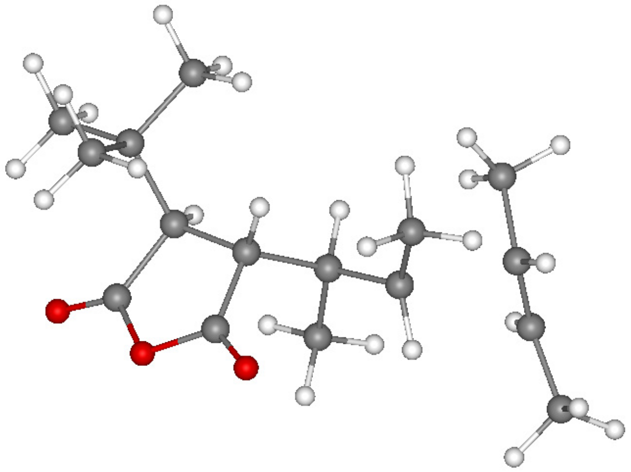

Zero-point vibrational energy	1058831.9 (Joules/Mol)
	253.06689 (Kcal/Mol)
Zero-point correction=	0.403288 (Hartree/Particle)
Thermal correction to Energy=	0.425842
Thermal correction to Enthalpy=	0.426786
Thermal correction to Gibbs Free Energy=	0.350537
Sum of electronic and zero-point Energies=	-851.156879
Sum of electronic and thermal Energies=	-851.134324
Sum of electronic and thermal Enthalpies=	-851.133380
Sum of electronic and thermal Free Energies=	-851.209629

cartesian

6	3.08947611	1.85082376	0.62427390	1	-4.86732388	0.12412381	1.06887388
6	3.40777612	0.38342381	0.66667390	1	-3.93942404	1.42192388	1.81717384
6	4.49497604	-0.13107619	0.01967391	1	-4.37922382	1.05752385	-1.88742614
1	3.06107593	-0.14957617	1.54817390	1	-2.93642402	0.09922382	-2.18692613
1	2.06217599	-1.49777615	-0.53022605	1	-4.31082392	-0.60887617	-1.32912612
6	1.63867617	-0.49937618	-0.46262610	1	-3.12702394	2.88962364	-0.67222607
6	0.43777609	-0.39697617	0.43937388	1	-1.98462391	2.48842382	0.60847390
6	-1.11302400	-1.93257618	-0.84222609	1	-1.62872386	2.06242371	-1.06982613
6	-0.90862393	-0.52337617	-0.34562612	6	1.66487598	0.26732385	-1.74352610
6	-2.15922403	-0.23297617	0.48847386	1	2.67727613	0.30822384	-2.15092611
6	-2.78652406	-1.60317612	0.66327393	1	1.31207609	1.29672384	-1.62412620
8	-2.17922401	-2.50627613	-0.18302609	1	1.03917599	-0.21157618	-2.50762606
8	-0.48772392	-2.54187632	-1.64902616	1	-0.35762390	-1.32717609	2.26007390
8	-3.67002392	-1.94437623	1.38147390	1	1.39117599	-1.23447609	2.20197392
6	-3.15802407	0.79932380	-0.11852609	1	0.55267608	-2.42427635	1.21787381
1	-1.89672399	0.13082385	1.48637390	1	2.02377605	2.05652380	0.74667394
6	-4.30892372	1.03622377	0.86297393	1	3.41207600	2.30212402	-0.31632611
6	-2.42502403	2.12762403	-0.32522610	1	3.60727596	2.37652397	1.43267381
6	-3.72022390	0.30272382	-1.45232618	6	5.00287580	-1.51987612	0.19997391
1	-0.84412390	0.12252384	-1.22102618	1	4.97527599	0.47212380	-0.74902606
1	0.39917606	0.61902380	0.85947394	1	4.86377621	-2.12467623	-0.70452607
6	0.50327611	-1.39777613	1.59107387	1	4.49657583	-2.03217602	1.02097392
1	-5.00162411	1.77572381	0.45317391	1	6.07697582	-1.53077614	0.41227388

TS__tBuMA--EB-rad_EB__SSR

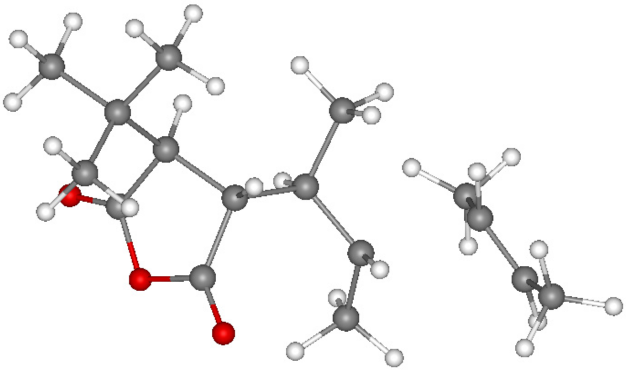

Zero-point vibrational energy	1059637.5 (Joules/Mol)
	253.25943 (Kcal/Mol)
Zero-point correction=	0.403595 (Hartree/Particle)
Thermal correction to Energy=	0.425989
Thermal correction to Enthalpy=	0.426933

Thermal correction to Gibbs Free Energy= 0.351819
Sum of electronic and zero-point Energies= -851.150895
Sum of electronic and thermal Energies= -851.128501
Sum of electronic and thermal Enthalpies= -851.127557
Sum of electronic and thermal Free Energies= -851.202671

cartesian

1	3.12908459	1.30883050	0.12418260	1	-5.38211536	1.18223047	0.18168260
6	3.56008458	0.38983044	0.51088262	1	-4.86351490	-0.24456958	1.08418262
6	4.52458477	-0.20076959	-0.25861740	1	-4.26491499	1.34823048	1.53768265
6	3.54868460	0.22973040	2.00598264	1	-4.62941504	0.17893043	-1.99671745
6	1.94428480	-2.05556965	0.42968261	1	-2.99411535	-0.42876959	-2.20811725
6	1.66048479	-0.67476958	-0.07301740	1	-4.13191509	-1.28526962	-1.16061735
6	0.43128470	0.03093043	0.44978261	1	-3.84261537	2.44353056	-1.25021732
1	1.22338474	-2.79606962	0.06808261	1	-2.65361547	2.60743046	0.03828261
6	-0.99271524	-1.73116958	-0.77301735	1	-2.18141532	1.94523048	-1.53171742
6	-0.87151527	-0.28246957	-0.37231740	1	2.92798471	-2.39456964	0.09958261
6	-2.14271522	-0.05896959	0.45818260	1	1.83508480	-0.52756959	-1.13721740
6	-2.45081544	-1.46426952	0.94188261	1	1.92368472	-2.09706950	1.52298260
8	-1.81681526	-2.37816954	0.12568261	1	-0.37031528	2.03833055	0.76198262
8	-0.50151527	-2.30696964	-1.68841732	1	0.85458469	1.93913054	-0.49891737
8	-3.13171530	-1.82066953	1.84778261	1	1.32028472	1.87503052	1.20378268
6	-3.36251545	0.56923044	-0.28841740	1	3.96588445	-0.73446959	2.30328274
1	-1.95011520	0.55543041	1.34128261	1	2.54638457	0.30183044	2.43368268
6	-4.53351498	0.71513045	0.68768263	1	4.15718508	1.00673044	2.47958255
6	-2.97771525	1.96503043	-0.78461736	1	5.11248493	-1.00936961	0.17208260
6	-3.79441547	-0.29456958	-1.47551739	6	4.74748468	0.09773043	-1.70091736
1	-0.83241522	0.30613044	-1.28951740	1	4.48138475	-0.75356960	-2.33971739
6	0.56818473	1.55463040	0.48388261	1	5.80028486	0.31673041	-1.90811741
1	0.25078472	-0.32266960	1.47388268	1	4.15888500	0.95633042	-2.03191733

TS__tBuMA--EB-rad_EB__SSS

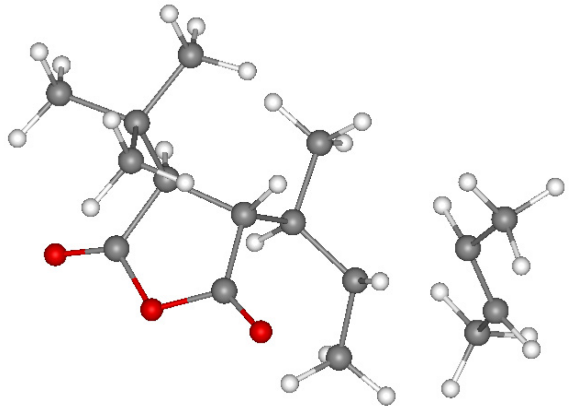

Zero-point vibrational energy	1059725.2 (Joules/Mol)
	253.28041 (Kcal/Mol)
Zero-point correction=	0.403628 (Hartree/Particle)
Thermal correction to Energy=	0.426035
Thermal correction to Enthalpy=	0.426979
Thermal correction to Gibbs Free Energy=	0.351196
Sum of electronic and zero-point Energies=	-851.147760
Sum of electronic and thermal Energies=	-851.125354
Sum of electronic and thermal Enthalpies=	-851.124409
Sum of electronic and thermal Free Energies=	-851.200192

cartesian

6	3.35984135	1.44054127	-1.34708703	1	-5.38245869	1.06784129	0.60411298
6	3.50024128	0.58934122	-0.11188696	1	-4.88005829	-0.55915880	1.07411301
6	4.54294157	-0.29265881	0.01011304	1	-4.25285864	0.84004122	1.94061303
1	3.13834119	1.03404117	0.81021309	1	-4.66195869	0.66774124	-1.77558696
6	2.05314136	-2.04405880	-0.07028696	1	-3.03385878	0.13034120	-2.15968704
6	1.62704122	-0.63365877	-0.33478695	1	-4.16785860	-0.96925879	-1.36678696
6	0.42474130	-0.14875880	0.45641306	1	-3.84095883	2.64584136	-0.47028697
1	1.25934124	-2.76355863	-0.30618697	1	-2.65515876	2.45294118	0.81791306
6	-1.01665866	-1.53825879	-1.13018703	1	-2.18115878	2.22784138	-0.86978698
6	-0.89725876	-0.23265880	-0.37988696	1	2.91944122	-2.32005882	-0.67258692
6	-2.15605879	-0.23375881	0.49831304	1	1.58794129	-0.37385881	-1.38958704
6	-2.47335863	-1.71365881	0.59941304	1	2.31424117	-2.19205880	0.98181295
8	-1.84405875	-2.38915873	-0.42498696	1	-0.38135868	1.60344124	1.48021305
8	-0.51725870	-1.87295890	-2.15448689	1	0.77454126	1.98934126	0.20961305
8	-3.15675879	-2.28875875	1.38281298	1	1.32964122	1.34184122	1.75511301
6	-3.37625885	0.58014119	-0.03868696	1	2.35264134	1.84124136	-1.47168696
1	-1.94905877	0.12144122	1.51061296	1	3.60204124	0.87264115	-2.24868703
6	-4.53795862	0.46814120	0.95271301	1	4.04414129	2.29394126	-1.30498695
6	-2.98075867	2.05484128	-0.14658695	6	4.98044157	-0.91805881	1.28951299

6	-3.82595873	0.06794122	-1.40888703	1	5.06484127	-0.60705876	-0.89238691
1	-0.87585866	0.57064122	-1.11768699	1	4.91714144	-2.01145887	1.25791299
6	0.54394126	1.27514124	1.00281298	1	4.38074160	-0.57285881	2.13461304
1	0.29994127	-0.82155883	1.31531298	1	6.02764130	-0.67995882	1.50941300

tBuMA--EB--EB-rad__RRR

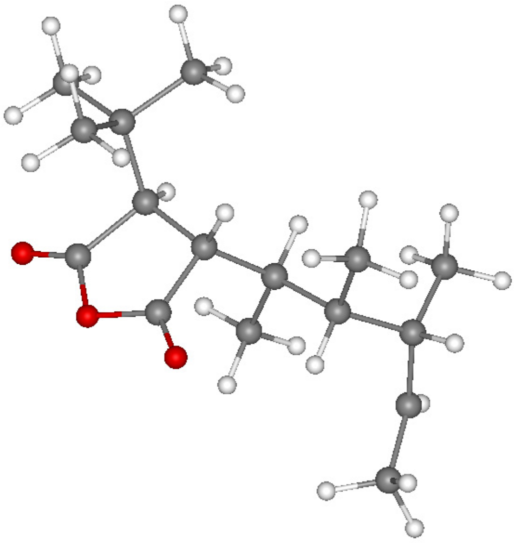

Zero-point vibrational energy	1068546.1 (Joules/Mol)
	255.38865 (Kcal/Mol)
Zero-point correction=	0.406988 (Hartree/Particle)
Thermal correction to Energy=	0.429117
Thermal correction to Enthalpy=	0.430062
Thermal correction to Gibbs Free Energy=	0.355814
Sum of electronic and zero-point Energies=	-851.193878
Sum of electronic and thermal Energies=	-851.171749
Sum of electronic and thermal Enthalpies=	-851.170805
Sum of electronic and thermal Free Energies=	-851.245052

cartesian

1	3.82103491	0.74531090	-0.80423915	1	-4.65076542	-0.41338915	1.16596091
6	3.16303492	0.46321085	0.03476087	1	-3.87336540	0.91991091	2.01466107
6	3.87023449	-0.64008915	0.75146085	1	-4.43966532	0.82841086	-1.68513918
6	3.01993465	1.70011091	0.91876084	1	-2.88446522	0.14531085	-2.13473892
1	2.01493502	-0.93628919	-1.12933910	1	-4.08496523	-0.85098910	-1.30233920
6	1.83263481	0.04241085	-0.67353916	1	-3.38916540	2.71551085	-0.36553913
6	0.64033478	-0.10848913	0.29806089	1	-2.15736532	2.37671089	0.84886086
6	-0.75306517	-1.73978913	-1.05033910	1	-1.80246520	2.15151095	-0.86743915
6	-0.70576519	-0.36168915	-0.43343914	6	1.52643478	1.01571095	-1.81453919

6	-1.93846524	-0.32148913	0.47666085	1	2.43383503	1.24741089	-2.37623906
6	-2.36116505	-1.77518916	0.55796087	1	1.11693478	1.96451092	-1.45383918
8	-1.69016516	-2.50968909	-0.39803916	1	0.82033479	0.59291089	-2.53073907
8	-0.10866518	-2.18428922	-1.94563913	1	0.02893481	-1.28628910	2.03826094
8	-3.14306498	-2.29728913	1.28466082	1	1.74553478	-0.93138909	1.98096097
6	-3.10146523	0.60291088	0.00126087	1	1.07593477	-2.15148926	0.91266084
1	-1.67886519	-0.00358912	1.49036086	1	2.45143461	1.48801088	1.82836080
6	-4.22866535	0.58241081	1.03716087	1	2.52373505	2.52291083	0.39946085
6	-2.57416534	2.03671098	-0.10313913	1	4.00313473	2.05791092	1.23186088
6	-3.64976501	0.15041086	-1.35393918	1	4.09233475	-0.52258915	1.80766082
1	-0.79456520	0.35531089	-1.24703920	6	4.28583479	-1.89168906	0.06776087
1	0.49943483	0.85791087	0.79926085	1	3.50353479	-2.66488910	0.07926087
6	0.88183486	-1.17468917	1.36416090	1	5.16503477	-2.33668923	0.53976083
1	-5.03146553	1.25561094	0.72596085	1	4.52513456	-1.71708906	-0.98673916

tBuMA--EB--EB-rad__RRS

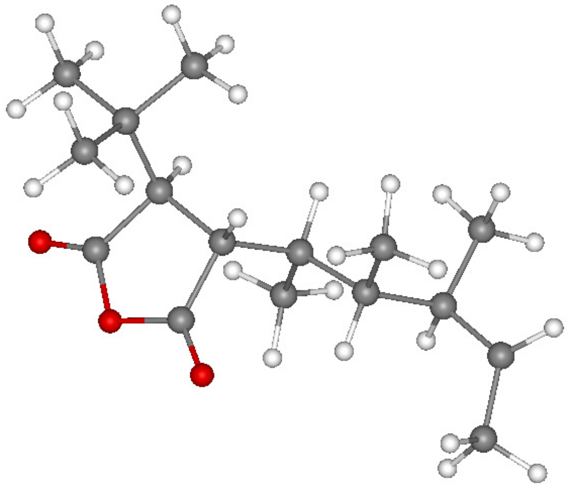

Zero-point vibrational energy	1067448.4 (Joules/Mol)
	255.12628 (Kcal/Mol)
Zero-point correction=	0.406570 (Hartree/Particle)
Thermal correction to Energy=	0.428911
Thermal correction to Enthalpy=	0.429856
Thermal correction to Gibbs Free Energy=	0.354568
Sum of electronic and zero-point Energies=	-851.196573
Sum of electronic and thermal Energies=	-851.174232
Sum of electronic and thermal Enthalpies=	-851.173287
Sum of electronic and thermal Free Energies=	-851.248575

cartesian							
6	-3.07667637	1.45450664	-1.29256523	1	4.89042377	-0.06949344	-0.76796520
6	-3.05367613	0.06870657	-0.65196520	1	4.13022375	1.32330656	-1.53276527
6	-4.30427647	-0.18169343	0.12823479	1	4.20922375	0.72720653	2.16423488
1	-3.03907633	-0.67799342	-1.45786524	1	2.66292381	-0.09769344	2.29063487
1	-1.88697612	-1.21459341	0.60033476	1	4.03432369	-0.89519346	1.50833476
6	-1.78257620	-0.19579343	0.21243478	1	3.20082378	2.72360659	0.98663479
6	-0.47907615	-0.15989344	-0.61516523	1	2.18032384	2.50360656	-0.43306524
6	0.82952386	-1.92949343	0.64603478	1	1.60572386	2.00640655	1.16253483
6	0.77332389	-0.47319344	0.24683480	6	-1.72897613	0.75080651	1.41243482
6	2.10902381	-0.23149343	-0.46556523	1	-2.72157621	0.86930662	1.85193479
6	2.64682388	-1.63139343	-0.68966520	1	-1.37187612	1.74770665	1.13663483
8	1.90222383	-2.53889346	0.03453479	1	-1.08287609	0.36960655	2.20503497
8	0.09952386	-2.54319334	1.35643482	1	0.42652386	-1.12459350	-2.35956502
8	3.56122398	-1.99349332	-1.35666525	1	-1.25557613	-0.70709348	-2.57746506
6	3.12662363	0.67720658	0.29043478	1	-0.82357609	-2.08719349	-1.57266521
1	1.95922387	0.21590656	-1.45256519	1	-2.21177626	1.63250661	-1.93586528
6	4.37902355	0.87110656	-0.56866521	1	-3.09567595	2.24410653	-0.53696525
6	2.48342371	2.04860663	0.51393479	1	-3.97017622	1.57340646	-1.90896523
6	3.52272367	0.06260657	1.63463473	6	-4.64787626	-1.52879333	0.65113479
1	0.70722389	0.10390657	1.16773474	1	-4.90707636	0.66720659	0.43723479
1	-0.32617617	0.87260658	-0.95466524	1	-4.18197632	-1.73729348	1.62623477
6	-0.53497612	-1.06759346	-1.84376526	1	-4.31127644	-2.32259345	-0.02356521
1	5.07842350	1.53940654	-0.05996522	1	-5.72507620	-1.64469337	0.79333478

tBuMA--EB--EB-rad__SSR

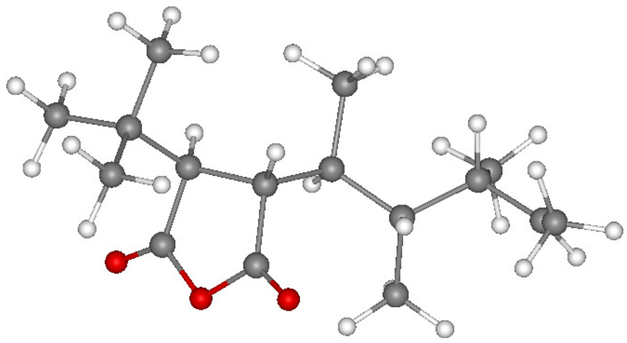

Zero-point vibrational energy	1068585.2 (Joules/Mol)
	255.39800 (Kcal/Mol)
Zero-point correction=	0.407003 (Hartree/Particle)
Thermal correction to Energy=	0.429067
Thermal correction to Enthalpy=	0.430011
Thermal correction to Gibbs Free Energy=	0.356285

Sum of electronic and zero-point Energies= -851.189325

Sum of electronic and thermal Energies= -851.167261

Sum of electronic and thermal Enthalpies= -851.166317

Sum of electronic and thermal Free Energies= -851.240043

cartesian

1	2.93914795	1.31067395	-0.12352826	1	-5.33645201	0.95957398	0.01807173
6	3.12094784	0.34127393	0.35917175	1	-4.77685213	-0.41152605	0.98047173
6	4.28774786	-0.27042606	-0.34782827	1	-4.29155207	1.22447395	1.41527176
6	3.45484781	0.59627390	1.82917166	1	-4.43115187	-0.06362605	-2.09452820
6	1.95544779	-1.84442604	0.87547171	1	-2.75415206	-0.57262605	-2.21902823
6	1.82814777	-0.50562608	0.14817172	1	-3.88095212	-1.46942604	-1.19362831
6	0.51134783	0.21627393	0.54207170	1	-3.81525207	2.25807405	-1.40492833
1	1.13224781	-2.52692604	0.66887170	1	-2.71895218	2.54737401	-0.05762827
6	-0.74235219	-1.71062601	-0.64842826	1	-2.10955215	1.86887407	-1.57232833
6	-0.72405219	-0.24252605	-0.29872826	1	2.87094784	-2.36002612	0.57757169
6	-2.04335213	-0.07012606	0.46877173	1	1.77644789	-0.70872605	-0.92762828
6	-2.28795218	-1.47312605	0.99277169	1	1.99104774	-1.70072603	1.95837176
8	-1.56795216	-2.37522602	0.23527172	1	-0.41035217	2.17987394	0.68317169
8	-0.18645218	-2.28512621	-1.52752829	1	0.80094779	2.05647397	-0.58582830
8	-2.98395205	-1.83932602	1.88277173	1	1.29144776	2.18187404	1.10407174
6	-3.26615214	0.45507392	-0.34852827	1	3.81124783	-0.31182605	2.32037187
1	-1.92535222	0.58677399	1.33407176	1	2.59684801	0.95797396	2.40087175
6	-4.48565197	0.55567396	0.57227170	1	4.24884796	1.34117401	1.91547167
6	-2.94865203	1.85727406	-0.87362832	1	5.02484798	-0.81352603	0.23607174
6	-3.59205198	-0.46882606	-1.52472830	6	4.35694790	-0.34972605	-1.82952833
1	-0.69735217	0.30097392	-1.24432826	1	3.85914803	-1.24622607	-2.22942805
6	0.55694783	1.73987389	0.43207175	1	5.38994789	-0.38822606	-2.18432808
1	0.29904783	-0.03712606	1.58907175	1	3.87194777	0.50977397	-2.30372810

tBuMA--EB--EB-rad__SSS

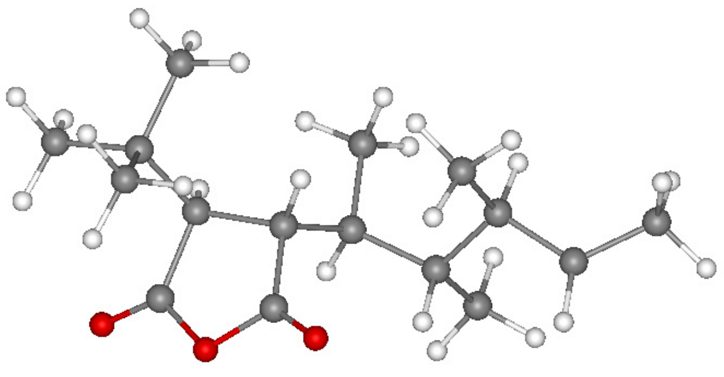

Zero-point vibrational energy	1068467.8 (Joules/Mol)
	255.36994 (Kcal/Mol)
Zero-point correction=	0.406958 (Hartree/Particle)
Thermal correction to Energy=	0.429148
Thermal correction to Enthalpy=	0.430092
Thermal correction to Gibbs Free Energy=	0.355522
Sum of electronic and zero-point Energies=	-851.191532
Sum of electronic and thermal Energies=	-851.169342
Sum of electronic and thermal Enthalpies=	-851.168398
Sum of electronic and thermal Free Energies=	-851.242968

cartesian

6	-2.08544779	0.96310437	1.24858487	1	5.17665243	1.28600430	-0.07951518
6	-2.70594764	0.11290434	0.12528481	1	4.93905258	-0.44729564	-0.32401520
6	-3.98874784	-0.47789565	0.62448484	1	4.40145254	0.69590437	-1.55081522
1	-2.95084786	0.78800428	-0.70691514	1	3.96935225	1.21380436	2.13368464
6	-2.48264766	-1.75909567	-1.54511511	1	2.35485220	0.53260434	2.26768470
6	-1.78044760	-1.00329566	-0.41351518	1	3.74025226	-0.52059567	1.95678484
6	-0.37294766	-0.56859565	-0.90231514	1	3.30055213	2.81310439	0.31208482
1	-1.89084756	-2.61539555	-1.87821519	1	2.44375229	2.24330449	-1.11831522
6	0.74915230	-1.54769564	1.15948486	1	1.63845241	2.26080441	0.45388481
6	0.71495235	-0.38099563	0.19688481	1	-3.45364785	-2.13369560	-1.21781516
6	2.13715219	-0.38359565	-0.38521519	1	-1.64584768	-1.71379566	0.40758482
6	2.59785223	-1.80439568	-0.12891519	1	-2.66184783	-1.11979568	-2.41451526
8	1.79505241	-2.38199568	0.83258486	1	0.53115237	0.85520434	-2.29531527
8	0.03045234	-1.79959571	2.07308483	1	-0.69484770	1.56450438	-1.25331521
8	3.50125217	-2.40639544	-0.61151516	1	-1.16204762	0.53020442	-2.59961534
6	3.12855220	0.66050434	0.21808481	1	-1.27934766	1.60710430	0.88798481
1	2.13605237	-0.23979564	-1.46781516	1	-1.68964767	0.32950434	2.04478478
6	4.48875237	0.53340435	-0.47311518	1	-2.83774781	1.62250435	1.68688488
6	2.58825231	2.06760454	-0.04931519	6	-5.26724768	0.27660438	0.58958483
6	3.30065227	0.45300439	1.72478485	1	-3.91544795	-1.34869564	1.27188480
1	0.49685234	0.51290441	0.77728486	1	-5.41954756	0.77030432	-0.37611520
6	-0.42514768	0.66280437	-1.80611515	1	-5.30954742	1.07210433	1.35068488
1	-0.01424766	-1.40339565	-1.52041519	1	-6.12644768	-0.37179562	0.77378482

S9. MA – ZB reaction profile

TS__tBuMA-rad_ZB__re

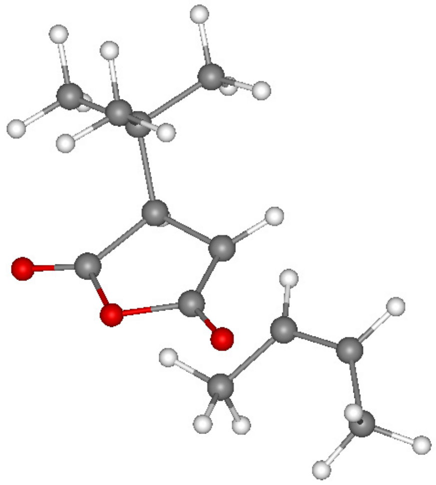

Zero-point vibrational energy	761854.2 (Joules/Mol)
	182.08753 (Kcal/Mol)
Zero-point correction=	0.290175 (Hartree/Particle)
Thermal correction to Energy=	0.307129
Thermal correction to Enthalpy=	0.308073
Thermal correction to Gibbs Free Energy=	0.245171
Sum of electronic and zero-point Energies=	-694.034786
Sum of electronic and thermal Energies=	-694.017833
Sum of electronic and thermal Enthalpies=	-694.016889
Sum of electronic and thermal Free Energies=	-694.079791

cartesian

6	4.17373514	0.20407355	-0.45772943	1	1.39423537	1.15287352	1.41997051
6	3.03343534	0.94907355	0.13627058	6	2.16043544	-0.90202647	1.62847054
6	2.05333543	0.43127352	0.94357055	1	3.97063541	-0.02732646	-1.50912952
1	4.37643528	-0.73882645	0.04967057	1	-3.07876468	2.32387352	-0.38802943
6	0.80253530	-1.04982638	-1.21302950	1	-1.83446467	2.23937345	0.85707057
6	0.34483534	0.12377355	-0.50402945	1	-1.38516462	2.26807356	-0.85412943
6	-0.85216469	-0.24932645	0.30757058	1	-3.46886468	0.34837353	-1.93902946
6	-0.81536466	-1.77112651	0.22207057	1	-1.75436461	0.17697352	-2.30502939
8	0.11913534	-2.16102648	-0.69672942	1	-2.66586471	-1.19112647	-1.65292943
8	1.62773538	-1.19052649	-2.06752944	1	-4.28036499	0.34967357	0.46257061
8	-1.46426463	-2.56712651	0.82557058	1	-3.43886471	-1.16762638	0.80747056
6	-2.21436453	0.35367352	-0.17452943	1	-3.13146472	0.25637352	1.79907048
1	-0.75256467	0.02227354	1.36597049	1	2.92403531	1.98617363	-0.17242943
6	-3.32816458	-0.08442646	0.77857059	1	1.20923543	-1.21732640	2.05947042

6	-2.11226463	1.87987351	-0.13782942	1	2.87383533	-0.84092641	2.45607042
6	-2.53686452	-0.10722646	-1.59662950	1	2.50203538	-1.69342649	0.96067053
1	0.46313536	1.08587360	-0.97752941	1	5.08543491	0.80757356	-0.43382943

TS__tBuMA-rad_ZB__si

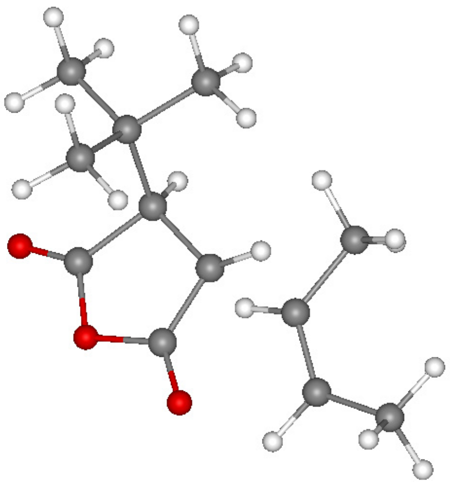

Zero-point vibrational energy	762153.9 (Joules/Mol)
	182.15915 (Kcal/Mol)
Zero-point correction=	0.290289 (Hartree/Particle)
Thermal correction to Energy=	0.307240
Thermal correction to Enthalpy=	0.308185
Thermal correction to Gibbs Free Energy=	0.245279
Sum of electronic and zero-point Energies=	-694.034099
Sum of electronic and thermal Energies=	-694.017148
Sum of electronic and thermal Enthalpies=	-694.016204
Sum of electronic and thermal Free Energies=	-694.079109

cartesian

6	3.15062046	-0.57176769	0.56296766	1	-1.49477947	2.30153227	-0.05093236
6	2.07702041	0.13693231	1.03986764	1	-1.04577947	1.59113240	-1.60863233
6	0.63802058	-1.83586764	-0.56963235	1	-3.36657953	-0.32426769	-1.95733237
6	0.38872054	-0.42916769	-0.33723235	1	-1.69167948	-0.83956766	-2.13803244
6	-0.85417944	-0.30456769	0.48196763	1	-2.77937937	-1.71266770	-1.05033231
6	-1.03087950	-1.73536766	0.97706765	1	-4.17307949	0.71513236	0.19816764
8	-0.19047944	-2.56886768	0.29146764	1	-3.53487945	-0.62106770	1.16496754
8	1.41242051	-2.39296770	-1.28933239	1	-3.05027938	1.03533232	1.52166772
8	-1.76707947	-2.14716768	1.81766772	6	4.14062071	-0.11996770	-0.44793236
6	-2.12207937	0.22233230	-0.27433237	1	0.90362060	1.94933224	1.08306766
1	-0.72667944	0.33713228	1.36136770	1	2.25392056	2.01043224	-0.04523237
6	-3.28547955	0.33793229	0.71266764	1	2.54162049	2.12393236	1.68906760

6	-1.81747949	1.61213231	-0.83623236	1	4.06682062	0.94103229	-0.68503237
6	-2.50407958	-0.72036767	-1.41633224	1	4.01252031	-0.69016767	-1.37483239
1	0.68502057	0.27653229	-1.09843230	1	5.16122055	-0.31746769	-0.10553236
6	1.93442047	1.62883234	0.92506766	1	1.54992056	-0.31306770	1.87826753
1	-2.71687937	2.03453231	-1.29093242	1	3.23522043	-1.60986769	0.87096763

TS__tBuMA-rad_to_ZB_transfer

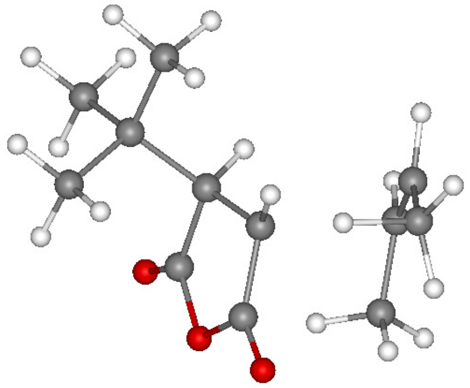

Zero-point vibrational energy	749920.4 (Joules/Mol)
	179.23528 (Kcal/Mol)
Zero-point correction=	0.285630 (Hartree/Particle)
Thermal correction to Energy=	0.302698
Thermal correction to Enthalpy=	0.303642
Thermal correction to Gibbs Free Energy=	0.238900
Sum of electronic and zero-point Energies=	-694.029721
Sum of electronic and thermal Energies=	-694.012653
Sum of electronic and thermal Enthalpies=	-694.011709
Sum of electronic and thermal Free Energies=	-694.076451

cartesian

6	2.39602065	0.37647355	1.52162349	1	3.26082063	-2.23512650	-0.55827641
6	2.64652061	-0.93092644	0.94812357	6	3.56732059	-0.20112643	-1.31037641
6	3.14992070	-1.19022644	-0.27877644	1	-3.44387937	-1.06822646	1.52182353
1	1.19382048	0.75767356	1.19192350	1	-1.76967955	-1.55222642	1.78822362
6	0.18302053	2.29697371	-0.08267644	1	-2.29017949	0.09897356	2.15112376
6	-0.09927947	1.15747356	0.78582358	1	-4.00707960	0.61997354	-0.29677644
6	-0.56767946	0.02657357	-0.08107644	1	-2.75367951	1.74187350	0.22362356
6	-0.14957947	0.51317358	-1.46257651	1	-2.78047943	1.14897358	-1.44227648
8	0.22142053	1.83057356	-1.40077651	1	-3.44007945	-1.78922653	-0.83507645
8	0.41222054	3.43947363	0.16942357	1	-2.15537930	-1.30202651	-1.94837642
8	-0.11487947	-0.09132645	-2.48807645	1	-1.79757953	-2.39742637	-0.61527646
6	-2.08577943	-0.33032647	0.00862357	1	2.97112060	1.21137357	1.12632358

1	-0.00597947	-0.89632642	0.11262356	1	2.33292055	0.40777355	2.60732365
6	-2.38287950	-1.52052653	-0.90577644	1	2.37042069	-1.78562641	1.56112349
6	-2.40717936	-0.73032641	1.45042360	1	4.59592056	-0.38892642	-1.63297641
6	-2.94747949	0.86507356	-0.39997643	1	3.50262070	0.83267355	-0.97197646
1	-0.54717946	1.37927353	1.74692357	1	2.94182062	-0.29442647	-2.20397639

tBuMA--ZB-rad__re__mpw

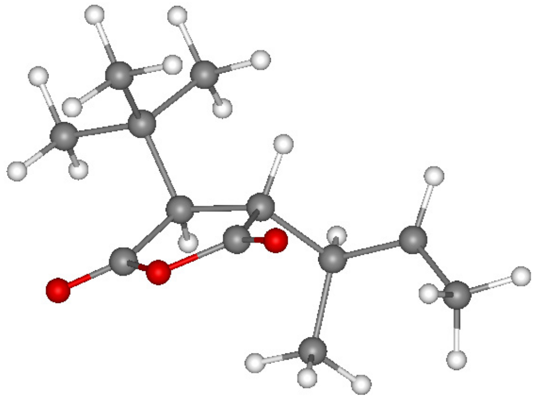

Zero-point vibrational energy	768951.4 (Joules/Mol)
	183.78380 (Kcal/Mol)
Zero-point correction=	0.292878 (Hartree/Particle)
Thermal correction to Energy=	0.309638
Thermal correction to Enthalpy=	0.310582
Thermal correction to Gibbs Free Energy=	0.248427
Sum of electronic and zero-point Energies=	-694.065970
Sum of electronic and thermal Energies=	-694.049211
Sum of electronic and thermal Enthalpies=	-694.048267
Sum of electronic and thermal Free Energies=	-694.110422

cartesian

6	2.81422353	-0.90579414	0.12490588	1	0.40522355	-0.67239416	1.09870589
6	4.10732365	-0.19439414	0.28760588	1	1.19802356	-1.25219405	-1.17689407
6	1.63892353	-0.39539412	-0.64609414	6	1.99772346	0.66400588	-1.68429410
1	4.03392363	0.61090589	1.03120589	1	-4.19317627	-0.59209406	-0.20849413
1	4.45472383	0.26660585	-0.64109409	1	-3.55447626	1.00240588	-0.62549412
6	0.83722359	1.38650584	0.96950591	1	-3.19647646	-0.38509414	-1.65009415
6	0.49282354	0.07380587	0.30780590	1	-3.17327642	-0.47359416	2.09340596
6	-0.84467643	0.34170586	-0.38709411	1	-1.46347654	-0.13479415	2.31590581
6	-0.97007644	1.85200596	-0.33299410	1	-2.55177641	1.13220584	1.73500586
8	-0.01497646	2.37000585	0.51560593	1	-2.73117638	-2.41829419	0.53840590
8	1.69982350	1.63330591	1.74850583	1	-1.65587652	-2.22949433	-0.84509414
8	-1.73867655	2.56740570	-0.88959414	1	-1.00657654	-2.20769429	0.79860586

6	-2.08537626	-0.37789413	0.22490588	1	2.62602353	-1.76719415	0.76030588
1	-0.80617642	0.06110588	-1.44409406	1	1.12642348	0.98320591	-2.26059413
6	-3.32707644	-0.06259412	-0.61349410	1	2.72532368	0.27060586	-2.39619422
6	-1.84617651	-1.88939416	0.17660588	1	2.43622375	1.55340588	-1.22589409
6	-2.32267642	0.06530586	1.67010593	1	4.88822365	-0.87619412	0.63040590

tBuMA--ZB-rad__si

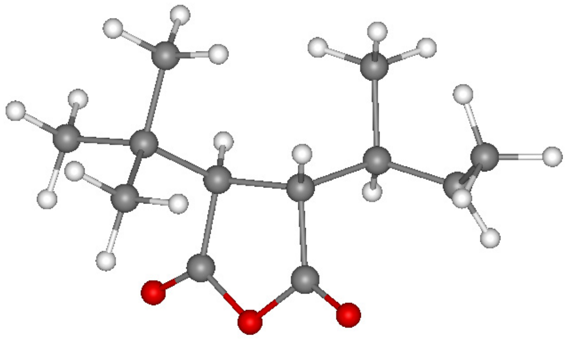

Zero-point vibrational energy	768915.9 (Joules/Mol)
	183.77531 (Kcal/Mol)
Zero-point correction=	0.292865 (Hartree/Particle)
Thermal correction to Energy=	0.309726
Thermal correction to Enthalpy=	0.310671
Thermal correction to Gibbs Free Energy=	0.248350
Sum of electronic and zero-point Energies=	-694.065140
Sum of electronic and thermal Energies=	-694.048278
Sum of electronic and thermal Enthalpies=	-694.047334
Sum of electronic and thermal Free Energies=	-694.109654

cartesian

6	3.08082056	-0.49884412	0.52214706	1	-3.61197948	-0.68384409	0.64194709
6	1.71892059	-0.11744413	1.01474702	1	-3.17057943	0.87325585	1.33594704
6	0.69572055	-1.92214406	-0.42255294	1	-2.80357933	-0.00994414	-2.28535295
6	0.58882052	-0.47324410	-0.00595294	1	-1.13267946	-0.54924411	-2.21655297
6	-0.82527947	-0.37664413	0.57814705	1	-2.41067934	-1.52034414	-1.47565293
6	-1.15737939	-1.82474411	0.88494706	1	-2.27007937	2.21155596	-1.23395300
8	-0.28777948	-2.65314388	0.20944707	1	-1.36127937	2.30305576	0.27284706
8	1.47942054	-2.44324398	-1.14725304	1	-0.56607950	1.78455579	-1.21725297
8	-2.01767945	-2.27254415	1.57134700	6	3.68272066	0.06395590	-0.71425295
6	-1.90667939	0.27935588	-0.33765292	1	0.68502051	1.64975584	1.80324697
1	-0.83867949	0.15965587	1.52994704	1	1.81552064	2.00205612	0.49954706
6	-3.24807930	0.31385589	0.39964706	1	2.41902065	1.61275589	2.10974693
6	-1.49277937	1.72115600	-0.64305294	1	3.40712070	-0.53404409	-1.59395301

6	-2.06417942	-0.49794412	-1.64645302	1	4.77422047	0.05805588	-0.66335291
1	0.73342055	0.14125592	-0.89545292	1	3.36932063	1.09265590	-0.91175294
6	1.64972055	1.37065589	1.37504697	1	1.51802063	-0.69244409	1.92674696
1	-3.99987936	0.80855584	-0.22025295	1	3.54752064	-1.37634408	0.95524710

TS__tBuMA--ZB-rad_MA__R_re

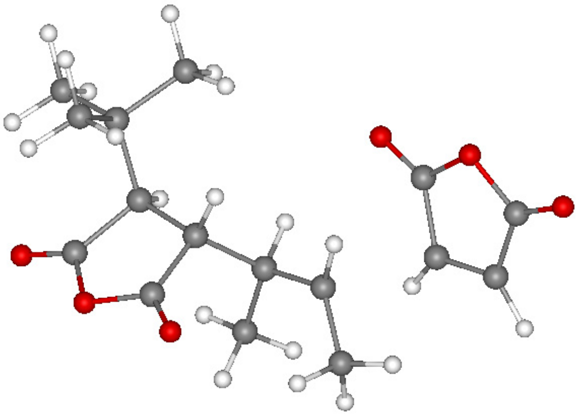

Zero-point vibrational energy	922004.5 (Joules/Mol)
	922004.5 (Kcal/Mol)
Zero-point correction=	0.351173 (Hartree/Particle)
Thermal correction to Energy=	0.373627
Thermal correction to Enthalpy=	0.374571
Thermal correction to Gibbs Free Energy=	0.297582
Sum of electronic and zero-point Energies=	-1073.299292
Sum of electronic and thermal Energies=	-1073.276838
Sum of electronic and thermal Enthalpies=	-1073.275894
Sum of electronic and thermal Free Energies=	-1073.352883

cartesian

6	3.15657449	1.12627685	0.49689767	6	-3.55592537	2.15357661	0.50289768
6	3.49207449	-0.21842325	1.02079773	6	-1.26742554	2.54757667	-0.37350231
6	4.67187452	-0.58382326	0.46519765	6	-2.93892550	1.29197681	-1.75960231
6	5.04087496	0.42967671	-0.52530229	1	-0.38202545	0.12667675	-1.21170235
8	4.07257462	1.43937683	-0.47330233	1	0.67807454	0.18127675	1.01419771
8	2.26847458	1.86427677	0.80299771	6	0.08567455	-1.77942324	1.65769768
8	5.96137476	0.49037671	-1.27610230	1	-3.87902570	3.10097671	0.06469769
1	3.00627446	-0.62962323	1.89209771	1	-4.42202520	1.49597681	0.56669772
1	5.27617455	-1.45822322	0.65109771	1	-3.21902537	2.35967660	1.52249765
6	2.13457465	-2.57982326	-0.38580233	1	-3.25712538	2.22977662	-2.22000241
6	1.64527452	-1.18572319	-0.25430232	1	-2.17152548	0.86347675	-2.41010237
1	1.85697448	-0.53232324	-1.09800231	1	-3.80132556	0.61997676	-1.76670229
6	0.44607455	-0.78892326	0.55479771	1	-1.61172545	3.50347662	-0.77500230

6	-1.36472547	-1.72342324	-0.95400232	1	-0.86862552	2.73477674	0.62639773
6	-0.76102543	-0.46392328	-0.37660232	1	-0.43582544	2.21887660	-0.99990231
6	-1.93232536	0.22967674	0.32509768	1	3.12707448	-2.60842323	-0.83880228
6	-3.00532556	-0.84052324	0.35109767	1	2.17127466	-3.11062336	0.56769770
8	-2.63502550	-1.89412320	-0.46800232	1	1.46137452	-3.14232326	-1.04480231
8	-0.87282550	-2.51172328	-1.69720232	1	-0.75842547	-1.42632318	2.25319767
8	-4.03592539	-0.86922324	0.93879771	1	0.91877460	-1.91982317	2.34959769
6	-2.42892551	1.55097675	-0.34040233	1	-0.17622545	-2.76292324	1.26059771
1	-1.69012547	0.47097671	1.36409771				

TS__tBuMA--ZB-rad_MA__R_si

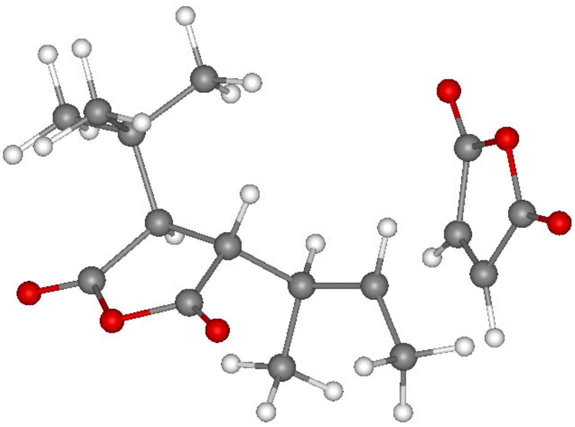

Zero-point vibrational energy	922356.5 (Joules/Mol)
	220.44849 (Kcal/Mol)
Zero-point correction=	0.351307 (Hartree/Particle)
Thermal correction to Energy=	0.373682
Thermal correction to Enthalpy=	0.374626
Thermal correction to Gibbs Free Energy=	0.297540
Sum of electronic and zero-point Energies=	-1073.297844
Sum of electronic and thermal Energies=	-1073.275469
Sum of electronic and thermal Enthalpies=	-1073.274525
Sum of electronic and thermal Free Energies=	-1073.351612

cartesian

6	-3.45259786	1.72493958	-0.38871628	6	2.88830233	1.12283957	1.81168377
6	-3.28619766	0.65533954	-1.37511623	1	0.40830222	-0.21216044	1.30258369
6	-3.60189772	-0.52106047	-0.77701628	1	-0.85029781	0.15293954	-0.82071626
6	-4.16249800	-0.19756046	0.55758375	6	-0.24919780	-1.65786052	-1.78931630
8	-3.97869778	1.15243959	0.76798373	1	3.47600222	3.27373958	0.24058373
8	-3.20779777	2.88913965	-0.45541626	1	4.08550215	1.83773959	-0.58961630
8	-4.68299770	-0.90476048	1.36168373	1	2.71500230	2.72663975	-1.25331628
1	-2.93829775	0.84283954	-2.37961650	1	3.14410233	2.00633955	2.40018368

1	-3.75479770	-1.48566043	-1.23591626	1	2.24320221	0.49963951	2.43718362
6	-1.65459776	-1.41606045	0.30828372	1	3.81710243	0.57453954	1.63358378
6	-0.54009783	-0.85936046	-0.52421629	1	1.26550221	3.30783963	1.31268370
6	1.47470224	-1.89716041	0.62778372	1	0.44720218	2.67443967	-0.11061627
6	0.73160219	-0.61176044	0.33998373	1	0.25610217	1.88113952	1.45608377
6	1.77620220	0.29093957	-0.32281628	6	-2.04459786	-2.84706044	0.34848374
6	2.93110228	-0.65156049	-0.59881628	1	-3.01589775	-2.96816039	0.83108371
8	2.71160221	-1.86046052	0.03808372	1	-1.31219780	-3.40896034	0.94188374
8	1.10790217	-2.84676027	1.24268377	1	-2.07769775	-3.30936027	-0.64021629
8	3.91410208	-0.47906047	-1.24081624	1	-1.84009778	-0.86036044	1.22518373
6	2.21250224	1.54053950	0.50378370	1	0.51960218	-1.17996049	-2.39991641
1	1.42510223	0.65973955	-1.29141629	1	-1.14319777	-1.74006045	-2.41051650
6	3.18240213	2.38623977	-0.32521626	1	0.08830220	-2.67166042	-1.56381631
6	0.97220218	2.38683963	0.80408370				

TS__tBuMA--ZB-rad_MA__S_re

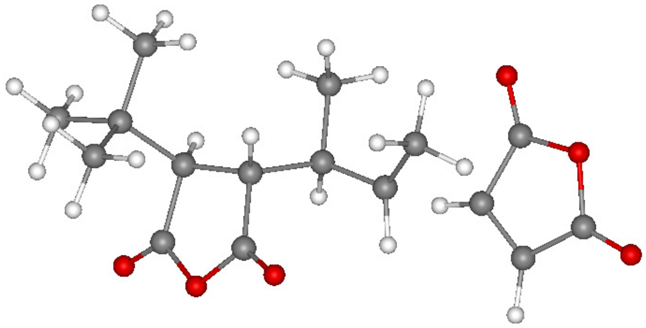

Zero-point vibrational energy	922194.9 (Joules/Mol)
	220.40987 (Kcal/Mol)
Zero-point correction=	0.351245 (Hartree/Particle)
Thermal correction to Energy=	0.373746
Thermal correction to Enthalpy=	0.374690
Thermal correction to Gibbs Free Energy=	0.297270
Sum of electronic and zero-point Energies=	-1073.294501
Sum of electronic and thermal Energies=	-1073.272001
Sum of electronic and thermal Enthalpies=	-1073.271056
Sum of electronic and thermal Free Energies=	-1073.348477

cartesian

6	4.34407473	0.78672320	0.65986514	6	-3.14692569	0.45612323	-0.14373487
6	3.69987440	-0.41937679	1.24186516	1	-1.69112563	0.51202321	1.44526517
6	4.52237463	-1.46877670	0.98776519	6	-4.29022551	0.66162324	0.85386515
6	5.60247421	-1.00377679	0.11706515	6	-2.76532555	1.82162309	-0.72053486
8	5.43587446	0.37462324	-0.05793486	6	-3.61362553	-0.46857679	-1.27023482

8	4.03657436	1.93522310	0.75046515	1	-0.71522552	0.08442321	-1.23953485
8	6.50457430	-1.58987689	-0.39043489	6	0.72257447	1.54012322	0.55406517
1	2.93637443	-0.34287676	2.00086522	1	0.65377450	-0.42157677	1.42746520
1	4.44107437	-2.49577689	1.30956519	1	-5.14542580	1.11522317	0.34736514
1	1.97047448	-1.62717676	-0.22833487	1	-4.62162542	-0.27387679	1.30286515
6	1.85717440	-0.55217677	-0.30723485	1	-3.99272537	1.33112311	1.66526520
6	2.35167456	0.04212323	-1.57673478	1	-4.45972538	-0.01957679	-1.79483485
6	0.67147446	0.01972324	0.42116514	1	-2.83442569	-0.64757681	-2.01623487
1	1.62987447	-0.15087678	-2.38313484	1	-3.94732571	-1.43837667	-0.89243484
1	3.28847432	-0.41697678	-1.89593494	1	-3.63932562	2.28312325	-1.18573487
6	-0.72112554	-1.89977670	-0.49823484	1	-2.41202569	2.50132322	0.05896514
6	-0.67122549	-0.40567678	-0.26633486	1	-1.99092555	1.75942326	-1.48833477
6	-1.90992546	-0.14357677	0.59936517	1	2.49797440	1.12192321	-1.51783478
6	-2.21302557	-1.51637673	1.16726518	1	-0.07672554	1.91342306	1.19526517
8	-1.55872560	-2.47977686	0.42296514	1	0.61657447	2.02802324	-0.41703483
8	-0.13952553	-2.55077672	-1.30543482	1	1.67117453	1.86572313	0.98026514
8	-2.89822555	-1.82047677	2.08736515				

TS__tBuMA--ZB-rad_MA__S_si

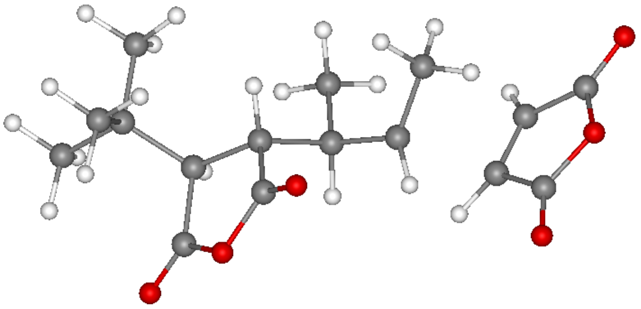

Zero-point vibrational energy 922318.4 (Joules/Mol)
220.43938 (Kcal/Mol)

Zero-point correction= 0.351293 (Hartree/Particle)
Thermal correction to Energy= 0.373790
Thermal correction to Enthalpy= 0.374734
Thermal correction to Gibbs Free Energy= 0.297076
Sum of electronic and zero-point Energies= -1073.293465
Sum of electronic and thermal Energies= -1073.270967
Sum of electronic and thermal Enthalpies= -1073.270023
Sum of electronic and thermal Free Energies= -1073.347681

cartesian

6	-5.45798826	-0.41883489	0.19583027	6	3.64921141	0.31226510	-1.28906977
6	-4.34578848	-0.59493494	1.13313019	1	0.71841180	-0.14193490	-1.25426972

6	-3.67218828	0.57776511	1.21813023	6	-0.78378826	-1.38383496	0.60123026
6	-4.44588804	1.58226502	0.44063026	1	-0.62438822	0.61816508	1.36403024
8	-5.46568823	0.91796505	-0.20066974	1	5.09701157	-1.14713490	0.51383030
8	-6.26488829	-1.19623494	-0.20596972	1	4.60231161	0.34726509	1.31433022
8	-4.27778816	2.75516510	0.33413026	1	3.91281176	-1.19233489	1.82083023
1	-4.15898848	-1.53493488	1.62943029	1	4.48611164	-0.21613494	-1.75086975
1	-2.93758821	0.87836510	1.94963026	1	2.88871193	0.44306511	-2.06396985
6	-1.84508824	0.69816506	-0.36026973	1	4.01311159	1.30296504	-1.00456977
6	-0.67328823	0.12346509	0.38483027	1	3.57931185	-2.41343474	-0.93006968
6	0.77771175	1.90326512	-0.71796978	1	2.32821178	-2.46813488	0.30803025
6	0.68231177	0.44256508	-0.33416975	1	1.95391166	-1.87083495	-1.31316972
6	1.90391171	0.23466510	0.56883025	1	-1.94268823	1.77726507	-0.32026973
6	2.24301195	1.65106511	0.99533021	6	-2.40218830	0.02756509	-1.56256974
8	1.62581170	2.54996514	0.14863026	1	-3.23838806	0.58606511	-1.98286986
8	0.22571176	2.47956514	-1.59766972	1	-2.73888826	-0.99233496	-1.35586977
8	2.93021154	2.02596521	1.88743019	1	-1.64308822	-0.04023489	-2.35366988
6	3.13101196	-0.47703490	-0.08426973	1	0.03291178	-1.76613486	1.21443021
1	1.65331185	-0.32043490	1.47613025	1	-0.76278824	-1.92453492	-0.34696972
6	4.24841166	-0.61753500	0.95323026	1	-1.71598828	-1.63703501	1.10623026
6	2.71391153	-1.88083494	-0.52906978				

tBuMA--ZB-rad--MA__R_re

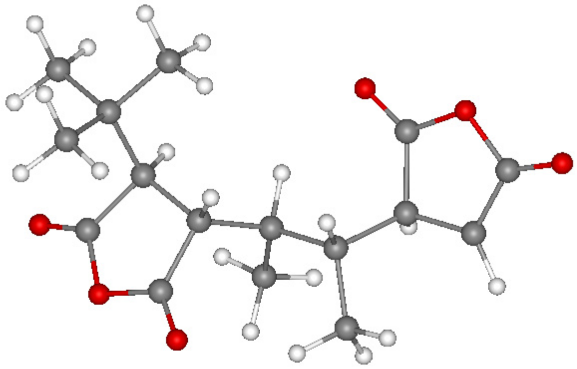

Zero-point vibrational energy	932064.7 (Joules/Mol)
	222.76881 (Kcal/Mol)
Zero-point correction=	0.355005 (Hartree/Particle)
Thermal correction to Energy=	0.376999
Thermal correction to Enthalpy=	0.377943
Thermal correction to Gibbs Free Energy=	0.301789
Sum of electronic and zero-point Energies=	-1073.336040
Sum of electronic and thermal Energies=	-1073.314046
Sum of electronic and thermal Enthalpies=	-1073.313102
Sum of electronic and thermal Free Energies=	-1073.389256

cartesian

6	3.08202314	1.07743478	0.58292788	6	-3.41067672	2.27183485	0.47382790
6	3.03782320	-0.44936514	0.60432792	6	-1.20607662	2.50203490	-0.64337212
6	4.36342335	-0.78866512	0.05032790	6	-3.01087666	1.13483477	-1.71657205
6	5.06622314	0.41183484	-0.31677210	1	-0.38887671	0.00063486	-1.21837211
8	4.24662352	1.50083482	0.01282790	1	0.66452330	0.27303487	1.05862784
8	2.26052332	1.84813476	0.97062790	6	0.22542331	-1.69746518	1.73862791
8	6.14222336	0.57273489	-0.80967212	1	-3.77237654	3.15883493	-0.05197210
1	2.95572329	-0.77736515	1.64992785	1	-4.26827669	1.64193475	0.70682788
1	4.79932308	-1.76906514	-0.06107210	1	-2.97417688	2.60373497	1.41972792
6	2.02882338	-2.50376511	-0.48437211	1	-3.34237671	2.02053499	-2.26277208
6	1.80912340	-1.02746511	-0.17117210	1	-2.31247687	0.60183489	-2.36797190
1	1.76452339	-0.48646516	-1.12347209	1	-3.88817692	0.49923486	-1.57017207
6	0.50882334	-0.71046513	0.60932791	1	-1.57777667	3.42433500	-1.09587204
6	-1.41267669	-1.77536523	-0.73197210	1	-0.69137669	2.77133489	0.28182790
6	-0.72577667	-0.49396515	-0.30587211	1	-0.46097669	2.09063482	-1.32787216
6	-1.84197676	0.29943484	0.38792789	1	2.87322330	-2.63766503	-1.16277206
6	-2.92247677	-0.73616511	0.61492789	1	2.23162317	-3.08566499	0.41872790
8	-2.63687682	-1.87186515	-0.12127209	1	1.15352333	-2.92796516	-0.97247213
8	-1.03037667	-2.62556505	-1.47167206	1	-0.62407672	-1.37486517	2.34522796
8	-3.89957666	-0.68276513	1.28692794	1	1.07462335	-1.78126514	2.42092800
6	-2.37377667	1.54553485	-0.38727209	1	0.00512332	-2.70106506	1.36872792
1	-1.52117670	0.65353489	1.37162793				

tBuMA--ZB-rad--MA__R_si

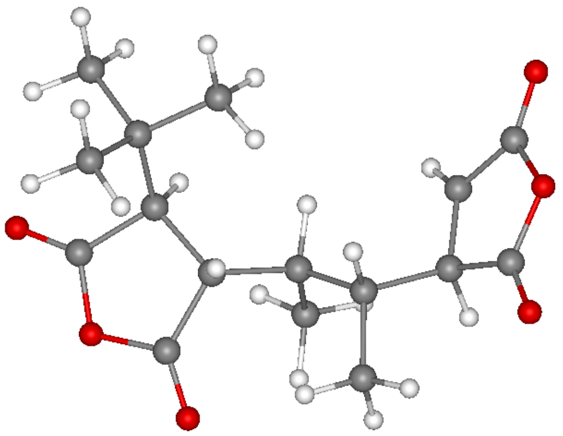

Zero-point vibrational energy	930741.8 (Joules/Mol)
	222.45263 (Kcal/Mol)
Zero-point correction=	0.354501 (Hartree/Particle)
Thermal correction to Energy=	0.376590
Thermal correction to Enthalpy=	0.377535

Thermal correction to Gibbs Free Energy= 0.301422
Sum of electronic and zero-point Energies= -1073.333111
Sum of electronic and thermal Energies= -1073.311022
Sum of electronic and thermal Enthalpies= -1073.310078
Sum of electronic and thermal Free Energies= -1073.386190

cartesian

6	4.08031845	1.35445571	0.63138366	6	-2.71448159	1.24205589	-1.85211623
6	3.06301856	0.50285584	1.19298375	1	-0.71008146	-0.61814415	-1.19651628
6	2.98951840	-0.76444417	0.43828371	1	0.56331855	0.26765582	0.94478369
6	4.13591862	-0.58874416	-0.55851632	6	0.39471853	-1.62484407	1.89828372
8	4.68311834	0.66055584	-0.41821629	1	-3.01418161	3.50935578	-0.35181630
8	4.42531824	2.45865583	0.93238366	1	-3.92868137	2.21585584	0.43488371
8	4.54091835	-1.35684419	-1.36871624	1	-2.47578144	2.86425591	1.19848371
1	2.50481844	0.78575581	2.07218361	1	-2.79048157	2.14475584	-2.46221638
1	3.22521853	-1.62554407	1.07768369	1	-2.13658166	0.51575583	-2.43061638
6	1.64121854	-1.07314420	-0.29041627	1	-3.72568178	0.84895581	-1.72591627
6	0.43921852	-0.77404422	0.63168371	1	-0.78548145	3.11565590	-1.31131625
6	-1.69408143	-2.07024407	-0.06041629	1	-0.18928146	2.44265580	0.20028371
6	-0.91288137	-0.77024418	-0.13091630	1	-0.01968145	1.53555584	-1.31101620
6	-1.91018140	0.28925583	0.36238372	6	1.67421854	-2.48094416	-0.87121630
6	-3.20718145	-0.47664419	0.54218370	1	2.53191853	-2.60214424	-1.53051627
8	-2.99558163	-1.81534410	0.29428372	1	0.76881850	-2.70034409	-1.43461621
8	-1.33088148	-3.18114424	-0.27401629	1	1.74911857	-3.23474407	-0.08441629
8	-4.28248167	-0.08664419	0.86098367	1	1.57251859	-0.35884419	-1.12141621
6	-2.06478167	1.57125592	-0.50601631	1	-0.45148143	-1.34284425	2.53118372
1	-1.62258148	0.61465579	1.37048376	1	1.29431856	-1.49074411	2.50258374
6	-2.92708158	2.59435582	0.23928370	1	0.29661855	-2.68714404	1.67548370
6	-0.68458152	2.18945599	-0.74121630				

tBuMA--ZB-rad--MA__S_re

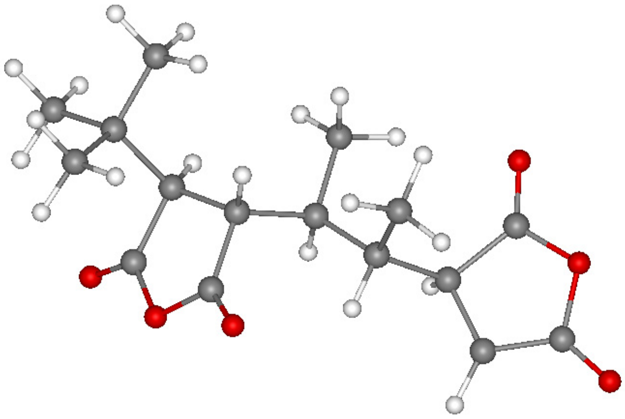

Zero-point vibrational energy	931938.3 (Joules/Mol)
	222.73859 (Kcal/Mol)
Zero-point correction=	0.354957 (Hartree/Particle)
Thermal correction to Energy=	0.376999
Thermal correction to Enthalpy=	0.377943
Thermal correction to Gibbs Free Energy=	0.302170
Sum of electronic and zero-point Energies=	-1073.333837
Sum of electronic and thermal Energies=	-1073.311795
Sum of electronic and thermal Enthalpies=	-1073.310851
Sum of electronic and thermal Free Energies=	-1073.386624

cartesian

6	4.17418861	0.78747201	0.58023953	6	-3.05731153	0.44707200	-0.16236044
6	3.26548839	-0.43252799	0.75063956	1	-1.66821170	0.51727200	1.48253953
6	4.21428871	-1.53832805	0.50613952	6	-4.23531151	0.66747200	0.79043955
6	5.51938820	-1.01732802	0.19993955	6	-2.65381169	1.80417192	-0.74436045
8	5.43678856	0.38067201	0.25493956	6	-3.48601151	-0.49182796	-1.29236042
8	3.90608835	1.93967187	0.70503956	1	-0.59361154	0.07917202	-1.15716040
8	6.54578829	-1.56882799	-0.06316046	6	0.73458844	1.53187203	0.76433957
1	2.93178844	-0.43822798	1.79823959	1	0.71138847	-0.46432799	1.53793955
1	3.99578834	-2.59582782	0.52883953	1	-5.07381153	1.10847199	0.24593955
1	1.85018849	-1.56362796	-0.33686048	1	-4.57951164	-0.26042798	1.24553955
6	2.00058842	-0.49612796	-0.16536047	1	-3.96841145	1.35367203	1.59863961
6	2.25678849	0.13047200	-1.53266048	1	-4.30981159	-0.04662797	-1.85436046
6	0.73048842	0.02037200	0.55123955	1	-2.68051171	-0.68472803	-2.00626063
1	1.43368840	-0.08162799	-2.21616054	1	-3.83841181	-1.45462799	-0.91366041
1	3.15368843	-0.29562798	-1.98926055	1	-3.51111174	2.26037216	-1.24456048
6	-0.62581152	-1.90312803	-0.41206044	1	-2.32441163	2.49367213	0.03683954
6	-0.57381159	-0.40712798	-0.18136047	1	-1.85471153	1.72997200	-1.48526049
6	-1.84891152	-0.14402798	0.63333952	1	2.39388847	1.21117198	-1.47836041

6	-2.17291164	-1.51122797	1.19903958	1	-0.07191157	1.85107195	1.42573953
8	-1.49131155	-2.47872782	0.48463953	1	0.61438847	2.06657219	-0.18026046
8	-0.03041154	-2.56352782	-1.20316041	1	1.67208838	1.86887205	1.20383954
8	-2.89131165	-1.81112802	2.09493947				

tBuMA--ZB-rad--MA__S_si

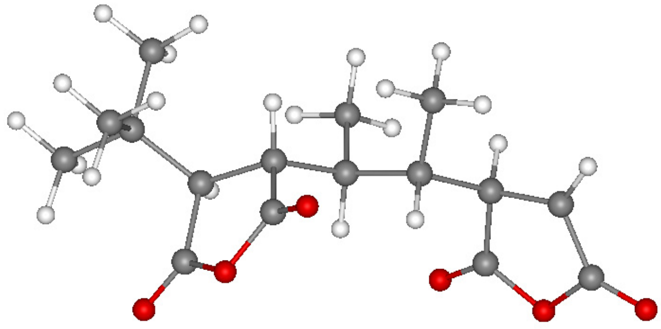

Zero-point vibrational energy	931355.7 (Joules/Mol)
	222.59936 (Kcal/Mol)
Zero-point correction=	0.354735 (Hartree/Particle)
Thermal correction to Energy=	0.376889
Thermal correction to Enthalpy=	0.377833
Thermal correction to Gibbs Free Energy=	0.302005
Sum of electronic and zero-point Energies=	-1073.335465
Sum of electronic and thermal Energies=	-1073.313311
Sum of electronic and thermal Enthalpies=	-1073.312367
Sum of electronic and thermal Free Energies=	-1073.388195

cartesian

6	5.42670965	-0.84553021	-0.33535117	6	-3.53109074	-0.00043020	-1.20995116
6	4.60120916	0.30956978	-0.57085115	1	-0.77469075	0.96006984	-0.75235116
6	3.38520908	0.24216980	0.26624882	6	0.68490928	0.96096975	1.52654886
6	3.62270927	-1.05403018	1.03934884	1	0.96780932	-1.00843024	0.77854884
8	4.78950930	-1.63333011	0.63154882	1	-5.03009081	-0.40063021	1.04464889
8	6.47390938	-1.16973019	-0.81005114	1	-4.17389059	-1.90463018	0.69244885
8	2.94580936	-1.54013014	1.88924873	1	-3.74059057	-0.95793021	2.11274862
1	4.88670921	1.08696985	-1.26225114	1	-4.50279045	0.49616981	-1.25425112
1	3.37270927	1.06256986	0.99694884	1	-2.85099077	0.57236975	-1.84645116
6	2.04880929	0.29356980	-0.53455120	1	-3.65529060	-0.99103022	-1.65465117
6	0.82000929	-0.01033020	0.35664880	1	-3.96009064	1.79196978	0.82004887
6	-0.39439070	-0.89843023	-1.69585121	1	-2.63509083	1.30696988	1.87424886
6	-0.51189065	-0.05233020	-0.44455120	1	-2.30059052	1.98896980	0.27814880
6	-1.64069068	-0.74833024	0.33104882	1	2.11370945	-0.49153021	-1.29285121

6	-1.64819062	-2.13973022	-0.27145118	6	1.95300925	1.63016987	-1.26725113
8	-0.97619069	-2.12143016	-1.48035121	1	2.79250908	1.76876998	-1.94965112
8	0.11750931	-0.63283020	-2.73685122	1	1.94420934	2.47776985	-0.57765120
8	-2.14099073	-3.13933015	0.13474882	1	1.05480933	1.67086983	-1.88155115
6	-3.04949093	-0.07963020	0.24084881	1	-0.19439071	0.73746985	2.13134861
1	-1.40029061	-0.86113024	1.39064884	1	0.59220928	1.99736977	1.19364882
6	-4.05279064	-0.88903022	1.06714880	1	1.54090929	0.88586980	2.19764876
6	-2.97039080	1.32926989	0.83424884				

TS__tBuMA--ZB-rad_ZB__RSR

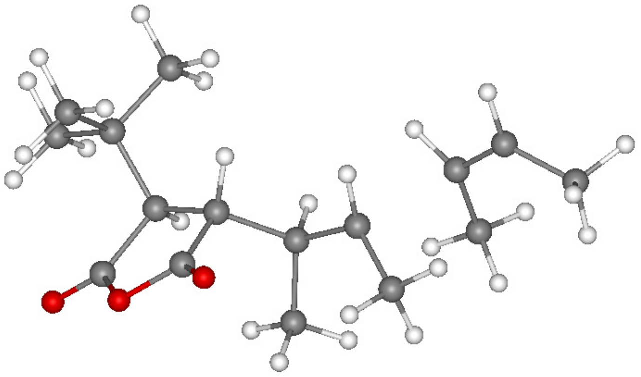

Zero-point vibrational energy	1059407.3 (Joules/Mol)
	253.20442 (Kcal/Mol)
Zero-point correction=	0.403507 (Hartree/Particle)
Thermal correction to Energy=	0.426085
Thermal correction to Enthalpy=	0.427029
Thermal correction to Gibbs Free Energy=	0.350887
Sum of electronic and zero-point Energies=	-851.151870
Sum of electronic and thermal Energies=	-851.129292
Sum of electronic and thermal Enthalpies=	-851.128348
Sum of electronic and thermal Free Energies=	-851.204489

cartesian

1	-2.17326951	1.57036519	-0.02289128	1	4.94283056	0.70796525	-0.79129124
6	-2.98786950	0.86856520	-0.18489128	1	3.88113046	1.88316524	-1.56249130
6	-3.96326947	0.84806526	0.77520871	1	4.19803047	1.44676518	2.14420867
6	-3.24246955	0.50206518	-1.62279129	1	2.86513042	0.32026520	2.35120869
6	-2.26126957	-2.00033474	0.42440870	1	4.34883070	-0.19903481	1.54220879
6	-1.49456966	-0.71883482	0.41890872	1	2.75453043	3.14676499	0.93440872
6	-0.27736959	-0.52613485	-0.45649132	1	1.74933040	2.65196514	-0.42589128
1	-1.66836953	-2.81763482	0.85220873	1	1.36013043	2.11506510	1.21240878
6	1.41033041	-1.90353477	0.84620875	1	-3.15976954	-1.90123475	1.03590870
6	1.03543043	-0.51543480	0.38410869	1	-1.38716960	-0.27273482	1.40450871

6	2.27173042	-0.02873480	-0.37679130	1	-2.57256961	-2.31543493	-0.57499129
6	3.08543038	-1.29383481	-0.56809127	1	0.66693044	-1.29803479	-2.26819134
8	2.57043028	-2.30253482	0.21860872	1	-1.08056962	-1.44173479	-2.25529146
8	0.84703040	-2.62083483	1.60790873	1	-0.11146960	-2.53943491	-1.28509128
8	4.03593063	-1.48553479	-1.25519121	1	-3.70006967	-0.48353481	-1.72849131
6	3.09463048	1.10266519	0.31350872	1	-2.32046962	0.50206518	-2.20519137
1	2.00613046	0.33706519	-1.37319124	1	-3.91706967	1.22336519	-2.09509134
6	4.25023031	1.52576518	-0.59719127	6	-5.29676914	0.18906519	0.66970873
6	2.18133044	2.31396508	0.52010870	1	-3.73456979	1.31006527	1.73310876
6	3.65153027	0.63456523	1.65970874	1	-5.44766951	-0.55513483	1.45990872
1	0.84463042	0.07966518	1.27780879	1	-5.44676924	-0.30953482	-0.28839129
6	-0.19266960	-1.50473475	-1.62629128	1	-6.10576916	0.91996527	0.78630877
1	4.80803061	2.34306502	-0.13309129	1	-0.31786957	0.49166521	-0.87529129

TS__tBuMA--ZB-rad_ZB__RSS

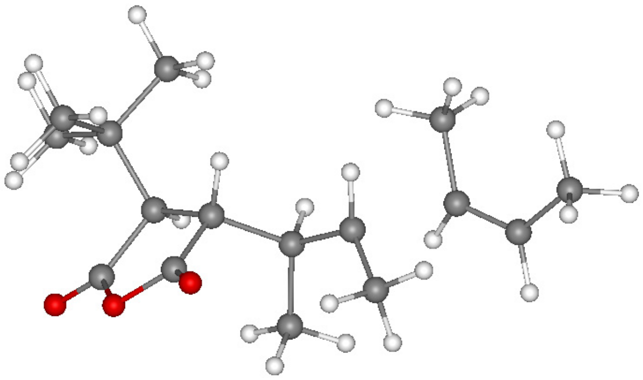

Zero-point vibrational energy	1059269.3 (Joules/Mol)
	253.17144 (Kcal/Mol)
Zero-point correction=	0.403454 (Hartree/Particle)
Thermal correction to Energy=	0.426039
Thermal correction to Enthalpy=	0.426983
Thermal correction to Gibbs Free Energy=	0.351048
Sum of electronic and zero-point Energies=	-851.153344
Sum of electronic and thermal Energies=	-851.130759
Sum of electronic and thermal Enthalpies=	-851.129815
Sum of electronic and thermal Free Energies=	-851.205751

cartesian

6	-2.75781322	1.80605865	-0.10552827	1	4.88328695	0.68095875	-0.88492823
6	-3.09531307	0.47885874	-0.73092824	1	3.81058693	1.87355876	-1.61192822
6	-4.30491304	-0.14364128	-0.58032823	1	4.18668699	1.33935869	2.07587171
1	-2.56661320	0.26245871	-1.65622818	1	2.85148692	0.21445873	2.27687168
6	-2.17131305	-2.10804129	0.57597178	1	4.31898689	-0.29164129	1.43077171

6	-1.54591298	-0.77084124	0.35067174	1	2.72548699	3.07275867	0.94397175
6	-0.31791303	-0.60854125	-0.50842828	1	1.70288706	2.62315869	-0.41912827
1	-1.51341295	-2.76524115	1.15477180	1	1.33098698	2.03695869	1.20667171
6	1.42588699	-1.96584129	0.76937175	1	-3.10401320	-2.00424123	1.13287175
6	1.00148702	-0.58464128	0.32787174	1	-1.55281305	-0.11894128	1.22277176
6	2.21298695	-0.06174129	-0.45122826	1	-2.40721321	-2.62104130	-0.35972828
6	3.02508688	-1.31454134	-0.71152824	1	0.59898698	-1.40064120	-2.32602835
8	2.55728698	-2.34294128	0.07837173	1	-1.15011299	-1.54634130	-2.27652836
8	0.92518699	-2.69374132	1.56407177	1	-0.15721300	-2.63254118	-1.31462824
8	3.94118690	-1.48314130	-1.44962823	1	-1.68371308	2.00225878	-0.12922826
6	3.05098677	1.04765880	0.25637174	1	-3.07651305	1.87135863	0.93617177
1	1.91608703	0.33815870	-1.42552829	1	-3.24291301	2.62485886	-0.64672822
6	4.19298697	1.49305880	-0.66082823	1	-4.51921320	-0.98324132	-1.23702824
6	2.14448690	2.25485873	0.51107174	6	-5.33651304	0.14425871	0.45717174
6	3.62808681	0.54205871	1.58037174	1	-5.52081299	-0.72724128	1.09627175
1	0.80588698	-0.00344127	1.22957182	1	-6.29971313	0.38725874	-0.00552827
6	-0.24761304	-1.60194123	-1.66532826	1	-5.06941319	0.97895873	1.10557175
1	4.75748682	2.29895878	-0.18492827	1	-0.35351303	0.40185872	-0.94162822

TS__tBuMA--ZB-rad_ZB__SRR

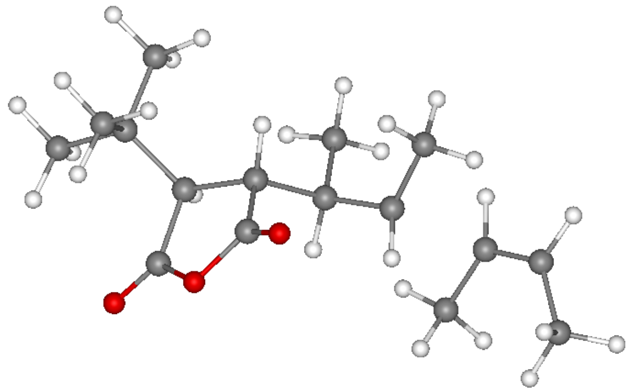

Zero-point vibrational energy	1059461.8 (Joules/Mol)
	253.21744 (Kcal/Mol)
Zero-point correction=	0.403528 (Hartree/Particle)
Thermal correction to Energy=	0.426202
Thermal correction to Enthalpy=	0.427146
Thermal correction to Gibbs Free Energy=	0.350935
Sum of electronic and zero-point Energies=	-851.151923
Sum of electronic and thermal Energies=	-851.129249
Sum of electronic and thermal Enthalpies=	-851.128305
Sum of electronic and thermal Free Energies=	-851.204515

cartesian							
1	3.31024361	1.37841964	0.69250876	1	-4.21885681	0.63941967	1.94940865
6	3.55544353	0.32011959	0.70160872	1	-4.78315687	0.02041960	-1.70409131
6	4.59454346	-0.05848038	-0.10229129	1	-3.11545658	-0.33758038	-2.12769127
6	3.16334343	-0.43578041	1.94510865	1	-4.05185652	-1.48278034	-1.15919137
1	1.92364359	-1.12738037	-0.64589125	1	-4.18845654	2.22121954	-0.64209127
6	1.68924356	-0.07018039	-0.56209129	1	-2.91475654	2.32291961	0.57080871
6	0.45824346	0.21721962	0.25910869	1	-2.51265645	1.98871970	-1.11689138
6	-0.77045655	-1.60638034	-1.01979125	6	1.93204355	0.70671964	-1.81619132
6	-0.86835647	-0.20498039	-0.46669129	1	2.88954353	0.43171963	-2.25989127
6	-2.06895661	-0.28858039	0.48470870	1	1.94334340	1.78791952	-1.65129137
6	-2.16825652	-1.77698040	0.75980872	1	1.16434348	0.49551961	-2.57209110
8	-1.45865643	-2.47208047	-0.19479130	1	-0.51005650	1.86351967	1.32400870
8	-0.20165652	-1.99998033	-1.98569131	1	0.23874348	2.33741975	-0.19509129
8	-2.75175643	-2.34788036	1.62340868	1	1.23744357	2.00141954	1.21730864
6	-3.41655660	0.29201958	-0.04759129	1	2.90064359	-1.47578037	1.73910868
1	-1.86345649	0.19841960	1.44130862	1	2.30944347	0.02511960	2.44270873
6	-4.50035667	0.13261959	1.02240872	1	3.98384356	-0.44678038	2.66930890
6	-3.23735642	1.78661966	-0.32529131	6	5.24274349	-1.40088034	-0.12079129
6	-3.85695648	-0.42058039	-1.32849133	1	4.93264341	0.64671957	-0.85729128
1	-1.02425647	0.47351959	-1.30589128	1	4.98094320	-1.96368039	-1.02589130
6	0.34714350	1.68391967	0.67310876	1	4.96614313	-2.01488042	0.73710871
1	-5.43545675	0.57701957	0.67240876	1	6.33364344	-1.31078041	-0.12449129
1	-4.69155693	-0.91218042	1.26390862	1	0.51194346	-0.39408040	1.16830862

TS__tBuMA--ZB-rad_ZB__SRS

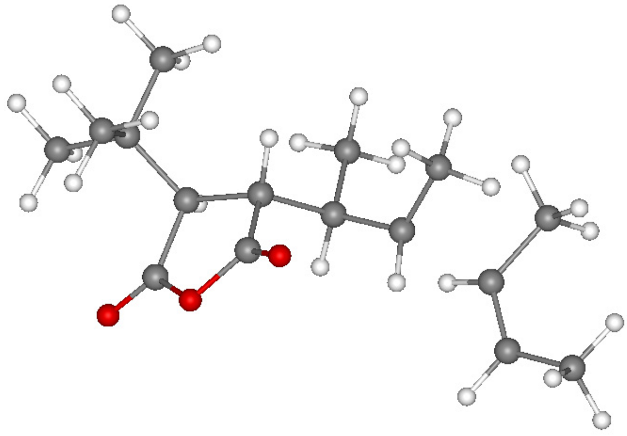

Zero-point vibrational energy	1059511.5 (Joules/Mol)
	1059511.5 (Kcal/Mol)
Zero-point correction=	0.403547 (Hartree/Particle)
Thermal correction to Energy=	0.426201
Thermal correction to Enthalpy=	0.427146

Thermal correction to Gibbs Free Energy= 0.350507
Sum of electronic and zero-point Energies= -851.150552
Sum of electronic and thermal Energies= -851.127897
Sum of electronic and thermal Enthalpies= -851.126953
Sum of electronic and thermal Free Energies= -851.203592

cartesian

6	4.05173254	1.21701527	0.41692609	1	-4.26266766	0.77441525	1.97182608
6	3.39093256	-0.01708481	0.96842611	1	-4.82486773	0.03961521	-1.66017389
6	3.99033284	-1.24898481	0.98772609	1	-3.17096734	-0.40358478	-2.05507398
1	2.66513276	0.16341519	1.75732613	1	-4.16186762	-1.47128475	-1.05297387
1	1.77773249	-1.36248481	-0.52197391	1	-4.13616753	2.25011539	-0.69127387
6	1.62913251	-0.28998479	-0.45827392	1	-2.88496733	2.34801531	0.54492611
6	0.40363255	0.09101522	0.33482608	1	-2.46196723	1.92711532	-1.11797392
6	-0.89636743	-1.73458481	-0.87577391	6	1.94013250	0.41861522	-1.73707390
6	-0.93486738	-0.30928481	-0.37867391	1	2.91263270	0.11121523	-2.12707400
6	-2.14476728	-0.30288479	0.56552613	1	1.95123255	1.50671518	-1.64107394
6	-2.31356740	-1.77338481	0.89622611	1	1.20533252	0.16261518	-2.51277399
8	-1.62866747	-2.53598499	-0.02457391	1	-0.51036739	1.82251513	1.30622613
8	-0.34136742	-2.19028473	-1.82227385	1	0.28023258	2.19551516	-0.21867390
8	-2.92936730	-2.28378487	1.77502608	1	1.24193251	1.88401520	1.22612607
6	-3.46056724	0.31611520	-0.00237392	1	3.34223270	2.03461504	0.28592607
1	-1.92496741	0.21101522	1.50452614	1	4.52623224	1.04491520	-0.54977387
6	-4.55996752	0.24751520	1.06102610	1	4.82883263	1.57121515	1.10212612
6	-3.21146727	1.78931510	-0.33587393	1	3.51613259	-2.02438474	1.58522606
6	-3.92106724	-0.42528480	-1.25987387	6	5.15673256	-1.68428481	0.16792609
1	-1.06026745	0.34001523	-1.24587393	1	4.86113262	-2.40998459	-0.60027391
6	0.34903255	1.57831526	0.67982608	1	5.90423250	-2.18918467	0.78852612
1	-5.46986771	0.72241521	0.68542612	1	5.65593243	-0.85588479	-0.33547392
1	-4.80276775	-0.77748477	1.33862615	1	0.43313256	-0.47728479	1.27492607

tBuMA--ZB--ZB-rad__RSR

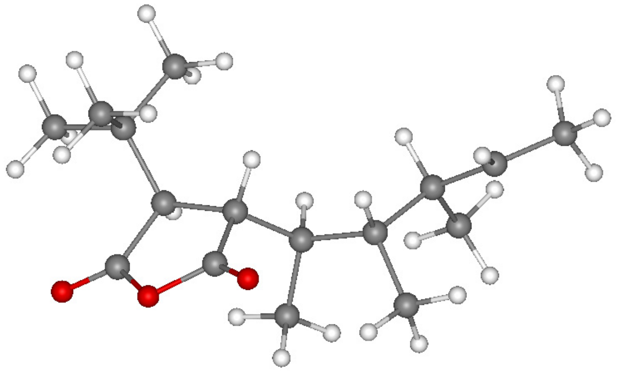

Zero-point vibrational energy	1067457.0 (Joules/Mol)
	255.12835 (Kcal/Mol)
Zero-point correction=	0.406573 (Hartree/Particle)
Thermal correction to Energy=	0.428864
Thermal correction to Enthalpy=	0.429808
Thermal correction to Gibbs Free Energy=	0.354077
Sum of electronic and zero-point Energies=	-851.191870
Sum of electronic and thermal Energies=	-851.169579
Sum of electronic and thermal Enthalpies=	-851.168635
Sum of electronic and thermal Free Energies=	-851.244365

cartesian

6	-2.67117405	0.29903913	-0.09148476	1	3.90812588	1.68233919	-1.66988480
6	-3.75697422	0.40003914	0.93171525	1	3.96312594	1.72623920	2.07441521
6	-3.21377420	0.11813912	-1.51078475	1	2.61482573	0.62953913	2.33231544
6	-2.22017407	-2.17616081	0.34081525	1	4.14952612	0.01823914	1.70091522
6	-1.62607396	-0.76946086	0.31351525	1	2.61572576	3.25003910	0.57641524
6	-0.31947407	-0.63046086	-0.52678478	1	1.71522593	2.59433913	-0.78958476
1	-1.52307403	-2.89026093	0.77921522	1	1.19622588	2.25573921	0.86581522
6	1.30622590	-1.76236093	1.06211531	1	-3.12487411	-2.18456078	0.95271522
6	0.92922592	-0.46666086	0.37941527	1	-2.49667406	-2.53836083	-0.65118474
6	2.20542598	-0.06306088	-0.36668473	1	0.77362597	-1.53696084	-2.18408465
6	3.02882576	-1.33556092	-0.36098474	1	-0.96207404	-1.76036084	-2.26748466
8	2.48882580	-2.23356104	0.53661525	1	-0.01627406	-2.71136093	-1.13398480
8	0.73072588	-2.36146092	1.91221523	1	-3.79787397	-0.80006087	-1.60508478
8	4.00352573	-1.61036086	-0.98248482	1	-2.40747428	0.08283913	-2.24658465
6	2.98722577	1.14703918	0.23171525	1	-3.86277437	0.94943917	-1.79258478
1	1.99672592	0.18183911	-1.41188478	6	-5.03017426	1.12713909	0.68891525
6	4.20592594	1.45413911	-0.64278477	1	-5.67177439	1.11583912	1.57111526
6	2.06972599	2.37303925	0.22081526	1	-5.60557413	0.69863915	-0.13928474
6	3.44812584	0.85573912	1.66161525	1	-4.86197424	2.18283916	0.42511523

1	0.67342591	0.24673915	1.16391528	1	-0.38077405	0.32693911	-1.05998480
6	-0.11777410	-1.71906090	-1.57878482	1	-1.36127400	-0.53566086	1.35101521
1	4.73402596	2.32633924	-0.24928476	1	-2.12977409	1.26583910	-0.08768475
1	4.90672588	0.62123913	-0.68008476	1	-3.49327421	0.15643913	1.95701528

tBuMA--ZB--ZB-rad__RSS

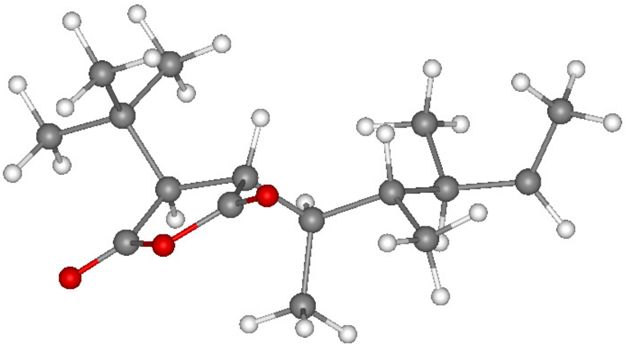

Zero-point vibrational energy	1067053.1 (Joules/Mol)
	255.03181 (Kcal/Mol)
Zero-point correction=	0.406419 (Hartree/Particle)
Thermal correction to Energy=	0.428932
Thermal correction to Enthalpy=	0.429876
Thermal correction to Gibbs Free Energy=	0.353089
Sum of electronic and zero-point Energies=	-851.191170
Sum of electronic and thermal Energies=	-851.168657
Sum of electronic and thermal Enthalpies=	-851.167713
Sum of electronic and thermal Free Energies=	-851.244500

cartesian

6	2.48444128	1.71184564	0.53091955	1	-3.86325884	1.69834566	1.55961955
6	2.80094123	0.21794558	0.69271952	1	-3.89155865	1.43534565	-2.17798042
6	4.21354103	-0.04965439	0.26961958	1	-2.53085876	0.33444560	-2.33408046
6	2.28294134	-2.09005451	-0.26688045	1	-4.06405878	-0.23735440	-1.66378045
6	1.75914121	-0.67105436	-0.05638043	1	-2.58805871	3.09784555	-0.78798044
6	0.38934121	-0.61475444	0.67431957	1	-1.67445874	2.56174564	0.62031955
1	1.54674125	-2.72255445	-0.76088047	1	-1.15225875	2.10924554	-1.00658047
6	-1.18785882	-1.89805436	-0.89168048	1	3.17484117	-2.07695436	-0.89528048
6	-0.83075881	-0.55925441	-0.28408042	1	2.56724119	-2.56175447	0.67721951
6	-2.12805891	-0.12555441	0.41091958	1	-0.68145883	-1.53865445	2.33561945
6	-2.92895865	-1.40945435	0.48801956	1	1.06334126	-1.65565443	2.45561957
8	-2.36515880	-2.35575438	-0.34128043	1	0.19484124	-2.69355440	1.33291960
8	-0.61285877	-2.53295445	-1.71538043	1	1.47274125	1.96844554	0.85361952
8	-3.90425873	-1.65795445	1.11971951	1	2.58104134	2.01864552	-0.51388043

6	-2.92385864	1.02094555	-0.28658044	1	3.17574120	2.32044554	1.11761951
1	-1.93905878	0.19974560	1.43791950	6	4.73864126	0.35674560	-1.06138051
6	-4.15235901	1.37674558	0.55531955	1	5.60864115	-0.23805441	-1.34818041
6	-2.02665877	2.25904560	-0.36968043	1	5.05984116	1.40864563	-1.08278048
6	-3.37125874	0.60774559	-1.69048047	1	3.99074125	0.25154561	-1.85498047
1	-0.56995881	0.10234559	-1.11148047	1	0.34554121	0.35274559	1.18531954
6	0.23004124	-1.68555439	1.75111949	1	1.61964118	-0.22495441	-1.04958045
1	-4.69635868	2.20104551	0.08741957	1	2.72654128	-0.03245440	1.75881958
1	-4.83595896	0.53544557	0.66221952	1	4.89284134	-0.51505440	0.97431958

tBuMA--ZB--ZB-rad__SRR

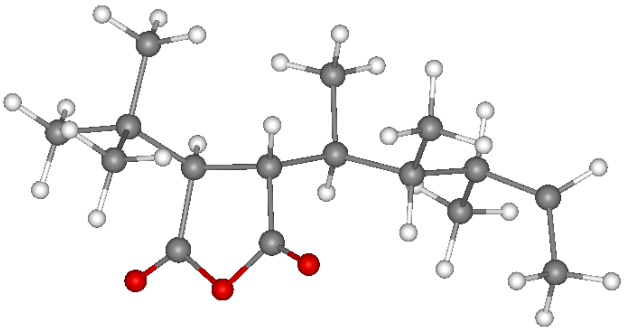

Zero-point vibrational energy	1068496.3 (Joules/Mol)
	255.37675 (Kcal/Mol)
Zero-point correction=	0.406969 (Hartree/Particle)
Thermal correction to Energy=	0.429269
Thermal correction to Enthalpy=	0.430213
Thermal correction to Gibbs Free Energy=	0.355556
Sum of electronic and zero-point Energies=	-851.193241
Sum of electronic and thermal Energies=	-851.170941
Sum of electronic and thermal Enthalpies=	-851.169997
Sum of electronic and thermal Free Energies=	-851.244653

cartesian

6	-3.11681080	0.37305215	-0.57323474	1	2.80838919	-0.66934788	2.13656521
6	-4.39341068	-0.12364785	0.04396523	1	3.78958917	-1.69454777	1.08196521
6	-3.03431082	-0.09824786	-2.03543472	1	4.05138922	2.03375196	1.14986527
6	-1.88011086	-0.03564784	0.26486522	1	2.90098906	2.36555195	-0.14273477
6	-0.54021084	0.31705216	-0.42103478	1	2.33218908	1.79785216	1.43046522
6	0.56438923	-1.68184781	0.69696528	6	-1.99121082	0.52905214	1.67996526
6	0.70558918	-0.21714786	0.34356523	1	-2.94161081	0.23635218	2.12926507
6	1.97858930	-0.19994785	-0.51653475	1	-1.96141064	1.62195218	1.68676531
6	2.06758928	-1.63344777	-1.00143480	1	-1.20091081	0.15495214	2.33176517
8	1.28278923	-2.43894792	-0.20343477	1	0.53728920	2.04985213	-1.20803475

8	-0.04961078	-2.19624782	1.57516527	1	-0.34821078	2.37415195	0.27526522
8	2.69568920	-2.09074783	-1.90013468	1	-1.20631075	2.21135211	-1.25483477
6	3.29068923	0.24695215	0.20166524	1	-2.86141086	-1.17644787	-2.09483480
1	1.86388922	0.42915216	-1.40143478	1	-2.23531079	0.39855215	-2.59133482
6	4.45518923	0.21025214	-0.79213476	1	-3.96951103	0.11345214	-2.55813479
6	3.12378907	1.68925226	0.68646526	6	-4.62071085	-1.55284786	0.38886523
6	3.60838914	-0.66194785	1.39156520	1	-5.59851074	-1.69924784	0.85016525
1	0.81098926	0.34045216	1.27446520	1	-3.87001085	-1.94594777	1.08386528
6	-0.37911078	1.81685221	-0.66323471	1	-4.58331108	-2.20734787	-0.49453476
1	5.36948919	0.55905211	-0.30533478	1	-0.53491080	-0.18434785	-1.39623475
1	4.63828897	-0.79204786	-1.17773473	1	-1.91081083	-1.12674785	0.34626523
1	4.26558924	0.86375213	-1.64793479	1	-3.17391086	1.46825218	-0.58213472
1	4.51228905	-0.31404784	1.89646530	1	-5.25141096	0.53985214	0.01566523

tBuMA--ZB--ZB-rad__SRS

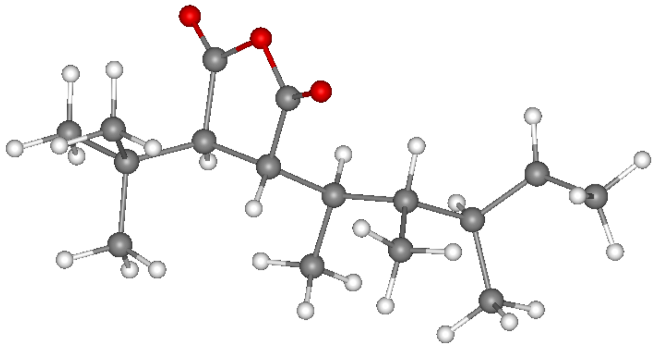

Zero-point vibrational energy	1069515.3 (Joules/Mol)
	255.62029 (Kcal/Mol)
Zero-point correction=	0.407357 (Hartree/Particle)
Thermal correction to Energy=	0.429429
Thermal correction to Enthalpy=	0.430373
Thermal correction to Gibbs Free Energy=	0.355935
Sum of electronic and zero-point Energies=	-851.188278
Sum of electronic and thermal Energies=	-851.166206
Sum of electronic and thermal Enthalpies=	-851.165262
Sum of electronic and thermal Free Energies=	-851.239700

cartesian

6	3.72677398	1.24896741	-0.52688044	1	-2.90952611	-0.64443266	2.08911967
6	3.00027394	-0.06253264	-0.82698047	1	-3.99512601	-1.58993268	1.06241953
6	3.93567419	-1.23623264	-0.82238048	1	-3.99032593	2.15026736	1.12451959
6	1.76127386	-0.35043260	0.09471957	1	-2.83462596	2.39506745	-0.18208042
6	0.43377393	0.10176738	-0.55988044	1	-2.29132605	1.78676724	1.38581955

6	-0.79112601	-1.80213261	0.60421956	6	1.94907403	0.15446737	1.52271962
6	-0.82902604	-0.33313262	0.24031956	1	2.90347385	-0.18863262	1.92701960
6	-2.12112594	-0.22763263	-0.58408046	1	1.94167399	1.24436736	1.59471953
6	-2.32392597	-1.65243268	-1.06038046	1	1.17467391	-0.24053262	2.18121958
8	-1.58002603	-2.50933266	-0.27708045	1	-0.58082604	1.90026736	-1.27688038
8	-0.20532602	-2.35483265	1.47831964	1	0.49137396	2.18466735	0.08631957
8	-3.00492597	-2.06613255	-1.94158041	1	1.14977396	1.90706730	-1.52808046
6	-3.37822604	0.31116739	0.16981958	1	3.06487393	2.11366725	-0.58048046
1	-1.98902607	0.38906738	-1.47518039	1	4.17637396	1.25126731	0.46741953
6	-4.56832600	0.35916737	-0.79268050	1	4.53167391	1.40576732	-1.24888039
6	-3.09682608	1.73716736	0.65031952	6	5.11277390	-1.37913263	0.07411957
6	-3.72802615	-0.57493263	1.36761963	1	4.82837391	-1.61403263	1.11201954
1	-0.86552602	0.23826739	1.16811955	1	5.76247406	-2.19193268	-0.25708041
6	0.36947393	1.60326743	-0.83028048	1	5.71967411	-0.47053263	0.12031958
1	-5.44332600	0.76606739	-0.27978042	1	0.38257396	-0.41163263	-1.53058040
1	-4.82922602	-0.62623262	-1.17718041	1	1.70137393	-1.44023263	0.16451958
1	-4.35802603	1.00336730	-1.65078044	1	2.60647392	0.00836739	-1.85108042
1	-4.58832598	-0.16163263	1.89881957	1	3.57817388	-2.13163280	-1.32428038

S10. MA – IB reaction profile

TS__tBuMA-rad_IB

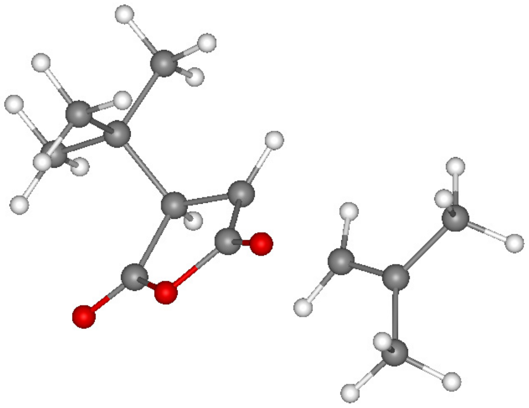

Zero-point vibrational energy	760607.4 (Joules/Mol)
	181.78954 (Kcal/Mol)
Zero-point correction=	0.289700 (Hartree/Particle)
Thermal correction to Energy=	0.306817
Thermal correction to Enthalpy=	0.307762
Thermal correction to Gibbs Free Energy=	0.243534
Sum of electronic and zero-point Energies=	-694.042206
Sum of electronic and thermal Energies=	-694.025088
Sum of electronic and thermal Enthalpies=	-694.024144
Sum of electronic and thermal Free Energies=	-694.088372

cartesian

6	3.06560612	0.13537940	0.52618235	1	-3.20689392	2.26587939	-0.44851765
6	1.86210585	0.29537937	1.14968240	1	-1.96619415	2.25277948	0.80298233
6	0.65190589	-1.15352058	-1.05001771	1	-1.51219416	2.16997957	-0.90581763
6	0.20940590	0.05087939	-0.38961765	1	-3.58909392	0.19717941	-1.88311768
6	-0.99869406	-0.26772061	0.42578235	1	-1.87159407	-0.00802061	-2.21721768
6	-0.96579409	-1.79232061	0.42158237	1	-2.80039406	-1.32572067	-1.48961759
8	-0.03949409	-2.23402047	-0.48381764	1	-4.42629385	0.34537938	0.50558233
8	1.47480583	-1.33332062	-1.89981759	1	-3.58769393	-1.14362061	0.96238232
8	-1.61379409	-2.55292058	1.06938231	1	-3.29119396	0.34537938	1.85728240
6	-2.35369396	0.30807939	-0.10831765	6	3.70720601	1.25247931	-0.22791764
1	-0.90969414	0.06307939	1.46728241	1	3.71100593	1.02967930	-1.30081761
6	-3.47719407	-0.06472063	0.86058235	1	4.75380611	1.37837934	0.06668235
6	-2.24359393	1.83297932	-0.16811764	1	3.19420600	2.20357943	-0.07911765
6	-2.66409397	-0.24132061	-1.50131762	6	3.74430609	-1.19232059	0.46378237
1	0.39700592	1.00287938	-0.86171764	1	4.77360582	-1.11892056	0.83078235
1	1.47060585	1.28947937	1.33678234	1	3.80040598	-1.54672062	-0.57011765
1	1.47590590	-0.49292061	1.78758240	1	3.22570610	-1.95072067	1.05068231

TS__tBuMA-rad_IB___syn

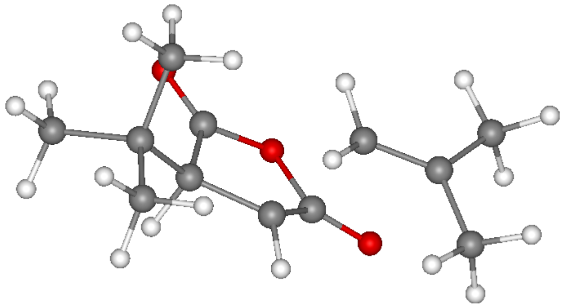

Zero-point vibrational energy	760933.0 (Joules/Mol)
	181.86735 (Kcal/Mol)
Zero-point correction=	0.289824 (Hartree/Particle)
Thermal correction to Energy=	0.306721
Thermal correction to Enthalpy=	0.307665
Thermal correction to Gibbs Free Energy=	0.244978
Sum of electronic and zero-point Energies=	-694.035002
Sum of electronic and thermal Energies=	-694.018105
Sum of electronic and thermal Enthalpies=	-694.017161
Sum of electronic and thermal Free Energies=	-694.079848

cartesian

6	2.76072645	-0.34745592	-0.44701764	6	3.51432657	0.75124407	-1.11911774
6	1.43162656	-0.56135589	-0.69011760	1	4.38832664	0.35074410	-1.64481771
6	0.90642649	1.77474403	0.83218241	1	3.88622665	1.47184408	-0.38501766
6	0.23432651	0.49674410	0.93298239	1	2.90182662	1.29254413	-1.84041762
6	-1.21457350	0.75224411	0.62858236	1	-1.65557349	1.05594409	1.59668231
6	-1.13177347	2.06014419	-0.14551765	6	-3.61457348	0.15664411	0.27888235
8	0.08412650	2.64034414	0.09738235	6	-1.97697341	-1.60905600	0.90428239
8	1.97442651	2.14054418	1.22638237	6	-1.95917344	-0.63395590	-1.40231764
8	-1.94147348	2.58264399	-0.84501761	1	-4.32147360	-0.60025591	-0.06971765
6	-2.17467356	-0.33405590	0.08228235	1	-3.79957342	1.07664418	-0.27461764
1	0.54722649	-0.18105590	1.71428227	1	-3.82867336	0.34454411	1.33538234
1	0.96582651	-1.48465586	-0.36611766	1	-2.69797349	-2.37115598	0.59888238
1	0.95142651	-0.05085588	-1.51711774	1	-2.12657356	-1.42595601	1.97238231
6	3.50252652	-1.14105582	0.57628238	1	-0.97827351	-2.03245592	0.77418238
1	3.76962662	-0.50075585	1.42448235	1	-2.70697355	-1.35525584	-1.74171770
1	4.44252634	-1.52995586	0.17248236	1	-0.98097354	-1.07015586	-1.60241771
1	2.91942644	-1.98215604	0.95438236	1	-2.07167339	0.26404411	-2.01131773

TS__tBuMA-rad_to_IB_transfer

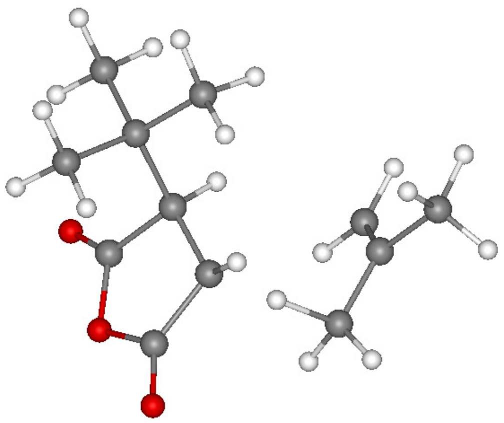

Zero-point vibrational energy	748413.0 (Joules/Mol)
	178.87500 (Kcal/Mol)
Zero-point correction=	0.285055 (Hartree/Particle)
Thermal correction to Energy=	0.302179
Thermal correction to Enthalpy=	0.303123
Thermal correction to Gibbs Free Energy=	0.238235
Sum of electronic and zero-point Energies=	-694.029191
Sum of electronic and thermal Energies=	-694.012068
Sum of electronic and thermal Enthalpies=	-694.011123
Sum of electronic and thermal Free Energies=	-694.076011

cartesian

6	2.39833522	1.53304410	-0.05886474	6	-1.09936476	-1.67465568	1.24813521
6	2.93343520	0.22914422	-0.42096472	6	-2.99786472	-0.12375581	0.74453527
6	3.42053533	-0.64405578	0.69953525	1	-0.07446472	0.90264422	1.72653532
6	2.95343518	-0.18695581	-1.70256472	1	3.33463526	-1.16395569	-1.97686470
1	1.14963520	1.36624420	0.33313525	1	2.60463524	0.44694421	-2.51046467
1	2.62903523	-0.82155579	1.43433523	1	-1.77366471	-2.47785568	1.55513525
1	4.24463511	-0.16515577	1.23593521	1	-0.18076472	-2.13965583	0.87953532
6	-0.77676469	2.49584436	0.43063527	1	-0.85406470	-1.10235572	2.14553523
6	-0.18256472	1.18204427	0.68503529	1	-3.69866467	-0.86445576	1.13663530
6	-0.72576469	0.23184422	-0.34386474	1	-2.74236488	0.55044419	1.56633532
6	-1.32046473	1.19644427	-1.36166477	1	-3.52996469	0.45554420	-0.01446474
8	-1.36606479	2.46194410	-0.83346474	1	-2.89026475	-2.47755575	-0.64056474
8	-0.77536470	3.49314427	1.08313525	1	-2.63746476	-1.17065573	-1.80446470
8	-1.71346474	0.97254419	-2.46226478	1	-1.31236482	-2.26005578	-1.40216470
6	-1.76336479	-0.81825584	0.16753528	1	2.27073526	2.24254417	-0.87416476
1	0.07273526	-0.32525581	-0.84906477	1	3.76863527	-1.61215568	0.33913529
6	-2.17536473	-1.72875571	-0.99126476	1	2.81003523	1.99674416	0.83633530

tBuMA--IB-rad

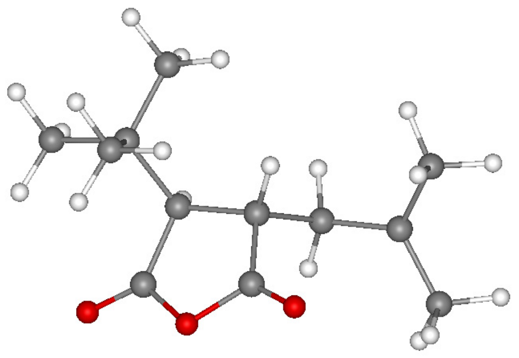

Zero-point vibrational energy	768357.4 (Joules/Mol)
	183.64183 (Kcal/Mol)
Zero-point correction=	0.292652 (Hartree/Particle)
Thermal correction to Energy=	0.309710
Thermal correction to Enthalpy=	0.310654
Thermal correction to Gibbs Free Energy=	0.246936
Sum of electronic and zero-point Energies=	-694.074378
Sum of electronic and thermal Energies=	-694.057320
Sum of electronic and thermal Enthalpies=	-694.056375
Sum of electronic and thermal Free Energies=	-694.120093

cartesian

6	2.86745310	0.14671767	0.47930294	1	-4.25904703	0.75701761	0.19230296
6	1.47975314	-0.00828236	1.00000298	1	-3.77504683	-0.89888239	0.57680291
6	0.64275301	-1.56608236	-0.79839706	1	-3.37364697	0.41441762	1.67970300
6	0.38575307	-0.26908237	-0.07409705	1	-3.10434699	0.64151764	-2.05189705
6	-1.00994694	-0.44588235	0.52870291	1	-1.41324687	0.18011767	-2.17919707
6	-1.24844694	-1.93958235	0.40730295	1	-2.62944698	-1.01998234	-1.72449696
8	-0.29784694	-2.49958229	-0.42019707	1	-2.61784697	2.48731756	-0.38439706
8	1.50665307	-1.83648241	-1.56829703	1	-1.64404690	2.15901756	1.04760301
8	-2.09574699	-2.61118221	0.89990294	1	-0.90244699	2.14881754	-0.55719703
6	-2.15144682	0.39101762	-0.12409705	6	3.16125298	1.26591766	-0.45809707
1	-1.00894690	-0.20958236	1.59760296	1	3.02455306	0.96391761	-1.50739706
6	-3.46404696	0.14391762	0.62420297	1	4.19835329	1.60291755	-0.36909705
6	-1.79964685	1.87571776	0.00300295	1	2.51525307	2.13211775	-0.28479704
6	-2.32644701	0.02281767	-1.59889698	6	3.86875319	-0.94428235	0.62910295
1	0.43625307	0.53391767	-0.81009704	1	4.88085318	-0.54368234	0.74720293
1	1.16765308	0.91161764	1.51410306	1	3.89185309	-1.59718239	-0.25489706
1	1.43585312	-0.80798233	1.74680305	1	3.65865302	-1.58108234	1.49190295

TS__tBuMA--IB-rad_MA__re

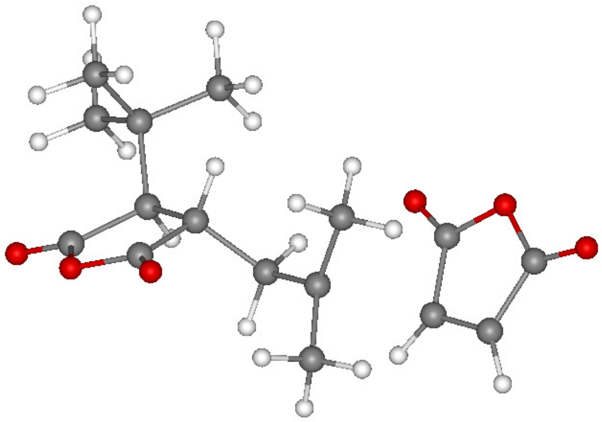

Zero-point vibrational energy	922809.1 (Joules/Mol)
	220.55667 (Kcal/Mol)
Zero-point correction=	0.351479 (Hartree/Particle)
Thermal correction to Energy=	0.373801
Thermal correction to Enthalpy=	0.374746
Thermal correction to Gibbs Free Energy=	0.298131
Sum of electronic and zero-point Energies=	-1073.307555
Sum of electronic and thermal Energies=	-1073.285233
Sum of electronic and thermal Enthalpies=	-1073.284289
Sum of electronic and thermal Free Energies=	-1073.360904

cartesian

6	-3.10463047	-1.16314888	0.63466042	6	2.63656950	-1.38784885	-0.23553956
6	-3.29403043	0.15135115	1.29016042	1	1.77086961	-0.50154883	1.52106047
6	-4.50643063	0.62255114	0.88906044	6	3.68646955	-2.08954906	0.63036042
6	-5.03423023	-0.28144884	-0.12853956	6	1.48956966	-2.36884880	-0.49163952
8	-4.13063049	-1.34634888	-0.25203955	6	3.26926947	-0.96624887	-1.56353962
8	-2.23483038	-1.97124875	0.78006041	1	0.68366969	0.16505116	-1.12813962
8	-6.02923059	-0.23574886	-0.78123957	1	-0.60263032	-0.45004886	0.88666046
1	-2.73213053	0.43285117	2.16756034	1	-0.15883033	1.06325114	1.66786039
1	-5.04043055	1.50215113	1.21366048	1	4.06196976	-2.97494888	0.11166045
6	-1.92693031	2.64655113	0.49736047	1	4.53276968	-1.44254887	0.85656041
6	-1.56363034	1.26615119	0.06616045	1	3.25586963	-2.42134905	1.57916045
6	-1.91083026	0.90085113	-1.33703959	1	3.62966967	-1.84484887	-2.10283947
6	-0.37333032	0.60415113	0.69716042	1	2.56036949	-0.45754886	-2.22293949
1	-1.29263031	1.49075115	-2.02663946	1	4.12706947	-0.30554885	-1.41343951
1	-2.95003033	1.14465117	-1.57013953	1	1.87636960	-3.27464867	-0.96413958
6	1.45986974	1.98025119	-0.40563953	1	0.99696970	-2.66744900	0.43676046
6	0.91966975	0.59275115	-0.15213954	1	0.72226965	-1.96614873	-1.15653956
6	2.07326961	-0.15184885	0.52816045	1	-2.94793034	2.90345097	0.20746046

6	3.11306953	0.92965114	0.75116044	1	-1.81973028	2.78995132	1.57496047
8	2.70436954	2.10825109	0.14906046	1	-1.26023030	3.36455107	0.00476045
8	0.94186974	2.88245106	-0.98463953	1	-1.74313033	-0.15484884	-1.55983961
8	4.14456940	0.88215113	1.33516037				

TS__tBuMA--IB-rad_MA__si

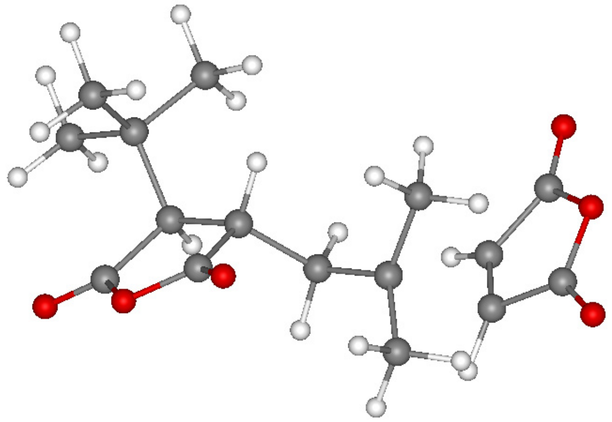

Zero-point vibrational energy 922944.6 (Joules/Mol)

220.58907 (Kcal/Mol)

Zero-point correction= 0.351531 (Hartree/Particle)

Thermal correction to Energy= 0.373827

Thermal correction to Enthalpy= 0.374771

Thermal correction to Gibbs Free Energy= 0.298256

Sum of electronic and zero-point Energies= -1073.304656

Sum of electronic and thermal Energies= -1073.282360

Sum of electronic and thermal Enthalpies= -1073.281416

Sum of electronic and thermal Free Energies= -1073.357931

cartesian

6	4.06846523	1.62311864	0.28266048	6	-3.37593484	0.68551862	-1.45253944
6	3.23206520	1.03091860	1.32276046	1	-0.63923490	-0.18058142	-1.16573954
6	3.37276530	-0.32448143	1.26536047	1	0.78156507	0.48111862	0.71526045
6	4.45966530	-0.59928143	0.29516047	1	0.35156509	-0.96658140	1.61706054
8	4.78416538	0.58571857	-0.32003951	1	-4.16873503	2.79351854	0.09586047
8	4.20216513	2.75571847	-0.06313954	1	-4.43743467	1.31861854	1.03066051
8	5.00076532	-1.62658143	0.02256047	1	-3.18313479	2.45521855	1.51916051
1	2.63026524	1.63001859	1.98906052	1	-3.85193491	1.47601855	-2.03643966
1	3.09946513	-1.04688144	2.01916051	1	-2.69473481	0.15801857	-2.12603951
6	1.66556513	-1.29718137	-0.04473954	1	-4.16223478	-0.01248142	-1.15373945
6	0.52026510	-0.57728142	0.60766047	1	-2.12043476	3.13501859	-1.22773945
6	-1.38823497	-1.97108138	-0.34843951	1	-1.08153498	2.74651837	0.13996047
6	-0.82053494	-0.58198142	-0.16813952	1	-0.88653493	1.89861858	-1.39893949

6	-1.92333496	0.17961858	0.57526046	6	1.93466508	-2.71908164	0.31446046
6	-2.86013484	-0.92288142	1.03096044	1	2.97656512	-2.98548150	0.12476047
8	-2.52373481	-2.11088157	0.40686047	1	1.31226516	-3.37728143	-0.30303952
8	-0.97293496	-2.86168146	-1.01833951	1	1.69916511	-2.93578148	1.35896051
8	-3.77003479	-0.87638140	1.79096055	6	2.00816512	-0.87868142	-1.43583953
6	-2.65673470	1.28561854	-0.24253953	1	2.97836518	-1.26138139	-1.75463951
1	-1.53173494	0.65671861	1.47926056	1	1.99706507	0.20701857	-1.56253946
6	-3.67243481	1.99691856	0.65536046	1	1.26896513	-1.30028141	-2.13003969
6	-1.62223494	2.31461859	-0.70653957				

tBuMA--IB-rad--MA__re

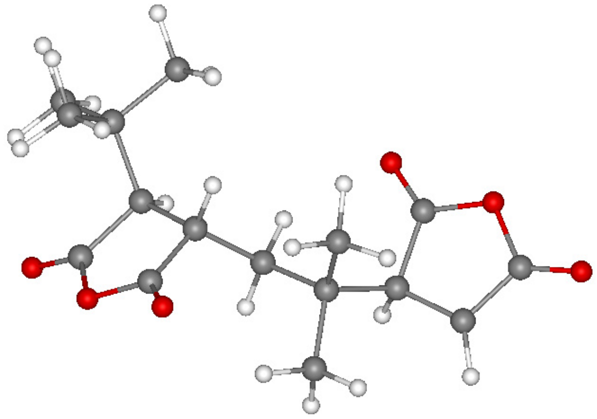

Zero-point vibrational energy	931038.8 (Joules/Mol)
	222.52361 (Kcal/Mol)
Zero-point correction=	0.354614 (Hartree/Particle)
Thermal correction to Energy=	0.376526
Thermal correction to Enthalpy=	0.377470
Thermal correction to Gibbs Free Energy=	0.302280
Sum of electronic and zero-point Energies=	-1073.337826
Sum of electronic and thermal Energies=	-1073.315914
Sum of electronic and thermal Enthalpies=	-1073.314970
Sum of electronic and thermal Free Energies=	-1073.390160

cartesian

8	4.40955544	1.40446281	0.06982557	1	-0.56694412	-0.00373717	-1.09757435
6	3.15315580	1.17656279	0.55062556	1	0.66195583	0.61606282	0.94212556
6	2.99125600	-0.30213720	0.90382558	1	0.30795586	-0.93053716	1.68012559
6	4.34895563	-0.81603718	0.63652557	1	-4.34244442	2.68716288	0.11882557
6	5.18165588	0.23596284	0.11612557	1	-4.50524426	1.20226288	1.06222558
1	4.72475576	-1.81153727	0.81422555	1	-3.32854414	2.42526269	1.53812563
8	2.35385585	2.05486274	0.64452553	1	-3.92044425	1.41026282	-2.02277446
1	2.75265598	-0.37203717	1.97472560	1	-2.69964409	0.14986283	-2.09807444

6	1.81745589	-1.01203716	0.13182557	1	-4.16554451	-0.08893718	-1.13767433
6	0.50445586	-0.44013718	0.71882558	1	-2.31734419	3.18046284	-1.16947436
6	-1.31854415	-1.85233712	-0.41517442	1	-1.21994412	2.81566286	0.16112557
6	-0.77874410	-0.46773717	-0.13457443	1	-1.02394414	2.01606274	-1.39947438
6	-1.93614411	0.22346282	0.60152555	6	1.92985594	-2.51483727	0.39832556
6	-2.80704403	-0.93653715	1.03702557	1	2.84175587	-2.93033719	-0.03607443
8	-2.43184400	-2.07973719	0.35222557	1	1.09495592	-3.04953718	-0.05037443
8	-0.91024411	-2.68053722	-1.16677439	1	1.93665588	-2.73103714	1.47072566
8	-3.69864392	-0.96703720	1.81962562	6	1.95115590	-0.74363720	-1.36757433
6	-2.73164415	1.28466272	-0.21917443	1	2.93645597	-1.04783726	-1.73057437
1	-1.58384407	0.72036284	1.50982559	1	1.81695592	0.31286281	-1.61497438
6	-3.78984427	1.92946267	0.67982554	1	1.21205592	-1.32183719	-1.92317438
6	-1.76224399	2.37686276	-0.68007445	8	6.32275581	0.23186281	-0.23707442
6	-3.41144419	0.64566284	-1.43207443				

tBuMA--IB-rad--MA__si

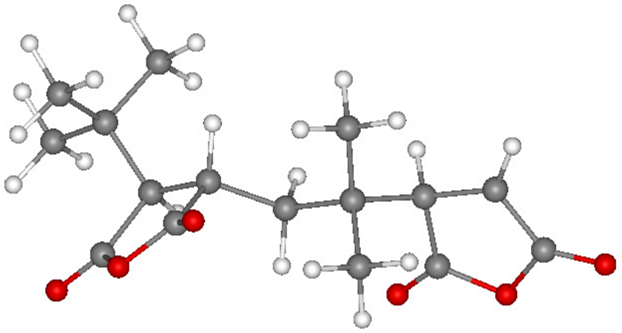

Zero-point vibrational energy	930984.8 (Joules/Mol)
	222.51072 (Kcal/Mol)
Zero-point correction=	0.354593 (Hartree/Particle)
Thermal correction to Energy=	0.376501
Thermal correction to Enthalpy=	0.377445
Thermal correction to Gibbs Free Energy=	0.302736
Sum of electronic and zero-point Energies=	-1073.336349
Sum of electronic and thermal Energies=	-1073.314441
Sum of electronic and thermal Enthalpies=	-1073.313497
Sum of electronic and thermal Free Energies=	-1073.388206

cartesian

6	-5.37102795	-0.18508139	0.34453487	6	3.71027184	0.19531859	-0.94786519
6	-4.35662794	-0.00188141	-0.66086513	1	0.84677196	0.48571861	-1.03816509
6	-3.04432797	-0.43618140	-0.14146513	1	-0.63042808	-1.08168149	0.13973486
6	-3.41952801	-0.92688143	1.25723481	1	-0.72342801	-0.05038141	1.52983499
8	-4.75452805	-0.72978139	1.47613478	1	4.67607212	-2.20958138	-0.08586514

8	-6.54332781	0.04451860	0.32363486	1	4.45757198	-1.18448138	1.33663487
8	-2.71672797	-1.41768146	2.08183479	1	3.42007208	-2.58738136	1.09493494
1	-4.58712816	0.37761861	-1.64426517	1	4.43997192	-0.23368141	-1.63796520
1	-2.67542791	-1.30428147	-0.70656508	1	3.05187201	0.83861864	-1.53836513
6	-1.90052807	0.63971859	-0.15646511	1	4.26277208	0.82921863	-0.24936515
6	-0.63822806	-0.02928141	0.44143486	1	3.01007199	-2.30548143	-1.88596511
6	1.05547190	1.91371858	0.48983487	1	1.69427192	-2.62228131	-0.75816518
6	0.75637197	0.49711859	0.04843485	1	1.58077204	-1.28538144	-1.91066527
6	1.86077201	-0.33118141	0.72253489	6	-2.35042810	1.84961855	0.66673487
6	2.44077206	0.62361860	1.74663496	1	-3.28332806	2.26341867	0.27393487
8	1.96907198	1.90241861	1.51023483	1	-1.60692811	2.64341879	0.63513488
8	0.62467194	2.93791866	0.06213486	1	-2.51702785	1.58481860	1.71443486
8	3.19377208	0.41311860	2.63883471	6	-1.67072809	1.05451858	-1.61026502
6	2.94867206	-0.91888142	-0.22616512	1	-2.57782793	1.46751857	-2.05506516
1	1.43417192	-1.17008138	1.28053486	1	-1.34952807	0.21081859	-2.22946525
6	3.93227196	-1.76858139	0.58233488	1	-0.91112804	1.83461857	-1.66496515
6	2.26167202	-1.82728148	-1.24976516				

TS__tBuMA--IB-rad_IB

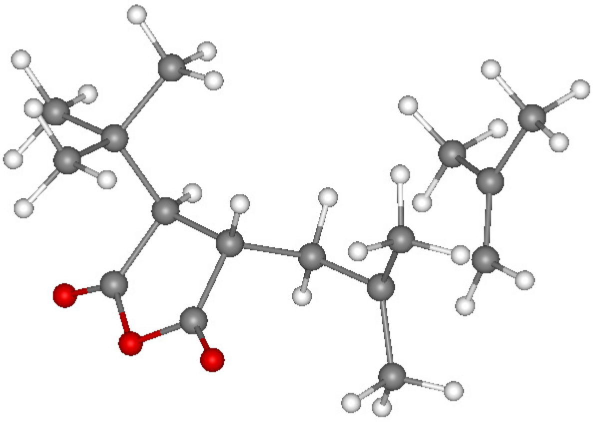

Zero-point vibrational energy	1059454.6 (Joules/Mol)
	253.21572 (Kcal/Mol)
Zero-point correction=	0.403525 (Hartree/Particle)
Thermal correction to Energy=	0.425968
Thermal correction to Enthalpy=	0.426912
Thermal correction to Gibbs Free Energy=	0.351203
Sum of electronic and zero-point Energies=	-851.166973
Sum of electronic and thermal Energies=	-851.144530
Sum of electronic and thermal Enthalpies=	-851.143585
Sum of electronic and thermal Free Energies=	-851.219295

cartesian

6	4.43949986	-1.71179998	-0.71179998	1	1.08690000	-0.69700003	0.27900001
6	3.80019999	-1.11650002	0.50349998	1	0.69379997	0.57900000	1.41589999
6	3.70580006	0.23370001	0.68159997	1	-4.00330019	-2.90599990	-0.32720000
1	4.30889988	0.89800000	0.07140000	1	-4.08190012	-1.67180002	0.93480003
6	3.24810004	-2.08949995	1.49890006	1	-2.81890011	-2.89829993	0.98079997
1	3.41709995	0.62669998	1.65139997	1	-3.93149996	-1.15890002	-2.14310002
6	2.08890009	2.58649993	0.26879999	1	-2.74099994	0.13349999	-2.11710000
6	1.89359999	1.19430006	-0.24910000	1	-4.05259991	0.10940000	-0.93169999
6	0.79869998	0.35789999	0.34740001	1	-2.17359996	-2.96620011	-1.96990001
1	1.36559999	3.28279996	-0.17030001	1	-0.94019997	-2.89660001	-0.71289998
6	-1.22469997	1.80299997	-0.20350000	1	-0.92540002	-1.73210001	-2.04310012
6	-0.60240000	0.43410000	-0.31790000	1	3.08520007	2.96420002	0.01920000
6	-1.61049998	-0.48170000	0.38470000	6	2.14809990	1.00199997	-1.71200001
6	-2.44000006	0.49190000	1.19949996	1	5.26919985	-2.37319994	-0.43509999
8	-2.21339989	1.77869999	0.75629997	1	3.73609996	-2.33290005	-1.28020000
8	-0.96649998	2.80119991	-0.79500002	1	3.11299992	1.42130005	-2.00889993
8	-3.20120001	0.27930000	2.08649993	1	2.14150000	-0.05450000	-1.99409997
6	-2.49210000	-1.37360001	-0.54089999	1	2.78559995	-1.58969998	2.35150003
1	-1.10920000	-1.14839995	1.09290004	1	4.03989983	-2.74180007	1.88680005
6	-3.40319991	-2.25690007	0.31540000	1	1.97469997	2.64000010	1.35490000
6	-1.57459998	-2.28520012	-1.36049998	1	1.38779998	1.51479995	-2.31730008
6	-3.34549999	-0.51830000	-1.48029995	1	2.50259995	-2.75880003	1.05170000
1	-0.49070001	0.21259999	-1.37930000	1	4.83059978	-0.94830000	-1.38540006

TS__tBuMA--IB-rad_IB_transfer

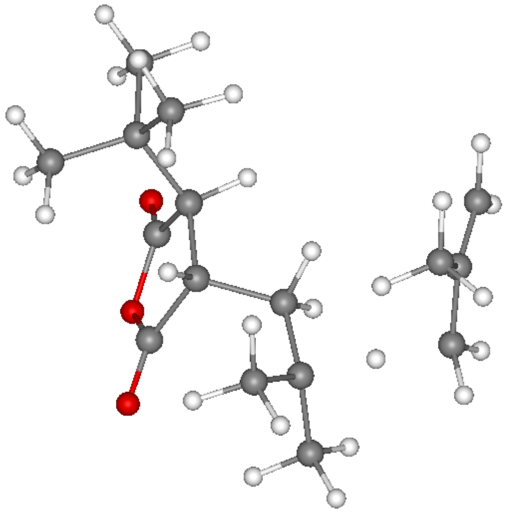

Zero-point vibrational energy	1047294.9 (Joules/Mol)
	250.30950 (Kcal/Mol)
Zero-point correction	0.398894 (Hartree/Particle)
Thermal correction to Energy	0.421635

Thermal correction to Enthalpy	0.422579
Thermal correction to Gibbs Free Energy	0.345074
Sum of electronic and zero-point Energies	-851.158793
Sum of electronic and Thermal Energies	-851.136052
Sum of electronic and Thermal Enthalpies	-851.135108
Sum of electronic and Thermal Free Energies	-851.212613

cartesian

6	4.05865192	-1.37781954	0.54976302	1	0.70605218	-0.36301962	-0.44103697
6	3.41935205	-0.78251958	-0.66553694	1	0.31295213	0.91298044	-1.57793689
6	3.32495213	0.56768042	-0.84363693	1	-4.38414812	-2.57201958	0.16516304
1	3.92805195	1.23198044	-0.23343696	1	-4.46274805	-1.33781958	-1.09683704
6	2.86725211	-1.75551951	-1.66093707	1	-3.19974804	-2.56431961	-1.14283693
1	3.03625202	0.96068037	-1.81343699	1	-4.31234789	-0.82491958	1.98106313
6	1.70805228	2.92048025	-0.43083695	1	-3.12184787	0.46748042	1.95506310
6	1.51275218	1.52828050	0.08706304	1	-4.43344784	0.44338042	0.76966304
6	0.41785213	0.69188040	-0.50943696	1	-2.55444789	-2.63221979	1.80786300
1	0.98475218	3.61678028	0.00826305	1	-1.32104778	-2.56261969	0.55086303
6	-1.60554779	2.13698030	0.04146305	1	-1.30624783	-1.39811957	1.88106322
6	-0.98324788	0.76808041	0.15586305	1	2.70435214	3.29818034	-0.18123695
6	-1.99134779	-0.14771959	-0.54673696	6	1.76725209	1.33598042	1.54996300
6	-2.82084799	0.82588041	-1.36153698	1	4.88835192	-2.03921962	0.27306303
8	-2.59424782	2.11268044	-0.91833693	1	3.35525203	-1.99891961	1.11816311
8	-1.34734786	3.13518023	0.63296306	1	2.73215199	1.75528049	1.84686303
8	-3.58204794	0.61328042	-2.24853683	1	1.76065218	0.27948040	1.83206296
6	-2.87294793	-1.03961957	0.37886304	1	2.40475202	-1.25571954	-2.51353693
1	-1.49004781	-0.81441951	-1.25493693	1	3.65905190	-2.40781975	-2.04883695
6	-3.78404784	-1.92291963	-0.47743696	1	1.59385216	2.97398043	-1.51693702
6	-1.95544779	-1.95121968	1.19846296	1	1.00695217	1.84878039	2.15526319
6	-3.72634792	-0.18431959	1.31826305	1	2.12175202	-2.42481971	-1.21373701
1	-0.87154782	0.54658043	1.21726298	1	4.44975185	-0.61431956	1.22336316

tBuMA--IB-rad--IB

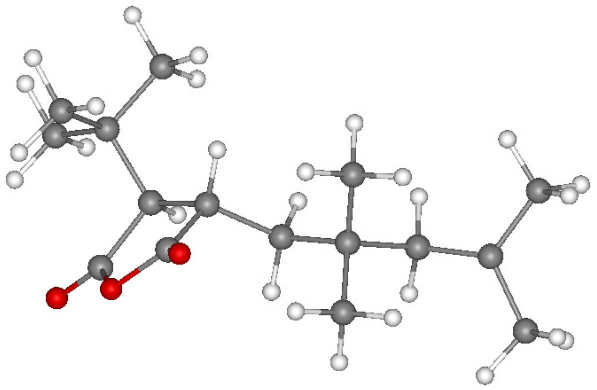

Zero-point vibrational energy	1067818.2 (Joules/Mol)
	255.21466 (Kcal/Mol)
Zero-point correction=	0.406710 (Hartree/Particle)
Thermal correction to Energy=	0.428954
Thermal correction to Enthalpy=	0.429898
Thermal correction to Gibbs Free Energy=	0.354658
Sum of electronic and zero-point Energies=	-851.201932
Sum of electronic and thermal Energies=	-851.179689
Sum of electronic and thermal Enthalpies=	-851.178744
Sum of electronic and thermal Free Energies=	-851.253984

cartesian

6	4.91081715	-0.85084569	1.10826528	1	0.14911732	0.73775423	0.95566517
6	4.01701736	0.13515431	0.43676519	1	0.09991732	-0.93814564	1.44126523
6	2.57491732	0.23105434	0.81816518	1	-5.27968264	1.76435435	0.58336520
1	2.43771720	-0.16294569	1.83466530	1	-5.06138277	0.14125434	1.24716520
6	4.66601706	1.24215424	-0.32273480	1	-4.16908264	1.49835432	1.92866528
1	2.28011727	1.28845429	0.85926521	1	-4.69588280	0.98585427	-1.74613476
6	1.91321731	-1.98544574	-0.17703480	1	-3.21598268	0.09165430	-2.06033468
6	1.55051732	-0.50114572	-0.10393481	1	-4.52728271	-0.65614569	-1.14023471
6	0.17421731	-0.28554571	0.56296521	1	-3.48938274	2.91645432	-0.63463479
1	1.23891723	-2.53824568	-0.82923484	1	-2.29658270	2.61035442	0.62616521
6	-1.40098274	-1.79984570	-0.79143482	1	-1.96768272	2.14045429	-1.04623473
6	-1.12148273	-0.41004568	-0.26543480	1	2.92411733	-2.10784554	-0.57253480
6	-2.35178256	-0.11484569	0.60596520	6	1.59091723	0.10275429	-1.50693476
6	-2.91578269	-1.49584568	0.87356520	1	5.44951725	-0.39514568	1.95496523
8	-2.36768270	-2.39834571	-0.01453481	1	5.68251705	-1.23144567	0.43036518
8	-0.92838264	-2.36684561	-1.72333479	1	2.59811735	0.03075430	-1.92223477
8	-3.71378279	-1.84484577	1.68136525	1	1.30921733	1.16125429	-1.50313473
6	-3.41298270	0.84745431	-0.00853481	1	3.94651747	1.83985436	-0.88513482
1	-2.05868268	0.30475429	1.57246530	1	5.19991732	1.93365419	0.34946519
6	-4.54618263	1.06775427	0.99706519	1	1.88681722	-2.44974566	0.81376517
6	-2.74558282	2.19815445	-0.28163481	1	0.92231727	-0.42994571	-2.18613482
6	-3.98718262	0.27935430	-1.30833471	1	5.41701746	0.87015426	-1.02843475
1	-1.04948270	0.25135431	-1.12853479	1	4.36341715	-1.70734572	1.50546527

TS__IB-allyl_MA

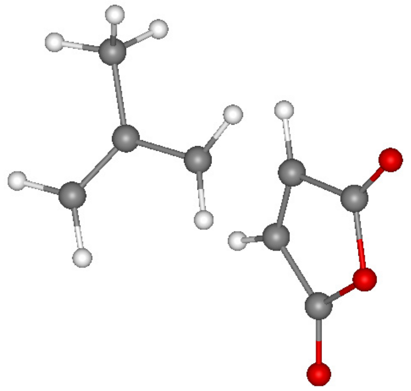

Zero-point vibrational energy	401266.3 (Joules/Mol)
	95.90495 (Kcal/Mol)
Zero-point correction=	0.152834 (Hartree/Particle)
Thermal correction to Energy=	0.163802
Thermal correction to Enthalpy=	0.164747
Thermal correction to Gibbs Free Energy=	0.113743
Sum of electronic and zero-point Energies=	-535.694897
Sum of electronic and thermal Energies=	-535.683928
Sum of electronic and thermal Enthalpies=	-535.682984
Sum of electronic and thermal Free Energies=	-535.733987

cartesian

6	-2.81728005	-0.87526000	-0.04516002	6	1.53162003	-0.16626000	0.39083996
6	-1.62897992	-0.75076002	-0.88515997	6	2.52092004	0.52704000	-0.50536001
6	-1.13678002	0.51403999	-0.74265999	6	1.66551995	-1.47766006	0.71354002
6	-2.13168001	1.27333999	0.06433998	1	2.93912005	1.41173995	-0.01946002
8	-3.07078004	0.37994000	0.51763999	1	2.04851985	0.86733997	-1.43206000
8	-3.51828003	-1.81076002	0.18263999	1	2.49851990	-2.07076001	0.35463998
8	-2.16468000	2.43343997	0.32913998	1	-0.20177996	0.15143999	1.64973998
1	-1.23668003	-1.57456005	-1.46066010	1	0.43772000	1.66463995	0.83644003
1	-0.45057997	1.02734005	-1.39946008	1	0.94822007	-1.97586012	1.35604000
6	0.42062002	0.58133996	0.87323999	1	3.34671998	-0.12976000	-0.77825999

S11. MA – OCT reaction profile

TS__tBuMA-rad_OCT

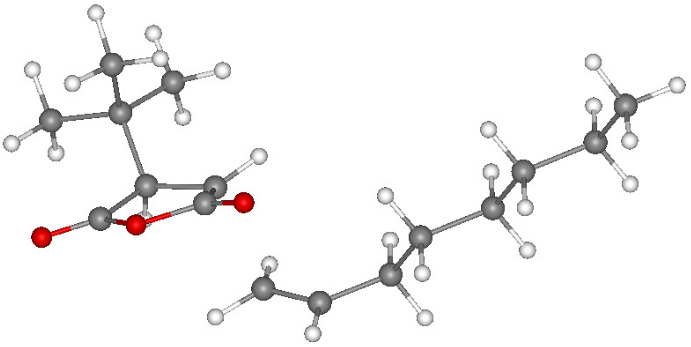

Zero-point vibrational energy	1063480.7 (Joules/Mol)
	254.17799 (Kcal/Mol)
Zero-point correction=	0.405058 (Hartree/Particle)
Thermal correction to Energy=	0.427374
Thermal correction to Enthalpy=	0.428318
Thermal correction to Gibbs Free Energy=	0.350001
Sum of electronic and zero-point Energies=	-851.168247
Sum of electronic and thermal Energies=	-851.145932
Sum of electronic and thermal Enthalpies=	-851.144988
Sum of electronic and thermal Free Energies=	-851.223305

cartesian

6	0.23614556	-2.38201737	0.77862394	6	-5.36175442	1.01728249	0.54572392
6	1.47994554	-1.70151734	1.23472393	6	-3.17045450	1.97468257	1.22562397
6	2.34144545	-1.18541741	0.07802394	6	-3.74645424	1.79118276	-1.20247602
6	3.60604572	-0.47921741	0.55032396	1	-1.05655444	0.35548258	0.22232395
6	-1.02265453	-2.05461740	1.17422390	1	-1.17525446	-1.40061736	2.02732396
1	2.07704544	-2.41341734	1.82212400	1	-1.85935450	-2.69201732	0.90922391
1	1.22924554	-0.88251740	1.91822386	1	-3.66875458	2.94168258	1.32732391
1	1.74594557	-0.51571739	-0.54977608	1	-3.19125462	1.49078250	2.20632386
1	2.61164570	-2.02791739	-0.56877607	1	-2.12845445	2.17538261	0.96732390
1	3.33404541	0.36468261	1.19722390	1	-4.15635443	2.80338264	-1.17407608
6	4.47504568	0.02488261	-0.59507608	1	-2.70225430	1.87008262	-1.51677608
6	5.74134588	0.73538262	-0.13127606	1	-4.29165459	1.24258256	-1.97477603
1	4.74854565	-0.81831741	-1.24137604	1	-5.81655455	2.01118255	0.56182396
1	3.88744545	0.70358258	-1.22527599	1	-5.91725445	0.39878261	-0.15817606
6	-1.60085440	-0.94441742	-1.44787610	1	-5.48455429	0.58048260	1.54082394
6	-1.78305447	-0.35531741	-0.13957605	1	4.19244576	-1.16081738	1.17952394
6	-3.24325466	-0.30081740	0.16122395	6	6.60304546	1.23408270	-1.28247607

6	-3.80835438	-1.19471741	-0.93767607	1	6.32784557	0.05568260	0.49792397
8	-2.83015442	-1.48371744	-1.84977603	1	5.46804571	1.57868266	0.51372397
8	-0.63245445	-1.04941738	-2.13997602	1	7.50404549	1.73608255	-0.92267603
8	-4.91555452	-1.61531734	-1.05907607	1	6.05734587	1.94558263	-1.90827608
6	-3.88315439	1.12858272	0.16882396	1	6.92074585	0.40988261	-1.92707610
1	-3.49665451	-0.76031739	1.12392390	1	0.35564554	-3.15191722	0.01982394

TS__tBuMA-rad_to_OCT_transfer

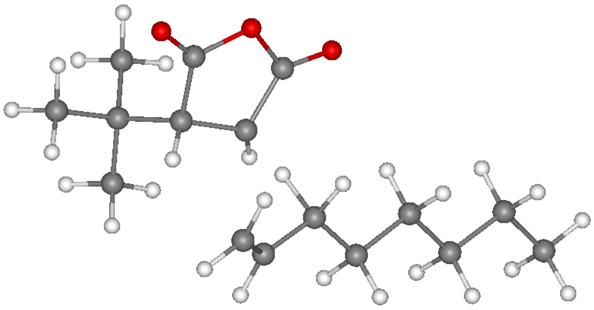

Zero-point vibrational energy	1051291.3 (Joules/Mol)
	251.26465 (Kcal/Mol)
Zero-point correction=	0.400416 (Hartree/Particle)
Thermal correction to Energy=	0.422890
Thermal correction to Enthalpy=	0.423834
Thermal correction to Gibbs Free Energy=	0.346103
Sum of electronic and zero-point Energies=	-851.161561
Sum of electronic and thermal Energies=	-851.139087
Sum of electronic and thermal Enthalpies=	-851.138143
Sum of electronic and thermal Free Energies=	-851.215874

cartesian

6	1.84972847	1.42683911	-0.05119783	1	-3.34427166	-0.82976091	-2.97529769
6	0.55042827	1.72103918	0.66770220	1	-2.54037166	0.61893916	-2.37319779
6	-0.28307167	2.79693913	0.14400218	1	-1.70567167	-0.94106090	-2.35129762
6	-1.08407164	3.58053923	0.88090217	1	-3.97257185	-2.69806075	-1.36699784
1	-0.14817168	0.63173914	0.52190220	1	-2.38007188	-2.72146082	-0.61559784
6	2.67602825	0.31723914	0.58950222	1	-3.82637167	-2.47376084	0.37180218
1	1.63752830	1.18143916	-1.09969783	1	-5.30047178	-0.54586089	-1.51189780
1	2.44602823	2.34863925	-0.08299783	1	-5.12467194	-0.24756086	0.22230218
6	-0.74367166	-1.32366085	1.51450217	1	-4.61227179	0.97513914	-0.93809783
6	-0.91857171	-0.53876090	0.29530218	1	0.64512837	1.73733914	1.75650215
6	-2.34177184	-0.06446086	0.25760219	1	-0.25047168	2.95243907	-0.93379784
6	-2.79447174	-0.34646085	1.68440211	6	3.96122837	0.01973914	-0.17239782
8	-1.85217166	-1.10636079	2.33120227	1	2.07982826	-0.59716082	0.67560220

8	0.15652832	-2.02086091	1.87350214	1	2.92202830	0.60483909	1.61870217
8	-3.79207182	-0.00236086	2.23390222	6	4.80842829	-1.07006085	0.47440219
6	-3.26157188	-0.72856081	-0.81649780	1	4.55662823	0.93753922	-0.26109782
1	-2.40617180	1.02073920	0.10870218	1	3.71562815	-0.27676088	-1.20019782
6	-4.65547180	-0.10116086	-0.74969780	6	6.08522844	-1.37086082	-0.29729781
6	-2.67127180	-0.45306087	-2.20129776	1	4.21092844	-1.98336089	0.56940222
6	-3.36017179	-2.23746085	-0.58819783	1	5.06022835	-0.77116084	1.49830210
1	-0.40997168	-0.90496087	-0.58879781	1	6.67292833	-2.15266085	0.18900219
1	-1.68887162	4.35783911	0.42920220	1	6.72042847	-0.48436087	-0.37799785
1	-1.15197170	3.47253919	1.95890212	1	5.86582804	-1.70946085	-1.31369781

tBuMA--OCT-rad

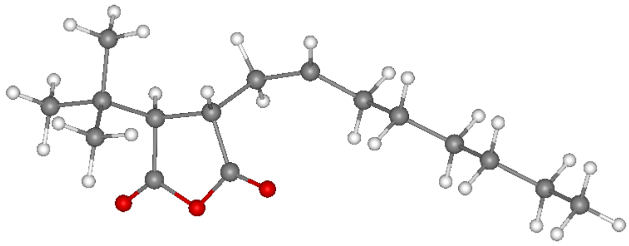

Zero-point vibrational energy	1070712.4 (Joules/Mol)
	255.90642 (Kcal/Mol)
Zero-point correction=	0.407813 (Hartree/Particle)
Thermal correction to Energy=	0.430056
Thermal correction to Enthalpy=	0.431000
Thermal correction to Gibbs Free Energy=	0.353032
Sum of electronic and zero-point Energies=	-851.202092
Sum of electronic and thermal Energies=	-851.179849
Sum of electronic and thermal Enthalpies=	-851.178904
Sum of electronic and thermal Free Energies=	-851.256872

cartesian

6	-0.27432173	-1.33994341	0.66814351	6	5.82647800	0.14825656	0.17024350
6	-1.52842176	-0.74934340	1.20524347	6	4.42617798	-1.69514346	-0.72175652
6	-2.61662173	-0.57114345	0.14614350	6	4.32127857	0.57475656	-1.77875650
6	-3.91272187	0.00055656	0.70644349	1	1.88257825	-0.71174347	-0.87185651
6	1.08767819	-0.95484340	1.11694348	1	1.68507826	-1.84744334	1.34374344
1	-1.31242180	0.21855655	1.67284346	1	1.04027820	-0.37424344	2.04504347
1	-1.92802167	-1.38074350	2.01744366	1	6.63367844	-0.13954344	-0.50815648
1	-2.82052183	-1.53984344	-0.32675651	1	5.92717838	1.21065652	0.38754350
1	-2.23082185	0.08225656	-0.64195651	1	5.97127819	-0.39644343	1.10724354
1	-4.29182148	-0.65714347	1.49904346	1	5.11107826	0.28325656	-2.47475672

6	-4.99212170	0.19085655	-0.35205650	1	3.36747837	0.37095654	-2.27315664
6	-6.29292202	0.76305652	0.19904350	1	4.40157795	1.65535653	-1.63445652
1	-4.61162186	0.85045654	-1.14155650	1	5.26027870	-1.98884344	-1.36335647
1	-5.19782162	-0.77064347	-0.83935648	1	4.51287842	-2.26194334	0.20924351
6	1.27057827	1.18955660	-0.18745649	1	3.50877833	-2.01074338	-1.22355652
6	1.90637827	-0.15544346	0.06614350	1	-3.70572162	0.96395653	1.18844354
6	3.32637835	0.17885655	0.52984351	1	-0.35262173	-2.10094357	-0.10415651
6	3.25777817	1.66775656	0.81354350	6	-7.36082172	0.95655656	-0.86835653
8	2.07777834	2.18425655	0.32244349	1	-6.08712196	1.72185659	0.68894351
8	0.23407826	1.44595659	-0.70785648	1	-6.67522144	0.10135655	0.98514348
8	4.04587841	2.36885667	1.36054349	1	-8.28092194	1.36925650	-0.44805652
6	4.47257805	-0.18724346	-0.46055651	1	-7.61592197	0.00945655	-1.35185647
1	3.55357814	-0.31424344	1.48064351	1	-7.02022171	1.64095652	-1.65025651

TS__tBuMA--OCT-rad_MA__re

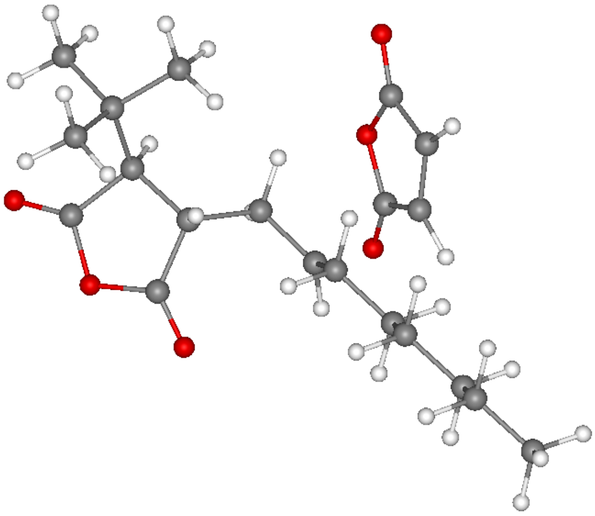

Zero-point vibrational energy	1223555.3 (Joules/Mol)
	292.43674 (Kcal/Mol)
Zero-point correction=	0.466028 (Hartree/Particle)
Thermal correction to Energy=	0.494093
Thermal correction to Enthalpy=	0.495037
Thermal correction to Gibbs Free Energy=	0.402280
Sum of electronic and zero-point Energies=	-1230.433226
Sum of electronic and thermal Energies=	-1230.405160
Sum of electronic and thermal Enthalpies=	-1230.404216
Sum of electronic and thermal Free Energies=	-1230.496973

cartesian

6	-0.30580363	3.62662363	1.62176192	6	-3.47990370	-0.46637642	0.38166189
6	0.79419643	3.13182354	0.75806189	1	-1.46440363	-1.11887646	-0.10583812

6	0.48429638	3.45652342	-0.51873809
6	-0.86430359	4.02882338	-0.52773809
8	-1.29730368	4.10932350	0.79586184
8	-0.40250361	3.63262367	2.80806208
8	-1.55150366	4.38902378	-1.43183815
1	1.74849629	2.84922361	1.17186189
1	1.06419635	3.32902336	-1.41993809
6	1.11569643	0.05622357	0.27886188
6	0.16179639	0.79402357	1.15096188
6	-1.28830361	0.83672357	0.79816186
6	2.57819629	0.10452355	0.70856190
1	0.80489641	-1.00067639	0.27156189
1	2.64889622	-0.18247646	1.76356184
6	3.47109652	-0.80557644	-0.12633812
1	2.95779634	1.13012362	0.64426190
6	-1.86670351	-1.34957647	1.93846190
6	-1.97680354	-0.53917646	0.66756189
6	-4.09000349	-1.35307646	1.45426190
8	-3.10240364	-1.80637634	2.31046200
8	-0.89070362	-1.60147643	2.57006168
8	-5.22170353	-1.66257656	1.63016188
6	-3.94110370	-0.82317638	-1.06003809
1	-3.84190369	0.54722357	0.59436190
6	-5.42960358	-0.50027645	-1.21733809
6	-3.15420365	0.03322357	-2.05563831
6	-3.70610380	-2.30587626	-1.35793817
1	-1.42010355	1.33382356	-0.17023814
1	-1.83780360	1.42722356	1.53546190
1	-5.75420380	-0.72847641	-2.23573828
1	-6.04490376	-1.07317638	-0.52573812
1	-5.62200356	0.56172359	-1.04073811
1	-3.98830366	-2.53097630	-2.38863802
1	-2.65680361	-2.59427643	-1.24483812
1	-4.30610371	-2.94757652	-0.70903814
1	-3.51250362	-0.15417641	-3.07043791
1	-3.28010368	1.10132360	-1.85873806
1	-2.08390355	-0.19007641	-2.04923820
1	1.00719643	0.39162356	-0.76213813
6	4.93729639	-0.75417650	0.28466189
1	3.38079643	-0.53437644	-1.18573809
1	3.10799623	-1.83747637	-0.04853812
6	5.83189631	-1.67047656	-0.54183811
1	5.02689648	-1.02057648	1.34506190
1	5.30319643	0.27712357	0.20376188
1	0.41859639	0.90212357	2.19946194
6	7.29489613	-1.61047649	-0.12633812
1	5.74029636	-1.40607643	-1.60173810
1	5.46909618	-2.70127630	-0.45783812
1	7.91239643	-2.27647638	-0.73313808
1	7.42369652	-1.90447652	0.91886187
1	7.69819641	-0.59987646	-0.23373812

TS__tBuMA--OCT-rad_MA__si

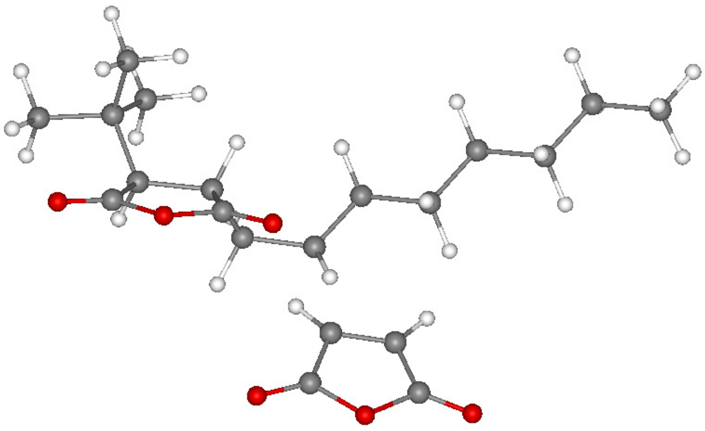

Zero-point vibrational energy 1223671.1 (Joules/Mol)

292.46442 (Kcal/Mol)

Zero-point correction= 0.466072 (Hartree/Particle)

Thermal correction to Energy= 0.494090

Thermal correction to Enthalpy= 0.495034

Thermal correction to Gibbs Free Energy= 0.402293

Sum of electronic and zero-point Energies= -1230.432082

Sum of electronic and thermal Energies= -1230.404064

Sum of electronic and thermal Enthalpies= -1230.403120

Sum of electronic and thermal Free Energies= -1230.495861

cartesian

6	-2.91401267	3.81179619	-0.12152362	6	4.36858702	-1.96940374	-0.13052362
6	-2.42731261	3.06379628	1.04137635	1	1.84798717	-0.70760369	-0.30402362
6	-1.07461262	3.09369636	1.02287626	1	1.37048721	1.11729622	1.28677630
6	-0.66931272	4.00629616	-0.07632361	1	1.60438728	2.32249641	0.02117637
8	-1.80251265	4.35619640	-0.76682365	1	6.18218708	-1.06710374	1.70627642
8	-4.01921272	3.98609638	-0.52582359	1	6.38088703	-0.10630369	0.23507637
8	0.41318727	4.40869617	-0.37162364	1	5.73168707	0.63889629	1.69517636
1	-3.09391260	2.57729626	1.73667622	1	4.79128695	-2.77580380	0.47277635
1	-0.38911271	2.80259633	1.80397630	1	3.37778735	-2.29930377	-0.45532364
6	-1.01321268	0.05579627	0.34047639	1	4.99828720	-1.86450362	-1.01712370
6	-0.18111271	1.18009627	-0.17742363	1	3.95538735	-1.65450382	2.57517624
6	1.25318718	1.30469632	0.21337640	1	3.42608738	0.02489626	2.54707623
6	-2.30111265	-0.17500371	-0.44092363	1	2.47088742	-1.22600377	1.73827624
1	-0.41301274	-0.86820376	0.32187641	1	-1.23571277	0.20939627	1.40657640
1	-2.04501271	-0.36620367	-1.48862362	6	-4.40711308	-1.57870364	-0.70022362
6	-3.13711262	-1.32630372	0.10257638	1	-3.40191269	-1.12500370	1.14857650
1	-2.89981270	0.74269629	-0.44032362	1	-2.53061271	-2.24090362	0.11867639
6	2.14828730	0.46589628	-2.01242352	6	-5.24931288	-2.72780371	-0.15872362
6	2.19358730	0.30949628	-0.50882363	1	-4.14041281	-1.78500366	-1.74402356
6	3.67228723	0.47589630	-0.14452362	1	-5.01221275	-0.66410375	-0.72272360
6	4.34878731	0.70689631	-1.48512363	1	-0.45211273	1.58319628	-1.14772367
8	3.41248727	0.67819631	-2.49852371	6	-6.51041269	-2.97940373	-0.97282362
8	1.20538735	0.41349629	-2.73442364	1	-5.52101278	-2.51840377	0.88257641
8	5.49428701	0.89849627	-1.72962356	1	-4.64211273	-3.64030385	-0.13202362
6	4.31818724	-0.67340374	0.68177634	1	-7.09461308	-3.80660367	-0.56352365
1	3.81208730	1.40219629	0.42527640	1	-6.27051306	-3.22760367	-2.01032352
6	5.73768711	-0.27640373	1.09667635	1	-7.15591288	-2.09710383	-0.98942363
6	3.48898745	-0.88950372	1.95027637				

tBuMA--OCT-rad--MA__re

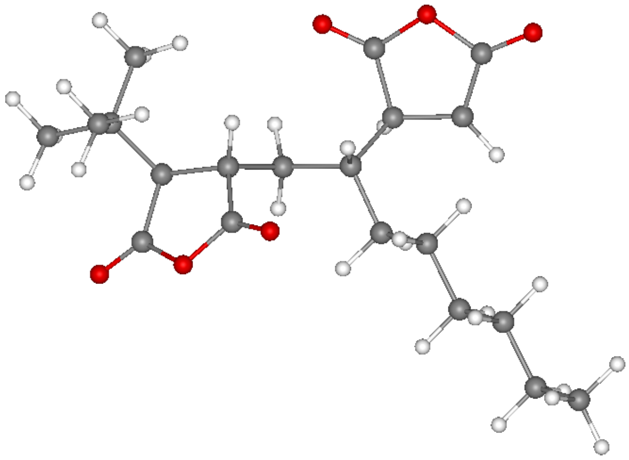

Zero-point vibrational energy	1233572.5 (Joules/Mol)
	294.83090 (Kcal/Mol)
Zero-point correction=	0.469843 (Hartree/Particle)
Thermal correction to Energy=	0.497371
Thermal correction to Enthalpy=	0.498315
Thermal correction to Gibbs Free Energy=	0.407672
Sum of electronic and zero-point Energies=	-1230.471028
Sum of electronic and thermal Energies=	-1230.443500
Sum of electronic and thermal Enthalpies=	-1230.442556
Sum of electronic and thermal Free Energies=	-1230.533199

cartesian

6	-0.06765625	3.51311088	0.02915274	1	-2.11335611	0.64451081	-0.98844725
6	0.67514378	2.46841097	0.85995275	1	-1.66585624	1.63591075	1.14915276
6	2.07714367	2.89541101	0.68825275	1	-1.08875632	0.12801081	1.83515275
6	2.13694382	4.05561066	-0.16044725	1	-6.78385639	-0.05518919	-0.21404725
8	0.82274377	4.37861061	-0.53314722	1	-5.98175621	-1.49918926	0.41265273
8	-1.23975623	3.60241079	-0.16424726	1	-5.96955633	-0.00758919	1.34995282
8	3.06464386	4.70601082	-0.53854722	1	-5.45735645	-0.19748917	-2.35524726
1	0.37244374	2.58191085	1.91145277	1	-3.70265627	-0.25078920	-2.33854747
1	2.95274377	2.48481083	1.16605282	1	-4.63985634	-1.64058924	-1.77174723
6	1.31464374	-0.00928918	0.93015277	1	-5.51875639	1.91321075	-0.98014724
6	0.29374376	1.02191079	0.42635274	1	-4.67465639	2.00851083	0.56305277
6	-1.13705623	0.71251082	0.90915275	1	-3.76215625	1.98181081	-0.94464725
6	2.48504376	-0.26068917	-0.01474726	1	0.80194378	-0.96298921	1.08555281
1	1.67554379	0.28791082	1.92395282	1	0.30714375	0.99611080	-0.66794723
1	2.97574377	0.68201077	-0.28654727	6	3.51864386	-1.21848917	0.56395274
1	2.08524370	-0.66928923	-0.94804722	1	3.02274370	-2.15588903	0.84395278
6	-1.42955625	-1.31098926	-0.60074723	1	3.92364359	-0.80408919	1.49635279

6	-2.02485609	-0.00758919	-0.11794726	6	4.66534376	-1.52278924	-0.39264727
6	-3.38725615	-0.41228917	0.45455274	1	5.16084385	-0.58668923	-0.67954725
6	-3.27875614	-1.91998923	0.57245278	1	4.25974369	-1.94288933	-1.32094729
8	-2.16225624	-2.36398911	-0.11524726	6	5.69854355	-2.48198915	0.18555275
8	-0.47375625	-1.48618925	-1.28774726	1	6.10614395	-2.06048918	1.11205280
8	-3.98475623	-2.68978906	1.13585281	1	5.20264387	-3.41578913	0.47425273
6	-4.63145638	0.03871080	-0.36974728	6	6.83674383	-2.78728914	-0.77794725
1	-3.51965618	-0.02388918	1.46915281	1	7.56204367	-3.47558904	-0.33834726
6	-5.91135645	-0.41488919	0.33695275	1	7.37494373	-1.87798917	-1.05924726
6	-4.63565636	1.56841075	-0.43694726	1	6.46544361	-3.24508905	-1.69884729
6	-4.59595633	-0.54808915	-1.78264725				

tBuMA--OCT-rad--MA__si

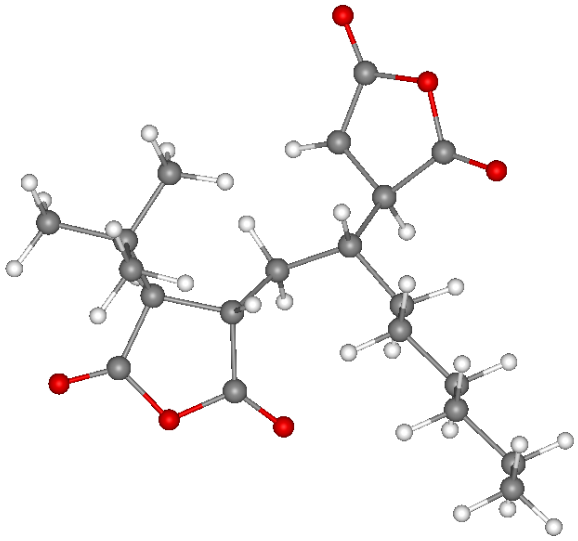

Zero-point vibrational energy 1232895.3 (Joules/Mol)
294.66905 (Kcal/Mol)
Zero-point correction= 0.469585 (Hartree/Particle)
Thermal correction to Energy= 0.497179
Thermal correction to Enthalpy= 0.498123
Thermal correction to Gibbs Free Energy= 0.407417
Sum of electronic and zero-point Energies= -1230.466155
Sum of electronic and thermal Energies= -1230.438561
Sum of electronic and thermal Enthalpies= -1230.437617
Sum of electronic and thermal Free Energies= -1230.528323

cartesian

6	2.45960546	-4.46247673	0.09559998	1	0.59800547	0.88032347	-0.13180001
6	2.28320551	-3.38277650	1.03250003	1	2.41190553	-0.88877648	0.97349995
6	0.85050547	-3.04687643	1.15530002	1	1.35660553	-0.60997653	2.34220004

6	0.21270549	-4.13187647	0.28569996	1	4.80800533	2.28772354	-2.05239987
8	1.19310546	-4.86197662	-0.33420002	1	4.42320538	3.31922364	-0.66840005
8	3.45280552	-4.99347687	-0.30470002	1	5.12770557	1.71892357	-0.41460001
8	-0.93899447	-4.36017656	0.10489997	1	2.44460535	2.45152354	-2.92030001
1	3.11260533	-2.96037650	1.57840002	1	1.06840551	2.07742357	-1.89800000
1	0.49750549	-3.16767645	2.18809986	1	2.03830552	3.52632356	-1.58789992
6	-1.05809450	-1.38497639	1.06630003	1	3.52150536	0.24042350	-2.52850008
6	0.41500551	-1.61757660	0.68460000	1	3.78780532	-0.37577647	-0.90320009
6	1.40030551	-0.58457649	1.24800003	1	2.14530540	-0.25607648	-1.55540001
6	-1.85949469	-0.52627647	0.09249997	1	-1.10029459	-0.94387650	2.06609988
1	-1.55719447	-2.35387635	1.12539995	1	0.50580549	-1.60657644	-0.40950006
1	-1.77199459	-0.94837648	-0.91650003	6	-3.33239460	-0.43937647	0.47499996
1	-1.45829439	0.49052352	0.04019998	1	-3.75249434	-1.45127654	0.52139997
6	0.50090551	1.73222351	1.77560008	1	-3.41669464	-0.02697650	1.48710001
6	1.22420549	0.86612350	0.76669997	6	-4.15509462	0.40802354	-0.48760003
6	2.51240540	1.65162349	0.47719997	1	-3.73999453	1.42322350	-0.52550006
6	2.21960545	3.02952361	1.04600000	1	-4.05689478	0.00522351	-1.50370002
8	1.06710553	2.98142362	1.80369997	6	-5.63199472	0.48462349	-0.11880003
8	-0.41669452	1.45642352	2.47860003	1	-6.04859447	-0.52857649	-0.08490004
8	2.82610536	4.04422331	0.93969995	1	-5.73039436	0.88322347	0.89739996
6	3.02600551	1.67212343	-0.98860008	6	-6.44519472	1.33812356	-1.08169997
1	3.31850553	1.24702358	1.10500002	1	-6.07739449	2.36772346	-1.10799992
6	4.42640543	2.29192352	-1.02839994	1	-7.49909449	1.37332344	-0.79650003
6	3.11890554	0.23762351	-1.51319993	1	-6.39359474	0.94622356	-2.10139990
6	2.08620548	2.47822356	-1.88899994				

TS__tBuMA--OCT-rad_OCT

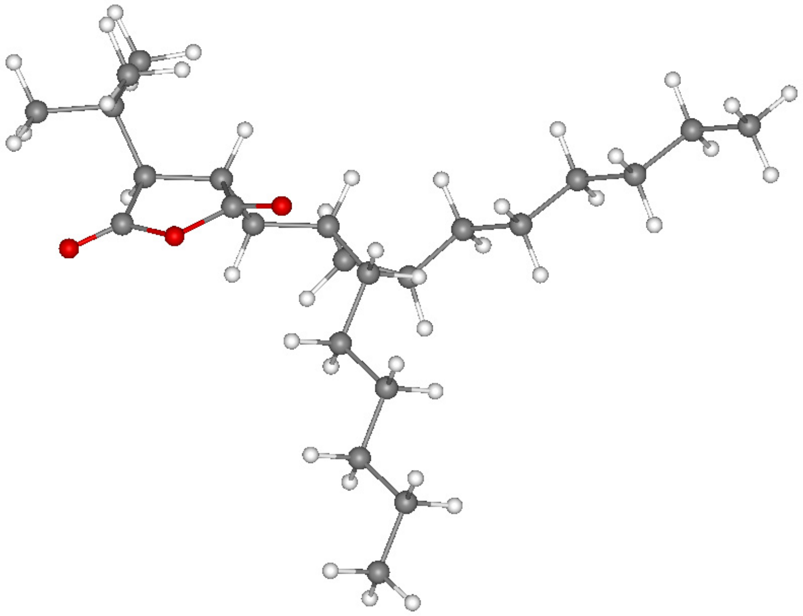

Zero-point vibrational energy	1664504.3 (Joules/Mol)
	397.82608 (Kcal/Mol)
Zero-point correction=	0.633976 (Hartree/Particle)
Thermal correction to Energy=	0.667086
Thermal correction to Enthalpy=	0.668030
Thermal correction to Gibbs Free Energy=	0.562780
Sum of electronic and zero-point Energies=	-1165.422457
Sum of electronic and thermal Energies=	-1165.389348
Sum of electronic and thermal Enthalpies=	-1165.388403
Sum of electronic and thermal Free Energies=	-1165.493654

cartesian

6	2.95697021	-1.32908142	-2.03033733	6	-4.30412960	-3.63098145	-0.27413717
6	1.88517010	-0.34068140	-2.35733724	6	-5.24583006	-2.43838143	1.71746278
6	0.55807018	-0.62238139	-2.42123723	1	-2.51352978	-1.86058140	0.77856284
1	-0.13392982	0.10541861	-2.83023739	1	-1.98572981	-1.27788138	-1.58653712
1	0.22487020	-1.65578139	-2.45023727	1	-2.23592997	0.41751859	-1.24243712
6	3.75007010	-0.98028141	-0.76793718	1	-6.99782991	-3.34128141	-0.17703718
1	3.66017008	-1.38928139	-2.87283731	1	-7.07912970	-1.57608140	-0.17643720
1	2.52077007	-2.32908154	-1.92883718	1	-6.38362980	-2.42428136	-1.55433714
6	4.89916992	-1.94238138	-0.48963720	1	-5.74973011	-3.35898137	2.02026272
1	3.07067013	-0.96208137	0.09156282	1	-4.28522968	-2.41258144	2.23906279
1	4.14447021	0.03881860	-0.86083716	1	-5.85032988	-1.60458148	2.08416271
6	5.68297005	-1.59448147	0.76946282	1	-4.85683012	-4.54098129	-0.02893718
1	5.57917023	-1.95438147	-1.35063720	1	-4.15712976	-3.61828136	-1.35763717
1	4.50726986	-2.96368146	-0.40383717	1	-3.32272959	-3.71478152	0.19776282
6	6.83967018	-2.54628134	1.05266285	1	1.21597016	0.74751860	0.14666282
1	5.00267029	-1.58548141	1.63016284	6	-0.12412980	4.67911863	-0.14273718
1	6.06957006	-0.57108140	0.68496281	1	1.43367016	3.23341846	0.19906282
6	0.12577021	0.80291861	0.24916282	1	0.20537019	3.26671863	1.44626284
6	-0.45772982	-0.42378139	-0.38183719	6	0.58217025	5.85421896	0.52326280
6	-1.89132977	-0.52188140	-0.79763716	1	-1.20542979	4.76801872	0.02016282
6	-0.35492980	2.14331865	-0.29693717	1	0.02067018	4.73791885	-1.22913718
1	-0.06992981	0.77471864	1.32976282	1	-0.03842980	-1.35718143	-0.00963718
1	-1.43422985	2.25281858	-0.13893718	1	2.20687008	0.69011861	-2.49773741
6	0.35207021	3.32261848	0.36086282	6	0.09857017	7.20741892	0.02146282
1	-0.19892982	2.18191862	-1.38183713	1	1.66267025	5.76701880	0.35946283
6	-3.05912971	0.09501860	1.37056279	1	0.43827021	5.79281902	1.60806286
6	-2.89892960	-0.95898139	0.30146283	1	0.61977017	8.03031921	0.51616281
6	-4.31732988	-1.12278140	-0.25623718	1	-0.97152984	7.33901882	0.20376283
6	-5.00472975	0.15111861	0.19556281	1	0.26197016	7.31431866	-1.05463719

8	-4.25192976	0.76031864	1.17786276	6	7.61406994	-2.18838143	2.31316280
8	-2.33112979	0.38751858	2.26256275	1	7.52066994	-2.55348134	0.19366282
8	-6.03152990	0.62591863	-0.16713718	1	6.45426989	-3.56908154	1.13726282
6	-5.08613014	-2.40158153	0.19606280	1	8.43566990	-2.88568139	2.49236274
1	-4.31552982	-1.11688149	-1.35033715	1	6.96817017	-2.20718145	3.19526267
6	-6.46712971	-2.42998147	-0.46413720	1	8.04337025	-1.18498147	2.24436259

TS__tBuMA--OCT-rad_OCT_transfer

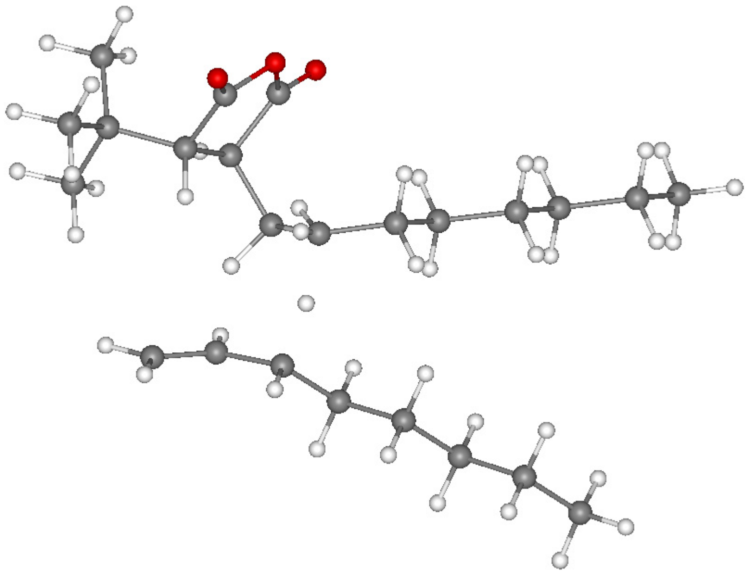

Zero-point vibrational energy	1651113.0 (Joules/Mol)
	394.62547 (Kcal/Mol)
Zero-point correction=	0.628876 (Hartree/Particle)
Thermal correction to Energy=	0.662482
Thermal correction to Enthalpy=	0.663426
Thermal correction to Gibbs Free Energy=	0.557607
Sum of electronic and zero-point Energies=	-1165.417586
Sum of electronic and thermal Energies=	-1165.383979
Sum of electronic and thermal Enthalpies=	-1165.383035
Sum of electronic and thermal Free Energies=	-1165.488854

cartesian

6	-0.33438700	-1.06149280	-0.40372854	1	-1.95498705	-3.29859281	-0.45172855
6	0.63621300	-0.08969286	0.21547145	1	-2.88538718	-1.94919288	-1.06622863
1	0.76461297	-0.22769286	1.29117143	6	-4.63228703	-3.74649286	0.13067144
6	1.93701291	0.15850714	-0.50992858	1	-4.18328714	-1.86829293	1.07947135
6	-1.50438714	-1.42469287	0.50187147	1	-3.25038719	-3.21649265	1.69217134
1	-0.70458704	-0.65759289	-1.35582864	6	-1.77858710	2.54100728	0.53097147

1	-2.03348708	-0.51459289	0.80817145	6	-0.27438700	2.47510719	0.35367146
6	-2.48918700	-2.39169288	-0.14342856	6	0.53151298	2.99020720	1.46047139
1	-1.10918713	-1.86949289	1.42247140	6	1.67571306	3.67490721	1.34657145
6	3.01391292	-2.05349278	0.09457145	1	0.04951298	1.19800711	0.26107144
6	3.17131281	-0.55149287	0.08897144	6	-2.56788707	2.12320733	-0.70352852
6	4.44901323	-0.31559286	-0.72282857	1	-2.07458711	1.92550719	1.38987136
6	4.67571306	-1.65139294	-1.40472865	1	-2.06068707	3.56850719	0.79827148
8	3.86291289	-2.61019278	-0.83662856	1	2.23071289	4.00510740	2.21707129
8	2.29011297	-2.73079276	0.75017148	1	2.08271289	3.94680738	0.37687147
8	5.41701269	-1.93169284	-2.28982854	1	0.05651298	2.85100722	-0.61902857
6	5.69511271	0.15940714	0.08377144	1	0.16301298	2.76610732	2.46167135
1	4.28031301	0.42070714	-1.51482856	6	-4.07658720	2.19930720	-0.50472856
6	6.87291288	0.38430712	-0.86782855	1	-2.28958702	1.10440707	-0.99442858
6	5.35811281	1.49440706	0.75287145	1	-2.27998710	2.76430726	-1.54592860
6	6.08871269	-0.87019289	1.14567137	6	-4.87478685	1.80820715	-1.74292862
1	3.26351285	-0.23279285	1.12857139	1	-4.35278702	3.21730733	-0.20262855
1	2.18111300	1.22710717	-0.49422854	1	-4.36678696	1.55150712	0.33207145
1	1.83651304	-0.11389285	-1.56712866	6	-6.38028717	1.88470709	-1.53292859
1	7.73401308	0.76010716	-0.30942854	1	-4.59818697	0.79160714	-2.04572845
1	7.17191315	-0.53029287	-1.37792861	1	-4.58748722	2.45770717	-2.57792854
1	6.62601280	1.12470710	-1.63352859	1	-6.92758703	1.60120714	-2.43482852
1	6.94641304	-0.50949287	1.71787143	1	-6.69318724	2.89720726	-1.26312864
1	5.28491306	-1.06239283	1.86147141	1	-6.70398712	1.21790707	-0.72892857
1	6.37811279	-1.82549286	0.69967145	6	-5.78148699	-4.12759304	1.05317140
1	6.22921276	1.87060702	1.29467142	1	-4.09668684	-4.65079260	-0.18022856
1	5.08121300	2.24950719	0.01217145	1	-5.03198719	-3.30449271	-0.78952855
1	4.53911304	1.41680706	1.47117138	1	-6.47168684	-4.82339287	0.57047147
1	0.19761297	-1.98539293	-0.66912854	1	-6.35818720	-3.24859285	1.35467136
6	-3.64728689	-2.77469277	0.76977146	1	-5.41608715	-4.60689259	1.96557140

tBuMA--OCT-rad--OCT

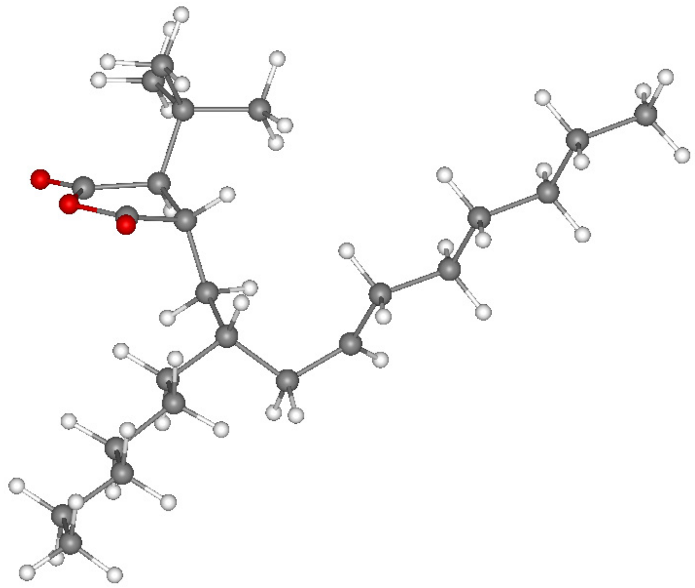

Zero-point vibrational energy	1670295.5 (Joules/Mol)
	399.21021 (Kcal/Mol)
Zero-point correction=	0.636182 (Hartree/Particle)
Thermal correction to Energy=	0.667933
Thermal correction to Enthalpy=	0.668877
Thermal correction to Gibbs Free Energy=	0.567798
Sum of electronic and zero-point Energies=	-1165.465932
Sum of electronic and thermal Energies=	-1165.434182
Sum of electronic and thermal Enthalpies=	-1165.433238
Sum of electronic and thermal Free Energies=	-1165.534317

cartesian

6	-1.54806566	-1.64697564	-0.74300277	1	-2.72196579	3.30242443	-1.15950274
6	-0.81916559	-1.24907565	-1.97940278	1	-2.53326583	2.36952448	0.32979718
6	0.65553433	-1.03337562	-1.99960268	1	3.09593415	0.89332438	-0.49540284
1	1.16653430	-1.99457562	-1.83220279	1	0.83803433	-0.33077568	0.02539718
1	-1.35236561	-1.29487562	-2.92400289	6	-3.01536584	-1.98927557	-0.97990286
1	0.96773434	-0.70677567	-2.99810290	1	-1.48466563	-0.85067570	0.01519718
6	2.72623420	-0.03687564	-0.93750286	1	-1.04216564	-2.50557566	-0.27070281
6	1.19183433	-0.02377564	-0.96560287	1	-3.07986569	-2.80817556	-1.70600283
6	0.64023435	1.37922430	-1.26510274	1	-3.51096582	-1.13107562	-1.45020282
6	3.33603430	-1.20007563	-0.16430283	6	4.85923433	-1.21627569	-0.21560283
1	3.09613419	-0.04047564	-1.97170281	1	5.24373436	-0.27547562	0.19689718
1	2.95393419	-2.15437555	-0.54610288	1	5.18843412	-1.24217570	-1.26200283
1	3.00923419	-1.13527560	0.87969720	6	5.47973442	-2.38727570	0.53639722
6	1.40203440	2.41842437	0.92139721	1	5.13193417	-3.32967567	0.09429718

6	0.25163433	2.22522449	-0.03740282	1	5.11383438	-2.38977575	1.57069719
6	-0.12926565	3.65832424	-0.42280284	6	7.00383425	-2.36697578	0.54249716
6	1.12993431	4.43322420	-0.08660282	1	7.36953402	-2.31997585	-0.49000284
8	1.92723441	3.68052435	0.75139713	1	7.34973431	-1.44527566	1.02419722
8	1.85143435	1.66952431	1.71449721	6	7.61703444	-3.56947565	1.24589717
8	1.45933437	5.51362419	-0.42210284	1	8.70843410	-3.52227545	1.24749720
6	-1.38766563	4.24662447	0.28529719	1	7.32903433	-4.50517559	0.75849712
1	-0.29156566	3.75082445	-1.50020278	1	7.28933430	-3.62997580	2.28749704
6	-1.64356565	5.66672421	-0.22590283	6	-3.76436567	-2.37357569	0.28919718
6	-2.59856582	3.38142443	-0.07590283	1	-3.27316570	-3.23677588	0.75519717
6	-1.20636559	4.27462435	1.80459726	1	-3.68656588	-1.55707562	1.01829720
1	-0.53556561	1.69762433	0.50219715	6	-5.23426580	-2.69737577	0.05129718
1	-0.27046567	1.29632437	-1.86720276	1	-5.31346560	-3.52117586	-0.66920280
1	1.35993433	1.93432438	-1.87900281	1	-5.72356558	-1.83797562	-0.42430282
1	-2.53696585	6.07842445	0.25029719	6	-5.99086571	-3.06637573	1.32169724
1	-0.80956566	6.33612442	-0.01780282	1	-5.50516558	-3.92767572	1.79489720
1	-1.81076562	5.67292404	-1.30640280	1	-5.90946579	-2.24447584	2.04249716
1	-2.11326575	4.65052414	2.28359699	6	-7.45896578	-3.38277578	1.07439721
1	-1.01296568	3.28192425	2.22019720	1	-7.97756577	-3.63717556	2.00159717
1	-0.38656563	4.93132401	2.10699701	1	-7.57426596	-4.22867584	0.39099717
1	-3.50996566	3.83032417	0.32619718	1	-7.98016548	-2.53047562	0.62959719

TS__OCT-allyl_MA

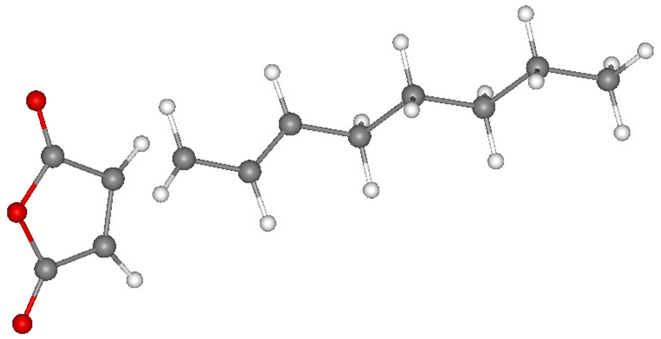

Zero-point vibrational energy	704141.5 (Joules/Mol)
	168.29387 (Kcal/Mol)
Zero-point correction=	0.268193 (Hartree/Particle)
Thermal correction to Energy=	0.284516
Thermal correction to Enthalpy=	0.285460
Thermal correction to Gibbs Free Energy=	0.220081
Sum of electronic and zero-point Energies=	-692.831568
Sum of electronic and thermal Energies=	-692.815245
Sum of electronic and thermal Enthalpies=	-692.814301
Sum of electronic and thermal Free Energies=	-692.879680

cartesian

6	5.86730289	-1.01071250	0.09112813	1	-0.97349668	-0.28101251	-1.56247187
6	4.62240314	-1.07901251	-0.66407192	1	-0.78789663	-1.31181252	-0.15267187
6	4.10140324	0.18258750	-0.75087190	6	-3.38559651	-0.31831250	-0.22787188
6	5.13440323	1.10568750	-0.20047188	1	-1.97949672	0.41028750	1.23612821
8	6.12400341	0.33958751	0.36102813	1	-2.14129686	1.43948758	-0.17097187
8	6.61210346	-1.86681247	0.45462814	6	-4.62449646	0.32338750	0.38562813
8	5.15820313	2.29628730	-0.19227187	1	-3.48889661	-0.33671251	-1.31997180
1	4.21900320	-2.00941253	-1.03267181	1	-3.32909656	-1.36821246	0.08522814
1	3.36140323	0.53498751	-1.45287180	1	0.48450327	1.48618758	-0.44667184
6	2.63760328	0.61378753	0.87952811	1	1.37560320	-1.12541246	0.88662809
6	1.44860327	-0.08851250	0.56432813	6	-5.91389656	-0.39541250	0.01512813
6	0.41210330	0.44518751	-0.13177188	1	-4.51729679	0.34588751	1.47612822
1	3.27710342	0.24438751	1.67172813	1	-4.68419647	1.37098753	0.06892813
1	2.68420315	1.68278754	0.70242810	1	-6.78469658	0.08358750	0.46812814
6	-0.85079670	-0.26631251	-0.47087187	1	-6.06889629	-0.40271249	-1.06727183
6	-2.09039664	0.39128751	0.14652812	1	-5.89949703	-1.43601251	0.35052815

S12. MA – BU2 reaction profile

TS__tBuMA-rad_BU2

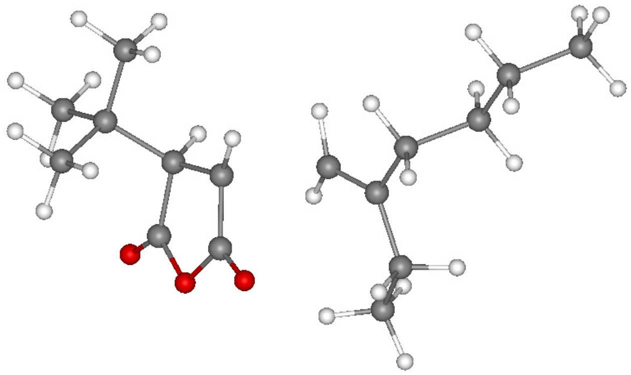

Zero-point vibrational energy	1063127.2 (Joules/Mol)
	254.09350 (Kcal/Mol)
Zero-point correction=	0.404924 (Hartree/Particle)
Thermal correction to Energy=	0.427266
Thermal correction to Enthalpy=	0.428210
Thermal correction to Gibbs Free Energy=	0.350736
Sum of electronic and zero-point Energies=	-851.173767
Sum of electronic and thermal Energies=	-851.151425
Sum of electronic and thermal Enthalpies=	-851.150481
Sum of electronic and thermal Free Energies=	-851.227955

cartesian

6	-1.00059795	3.39054775	0.74125654	6	3.02560210	1.31724787	0.40345654
6	-1.75779796	2.39264774	-0.12024346	8	2.41360211	2.07014799	-0.56124347
6	-1.49429798	0.93854785	0.13195655	8	0.79380202	1.80124784	-2.11584353
6	-2.42699790	-0.03245217	-0.53134346	8	3.86610198	1.77694786	1.11095655
6	-3.74299812	-0.22285217	0.23365656	6	3.53020191	-1.16905212	-0.03184346
6	-4.69769812	-1.18615222	-0.46174344	1	2.16300201	-0.34685218	1.41155660
6	-0.43289796	0.48734784	0.86275655	6	4.62130213	-1.24005222	1.03815651
1	-2.83679795	2.56564784	-0.02234346	6	2.84160209	-2.53245211	-0.12044346
1	-1.31259799	4.40864801	0.50245655	6	4.15050220	-0.81575215	-1.38404346
1	-1.18869793	3.22894788	1.80565655	1	0.80460203	-0.75855219	-1.03864348
1	0.07690203	3.34284782	0.57485658	1	-0.38289794	-0.55945218	1.14425659
1	-1.93719792	-1.00625217	-0.63674343	1	0.14290205	1.16624784	1.48175657
1	-2.65089798	0.31554782	-1.54694343	1	3.58050203	-3.31185222	-0.32144347
1	-4.23859787	0.74534786	0.36565655	1	2.34100199	-2.78965211	0.81745654
1	-3.52039790	-0.58805215	1.24245656	1	2.10270214	-2.57455206	-0.92334348
1	-4.91759777	-0.81375217	-1.46864343	1	4.86530209	-1.58575213	-1.68394339
6	-5.99739790	-1.38915217	0.30375656	1	3.39670205	-0.74395216	-2.17254353

1	-6.66319799	-2.08285213	-0.21444345	1	4.69280195	0.13254783	-1.34704340
1	-5.81219816	-1.79475212	1.30205655	1	5.36240196	-1.99535227	0.76405656
1	-6.53759813	-0.44675219	0.42855656	1	5.13390207	-0.28715217	1.16285658
6	1.41000199	1.32844782	-1.20594347	1	4.20380211	-1.52205217	2.00895643
6	1.30220199	0.05614781	-0.53574347	1	-1.53729796	2.58364773	-1.17874348
6	2.46470213	-0.10065216	0.38635656	1	-4.19949818	-2.15255213	-0.60164344

TS__tBuMA-rad_to_BU2_transfer

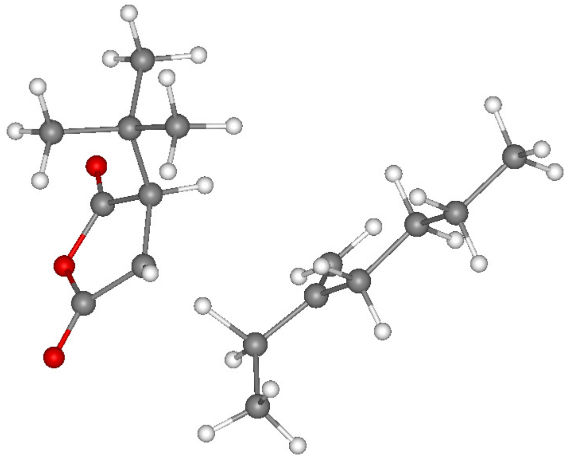

Zero-point vibrational energy	1050500.9 (Joules/Mol)
	251.07574 (Kcal/Mol)
Zero-point correction=	0.400115 (Hartree/Particle)
Thermal correction to Energy=	0.422586
Thermal correction to Enthalpy=	0.423530
Thermal correction to Gibbs Free Energy=	0.345924
Sum of electronic and zero-point Energies=	-851.164341
Sum of electronic and thermal Energies=	-851.141870
Sum of electronic and thermal Enthalpies=	-851.140925
Sum of electronic and thermal Free Energies=	-851.218532

cartesian

6	-0.34910423	-3.38464570	0.84066522	6	3.09099579	-0.17164566	-1.25863481
6	-0.04400426	-2.49084568	-0.33743477	8	3.71179581	-1.27414560	-0.72913480
6	-1.08710432	-1.62504566	-0.90413475	8	3.50999570	-2.59464574	1.08856511
6	-2.11010408	-1.06224561	0.04956523	8	3.43959570	0.29335433	-2.29723477
6	-3.09780431	-0.05604565	-0.52213478	6	2.24529576	1.69855440	0.28776523
6	-4.07660437	0.46825433	0.52306521	1	1.06439579	0.37525433	-0.93913478
6	-1.07310414	-1.33404565	-2.21793461	6	2.24359584	2.75015426	-0.82363480
1	0.86569571	-1.68514562	0.12766522	6	1.11709571	2.02725434	1.26816511
1	0.55949575	-3.87444568	1.19406533	6	3.58779573	1.71995437	1.01996517
1	-0.77910429	-2.83604574	1.68066525	1	1.54569578	-0.73414564	1.65286517

1	-1.06100416	-4.16814566	0.56036520	1	-1.80250430	-0.67864567	-2.67683482
1	-1.57900429	-0.60614562	0.89636523	1	-0.32420427	-1.76024568	-2.87683463
1	-2.66710424	-1.89824569	0.49106520	1	1.23769569	3.04455423	1.64836526
1	-3.66160440	-0.51694566	-1.34093475	1	0.13889575	1.97165442	0.78226525
1	-2.55400419	0.78715432	-0.96373475	1	1.10509574	1.36095428	2.13366532
1	-4.61700439	-0.37444568	0.96926528	1	3.75199580	2.69765425	1.47886515
6	-5.07330418	1.46705437	-0.04723477	1	3.63149571	0.97385430	1.81796527
1	-5.76220417	1.82855439	0.71916521	1	4.42379570	1.53665435	0.34026521
1	-4.56580400	2.33815432	-0.47063479	1	2.41449571	3.74245429	-0.39823478
1	-5.67320442	1.01875436	-0.84373480	1	3.01549578	2.55975437	-1.56803489
6	3.08459592	-1.68034565	0.45026523	1	1.28159571	2.77305436	-1.34323478
6	1.89349580	-0.85624564	0.63416523	1	0.54209572	-2.99524570	-1.10813475
6	1.97399569	0.29035437	-0.33123475	1	-3.51830435	0.93435431	1.34316516

tBuMA--BU2-rad

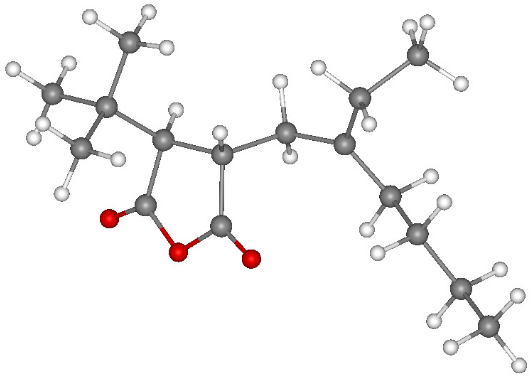

Zero-point vibrational energy	1070477.2 (Joules/Mol)
	1070477.2 (Kcal/Mol)
Zero-point correction=	0.407723 (Hartree/Particle)
Thermal correction to Energy=	0.430144
Thermal correction to Enthalpy=	0.431088
Thermal correction to Gibbs Free Energy=	0.352286
Sum of electronic and zero-point Energies=	-851.205151
Sum of electronic and thermal Energies=	-851.182731
Sum of electronic and thermal Enthalpies=	-851.181786
Sum of electronic and thermal Free Energies=	-851.260588

cartesian

6	1.53838694	1.87950635	-0.29470217	6	-2.59071302	-1.84859347	0.80679786
6	1.27788687	0.66550654	0.54019785	8	-1.53571308	-2.48039365	0.18389782
6	2.40618682	-0.21769349	0.96849787	8	0.26838687	-1.91819358	-1.01560211
6	3.39708686	-0.59429348	-0.13530219	8	-3.40201306	-2.47259355	1.41039789

6	4.47768688	-1.55299354	0.34999782	6	-3.66621304	0.17540652	-0.32360217
6	-0.10161312	0.43200654	1.05339777	1	-2.60721302	0.12960652	1.54939783
6	2.36328697	2.95260644	0.42719781	6	-5.00491333	-0.03829348	0.38759783
1	2.05868697	1.60190654	-1.21960211	6	-3.47201300	1.67910647	-0.53620219
1	3.34358692	2.56970644	0.71919787	6	-3.68601298	-0.54269350	-1.67500210
1	2.52568698	3.82170653	-0.21480218	1	-1.05701315	0.44740653	-0.88350213
1	1.85988688	3.29530644	1.33449781	1	-0.55411315	1.37950659	1.37579787
1	1.99798679	-1.13799357	1.40019786	1	-0.08611313	-0.21649349	1.93669784
1	2.96778679	0.25910652	1.79079783	1	-5.81791353	0.36200649	-0.22340217
1	3.87418675	0.30820650	-0.53530216	1	-5.20731354	-1.09189343	0.57519782
1	2.84468699	-1.04749346	-0.96390212	1	-5.02731323	0.47980648	1.35009789
1	5.00768661	-1.10579348	1.19959784	1	-4.47761345	-0.13369349	-2.30690217
6	5.47678661	-1.92399359	-0.73650217	1	-2.74671316	-0.42709351	-2.22250223
1	6.23408651	-2.61889362	-0.36620218	1	-3.88191319	-1.61239338	-1.56400216
1	4.97918653	-2.40079355	-1.58510220	1	-4.30751324	2.08560634	-1.11110210
1	5.99848652	-1.04119349	-1.11660218	1	-3.43951321	2.21350646	0.41729781
6	-0.66501313	-1.56539345	-0.37060219	1	-2.55861306	1.91560650	-1.08630216
6	-1.09081316	-0.17079349	0.01419782	1	4.00408649	-2.46279359	0.73639786
6	-2.50501299	-0.35229349	0.57179785	1	0.58718693	2.32160640	-0.61240220

TS__tBuMA--BU2-rad_MA__re

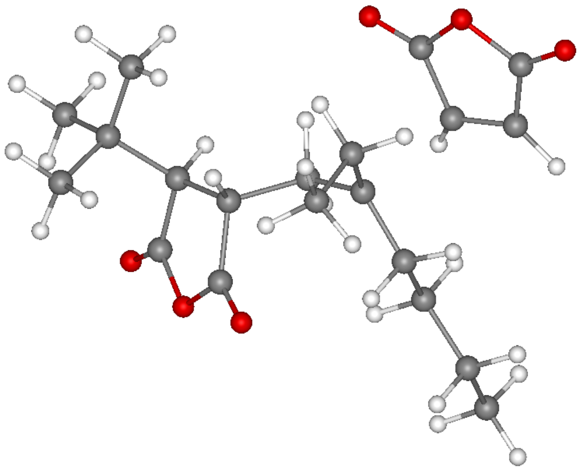

Zero-point vibrational energy	1225419.0 (Joules/Mol)
	292.88217 (Kcal/Mol)
Zero-point correction=	0.466737 (Hartree/Particle)
Thermal correction to Energy=	0.494537
Thermal correction to Enthalpy=	0.495481
Thermal correction to Gibbs Free Energy=	0.406181
Sum of electronic and zero-point Energies=	-1230.434358
Sum of electronic and thermal Energies=	-1230.406558
Sum of electronic and thermal Enthalpies=	-1230.405614

Sum of electronic and thermal Free Energies= -1230.494914

cartesian

6	-2.45779991	-2.26834536	1.13406360	8	1.12549996	1.82365453	-1.66623640
6	-2.75559998	-0.82314545	1.27396357	8	3.94870019	1.20735455	1.69406366
6	-4.04279995	-0.64574540	0.85216361	6	3.50390005	-1.55644548	0.19106364
6	-4.52079964	-1.90514541	0.29546362	1	2.11669993	-0.81674546	1.65776360
8	-3.50710011	-2.85754538	0.48266363	6	4.55240011	-1.80304539	1.27876365
8	-1.49520004	-2.88664556	1.48316360	6	2.74830008	-2.86564541	-0.05383637
8	-5.55339956	-2.19644547	-0.22283638	6	4.19690037	-1.10534537	-1.09623635
1	-2.20589995	-0.20884542	1.96966362	1	1.29359996	-0.83924544	-1.12303638
1	-4.65899992	0.23915456	0.89176363	1	-0.07489996	-1.21954548	0.92606366
6	-1.86539996	1.30985451	-0.59493637	1	-0.02119996	0.49535453	1.29056358
6	-1.27279997	-0.04894543	-0.37013638	1	5.25100040	-2.57634544	0.95026362
6	-1.51189995	-1.04644537	-1.47193635	1	5.12440014	-0.90584546	1.51206362
6	-1.07769990	-0.58384544	-2.87093639	1	4.08600044	-2.15064549	2.20446372
6	-0.03879996	-0.22614542	0.46826363	1	4.87370014	-1.88544548	-1.45153642
6	-1.77839994	2.34095454	0.52446365	1	3.48810005	-0.90614545	-1.90513635
1	-1.35990000	1.72825456	-1.47623634	1	4.79630041	-0.20404543	-0.94303632
1	-0.73029995	2.55445457	0.75956362	1	3.45499992	-3.65784550	-0.31143638
6	-2.46670008	3.65035462	0.15286364	1	2.20239997	-3.18804550	0.83636361
1	-2.22650003	1.95265460	1.44656360	1	2.03340006	-2.79784536	-0.87703639
1	-2.58389997	-1.26474547	-1.52943635	1	-2.91059995	1.17985451	-0.90343642
1	-1.01909995	-1.99184549	-1.22683632	1	-1.18770003	-1.40864539	-3.57723641
1	-0.04369996	-0.24074543	-2.90153646	6	-2.36960006	4.70365477	1.24676359
1	-1.69879997	0.23855458	-3.22813630	1	-3.52029991	3.45455456	-0.07753637
6	1.63429999	1.22225451	-0.77383637	1	-2.02370000	4.03815460	-0.77083635
6	1.32599998	-0.16704543	-0.26583636	1	-2.86619997	5.63065481	0.95296365
6	2.48720002	-0.48164541	0.68436360	1	-1.32809997	4.94675493	1.47266364
6	3.13319993	0.87215459	0.90026361	1	-2.83620000	4.36155462	2.17476368
8	2.63299990	1.77995455	-0.01863636				

TS__tBuMA--BU2-rad_MA__si

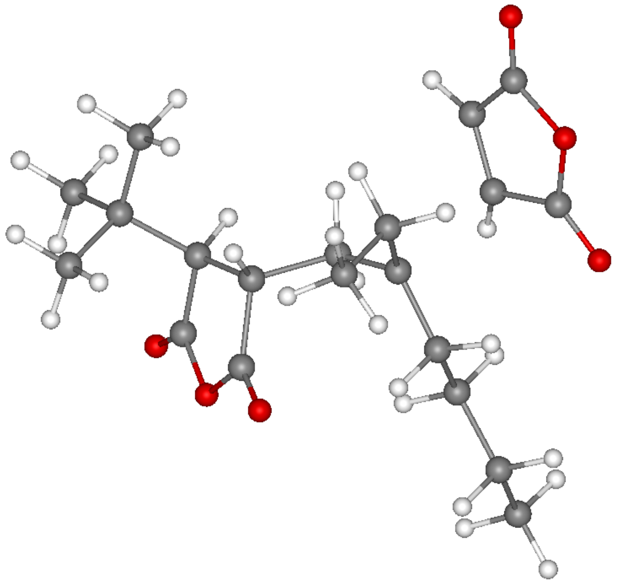

Zero-point vibrational energy	1224910.7 (Joules/Mol)
	292.76068 (Kcal/Mol)
Zero-point correction=	0.466544 (Hartree/Particle)
Thermal correction to Energy=	0.494454
Thermal correction to Enthalpy=	0.495398
Thermal correction to Gibbs Free Energy=	0.405256
Sum of electronic and zero-point Energies=	-1230.431090
Sum of electronic and thermal Energies=	-1230.403181
Sum of electronic and thermal Enthalpies=	-1230.402237
Sum of electronic and thermal Free Energies=	-1230.492379

cartesian

6	-3.68890333	-2.83621097	0.67972904	8	1.12689662	1.75398910	-1.74327099
6	-2.63840342	-2.16351080	1.43342900	8	3.72969651	1.26038909	1.81052911
6	-2.79400349	-0.81261092	1.28072906	6	3.47329664	-1.49511087	0.23162907
6	-4.08120346	-0.61421090	0.57302904	1	2.01219654	-0.84351093	1.67002904
8	-4.53160334	-1.84581089	0.16142908	6	4.51509666	-1.69641089	1.33512902
8	-3.89980340	-3.99381089	0.48912907	6	2.78939652	-2.83911085	-0.03317093
8	-4.68130350	0.39118907	0.34542906	6	4.16509676	-1.00361085	-1.04177094
1	-1.89600348	-2.70931077	1.99542904	1	1.24359655	-0.87721092	-1.11567092
1	-2.38320351	-0.03671092	1.90742910	1	-0.16870347	-1.26631081	0.86722904
6	-1.85960340	1.34018910	-0.66247094	1	-0.10820346	0.43498909	1.28952909
6	-1.35650349	-0.04581092	-0.39647090	1	5.25069666	-2.43941092	1.01752913
6	-1.62750340	-1.06971085	-1.46827090	1	5.04519653	-0.77591091	1.57592905
6	-1.11350346	-0.69511092	-2.86557078	1	4.05129671	-2.06201100	2.25532913
6	-0.12470347	-0.25831091	0.44332910	1	4.89409637	-1.74231088	-1.38177097

6	-1.74220347	2.38178921	0.44512910	1	3.46369648	-0.84561092	-1.86577094
1	-1.30310345	1.70998919	-1.53617096	1	4.70739651	-0.06891092	-0.87687093
1	-0.69290346	2.53258920	0.72352904	1	3.53549671	-3.58841085	-0.30687094
6	-2.33270335	3.72378922	0.02652907	1	2.27109671	-3.20501089	0.85732907
1	-2.26080346	2.04058909	1.34732902	1	2.06659651	-2.79671097	-0.85127097
1	-2.70580339	-1.22901082	-1.56167090	1	-2.90120339	1.28008914	-0.99297094
1	-1.20590353	-2.03321099	-1.16327095	1	-1.26650357	-1.53321087	-3.54817080
1	-0.05540346	-0.43431091	-2.87577081	6	-2.20840335	4.78768921	1.10712910
1	-1.65650344	0.16008908	-3.26937079	1	-3.38700342	3.58228922	-0.23287092
6	1.56959653	1.19088912	-0.79427093	1	-1.83450341	4.06668901	-0.88707095
6	1.25219655	-0.19151092	-0.26937091	1	-2.64300346	5.73618937	0.78462905
6	2.39509654	-0.47641093	0.71312904	1	-1.16230345	4.97628927	1.36352909
6	2.97579670	0.90078908	0.96792907	1	-2.72360349	4.48648930	2.02342916
8	2.50149655	1.78738916	0.01862907				

tBuMA--BU2-rad--MA__re

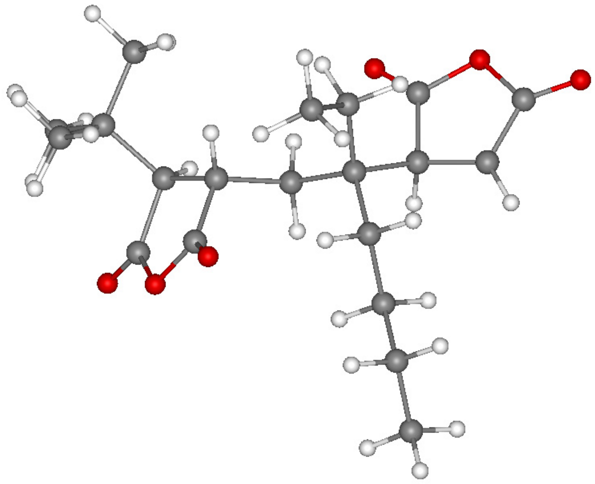

Zero-point vibrational energy	1232815.8 (Joules/Mol)
	294.65005 (Kcal/Mol)
Zero-point correction=	0.469555 (Hartree/Particle)
Thermal correction to Energy=	0.497023
Thermal correction to Enthalpy=	0.497967
Thermal correction to Gibbs Free Energy=	0.409809
Sum of electronic and zero-point Energies=	-1230.462465
Sum of electronic and thermal Energies=	-1230.434997
Sum of electronic and thermal Enthalpies=	-1230.434053
Sum of electronic and thermal Free Energies=	-1230.522210

cartesian

6	-2.47413993	-2.27190542	1.06451821	8	1.18825996	1.90479469	-1.43838179

6	-2.51834011	-0.75930548	0.83861822
6	-3.93303990	-0.56190544	0.46711820
6	-4.62364006	-1.82400537	0.45591819
8	-3.70023990	-2.81590533	0.80791819
8	-1.55794001	-2.94750547	1.41541815
8	-5.76223993	-2.09350538	0.21261820
1	-2.31123996	-0.28380543	1.80781817
1	-4.43883991	0.36979455	0.26741821
6	-1.80983996	1.22439456	-0.55868179
6	-1.44114006	-0.22110543	-0.17958181
6	-1.48433995	-1.13240540	-1.43318188
6	-0.98423994	-0.54790545	-2.75198174
6	-0.09593996	-0.33950543	0.57601821
6	-1.93843997	2.23949456	0.57341820
1	-1.06283998	1.59059465	-1.26328182
1	-0.99993992	2.30859470	1.13451815
6	-2.29274011	3.63079453	0.05621820
1	-2.69914007	1.92899466	1.30061817
1	-2.52344012	-1.44120538	-1.59738183
1	-0.94173992	-2.05660534	-1.20718181
1	0.00766005	-0.10130544	-2.68788171
1	-1.65263999	0.22879456	-3.12618184
6	1.58625996	1.23869467	-0.53568178
6	1.24875998	-0.19020544	-0.16478179
6	2.41106009	-0.58680546	0.76091820
6	2.95605993	0.75179458	1.21041811
8	2.48745990	1.74489462	0.36761820
8	3.68095994	1.01589465	2.11221814
6	3.51205993	-1.49030542	0.12401819
1	2.04836011	-1.10420537	1.65291810
6	4.57675982	-1.81120539	1.17621815
6	2.86545992	-2.80540538	-0.32058179
6	4.16866016	-0.80110544	-1.07368183
1	1.22855997	-0.78330547	-1.07698190
1	-0.06903996	-1.34110534	1.00891817
1	-0.10263996	0.34639454	1.43071818
1	5.33576012	-2.46920538	0.74601823
1	5.07566023	-0.91570544	1.54531813
1	4.14116001	-2.32620549	2.03641820
1	4.91056013	-1.46290541	-1.52578187
1	3.44835997	-0.54780543	-1.85638177
1	4.68856001	0.11569457	-0.78358179
1	3.63386011	-3.49260545	-0.68248177
1	2.34796000	-3.29700541	0.50721818
1	2.14775991	-2.67390537	-1.13338184
1	-2.75024009	1.20089459	-1.12288189
1	-0.94923997	-1.33380544	-3.50958180
6	-2.43054008	4.66199446	1.16671813
1	-3.22684002	3.57859468	-0.51528180
1	-1.52344000	3.95269442	-0.65328181
1	-2.68124008	5.64699459	0.76761824
1	-1.50023997	4.76479483	1.73151815
1	-3.21643996	4.38509464	1.87511814

tBuMA--BU2-rad--MA__si

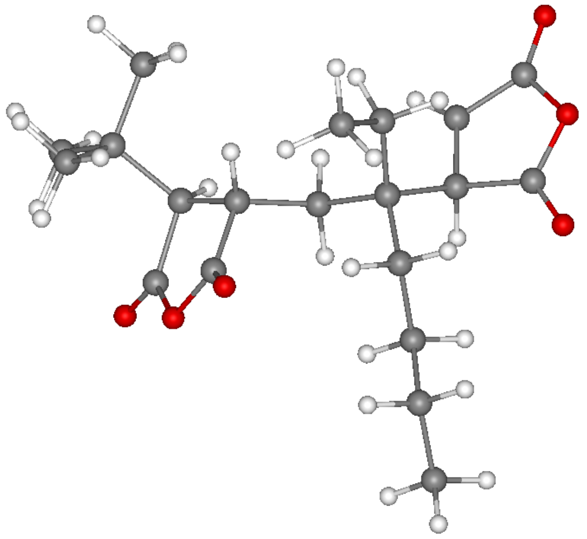

Zero-point vibrational energy	1232124.7 (Joules/Mol)
	294.48487 (Kcal/Mol)
Zero-point correction=	0.469292 (Hartree/Particle)
Thermal correction to Energy=	0.496803
Thermal correction to Enthalpy=	0.497747
Thermal correction to Gibbs Free Energy=	0.409807
Sum of electronic and zero-point Energies=	-1230.459016
Sum of electronic and thermal Energies=	-1230.431504
Sum of electronic and thermal Enthalpies=	-1230.430560
Sum of electronic and thermal Free Energies=	-1230.518501

cartesian

6	-3.77194905	-2.79205441	0.88284725	8	1.18355095	1.90204549	-1.44685268
6	-2.52734900	-2.14555454	1.20574725	8	3.51515102	0.98564547	2.20454717
6	-2.59034896	-0.71175456	0.85384727	6	3.47015095	-1.44705462	0.10644724
6	-4.03994894	-0.58525455	0.38384724	1	1.97255087	-1.15875459	1.62444723
8	-4.63684893	-1.81965446	0.37794724	6	4.54045105	-1.75645447	1.15644729
8	-4.10894918	-3.93425441	0.99044728	6	2.87315106	-2.77285457	-0.37405276
8	-4.63874912	0.39214545	0.07064724	6	4.10955095	-0.70925456	-1.07165277
1	-1.71544898	-2.68675447	1.66644728	1	1.16455102	-0.77495456	-1.09485269
1	-2.48514891	-0.08505455	1.74954724	1	-0.13454905	-1.37945461	0.94984728
6	-1.82384896	1.25414538	-0.58185273	1	-0.17014904	0.28984544	1.44054723
6	-1.51784897	-0.20145455	-0.18935277	1	5.31685114	-2.38835454	0.71834725
6	-1.59324896	-1.12895465	-1.42915273	1	5.01605082	-0.85445458	1.53984725
6	-1.07394910	-0.57875454	-2.75505280	1	4.11745119	-2.29455447	2.00894713
6	-0.17094904	-0.35925457	0.55804724	1	4.88115120	-1.33305454	-1.52795279
6	-1.90884900	2.28334546	0.54054725	1	3.38795090	-0.46925455	-1.85725272
1	-1.06134903	1.57414556	-1.29255271	1	4.59105110	0.22124544	-0.75995272
1	-0.99554908	2.28424549	1.14834726	1	3.66265106	-3.41195440	-0.77595276
6	-2.12784910	3.69314551	-0.00045276	1	2.39975095	-3.31745458	0.44764721
1	-2.73524904	2.03964543	1.21674728	1	2.13235092	-2.64535451	-1.16675270
1	-2.63954902	-1.40925455	-1.59365273	1	-2.76874900	1.27014542	-1.12995279
1	-1.08214903	-2.06995463	-1.19035280	1	-1.06894910	-1.37305450	-3.50465274
1	-0.06654905	-0.16695455	-2.69775271	6	-2.24624896	4.73894548	1.09844720
1	-1.71624899	0.21704546	-3.13365269	1	-3.03474903	3.70134544	-0.61475277
6	1.52635098	1.23834538	-0.52225274	1	-1.30154896	3.95274544	-0.67085272
6	1.17845094	-0.19525456	-0.17435275	1	-2.40324903	5.73724556	0.68424726
6	2.33395100	-0.59925455	0.75744724	1	-1.34164906	4.77794552	1.71194720
6	2.83265090	0.73744541	1.26604724	1	-3.08684897	4.52344561	1.76364720
8	2.37885094	1.74144554	0.43204725				

TS__tBuMA--BU2-rad_BU2

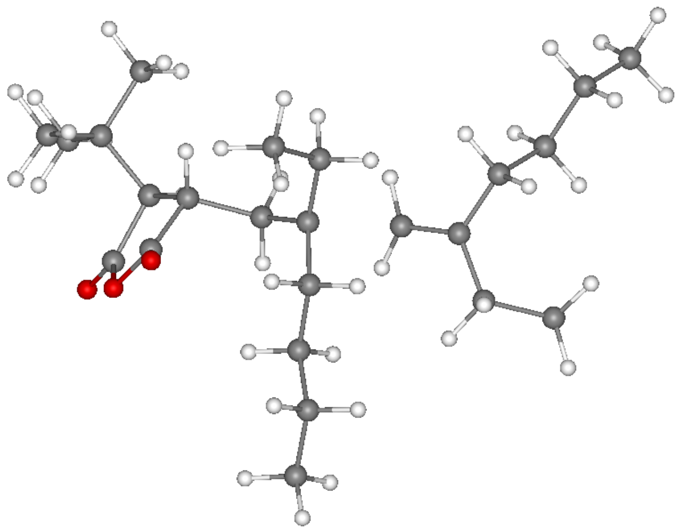

Zero-point vibrational energy	1662413.1 (Joules/Mol)
	397.32626 (Kcal/Mol)
Zero-point correction=	0.633180 (Hartree/Particle)
Thermal correction to Energy=	0.666639
Thermal correction to Enthalpy=	0.667583
Thermal correction to Gibbs Free Energy=	0.564235
Sum of electronic and zero-point Energies=	-1165.422633
Sum of electronic and thermal Energies=	-1165.389174
Sum of electronic and thermal Enthalpies=	-1165.388230
Sum of electronic and thermal Free Energies=	-1165.491578

cartesian

6	-3.72396970	-0.59605849	-0.40034428	1	-0.17726991	-1.56715858	-1.48784435
6	-2.82386994	0.38334149	0.30905572	1	1.53963006	-0.36215848	-3.03064418
6	-1.58087003	0.00494150	0.74985576	1	0.22503006	0.66224152	-3.56384420
1	-1.08156991	0.60344148	1.50455570	6	3.27643013	0.90874153	-0.78244424
6	-3.32386994	1.78844142	0.49595574	6	2.61573005	-0.36395848	-0.31304428
1	-1.33316994	-1.05295849	0.76315570	6	3.56293011	-0.86985850	0.78225577
6	-4.50677013	-1.51895857	0.54395568	6	4.33093023	0.38134152	1.16065574
1	-3.13256979	-1.22925854	-1.07034433	8	4.17122984	1.33574140	0.17695573
1	-4.43267012	-0.05825850	-1.04184425	8	3.11523008	1.52484143	-1.78594434
6	-5.40947008	-2.49805856	-0.19694428	8	5.00143003	0.59884149	2.11685586
1	-5.10647011	-0.92045850	1.23735571	6	4.50913000	-2.04485846	0.38775572
1	-3.79556966	-2.07255864	1.16745567	1	3.00753021	-1.18845856	1.66925573
1	-2.55386972	2.38584137	0.99155569	6	5.36532974	-2.43925858	1.59405565
6	-4.62977028	1.90394151	1.28425574	6	3.65063024	-3.25025845	-0.00524428
1	-3.47866988	2.25144148	-0.48924425	6	5.41933012	-1.65345860	-0.77834427

1	-4.92177010	2.95104146	1.39405572	1	2.53943014	-1.04225850	-1.16134429
1	-4.52647018	1.47784150	2.28485584	1	0.88443005	-1.08365858	0.66455567
1	-5.45307016	1.38824141	0.78495574	1	1.28663003	0.55314147	1.12395573
6	-6.17597008	-3.42665863	0.73425567	1	6.00942993	-3.28235865	1.33165574
1	-4.80546999	-3.09285855	-0.89244425	1	5.99952984	-1.62175858	1.93455565
1	-6.11667013	-1.93835855	-0.82034433	1	4.74193001	-2.74795866	2.43755579
1	-6.81427002	-4.11705828	0.17795572	1	6.04702997	-2.50005865	-1.06574428
1	-6.81797028	-2.86405849	1.41765571	1	4.85512972	-1.35655856	-1.66654432
1	-5.49627018	-4.02715826	1.34505570	1	6.08893013	-0.83075851	-0.51434433
6	0.00793007	1.85924149	-0.96044433	1	4.29003000	-4.11265850	-0.20874427
6	0.10973006	0.38494152	-0.66154432	1	2.96733022	-3.53085852	0.80095577
6	-0.28476992	-0.52175850	-1.80314434	1	3.05633020	-3.07295847	-0.90424430
6	0.45513007	-0.31255847	-3.13084412	1	-1.01616991	2.06134152	-1.29884434
6	1.19183004	-0.11225849	0.25915572	1	0.14723009	-1.07105851	-3.85514426
6	0.37283009	2.83264136	0.15565573	6	0.50153005	5.27164173	0.86025572
1	0.64643008	2.09134150	-1.82314432	1	-0.95137000	4.40724182	-0.47414428
1	1.43293011	2.73184156	0.41455573	1	0.65963006	4.51494169	-1.14894426
6	0.11013007	4.28414154	-0.22964427	1	0.30453008	6.30264187	0.55735576
1	-0.18436995	2.59734154	1.06965566	1	1.56563008	5.19744158	1.09975564
1	-1.35157001	-0.37735850	-2.00134420	1	-0.05556992	5.08884192	1.78365564

TS__tBuMA--BU2-rad_BU2_transfer

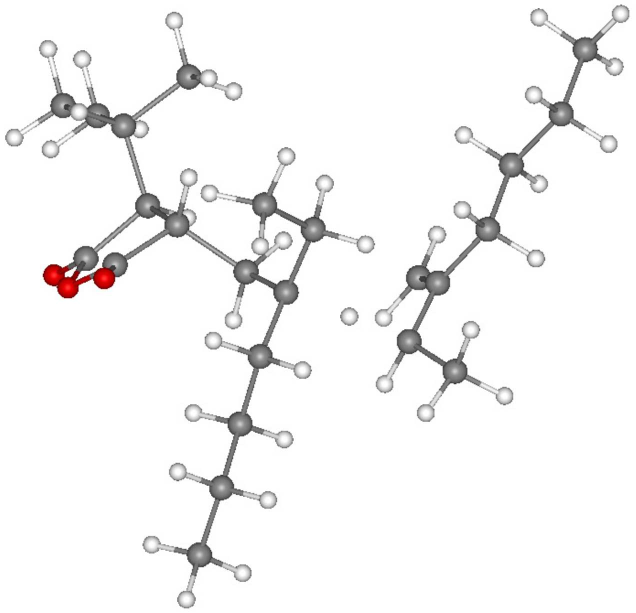

Zero-point vibrational energy 1651613.7 (Joules/Mol)

394.74515 (Kcal/Mol)

Zero-point correction= 0.629066 (Hartree/Particle)

Thermal correction to Energy= 0.662541

Thermal correction to Enthalpy= 0.663485

Thermal correction to Gibbs Free Energy=	0.560560
Sum of electronic and zero-point Energies=	-1165.418143
Sum of electronic and thermal Energies=	-1165.384668
Sum of electronic and thermal Enthalpies=	-1165.383724
Sum of electronic and thermal Free Energies=	-1165.486650

cartesian

XXX	XXX

tBuMA--BU2-rad--BU2

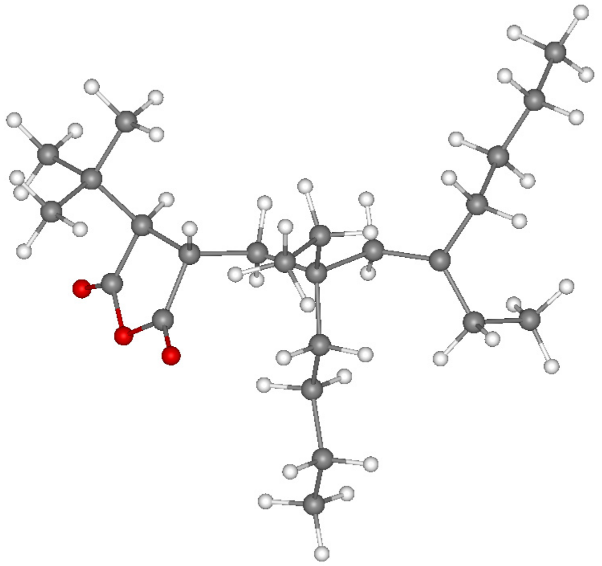

Zero-point vibrational energy	XXX (Joules/Mol)
	XXX (Kcal/Mol)
Zero-point correction	XXX (Hartree/Particle)
Thermal correction to Energy	XXX
Thermal correction to Enthalpy	XXX
Thermal correction to Gibbs Free Energy	XXX
Sum of electronic and zero-point Energies	XXX
Sum of electronic and Thermal Energies	XXX
Sum of electronic and Thermal Enthalpies	XXX
Sum of electronic and Thermal Free Energies	XXX

cartesian

6	0.94682717	2.54200149	-0.85118282	1	1.32862711	-4.79009819	-0.71568280
6	0.24852714	1.21210146	-0.64608282	1	0.49402714	-3.68219852	0.37251717
6	-0.46487287	0.68040144	-1.87998283	1	0.90772712	-3.17709851	-1.27028286
6	0.34492713	0.67220145	-3.17978287	1	0.22522715	3.23320150	-1.30638289
6	0.90102714	0.16380145	0.23121718	1	-0.22007284	0.18150145	-3.97628284
6	1.54362714	3.19520140	0.39151716	6	2.73892713	5.23160172	1.33321714

1	1.74442720	2.42590141	-1.59438288	1	1.30482709	5.22820187	-0.27348280
1	2.35042715	2.57130146	0.79271716	1	2.84632707	4.51440144	-0.69348282
6	2.10492706	4.58440161	0.11031717	1	3.13252711	6.22430182	1.10331714
1	0.78952718	3.26180148	1.18481708	1	3.56822729	4.63020182	1.71491718
1	-1.36197281	1.28690147	-2.05508280	1	2.01532722	5.34530163	2.14551711
1	-0.83217287	-0.33119854	-1.66998291	6	-2.55187273	2.96360135	0.37271714
1	1.30292714	0.16110145	-3.07788277	6	-1.82437277	1.81520140	1.03331721
1	0.56592715	1.68690145	-3.51578283	6	-2.55387282	0.56970143	1.31931710
6	3.26282716	-0.00189855	-0.69018281	6	-3.64787269	0.17330144	0.35741717
6	1.99452722	-0.75609857	-0.37028283	6	-4.30417299	-1.18049860	0.58211720
6	2.45532727	-1.80729854	0.64771718	6	-5.38637304	-1.48829854	-0.44728279
6	3.76332712	-1.23309863	1.15361714	6	-2.22627282	-0.18009855	2.38701701
8	4.19752693	-0.24479856	0.29301718	1	-0.82267284	1.51550138	0.18811718
8	3.51032710	0.71780145	-1.60378289	1	-1.87567282	3.80300140	0.19891717
8	4.39862728	-1.52469862	2.11411715	1	-2.98417282	2.68700147	-0.59128278
6	2.61812711	-3.26149845	0.10931717	1	-3.37107277	3.33350134	1.00031722
1	1.77332723	-1.85579860	1.50161707	1	-3.24677277	0.21220146	-0.66318280
6	3.05382729	-4.18419838	1.25061715	1	-4.42277288	0.95050144	0.38101715
6	1.25932717	-3.74459863	-0.40588284	1	-4.74367285	-1.21669853	1.58531713
6	3.65132713	-3.32049847	-1.01768291	1	-3.54387283	-1.96989858	0.55471718
1	1.61732721	-1.19359851	-1.29398286	1	-6.14597321	-0.69849855	-0.42158282
1	0.11052716	-0.51399857	0.57321721	6	-6.05037308	-2.83999848	-0.22718282
1	1.29712713	0.62750143	1.14081717	1	-6.81947327	-3.03699851	-0.97728282
1	3.13202715	-5.21199846	0.88751721	1	-5.32237291	-3.65409851	-0.28098285
1	4.01872683	-3.89739847	1.66711712	1	-6.52777290	-2.89319849	0.75511718
1	2.32612705	-4.17479849	2.06661701	1	-2.71547294	-1.11829853	2.61721706
1	3.72542715	-4.33849812	-1.40698290	1	-1.45317280	0.14210145	3.07721710
1	3.38782716	-2.67459846	-1.85958290	1	-1.23537278	2.14000154	1.89321709
1	4.64822674	-3.03399849	-0.67228281	1	-4.95207310	-1.45309854	-1.45328283

TS__BU2-allyl_MA

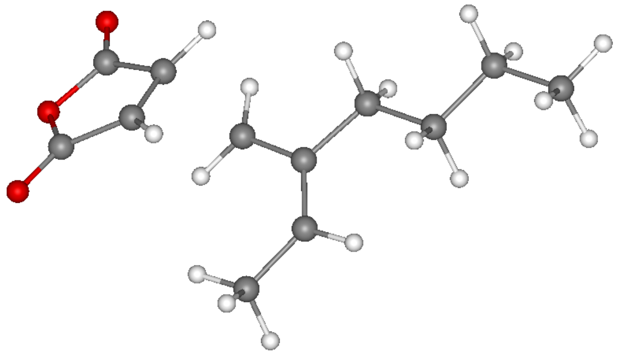

Zero-point vibrational energy 702684.8 (Joules/Mol)

167.94571 (Kcal/Mol)

Zero-point correction=	0.267639 (Hartree/Particle)
Thermal correction to Energy=	0.284144
Thermal correction to Enthalpy=	0.285088
Thermal correction to Gibbs Free Energy=	0.219965
Sum of electronic and zero-point Energies=	-692.832233
Sum of electronic and thermal Energies=	-692.815728
Sum of electronic and thermal Enthalpies=	-692.814784
Sum of electronic and thermal Free Energies=	-692.879906

cartesian

6	4.25250912	0.36779374	-0.82398438	6	1.14790928	2.53549385	0.90241563
6	2.91660929	0.05209373	-1.31568444	1	2.11520910	0.07339373	1.65601552
6	2.46840930	-1.05410624	-0.65318441	1	1.24390924	-1.52060628	1.49501550
6	3.61510921	-1.57210636	0.14121561	6	-2.31409073	-0.03170627	-0.08048439
8	4.62610912	-0.64460623	0.06701561	6	-3.42249084	-1.03210628	-0.39038438
8	4.98030949	1.28039384	-1.06508446	1	-2.10429072	0.56059372	-0.97908437
8	3.71090937	-2.58010626	0.76771563	1	-2.67239070	0.67599374	0.67621559
1	2.40790939	0.66159374	-2.04608440	1	1.36170924	3.13729382	0.01341561
1	1.65300930	-1.70240617	-0.93658441	1	2.08720922	2.09489369	1.23171556
6	1.30910921	-0.48120627	1.19331551	1	0.82060927	3.24209380	1.67281556
6	0.13560927	0.18689373	0.75101560	6	-4.70459080	-0.36870629	-0.87188441
6	-1.03999066	-0.70560628	0.40581560	1	-3.63249087	-1.62900639	0.50441563
6	0.07540929	1.54339361	0.61711562	1	-3.06989074	-1.74150634	-1.14798450
1	-1.27379084	-1.31440616	1.28731561	1	-5.48039055	-1.10650635	-1.08688450
1	-0.71199071	-1.42740631	-0.35448438	1	-4.53649044	0.20699373	-1.78608441
1	-0.86089075	1.97489381	0.27611560	1	-5.10379028	0.31829372	-0.12078439

S13. MA – MB2 reaction profile

TS__tBuMA-rad_MB2__re__mpw

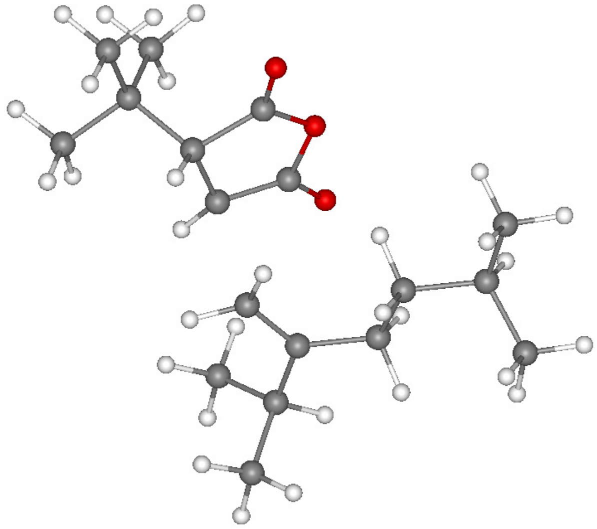

Zero-point vibrational energy	1212630.1 (Joules/Mol)
	289.82556 (Kcal/Mol)
Zero-point correction=	0.461866 (Hartree/Particle)
Thermal correction to Energy=	0.486665
Thermal correction to Enthalpy=	0.487609
Thermal correction to Gibbs Free Energy=	0.405822
Sum of electronic and zero-point Energies=	-929.739160
Sum of electronic and thermal Energies=	-929.714362
Sum of electronic and thermal Enthalpies=	-929.713418
Sum of electronic and thermal Free Energies=	-929.795205

cartesian

6	2.46162701	0.22725388	-0.30350959	1	-1.66297305	1.49795389	-0.65400958
6	1.45112705	1.23935390	0.14589038	1	-0.26307306	1.69025397	1.32559037
6	1.73732698	2.66245389	-0.28310961	1	0.27172697	-0.06084611	1.34439039
6	0.52782691	3.38655376	-0.86890960	1	3.45512700	0.64585388	-0.09660961
6	0.34062696	0.91165394	0.87229037	6	3.05292702	-3.58334613	0.17379040
6	2.35242701	-1.17954612	0.26659039	1	3.29082704	-2.14994621	-1.38990963
1	2.40242696	0.17915389	-1.40070963	6	4.81342697	-1.80094600	0.07219039
1	1.35672700	-1.57924604	0.05449039	1	0.81562692	4.37955379	-1.22250962
6	3.37882710	-2.16864610	-0.29560959	1	2.65062690	4.45045376	0.55209041
1	2.44852710	-1.14804614	1.35979044	1	1.62482703	3.57325387	1.69249046
1	2.49492693	2.60805392	-1.07360959	1	3.22742701	2.95425391	1.29159045
6	2.34682703	3.45315385	0.88059044	1	5.51852703	-2.53784609	-0.32040960
1	-0.26207304	3.52805376	-0.12560961	1	5.11232710	-0.82814610	-0.32430959

1	0.11472695	2.83395386	-1.71420956	1	4.94232702	-1.77184606	1.15929043
6	-0.79657304	-0.31214613	-1.50210953	1	3.74792695	-4.31284618	-0.24940962
6	-1.44037306	0.45955390	-0.46830961	1	3.12012696	-3.66024613	1.26379037
6	-2.29757309	-0.45094612	0.34599039	1	2.04202700	-3.87914610	-0.11690961
6	-1.80897295	-1.81754613	-0.12000960	1	-5.29667282	1.29015398	0.53029037
8	-0.99907309	-1.67154622	-1.21410954	1	-3.87767291	1.40345383	1.56979048
8	-0.14417306	0.01715389	-2.45020962	1	-3.78457308	1.90105391	-0.12620960
8	-2.04637313	-2.89134622	0.33699039	1	-5.33257294	-0.36854613	-1.39730954
6	-3.84357309	-0.27024612	0.17059040	1	-3.74387312	0.12355390	-1.97790956
1	-2.09557295	-0.37534612	1.42109048	1	-4.05947304	-1.56954622	-1.57450962
6	-4.57347298	-1.23394608	1.10799038	1	-5.65437317	-1.10864615	1.00309038
6	-4.21167278	1.16355395	0.55639040	1	-4.32787323	-2.27394629	0.89629042
6	-4.25997305	-0.53714609	-1.27640963	1	-4.31717300	-1.03814614	2.15309024

TS__tBuMA-rad_MB2__si

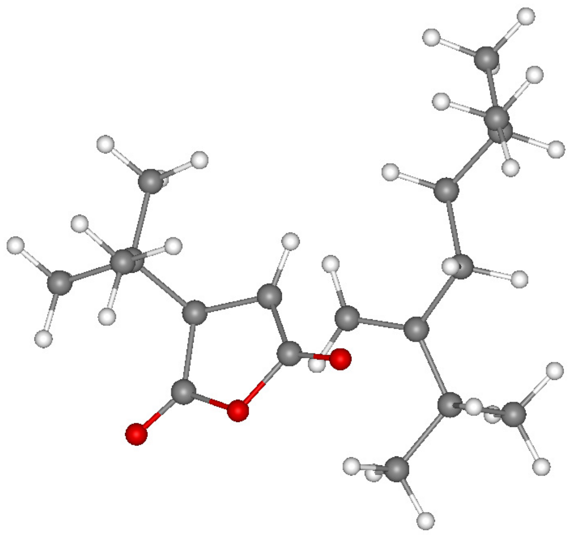

Zero-point vibrational energy 1212094.4 (Joules/Mol)
1212094.4 (Kcal/Mol)

Zero-point correction= 0.461662 (Hartree/Particle)

Thermal correction to Energy= 0.486537

Thermal correction to Enthalpy= 0.487481

Thermal correction to Gibbs Free Energy= 0.405166

Sum of electronic and zero-point Energies= -929.738531

Sum of electronic and thermal Energies= -929.713656

Sum of electronic and thermal Enthalpies= -929.712712

Sum of electronic and thermal Free Energies= -929.795027

cartesian

6	-2.55499077	3.55889821	0.72900957	1	-3.12949085	-3.45760179	0.63750958

6	-1.37349069	2.95099831	-0.04439040	1	-4.17739105	-2.50500178	1.69150960
6	-1.23949075	1.48529816	0.29540962	6	1.38660932	1.00099814	-1.44149029
6	-2.26469088	0.58089817	-0.32779038	6	1.27240920	0.00809816	-0.40219039
6	-2.52729082	-0.74880189	0.37100959	6	2.55730915	-0.04560184	0.35490960
6	-3.70629072	-1.53950191	-0.20469040	6	3.25830936	1.20829821	-0.15599039
6	-0.19909072	0.98449814	1.02630961	8	2.56530905	1.72869825	-1.21579039
6	-0.12559071	3.79319811	0.19150959	8	0.66060925	1.28579807	-2.34939051
1	-1.61619067	3.00279832	-1.11419034	8	4.25580931	1.71999812	0.24500962
1	-2.71669054	4.59389782	0.41910958	6	3.41330910	-1.34200191	0.15820961
1	-3.48909092	3.01839828	0.56640959	1	2.41770935	0.07379815	1.43590963
1	-2.35139084	3.55889821	1.80290961	6	4.67090893	-1.25570178	1.02530968
1	0.73890924	3.42509818	-0.36059040	6	2.58550930	-2.54450178	0.61610961
1	-0.30499071	4.81879807	-0.13789040	6	3.80590916	-1.51460195	-1.30949032
1	0.13650928	3.84239817	1.25230968	1	0.63130927	-0.84320182	-0.57199037
1	-1.92759073	0.40879816	-1.35929036	1	-0.26459074	-0.00120184	1.47030962
1	-3.21049070	1.12439823	-0.42999041	1	0.51280928	1.65499806	1.49410963
1	-2.71609068	-0.56000185	1.43400967	1	3.18970919	-3.45380187	0.56840956
1	-1.63409078	-1.38510180	0.33080959	1	2.24910927	-2.42810178	1.65020967
1	-4.59339094	-0.89310187	-0.15889040	1	1.70680928	-2.70880175	-0.01129040
6	-3.48609066	-1.93430185	-1.66209030	1	4.37760925	-2.43640184	-1.44109035
6	-3.98309088	-2.77250171	0.64960957	1	2.93090916	-1.57640195	-1.96209037
1	-4.32949066	-2.51590180	-2.04229045	1	4.43280935	-0.69160187	-1.66209030
1	-3.36969090	-1.06760192	-2.31549048	1	5.26330900	-2.16730189	0.91150957
1	-2.58839083	-2.55220175	-1.76829040	1	5.29620934	-0.40550184	0.75590956
1	-4.85099077	-3.32510185	0.28160959	1	4.41440916	-1.15650177	2.08380961

TS__tBuMA-rad_to_MB2_transfer

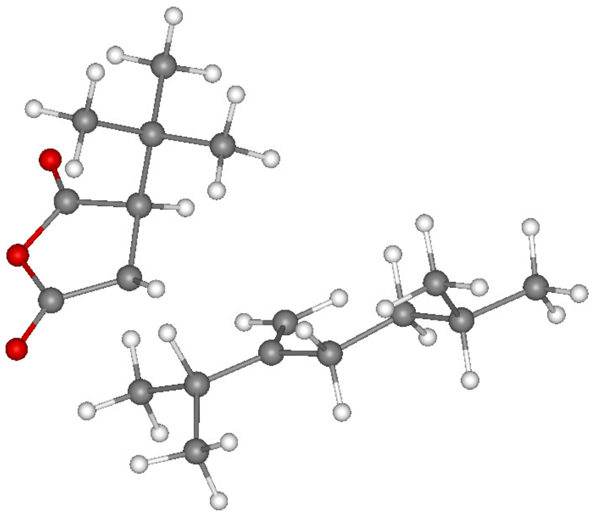

Zero-point vibrational energy 1199170.0 (Joules/Mol)
1199170.0 (Kcal/Mol)

Zero-point correction=	0.456740 (Hartree/Particle)
Thermal correction to Energy=	0.481903
Thermal correction to Enthalpy=	0.482847
Thermal correction to Gibbs Free Energy=	0.399102
Sum of electronic and zero-point Energies=	-929.732579
Sum of electronic and thermal Energies=	-929.707416
Sum of electronic and thermal Enthalpies=	-929.706472
Sum of electronic and thermal Free Energies=	-929.790217

cartesian

6	-0.05053264	-3.12494993	1.21498084	1	-4.69743252	1.87035012	-1.03971922
6	0.29896736	-2.40624976	-0.07451925	1	-5.53883266	0.32655013	-1.19461918
6	-0.80933261	-1.70894992	-0.77481925	6	3.15816736	-1.15734982	1.06248081
6	-1.85833251	-1.06454992	0.10248075	6	1.91996741	-0.41924986	0.84208071
6	-2.91493273	-0.22154987	-0.59741926	6	2.13536739	0.50415015	-0.32131925
6	-3.98853254	0.34645015	0.33738074	6	3.42956734	-0.04484987	-0.90811926
6	-0.85473263	-1.62524986	-2.11451936	8	3.98876715	-0.94754988	-0.04061925
6	1.27206731	-3.19024992	-0.92881924	8	3.49166727	-1.88124990	1.95208085
1	1.02996731	-1.43444991	0.35898075	8	3.94786739	0.21565014	-1.94741917
1	0.85576737	-3.46134996	1.72068083	6	2.22856736	2.02745008	0.01698074
1	-0.60423267	-2.49824977	1.91438079	1	1.35546732	0.39235014	-1.08451915
1	-0.66333258	-4.00944996	1.00618076	6	2.39416742	2.82515001	-1.27791917
1	1.71436739	-2.59034991	-1.72591925	6	0.92406738	2.45965004	0.68968076
1	2.08846736	-3.58104992	-0.31951925	6	3.40716743	2.30455017	0.95138073
1	0.77436739	-4.04564953	-1.39751923	1	1.37166739	-0.12104987	1.72738075
1	-1.34123266	-0.45934987	0.85708076	1	-1.64763272	-1.09584987	-2.62711930
1	-2.36503267	-1.85274982	0.67288071	1	-0.11433262	-2.09584999	-2.74801922
1	-3.41483259	-0.83284986	-1.35651922	1	0.92636740	3.54085016	0.84798074
1	-2.43763256	0.60535014	-1.13861918	1	0.05676740	2.21935010	0.06878074
1	-4.43813276	-0.49694988	0.87898076	1	0.78116739	1.99385023	1.66728079
6	-3.42093277	1.31875014	1.36758077	1	3.43676758	3.36355019	1.21808076
6	-5.09063292	1.01815009	-0.47661927	1	3.33476734	1.73595011	1.88248074
1	-4.21413279	1.72545016	1.99948084	1	4.36396742	2.06235003	0.48178071
1	-2.69223261	0.84725010	2.03018069	1	2.44436741	3.89355016	-1.05261922
1	-2.92753267	2.16415024	0.87718076	1	3.29876757	2.54745007	-1.81741917
1	-5.89073277	1.39205015	0.16698074	1	1.54586744	2.66685009	-1.94961917

tBuMA--MB2-rad__re

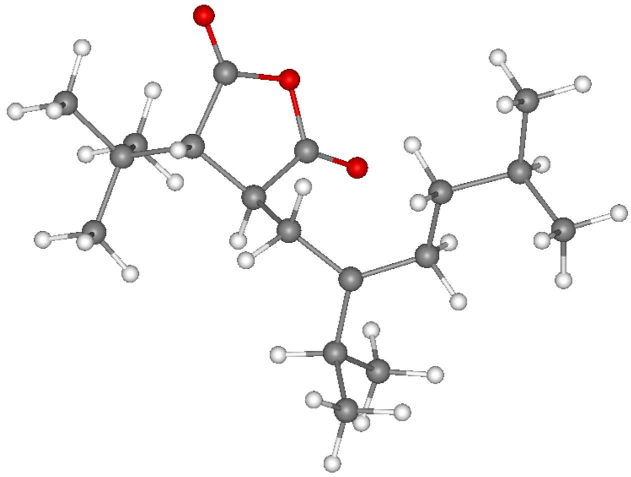

Zero-point vibrational energy	1220007.4 (Joules/Mol)
	291.58878 (Kcal/Mol)
Zero-point correction=	0.464676 (Hartree/Particle)
Thermal correction to Energy=	0.489545
Thermal correction to Enthalpy=	0.490489
Thermal correction to Gibbs Free Energy=	0.407839
Sum of electronic and zero-point Energies=	-929.771076
Sum of electronic and thermal Energies=	-929.746208
Sum of electronic and thermal Enthalpies=	-929.745263
Sum of electronic and thermal Free Energies=	-929.827913

cartesian

6	2.27106166	0.20739612	-0.33399999	1	-1.61353827	1.02789617	-0.67189997
6	1.05606174	0.95289612	0.12710002	1	-0.53413826	0.89169610	1.53800011
6	0.97586179	2.43799591	-0.11279999	1	0.12796172	-0.68520391	1.16269994
6	1.82856178	3.21719599	0.89899999	1	3.14796185	0.84359610	-0.16450000
6	-0.13383827	0.27949613	0.71880001	6	3.81306195	-3.30330372	0.33570001
6	2.50926185	-1.16070390	0.30000001	1	3.76766181	-1.89160395	-1.26609993
1	2.22456169	0.07489613	-1.42599988	6	5.05356169	-1.12770391	0.26340002
1	1.66456175	-1.81940389	0.06920001	1	1.72256172	4.29559612	0.75090003
6	3.78796172	-1.86340392	-0.16849999	1	1.17806172	3.89759588	-1.71079993
1	2.53536177	-1.06750393	1.39380002	1	2.40086174	2.63119602	-1.75429988
1	-0.06633827	2.74519634	0.05020000	1	0.75656176	2.27259588	-2.27660012
6	1.34646177	2.82999611	-1.54640007	1	5.94836140	-1.66610384	-0.05889999
1	2.88876176	2.97379589	0.79170001	1	5.11616135	-0.12130389	-0.15499999
1	1.53826177	2.98929596	1.92729998	1	5.09976149	-1.03820395	1.35369992
6	-0.93873823	-0.83810389	-1.39429998	1	4.70086145	-3.83420372	-0.01699999
6	-1.33313823	0.06159613	-0.25180000	1	3.82466197	-3.33570385	1.42989993

6	-2.49873829	-0.67250389	0.41589999	1	2.93716168	-3.86260366	-0.00240000
6	-2.38143826	-2.07680392	-0.14719999	1	-5.25453854	0.42879611	-1.43389988
8	-1.51483822	-2.07870388	-1.22090006	1	-3.55953813	0.54569614	-1.88330007
8	-0.23103827	-0.61220390	-2.32210016	1	-4.32823849	-1.03830385	-1.71779990
8	-2.91523814	-3.07850409	0.20360002	1	-4.92423868	1.79189622	0.67339998
6	-3.91483831	-0.06130388	0.19490001	1	-3.65263820	1.37049615	1.81850004
1	-2.34643817	-0.74890387	1.49720001	1	-3.24033833	2.03189611	0.23190001
6	-4.95673847	-0.88470387	0.95660001	1	-5.94613838	-0.43700388	0.83300000
6	-3.92343831	1.36209607	0.75910002	1	-5.00373840	-1.91490388	0.60619998
6	-4.27463865	-0.03280389	-1.29229999	1	-4.73543835	-0.90910387	2.02709985

tBuMA--MB2-rad__si

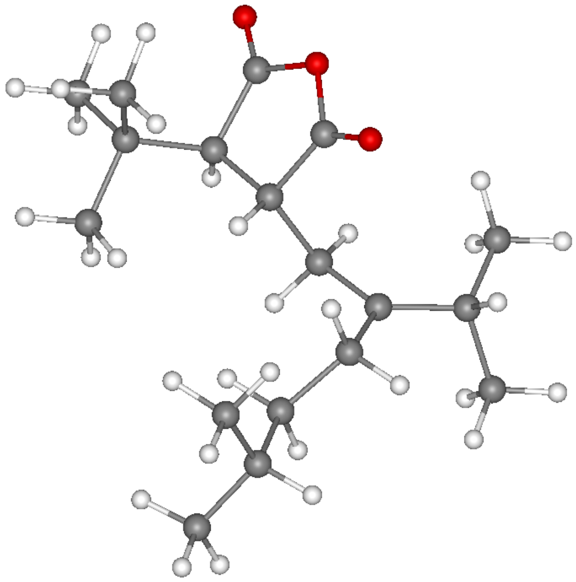

Zero-point vibrational energy	1219521.5 (Joules/Mol)
	291.47263 (Kcal/Mol)
Zero-point correction=	0.464491 (Hartree/Particle)
Thermal correction to Energy=	0.489392
Thermal correction to Enthalpy=	0.490336
Thermal correction to Gibbs Free Energy=	0.406771
Sum of electronic and zero-point Energies=	-929.770849
Sum of electronic and thermal Energies=	-929.745948
Sum of electronic and thermal Enthalpies=	-929.745004
Sum of electronic and thermal Free Energies=	-929.828569

cartesian

6	-3.11660004	2.95269990	-1.14789999	1	-3.31110001	-3.58279991	-1.07299995
6	-1.96159995	2.72580004	-0.15820000	1	-4.58780003	-2.51740003	-1.66470003
6	-1.50230002	1.29240000	-0.18920000	6	1.18799996	1.05350006	1.23880005

6	-2.36409998	0.26570001	0.47979999	6	0.78079998	0.14100000	0.11190000
6	-2.72740006	-0.94379997	-0.38900000	6	2.09780002	-0.20000000	-0.58929998
6	-3.73410010	-1.90750003	0.24560000	6	3.05310011	0.84670001	-0.04460000
6	-0.22910000	0.85630000	-0.83039999	8	2.48550010	1.47730005	1.04229999
6	-0.86820000	3.76640010	-0.38110000	8	0.54930001	1.43079996	2.16739988
1	-2.36770010	2.89849997	0.84909999	8	4.14099979	1.14330006	-0.41909999
1	-3.52239990	3.96379995	-1.05060005	6	2.63350010	-1.64979994	-0.38650000
1	-3.93589997	2.24970007	-0.98159999	1	2.02239990	-0.03340000	-1.66859996
1	-2.77460003	2.82719994	-2.17910004	6	3.92490005	-1.83840001	-1.18659997
1	-0.03580000	3.63980007	0.31400001	6	1.59010005	-2.63380003	-0.92210001
1	-1.27020001	4.77040005	-0.22730000	6	2.90260005	-1.93340003	1.09300005
1	-0.47380000	3.73760009	-1.40100002	1	0.28790000	-0.72740000	0.54970002
1	-1.86800003	-0.07560000	1.40040004	1	-0.42620000	0.13800000	-1.63900006
1	-3.28999996	0.74379998	0.81819999	1	0.27980000	1.69970000	-1.30359995
1	-3.13770008	-0.58080000	-1.33870006	1	4.28730011	-2.86310005	-1.07089996
1	-1.82290006	-1.50779998	-0.65210003	1	4.71280003	-1.16030002	-0.86199999
1	-4.62419987	-1.32420003	0.51859999	1	3.75799990	-1.66470003	-2.25320005
6	-3.19549990	-2.56299996	1.51400006	1	3.24219990	-2.96350002	1.22309995
6	-4.16270018	-2.96799994	-0.76410002	1	2.00889993	-1.81029999	1.71070004
1	-3.92510009	-3.25839996	1.93659997	1	3.68300009	-1.28299999	1.49670005
1	-2.95630002	-1.83350003	2.28990006	1	1.97070003	-3.65560007	-0.85280001
1	-2.28579998	-3.13490009	1.30190003	1	1.36290002	-2.43869996	-1.97370005
1	-4.91470003	-3.63969994	-0.34250000	1	0.65249997	-2.60190010	-0.36309999

TS__tBuMA--MB2-rad_MA__re

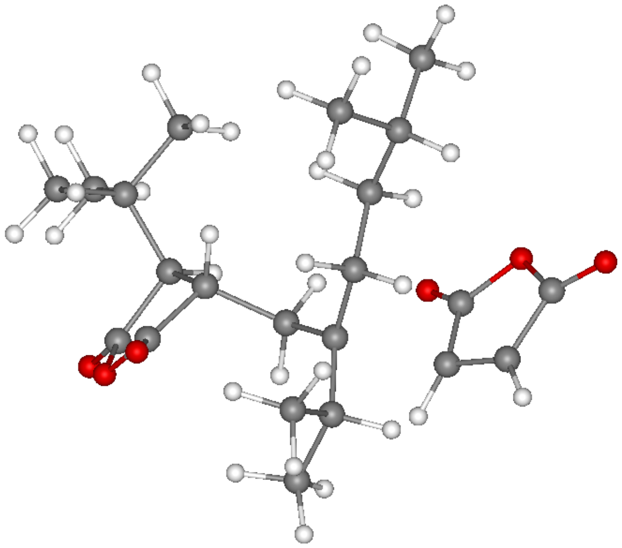

Zero-point vibrational energy	1374964.1 (Joules/Mol)
	328.62431 (Kcal/Mol)
Zero-point correction=	0.523696 (Hartree/Particle)

Thermal correction to Energy= 0.553930
Thermal correction to Enthalpy= 0.554874
Thermal correction to Gibbs Free Energy= 0.460373
Sum of electronic and zero-point Energies= -1308.990916
Sum of electronic and thermal Energies= -1308.960683
Sum of electronic and thermal Enthalpies= -1308.959739
Sum of electronic and thermal Free Energies= -1309.054240

cartesian

6	2.32434583	-0.51837867	2.47493768	6	-4.26935434	1.63592136	1.97453773
6	2.39754581	-1.69257867	1.56973767	6	-2.27645421	2.51692128	0.78943765
6	3.68044591	-1.70057869	1.06793773	6	-4.07725430	1.35202134	-0.50306237
6	4.34994602	-0.47647867	1.47763765	1	-1.21125424	0.44672132	-0.64996231
8	3.48084593	0.20902133	2.33693767	1	0.04234580	-0.21187867	1.40833771
8	1.44994581	-0.18267867	3.21643782	1	-0.59425420	-1.82407868	1.31193769
8	5.42944574	-0.04257867	1.21483767	1	-4.77875423	2.59122133	1.82663774
1	1.79414570	-2.56017876	1.78793776	1	-5.02795410	0.85852134	2.06023765
1	4.17684603	-2.45017862	0.47073770	1	-3.73865414	1.68752134	2.92883778
6	1.07524574	-2.78447866	-0.69576228	1	-4.56555414	2.31452131	-0.67046237
6	0.92754585	-1.36777866	-0.13346231	1	-3.42965412	1.16892135	-1.36526227
6	1.56554580	-0.29507867	-0.97036231	1	-4.86065435	0.58972132	-0.50176227
6	1.64944577	1.12092137	-0.40626231	1	-2.79035401	3.47892141	0.72763765
6	-0.26725420	-0.98107868	0.69853771	1	-1.66645420	2.52732134	1.69643772
6	0.20424581	-3.83667874	-0.01276231	1	-1.60425425	2.46112132	-0.06966233
6	0.89344585	-2.86847878	-2.22176218	1	2.12114596	-3.06657863	-0.52586234
1	-0.85675418	-3.69347858	-0.22646233	6	2.47884583	2.07732129	-1.27106225
1	0.32394579	-3.86157870	1.07343769	1	0.46974578	-4.82897854	-0.38186231
1	0.99874580	-0.26797867	-1.91146231	1	1.03294575	-3.90547872	-2.53556228
1	2.56874585	-0.63147867	-1.26026225	1	1.63374579	-2.27057862	-2.75416231
1	2.08794594	1.10812128	0.59233767	1	-0.09945420	-2.55327868	-2.53636241
1	0.64734584	1.54632127	-0.28216234	1	3.47684598	1.63502133	-1.38946223
6	-2.27795410	-1.37147868	-0.83676231	6	2.64724588	3.41452122	-0.55646229
6	-1.51765418	-0.37167865	-0.00076231	6	1.88124573	2.27992129	-2.66036224
6	-2.54975414	0.03582132	1.06213772	1	3.27394581	4.09632111	-1.13586235
6	-3.49435401	-1.14927864	1.06613767	1	3.11274600	3.28822136	0.42363769
8	-3.32345414	-1.87477863	-0.10266232	1	1.68024576	3.90522146	-0.40586230
8	-2.07895422	-1.72967863	-1.95356226	1	2.47964597	2.98412132	-3.24316239
8	-4.29785395	-1.48107862	1.87293768	1	0.86794585	2.69052124	-2.59516239
6	-3.30195403	1.38052130	0.81573772	1	1.82804573	1.35272133	-3.23516226
1	-2.09615421	0.09652132	2.05493760				

TS__tBuMA--MB2-rad_MA__si

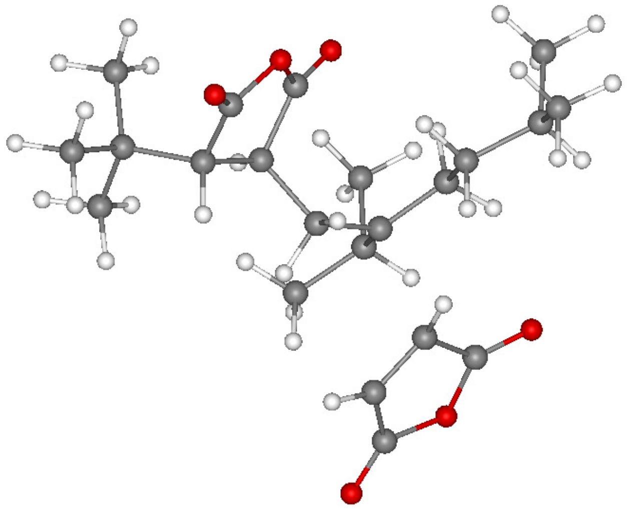

Zero-point vibrational energy	1374435.0 (Joules/Mol)
	1374435.0 (Kcal/Mol)
Zero-point correction=	0.523495 (Hartree/Particle)
Thermal correction to Energy=	0.553784
Thermal correction to Enthalpy=	0.554729
Thermal correction to Gibbs Free Energy=	0.460358
Sum of electronic and zero-point Energies=	-1308.993260
Sum of electronic and thermal Energies=	-1308.962971
Sum of electronic and thermal Enthalpies=	-1308.962026
Sum of electronic and thermal Free Energies=	-1309.056397

cartesian

6	-3.58837032	-2.93571305	1.14740825	1	2.05442953	-1.22341299	1.65770817
6	-2.61437035	-2.06051302	1.78640819	6	4.63802958	-1.82601297	1.32890821
6	-2.79917049	-0.78581303	1.31430817	6	3.05042958	-2.93521309	-0.22249183
6	-4.03617048	-0.82091302	0.49670818	6	4.30002928	-0.89541304	-0.96639186
8	-4.41227055	-2.13391304	0.35090816	1	1.33912945	-0.99791306	-1.11369181
8	-3.75287032	-4.11431313	1.22260809	1	-0.13007051	-1.54981303	0.78720820
8	-4.65267086	0.07328697	0.00240818	1	-0.04757050	0.09388698	1.38340807
1	-1.90407050	-2.42291307	2.51380825	1	5.44112921	-2.46651292	0.95660818
1	-2.49417043	0.12428697	1.80410814	1	5.08532953	-0.90081304	1.69070816
6	-1.69917059	1.28098702	-0.46649182	1	4.18212938	-2.33021307	2.18500829
6	-1.29407048	-0.16271302	-0.33369184	1	5.09402943	-1.53401303	-1.35919189
6	-1.66967058	-1.05071306	-1.51169181	1	3.61102962	-0.69861305	-1.79239178
6	-1.17077053	-0.48261303	-2.85589170	1	4.76212931	0.05248698	-0.67899179
6	-0.06727050	-0.50621301	0.46850818	1	3.86342978	-3.59121299	-0.54179180
6	-1.35717058	2.24478698	0.66810817	1	2.52932954	-3.43581295	0.59790814
1	-1.21537054	1.65588701	-1.37589192	1	2.35782957	-2.85621309	-1.06349182

1	-0.27207050	2.37618709	0.74770820	1	-2.77407050	1.32028699	-0.67629182
6	-1.99527049	3.62908697	0.50380814	1	-1.40187049	-1.19351292	-3.65219188
1	-1.67917049	1.84608698	1.63690829	6	-1.68277049	4.49608660	1.71960807
1	-2.76237035	-1.03631306	-1.58829188	1	-3.08287048	3.48718691	0.45770818
6	-1.22997046	-2.50791287	-1.38189173	6	-1.55077052	4.32618666	-0.77929181
1	-0.09467050	-0.30431303	-2.86159182	1	-2.17217040	5.47018671	1.64730811
1	-1.65897048	0.45848697	-3.10779166	1	-0.60677052	4.67498684	1.80820823
6	1.63982952	1.04168701	-0.63089180	1	-2.01867056	4.02678680	2.64820814
6	1.32122946	-0.37751302	-0.21979183	1	-1.69727051	-3.10541296	-2.16629171
6	2.44302964	-0.71271306	0.77230817	1	-1.50897050	-2.95821309	-0.42929184
6	2.91122961	0.66038698	1.21240807	1	-0.14887050	-2.62261295	-1.49709177
8	2.47092962	1.60888708	0.30530816	1	-1.96657050	5.33498669	-0.83539182
8	1.27292943	1.65138698	-1.58339190	1	-1.87567055	3.79488707	-1.67599177
8	3.56402969	0.97468692	2.15170813	1	-0.46097052	4.41328669	-0.82319182
6	3.61442947	-1.58131301	0.21680817				

tBuMA--MB2-rad--MA__re

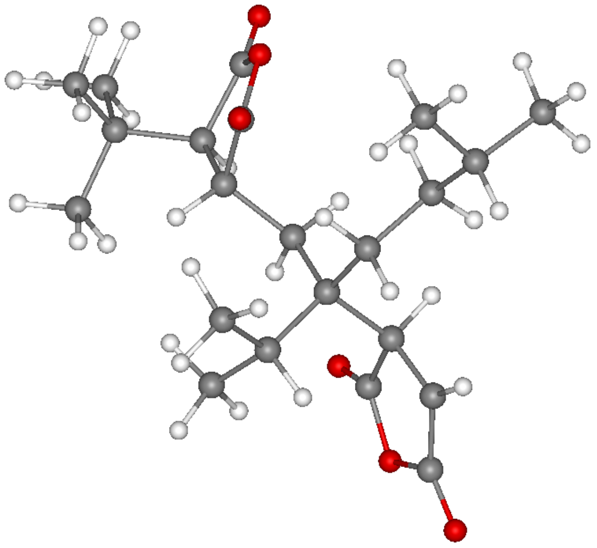

Zero-point vibrational energy	1381150.7 (Joules/Mol)
	330.10294 (Kcal/Mol)
Zero-point correction=	0.526053 (Hartree/Particle)
Thermal correction to Energy=	0.556252
Thermal correction to Enthalpy=	0.557196
Thermal correction to Gibbs Free Energy=	0.463531
Sum of electronic and zero-point Energies=	-1309.021623
Sum of electronic and thermal Energies=	-1308.991424
Sum of electronic and thermal Enthalpies=	-1308.990480
Sum of electronic and thermal Free Energies=	-1309.084145

cartesian

6	-2.28520012	-2.05979991	-1.21550000
6	-2.29909992	-0.62050003	-0.70380002
6	-3.69829988	-0.48469999	-0.25400001
6	-4.39289999	-1.73469996	-0.41220000
8	-3.49620008	-2.64890003	-0.97740000
8	-1.41620004	-2.64150000	-1.78579998
8	-5.51650000	-2.04670000	-0.15040000
1	-2.12389994	-0.00320000	-1.59490001
1	-4.18779993	0.39809999	0.12670000
6	-1.45239997	1.31389999	0.64740002
6	-1.19669998	-0.18200000	0.34599999
6	-1.39610004	-1.00870001	1.66190004
6	-0.90460002	-2.45650005	1.59570003
6	0.15520000	-0.42350000	-0.36300001
6	-1.40559995	2.29850006	-0.51959997
1	-0.38870001	2.36080003	-0.92540002
1	-2.03760004	1.95949996	-1.34860003
6	-0.84050000	-0.35089999	2.92960000
1	-2.48460007	-1.05289996	1.80900002
1	-1.29260004	-3.02440000	0.75169998
1	0.18529999	-2.52010012	1.55219996
6	1.83860004	1.23819995	0.64660001
6	1.51279998	-0.20800000	0.34410000
6	2.64479995	-0.60240000	-0.62190002
6	3.05949998	0.73470002	-1.19959998
8	2.61290002	1.74839997	-0.36790001
8	1.53450000	1.90910006	1.58099997
8	3.67969990	0.98329997	-2.18000007
6	3.85310006	-1.36740005	0.00130000
1	2.26710010	-1.21309996	-1.44570005
6	4.85580015	-1.70799994	-1.10459995
6	3.34540009	-2.67820001	0.60799998
6	4.54610014	-0.53359997	1.08140004
1	1.54260004	-0.76400000	1.27820003
1	0.17309999	-1.46060002	-0.69819999
1	0.16110000	0.16419999	-1.28830004
1	5.69610023	-2.26589990	-0.68409997
1	5.25199986	-0.81790000	-1.59230006
1	4.39690018	-2.33179998	-1.87619996
1	5.36250019	-1.10520005	1.52830005
1	3.86910009	-0.25540000	1.89400005
1	4.98050022	0.38389999	0.67600000
1	4.19029999	-3.27830005	0.95370001
1	2.79719996	-3.27430010	-0.12620001
1	2.69460011	-2.52090001	1.47119999
1	-2.42499995	1.40059996	1.14649999
6	-1.86160004	3.71690011	-0.15140000
1	-0.97530001	-1.02970004	3.77489996
1	0.22270000	-0.11680000	2.86430001
1	-1.35599995	0.57580000	3.18099999
1	-1.21570003	-2.98609996	2.49850011
1	-0.72340000	1.63129997	1.38909996
6	-1.90059996	4.59410000	-1.39919996
1	-2.88639998	3.64369988	0.23930000
6	-0.99010003	4.35720015	0.92490000
1	-2.27259994	5.59499979	-1.16789997
1	-0.90079999	4.70769978	-1.82920003
1	-2.54800010	4.17150021	-2.17249990
1	-1.32400000	5.37620020	1.13549995
1	-1.01429999	3.80579996	1.86640000
1	0.05460000	4.40929985	0.60630000

tBuMA--MB2-rad--MA__si

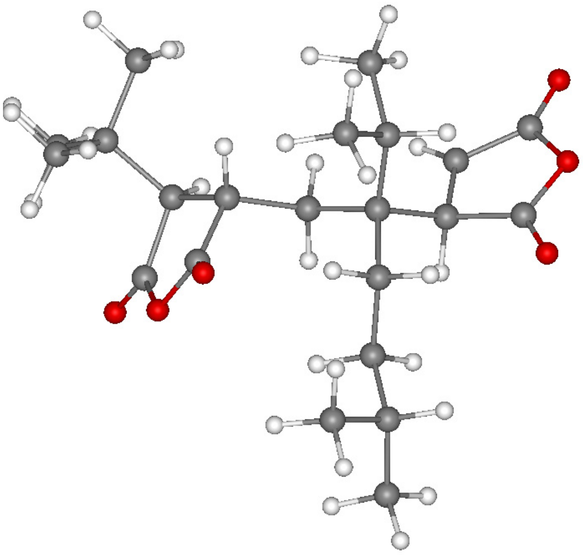

Zero-point vibrational energy	1381504.3 (Joules/Mol)
	330.18745 (Kcal/Mol)
Zero-point correction=	0.526187 (Hartree/Particle)
Thermal correction to Energy=	0.556215
Thermal correction to Enthalpy=	0.557159
Thermal correction to Gibbs Free Energy=	0.464160
Sum of electronic and zero-point Energies=	-1309.019822
Sum of electronic and thermal Energies=	-1308.989794
Sum of electronic and thermal Enthalpies=	-1308.988850
Sum of electronic and thermal Free Energies=	-1309.081850

cartesian

6	-3.46630001	-2.73140001	-1.03840005	1	2.30699992	-1.27460003	-1.46130002
6	-2.20280004	-2.07690001	-1.25139999	6	4.91200018	-1.69540000	-1.13230002
6	-2.24909997	-0.69639999	-0.73110002	6	3.43530011	-2.67459989	0.60490000
6	-3.69740009	-0.60689998	-0.24760000	6	4.58640003	-0.49460000	1.03670001
8	-4.31850004	-1.81649995	-0.41780001	1	1.58539999	-0.78780001	1.25489998
8	-3.82509995	-3.84150004	-1.30120003	1	0.22040001	-1.53460002	-0.67269999
8	-4.27969980	0.32810000	0.19970000	1	0.18380000	0.07990000	-1.32130003
1	-1.40409994	-2.55450010	-1.79779994	1	5.76599979	-2.22959995	-0.70920002
1	-2.15809989	0.00390000	-1.57229996	1	5.28690004	-0.80360001	-1.63360000
6	-1.40550005	1.26450002	0.63110000	1	4.46369982	-2.33890009	-1.89419997
6	-1.16659999	-0.23190001	0.32980001	1	5.42430019	-1.03600001	1.48130000
6	-1.35769999	-1.07110000	1.63810003	1	3.91059995	-0.22650000	1.85339999
6	-0.80280000	-0.42109999	2.91050005	1	4.98859978	0.43040001	0.61559999
6	0.18480000	-0.48260000	-0.38049999	1	4.29519987	-3.24429989	0.96420002
6	-1.31809998	2.24729991	-0.53460002	1	2.90809989	-3.30080009	-0.12010000

1	-0.68480003	1.56739998	1.38720000	1	2.77690005	-2.52010012	1.46280003
1	-0.28909999	2.32049990	-0.90869999	1	-2.39030004	1.36119998	1.09340000
6	-1.80309999	3.65989995	-0.18240000	1	-0.92650002	-1.10969996	3.74970007
1	-1.92349994	1.90610003	-1.38240004	6	-1.78709996	4.54370022	-1.42589998
1	-2.44180012	-1.12820005	1.79729998	1	-2.84389997	3.56979990	0.15470000
6	-0.85119998	-2.51110005	1.54740000	6	-0.99119997	4.30019999	0.93949997
1	0.25740001	-0.17350000	2.84249997	1	-2.17939997	5.54010010	-1.20819998
1	-1.33099997	0.49579999	3.16930008	1	-0.76859999	4.66860008	-1.80639994
6	1.86749995	1.20829999	0.59869999	1	-2.39339995	4.11969995	-2.23110008
6	1.54620004	-0.24390000	0.31410000	1	-1.19400001	-3.07850003	2.41499996
6	2.67689991	-0.63739997	-0.65380001	1	-1.21150005	-3.04469991	0.66460001
6	3.05489993	0.69630003	-1.26580000	1	0.24020000	-2.56879997	1.54250002
8	2.61260009	1.71490002	-0.44220001	1	-1.34189999	5.31619978	1.13769996
8	1.59280002	1.87899995	1.54030001	1	-1.06359994	3.74390006	1.87580001
8	3.64650011	0.93269998	-2.26699996	1	0.06880000	4.36009979	0.67680001
6	3.90739989	-1.36189997	-0.02590000				

TS__tBuMA--MB2-rad_MB2

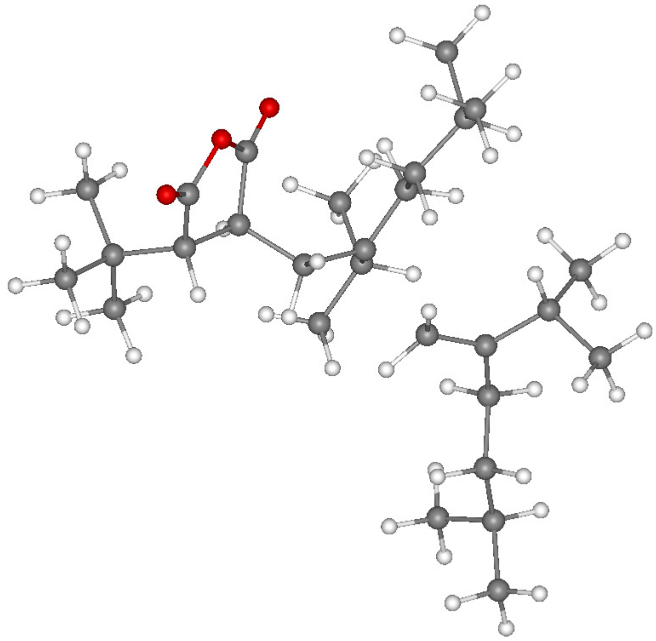

Zero-point vibrational energy	1961447.7 (Joules/Mol)
	468.79724 (Kcal/Mol)
Zero-point correction=	0.747076 (Hartree/Particle)
Thermal correction to Energy=	0.785413
Thermal correction to Enthalpy=	0.786357
Thermal correction to Gibbs Free Energy=	0.674463
Sum of electronic and zero-point Energies=	-1322.551561
Sum of electronic and thermal Energies=	-1322.513223

Sum of electronic and thermal Enthalpies= -1322.512279
Sum of electronic and thermal Free Energies= -1322.624174

cartesian

6	-3.52026677	-0.47182679	-0.17893171	8	5.15743303	-0.07922682	2.34066820
6	-2.59356689	0.56827319	0.40196830	6	4.53103304	-2.50162697	0.34396830
6	-1.36166692	0.20857318	0.89356834	1	3.05703306	-1.66142678	1.66916823
1	-0.85426700	0.87787318	1.57836831	6	5.32153273	-3.07292700	1.52436829
6	-3.06716681	2.00267315	0.48006830	6	3.60093307	-3.59692693	-0.18403171
1	-1.15086699	-0.84382683	1.05446827	6	5.50093269	-2.07112694	-0.75853169
6	-4.14056730	-1.39372683	0.88106835	1	2.68153310	-1.20672679	-1.14123178
1	-2.98006678	-1.08052683	-0.90983170	1	0.98033309	-1.28132677	0.63646829
1	-4.33026695	0.00877319	-0.73703170	1	1.50983298	0.25087318	1.27616823
6	-5.17256689	-2.38822699	0.34116828	1	5.90713310	-3.93532681	1.19606829
1	-4.61406708	-0.77662683	1.65136826	1	6.00633287	-2.34282684	1.95466828
1	-3.34356689	-1.94632685	1.39376831	1	4.65433311	-3.40952682	2.32246828
1	-2.23326683	2.59447312	0.87476832	1	6.07673311	-2.93082690	-1.10863173
6	-4.24006701	2.15547299	1.45576823	1	4.98483276	-1.65582681	-1.62843180
1	-4.53326702	3.20507312	1.54706824	1	6.21813297	-1.32582676	-0.40503171
1	-3.98106694	1.78977323	2.45156837	1	4.18353319	-4.47952652	-0.45833173
1	-5.11856699	1.60187316	1.11256826	1	2.87693310	-3.90392685	0.57546830
6	-5.85186720	-3.12242699	1.49366832	1	3.04703307	-3.29052687	-1.07393169
1	-5.94276714	-1.81422675	-0.19213171	1	-0.48476693	2.28427315	-1.01743174
1	-6.61916685	-3.81202698	1.13226831	1	0.17673308	-0.54772681	-3.88413167
1	-6.33166695	-2.42652702	2.18656826	6	1.24343312	4.96317339	1.59726822
1	-5.12616730	-3.70932698	2.06556821	1	-0.18876693	4.47137308	0.09006830
6	0.48783308	1.90097320	-0.68543166	1	1.13323307	6.04527330	1.48986828
6	0.36423308	0.40247321	-0.52083170	1	2.28603315	4.76447344	1.86406827
6	-0.16906694	-0.31022680	-1.75643170	1	0.62133306	4.64887333	2.43976831
6	0.60913306	0.00497318	-3.04543185	6	-0.29176691	-1.82532680	-1.60163176
6	1.37633312	-0.30442679	0.34156829	1	-0.83446693	-2.11462688	-0.70013165
6	0.96343303	2.71867299	0.51366830	1	0.68503308	-2.31662703	-1.57083178
1	1.17403305	2.09867311	-1.51573169	1	-0.82826698	-2.24992681	-2.45353174
1	2.00763321	2.48087311	0.74896830	6	1.71333301	4.72777319	-0.85463166
6	0.86133307	4.23507309	0.31196830	1	1.38623309	4.31587315	-1.81103170
1	0.38873306	2.45707297	1.40846825	1	2.76203322	4.44727325	-0.71933168
1	-1.18146694	0.08547318	-1.91033173	1	1.66833305	5.81677341	-0.93723166
1	1.66403306	-0.26782680	-2.97513175	6	-3.44436693	2.59557319	-0.88143170
1	0.56973308	1.06427312	-3.30053163	1	-2.63566685	2.49617314	-1.60823178
6	3.57803321	0.62117320	-0.57293171	1	-3.67526698	3.65967298	-0.78383166
6	2.79043317	-0.61972678	-0.23083171	1	-4.32926702	2.11287308	-1.30413175

6	3.66223311	-1.30232680	0.83176833	6	-4.56566715	-3.38642693	-0.64083171
6	4.50963306	-0.15592682	1.34806824	1	-3.76686692	-3.96412683	-0.16403171
8	4.46403313	0.89107323	0.44946828	1	-4.14286709	-2.89992690	-1.52213168
8	3.52043319	1.31987321	-1.53273177	1	-5.31726694	-4.09752655	-0.99313170

tBuMA--MB2-rad--MB2

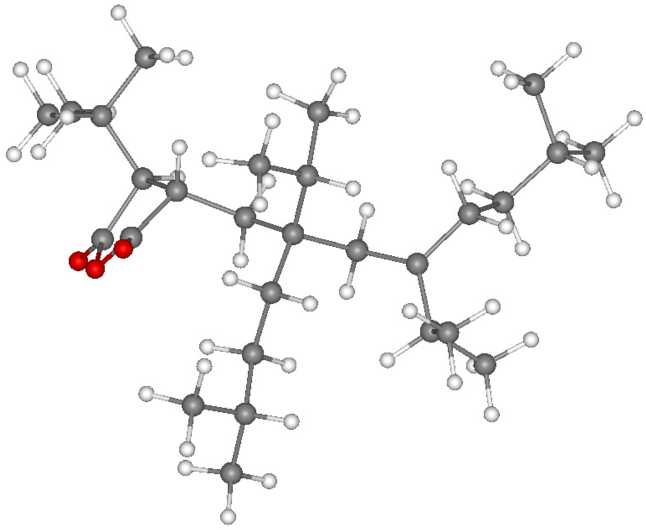

Zero-point vibrational energy	1970024.2 (Joules/Mol)
	470.84708 (Kcal/Mol)
Zero-point correction=	0.750343 (Hartree/Particle)
Thermal correction to Energy=	0.788408
Thermal correction to Enthalpy=	0.789352
Thermal correction to Gibbs Free Energy=	0.678761
Sum of electronic and zero-point Energies=	-1322.583478
Sum of electronic and thermal Energies=	-1322.545413
Sum of electronic and thermal Enthalpies=	-1322.544468
Sum of electronic and thermal Free Energies=	-1322.655059

cartesian

6	-3.47728133	-0.49623901	-0.28834632	8	4.92471838	-0.07833903	2.47265363
6	-2.48108149	0.55526102	0.10835366	6	4.53231859	-2.36873913	0.25205368
6	-1.12378144	0.12196098	0.57255369	1	2.97641850	-1.74073899	1.59925365
1	-0.88538152	0.62846100	1.51645362	6	5.34521818	-2.97023916	1.40195370
6	-2.94028139	1.97826099	0.30445367	6	3.68691850	-3.48333883	-0.37024632
1	-1.16708136	-0.94123900	0.83025366	6	5.48401833	-1.79483902	-0.79964632
6	-3.96698117	-1.37973905	0.87295365	1	2.60581851	-1.09413898	-1.15824628
1	-3.03968143	-1.14433897	-1.05684638	1	0.94391853	-1.35903907	0.65945369
1	-4.35148144	-0.03163902	-0.75724632	1	1.38621855	0.17346098	1.34225368
6	-5.01598167	-2.42663908	0.48365366	1	5.98321819	-3.77353907	1.02505362

1	-4.38478184	-0.73693901	1.65405369	1	5.98301840	-2.23253918	1.88805366
1	-3.11348152	-1.89193904	1.33425367	1	4.69261837	-3.39733887	2.16815352
1	-2.07058144	2.56726074	0.61185366	1	6.12571859	-2.58563900	-1.19454634
6	-3.98108125	2.10296106	1.42745364	1	4.95251846	-1.35993898	-1.65024638
1	-4.23828173	3.15176105	1.60115373	1	6.13971853	-1.02573907	-0.38314632
1	-3.61058164	1.68956101	2.36855364	1	4.33231831	-4.30023909	-0.70184630
1	-4.90578175	1.57866096	1.17145371	1	2.97901869	-3.89763880	0.35295367
6	-5.58088160	-3.09773922	1.73245370	1	3.12161851	-3.14903879	-1.24264634
1	-5.84118176	-1.90273905	-0.01844634	1	-0.55508149	2.23426104	-1.08194637
1	-6.35578156	-3.82553911	1.47825372	1	0.34431854	-0.56493902	-3.81604648
1	-6.02178144	-2.36803913	2.41645360	6	0.99241853	4.94526100	1.60985363
1	-4.79688168	-3.63083887	2.27955365	1	-0.34488147	4.42986107	0.02485366
6	0.35341853	1.82136095	-0.62894630	1	0.90781856	6.02496099	1.46265364
6	0.10891855	0.31696099	-0.38624632	1	2.01191854	4.73926115	1.94995368
6	-0.22458148	-0.38173902	-1.74214637	1	0.31151855	4.66676092	2.41895366
6	0.61771852	0.04296098	-2.94924641	6	-0.25478148	-1.90883899	-1.65234637
6	1.27111852	-0.35093904	0.38675368	1	0.74711853	-2.33843899	-1.56464636
6	0.73941851	2.67136097	0.58125365	1	-0.69738144	-2.33013916	-2.55824637
1	1.13181853	1.93546093	-1.38094628	1	-0.84348148	-2.27643919	-0.80964631
1	1.75601864	2.42496109	0.91135365	6	1.61951852	4.62166119	-0.79684633
6	0.68301857	4.18236113	0.32525367	1	1.35311854	4.17646122	-1.75704634
1	0.09121853	2.45056105	1.43625367	1	2.65301871	4.33596087	-0.58114630
1	-1.24738145	-0.05753903	-1.96984637	1	1.59341860	5.70736122	-0.92234635
1	1.69171858	-0.06943902	-2.79624653	6	-3.48868132	2.61156082	-0.97964633
1	0.44641852	1.08356094	-3.22574639	1	-3.74968123	3.66016102	-0.81244636
6	3.44761848	0.72276098	-0.46764630	1	-4.39358187	2.10396099	-1.32324636
6	2.68121862	-0.55653900	-0.21524633	1	-2.76008129	2.57326078	-1.79234636
6	3.57471871	-1.27363908	0.81265366	6	-4.46838188	-3.47493887	-0.48084635
6	4.33021832	-0.12313902	1.44545364	1	-3.61548162	-4.00153875	-0.03994634
8	4.26871824	0.97586095	0.61185366	1	-4.13758183	-3.04043913	-1.42604637
8	3.43621850	1.45116103	-1.40664637	1	-5.22788143	-4.22433901	-0.71794635

S14. MMI – MMI reaction profile

TS__tBuMMI-rad_MMI__re

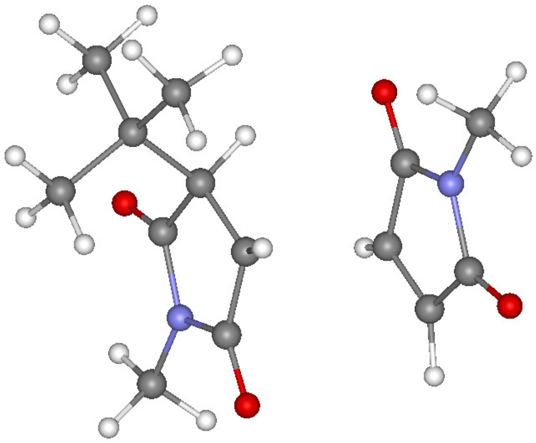

Zero-point vibrational energy	837083.7 (Joules/Mol)
	200.06780 (Kcal/Mol)
Zero-point correction=	0.318828 (Hartree/Particle)
Thermal correction to Energy=	0.339902
Thermal correction to Enthalpy=	0.340846
Thermal correction to Gibbs Free Energy=	0.266458
Sum of electronic and zero-point Energies=	-954.991874
Sum of electronic and thermal Energies=	-954.970801
Sum of electronic and thermal Enthalpies=	-954.969856
Sum of electronic and thermal Free Energies=	-955.044244

cartesian

6	-3.67514348	1.12213588	-0.13850513	1	-0.68424356	0.25873590	-1.13680506
6	-2.59154344	1.97393596	0.40289488	1	-0.96174359	1.49733591	1.78809488
6	-1.71974361	1.18463588	1.08449495	1	0.79905641	-3.22746420	-1.35870504
6	-2.28864360	-0.20366409	1.12149489	1	-0.27844355	-2.59266400	-0.11160512
7	-3.41524339	-0.17556411	0.32409489	1	-0.18664354	-1.81456399	-1.69940507
8	-4.61534357	1.43603587	-0.82120514	1	2.77135658	-1.79406404	-2.15740514
8	-1.86384356	-1.16826403	1.71009493	1	1.81075633	-0.32186410	-2.27070522
1	-2.54084349	3.03523588	0.21699488	1	3.25555658	-0.36756411	-1.24720502
6	0.54225647	1.98113596	-0.51140511	1	2.83925653	-3.12276411	-0.00370511
6	-0.00264353	0.62763590	-0.38510513	1	3.22325659	-1.69386411	0.96919489
6	0.94765639	-0.22766414	0.38449487	1	1.81185639	-2.67656398	1.36119497
6	1.99765646	0.78293586	0.84729487	6	-4.26364374	-1.31366408	0.06869488
7	1.69465637	2.00903583	0.27329490	1	-4.57014370	-1.77546406	1.00709486
8	0.11045647	2.92853594	-1.12710512	1	-3.74584341	-2.05836415	-0.53700513
8	2.93625665	0.59393585	1.57869494	1	-5.13854361	-0.95496404	-0.46940511

6	1.54755640	-1.43786407	-0.40770513	6	2.49435663	3.19693589	0.44949487
1	0.46685645	-0.65166414	1.27479494	1	3.30695653	2.95133591	1.12919497
6	2.40995646	-2.27596402	0.53839487	1	1.88895643	4.00223589	0.86599487
6	0.39895645	-2.30966401	-0.92080516	1	2.89745665	3.52753592	-0.50810510
6	2.39115644	-0.94516408	-1.58410501				

TS__tBuMMI-rad_MMI__si

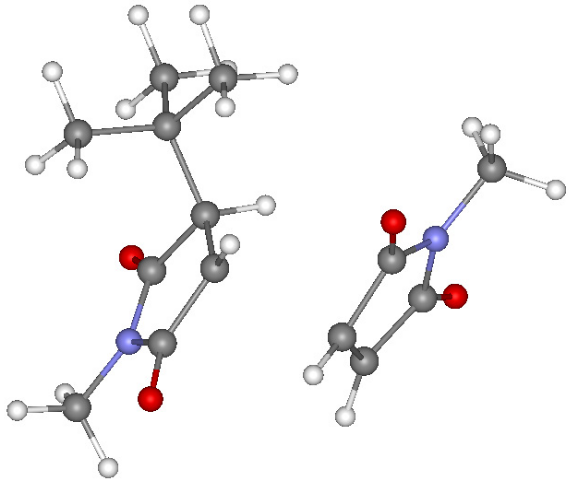

Zero-point vibrational energy	837372.7 (Joules/Mol)
	200.13687 (Kcal/Mol)
Zero-point correction=	0.318938 (Hartree/Particle)
Thermal correction to Energy=	0.340040
Thermal correction to Enthalpy=	0.340984
Thermal correction to Gibbs Free Energy=	0.265798
Sum of electronic and zero-point Energies=	-954.991238
Sum of electronic and thermal Energies=	-954.970136
Sum of electronic and thermal Enthalpies=	-954.969192
Sum of electronic and thermal Free Energies=	-955.044378

cartesian

6	-2.73599243	-1.30046666	-1.09790254	1	-0.02889237	-0.19746672	1.96319747
6	-1.83069229	-2.23776674	-0.39550257	1	-1.23569238	-2.34666657	1.71119750
6	-1.63459229	-1.79166675	0.87409747	1	0.37740761	3.45413327	1.02509749
6	-2.51139235	-0.58696669	1.07519746	1	-0.91699237	2.42163324	0.42239743
7	-3.09269238	-0.32986671	-0.15520257	1	-0.05439237	1.99173331	1.90039742
8	-3.13269234	-1.33336675	-2.23440266	1	2.78110766	2.64953327	0.98769748
8	-2.67749238	0.04953328	2.08449745	1	2.37520766	1.11003327	1.73979747
1	-1.43529236	-3.12286663	-0.87110251	1	3.21410751	1.14693332	0.18109743
6	1.22740769	-1.75986671	1.03569746	1	1.59300768	3.41393328	-1.11100256
6	0.33420759	-0.59946668	1.02839744	1	1.95520771	1.89333320	-1.94290257

6	0.57760763	0.20983329	-0.20160256	1	0.28980762	2.45153332	-1.81070256
6	1.56190765	-0.66486669	-0.98250252	6	2.82490754	-2.77916670	-0.58180255
7	1.89390767	-1.74996674	-0.18620257	1	3.11350751	-2.58686686	-1.61250257
8	1.36740768	-2.60846663	1.88739741	1	2.35650754	-3.75966668	-0.49830258
8	1.99610770	-0.48066670	-2.09060264	1	3.70720744	-2.76176667	0.05879744
6	1.09500766	1.66403317	0.05349744	6	-4.01889229	0.74473321	-0.41550258
1	-0.32649237	0.30283329	-0.81640255	1	-4.12129211	1.32103324	0.50149745
6	1.24620771	2.39173317	-1.28400254	1	-4.99129200	0.34833327	-0.70890254
6	0.06560762	2.41423321	0.90129745	1	-3.64869237	1.38633323	-1.21540248
6	2.44010758	1.63243318	0.78119749				

tBuMMI--MMI-rad__re

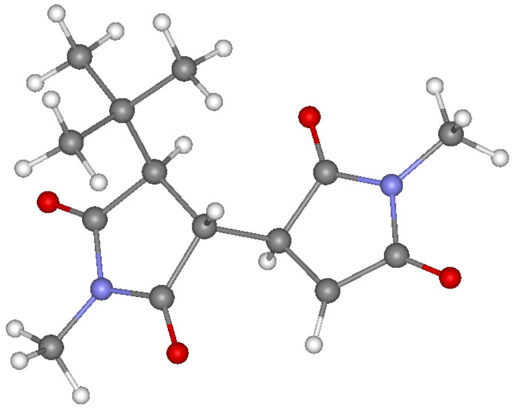

Zero-point vibrational energy	847176.9 (Joules/Mol)
	202.48015 (Kcal/Mol)
Zero-point correction=	0.322673 (Hartree/Particle)
Thermal correction to Energy=	0.343235
Thermal correction to Enthalpy=	0.344180
Thermal correction to Gibbs Free Energy=	0.271790
Sum of electronic and zero-point Energies=	-955.035074
Sum of electronic and thermal Energies=	-955.014511
Sum of electronic and thermal Enthalpies=	-955.013567
Sum of electronic and thermal Free Energies=	-955.085956

cartesian

6	3.61284876	-1.22696161	-0.15635638	1	0.37474874	-0.41496158	-1.18685639
6	2.33144879	-1.88456154	-0.02385639	1	1.05814874	-1.37276161	1.62494361
6	1.36154866	-0.97906154	0.64424366	1	-0.57715124	3.06093836	-1.60445631
6	2.19884872	0.27793843	0.91164362	1	0.41974872	2.56673861	-0.23125638
7	3.45944858	0.05193844	0.41544360	1	0.39744875	1.60593843	-1.71455634
8	4.64724874	-1.62686157	-0.63945639	1	-2.53865123	1.64593840	-2.31995654
8	1.83844876	1.28433847	1.47344363	1	-1.66975117	0.12103844	-2.23405647

1	2.11834884	-2.87106180	-0.40345639	1	-3.14775133	0.35723841	-1.28875637
6	-0.69465125	-2.01916170	-0.29195637	1	-2.64285135	3.19373846	-0.32465637
6	0.07634875	-0.71146154	-0.18025638	1	-3.14355135	1.89943850	0.77284366
6	-0.90995127	0.28113842	0.46124363	1	-1.70295119	2.85093832	1.12984359
6	-2.05205131	-0.60296154	0.93784362	6	4.54144907	1.00283849	0.49604362
7	-1.87025142	-1.87766159	0.40754360	1	4.18514872	1.86833847	1.04944360
8	-0.34465128	-3.03126144	-0.84735638	1	4.85564899	1.30513847	-0.50345635
8	-2.98425126	-0.30386156	1.63804364	1	5.39444876	0.55583841	1.00634360
6	-1.41635132	1.41913843	-0.48225635	6	-2.81505132	-2.95626163	0.58564365
1	-0.46455127	0.76273847	1.33344364	1	-3.55455136	-2.62956142	1.31264365
6	-2.28175139	2.39153862	0.32444364	1	-2.29915142	-3.84666157	0.94264364
6	-0.22005126	2.19933844	-1.03495634	1	-3.30585122	-3.19276142	-0.35905635
6	-2.23555136	0.84563839	-1.64075637				

tBuMMI--MMI-rad__si

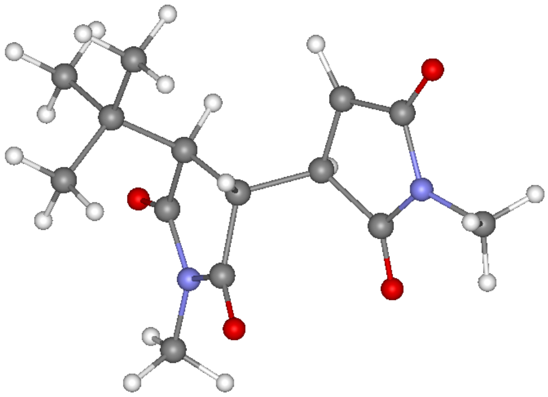

Zero-point vibrational energy	847624.6 (Joules/Mol)
	202.58713 (Kcal/Mol)
Zero-point correction=	0.322843 (Hartree/Particle)
Thermal correction to Energy=	0.343337
Thermal correction to Enthalpy=	0.344281
Thermal correction to Gibbs Free Energy=	0.272259
Sum of electronic and zero-point Energies=	-955.026250
Sum of electronic and thermal Energies=	-955.005757
Sum of electronic and thermal Enthalpies=	-955.004813
Sum of electronic and thermal Free Energies=	-955.076834

cartesian

6	-3.44789743	-1.06807423	0.39116147	1	-0.47389749	-0.37707430	-1.04253852
6	-2.08179736	-1.15247440	0.87196147	1	-1.09489751	0.52912569	1.79516149
6	-1.43649745	0.18252571	0.81176150	1	1.71710253	-3.30057430	-1.11143851
6	-2.59839749	1.09472573	0.40156150	1	0.72260249	-3.05907440	0.31996149

7	-3.67949748	0.28592572	0.10926149	1	0.24240252	-2.35397434	-1.22973847
8	-4.27089739	-1.94487429	0.25446150	1	3.01290250	-1.40247440	-2.11333847
8	-2.59379745	2.29592562	0.38176149	1	1.61590254	-0.33747429	-2.17913842
1	-1.62409747	-2.08367443	1.17076147	1	3.08360243	0.17172571	-1.33413851
6	0.18300250	1.58952570	-0.58693850	1	3.72220230	-2.49617434	0.04296149
6	-0.21189748	0.18582571	-0.14673850	1	3.75960255	-0.94127434	0.88576150
6	1.04110253	-0.34237432	0.57566148	1	2.79060245	-2.26417446	1.52386153
6	1.78690243	0.93832570	0.94516146	6	1.80440259	3.31202555	0.17276150
7	1.24900246	1.97872567	0.20576149	1	2.57980251	3.36632562	0.93316150
8	-0.30199748	2.24862576	-1.46403849	1	1.02270246	4.04222584	0.37776148
8	2.70510244	1.05642569	1.71606147	1	2.23060250	3.52122569	-0.80893850
6	1.92520261	-1.32947421	-0.24473852	6	-4.96169758	0.78592569	-0.32273853
1	0.78190243	-0.84787434	1.51066148	1	-4.88169765	1.86632562	-0.41673851
6	3.12000251	-1.77587438	0.60276151	1	-5.73299742	0.53102571	0.40476149
6	1.09670246	-2.57167435	-0.58473849	1	-5.23179770	0.34722570	-1.28333855
6	2.43040252	-0.68107432	-1.53563857				

TS__tBuMMI-rad_to_MMI_transfer

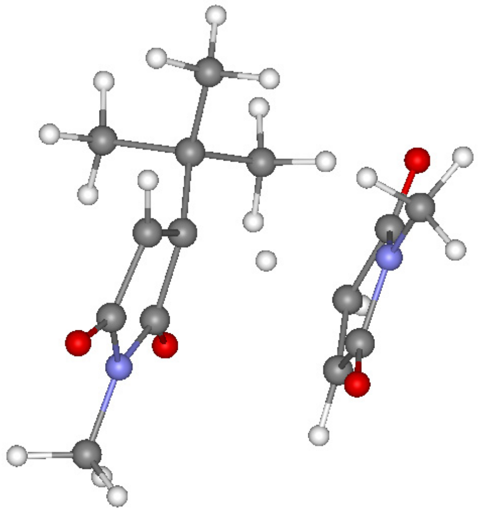

Zero-point vibrational energy	824002.9 (Joules/Mol)
	196.94142 (Kcal/Mol)
Zero-point correction=	0.313846 (Hartree/Particle)
Thermal correction to Energy=	0.335094
Thermal correction to Enthalpy=	0.336038
Thermal correction to Gibbs Free Energy=	0.261753
Sum of electronic and zero-point Energies=	-954.965291
Sum of electronic and thermal Energies=	-954.944043
Sum of electronic and thermal Enthalpies=	-954.943099
Sum of electronic and thermal Free Energies=	-955.017384

cartesian

6	-2.91829729	1.28390527	0.41804612	1	0.09960252	0.26010522	-2.26395392
6	-1.80539751	1.23620522	1.37424624	1	-0.78339744	-0.47989476	2.27384615
6	-1.29529750	-0.05759478	1.41644621	1	1.20810246	-3.31319475	-1.34125376
6	-2.24989748	-0.91419482	0.61654615	1	-0.26269749	-2.36389470	-1.17645383
7	-3.11239743	-0.04019478	-0.01125387	1	0.98940253	-1.88429475	-2.34225392
8	-3.58989739	2.22190523	0.06824613	1	3.43650270	-2.36509466	-0.67795390
8	-2.25419736	-2.11529469	0.50894612	1	3.16420269	-0.84359479	-1.53475380
1	-1.48709750	2.10010529	1.93754613	1	3.55020261	-0.83589482	0.19584614
6	0.65570247	2.09600520	-1.12825382	1	1.87660253	-3.14339471	1.06464624
6	0.51390254	0.65820527	-1.35045385	1	1.75040245	-1.59829473	1.90914619
6	0.94410253	-0.05049476	-0.21995386	1	0.29370251	-2.41569471	1.29174614
6	1.56440246	1.00870526	0.69064611	6	1.64870250	3.50390530	0.69304615
7	1.27550244	2.23170519	0.12424614	1	2.09980249	3.31450534	1.66444623
8	0.35670254	3.02000523	-1.84485376	1	0.77150249	4.14100552	0.80454612
8	2.15070271	0.84010524	1.73154616	1	2.36640263	4.01290512	0.04874614
6	1.50850248	-1.47699475	-0.25545388	6	-4.15609741	-0.43929476	-0.92375386
1	-0.20879748	-0.11679476	0.53504616	1	-4.36349773	-1.49549472	-0.76525390
6	1.34260249	-2.19049478	1.08984613	1	-3.85169744	-0.28399479	-1.96035373
6	0.81340253	-2.29469466	-1.34495378	1	-5.04739761	0.15450522	-0.72885388
6	3.00540257	-1.36499476	-0.58735389				

S15. MMI – ET reaction profile

TS__tBuMMI-rad_ET

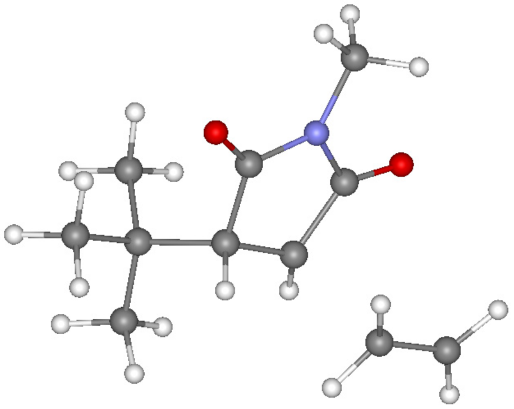

Zero-point vibrational energy	718973.4 (Joules/Mol)
	171.83878 (Kcal/Mol)
Zero-point correction=	0.273843 (Hartree/Particle)
Thermal correction to Energy=	0.290020
Thermal correction to Enthalpy=	0.290964
Thermal correction to Gibbs Free Energy=	0.229379
Sum of electronic and zero-point Energies=	-634.870858
Sum of electronic and thermal Energies=	-634.854680
Sum of electronic and thermal Enthalpies=	-634.853736
Sum of electronic and thermal Free Energies=	-634.915321

cartesian

6	-3.25699997	-2.06290007	0.53277498	1	-2.12349987	-0.78610009	1.79367495
6	-2.12310004	-1.66940022	1.16347504	1	3.02969980	-1.90830016	-0.84452504
6	-1.66020000	0.49149990	-0.71262503	1	1.88510001	-2.36690021	0.41427502
6	-0.79500002	-0.62340009	-0.34462500	1	1.33780003	-2.15020013	-1.25442505
6	0.36019999	-0.10950011	0.46017501	1	2.82390022	0.36909992	-1.97502506
6	-0.02990001	1.35139990	0.70327497	1	1.08379996	0.22039990	-2.21212506
7	-1.17919993	1.60920000	-0.01622500	1	1.74669993	1.59669995	-1.31842506
8	-2.63030005	0.51669991	-1.43512499	1	3.83389997	0.09339990	0.33087501
8	0.53979999	2.16089988	1.39447498	1	2.70469999	1.25199986	1.05277503
6	1.76809990	-0.27500010	-0.19592500	1	2.82929993	-0.38130009	1.70247495
1	0.41859996	-0.59160012	1.44447494	1	-4.11380005	-1.40720010	0.44367501
6	2.84369993	0.20709990	0.78037500	1	-3.31549978	-3.01510024	0.01797500
6	2.00820017	-1.75860000	-0.48642498	6	-1.82480001	2.89759994	-0.06412500
6	1.85350001	0.52379990	-1.49712503	1	-1.28929996	3.56269979	0.60937500
1	-0.73710001	-1.47760010	-1.00132501	1	-2.86619997	2.80879974	0.24597500
1	-1.32140005	-2.37660027	1.34617496	1	-1.80070007	3.29839993	-1.07822502

tBuMMI--ET-rad

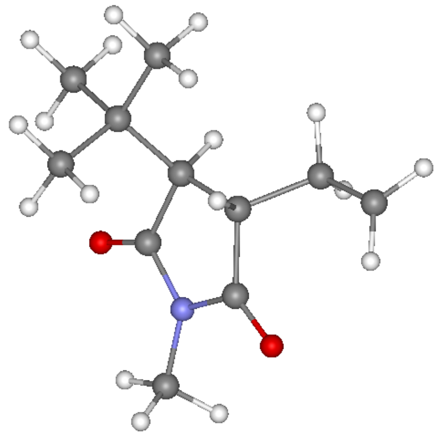

Zero-point vibrational energy	726290.5 (Joules/Mol)
	173.58758 (Kcal/Mol)
Zero-point correction=	0.276629 (Hartree/Particle)
Thermal correction to Energy=	0.292492
Thermal correction to Enthalpy=	0.293436
Thermal correction to Gibbs Free Energy=	0.233440
Sum of electronic and zero-point Energies=	-634.909125
Sum of electronic and thermal Energies=	-634.893263
Sum of electronic and thermal Enthalpies=	-634.892319
Sum of electronic and thermal Free Energies=	-634.952315

cartesian

6	2.41446257	2.69023418	0.26809373	1	1.73906255	1.36923432	1.80899370
6	1.37536263	1.81443429	0.86679375	1	-3.74003720	-0.43986568	0.14879374
6	1.97126245	-0.35826570	-0.29960626	1	-2.60043740	-1.67436576	0.71099377
6	0.88996255	0.67533433	-0.04340626	1	-2.83243752	-0.19496568	1.64199376
6	-0.29593748	-0.11816568	0.53159374	1	-2.58943748	-0.28846568	-2.09640646
6	0.17626253	-1.56666565	0.53449374	1	-0.85473746	-0.02436568	-2.20910645
7	1.47356248	-1.59996569	0.04869374	1	-1.48173738	-1.55926573	-1.59040630
8	3.08256269	-0.18046568	-0.73200625	1	-2.96383739	1.74673426	-0.67310625
8	-0.42523748	-2.54606581	0.89889371	1	-2.00733733	2.02203441	0.77949375
6	-1.66503739	0.08973432	-0.17340626	1	-1.25853753	2.15383434	-0.81680626
1	-0.43373746	0.15153432	1.58629370	1	3.17116261	2.27683425	-0.38360626
6	-2.77113748	-0.60256565	0.62859374	1	2.53436255	3.70633435	0.62269372
6	-1.98043752	1.58743429	-0.22380626	6	2.24796247	-2.81306577	-0.07230626
6	-1.63743758	-0.47786567	-1.59470630	1	1.64496255	-3.62976575	0.31699374
1	0.64036250	1.10283434	-1.01880622	1	3.17396259	-2.72466564	0.49539375
1	0.50426257	2.41173434	1.15559375	1	2.49796271	-2.99946570	-1.11690629

TS__tBuMMI--ET-rad_MMI__re

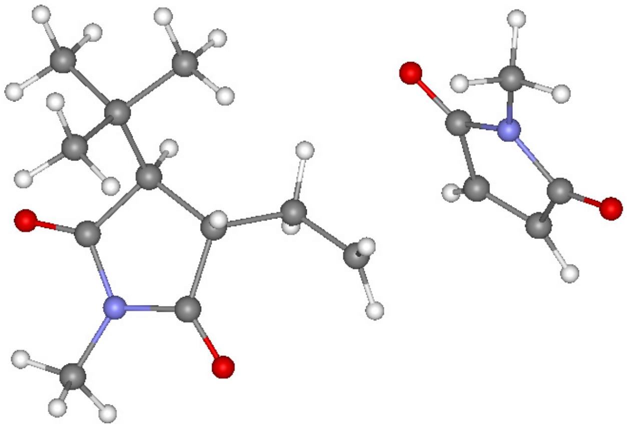

Zero-point vibrational energy	986464.8 (Joules/Mol)
	235.77073 (Kcal/Mol)
Zero-point correction=	0.375725 (Hartree/Particle)
Thermal correction to Energy=	0.399483
Thermal correction to Enthalpy=	0.400427
Thermal correction to Gibbs Free Energy=	0.319024
Sum of electronic and zero-point Energies=	-1033.550591
Sum of electronic and thermal Energies=	-1033.526833
Sum of electronic and thermal Enthalpies=	-1033.525889
Sum of electronic and thermal Free Energies=	-1033.607292

cartesian

6	3.52129102	0.32417789	0.77435559	1	-0.61590898	-0.25482211	-1.10784447
6	3.49539089	-1.05792212	1.35245550	1	0.70949101	0.16367787	0.99525559
6	4.54479122	-1.74182212	0.85205555	1	0.07529104	-1.33782220	1.65975547
6	5.23439121	-0.89132214	-0.14474444	1	-3.27230906	3.46587801	0.32495555
7	4.55299091	0.33197787	-0.14544444	1	-4.11290884	2.00027800	0.85715556
8	2.81039095	1.26487792	1.03795552	1	-2.72010899	2.60037804	1.75935555
8	6.19409084	-1.14832211	-0.82624441	1	-2.87080908	2.52077794	-1.98334455
1	2.86919093	-1.31062210	2.19435549	1	-2.15610909	0.92937791	-2.19814444
1	4.87519121	-2.74432206	1.07815552	1	-3.78190899	1.08927786	-1.51614451
6	1.43409097	-1.57822216	0.03215556	1	-0.98160899	3.37367797	-0.57564443
6	0.35129103	-0.83572209	0.72665554	1	-0.38550898	2.45177794	0.80145556
6	-1.59490895	-1.97832215	-0.40684444	1	-0.12470898	1.86197793	-0.83954442
6	-0.91660905	-0.65192211	-0.13344444	1	1.82459104	-1.17802215	-0.89794445
6	-1.99630904	0.23527789	0.50715554	1	1.54069102	-2.64632201	0.16135556
6	-3.25350904	-0.62682211	0.48455557	6	4.93149090	1.47377789	-0.93904442
7	-2.92540908	-1.86192214	-0.05714444	1	5.84929085	1.21747780	-1.46424448
8	-1.09410906	-2.98432207	-0.84404445	1	5.10129118	2.34137797	-0.30124444
8	-4.35860920	-0.34312212	0.87235558	1	4.15589094	1.71967781	-1.66594446

6	-2.19400907	1.64237785	-0.12304443	6	-3.87050915	-2.94502211	-0.20034444
1	-1.75570893	0.38797787	1.56675553	1	-4.80420923	-2.63302207	0.26125556
6	-3.13620901	2.47037792	0.75605553	1	-3.48700905	-3.84022212	0.28795555
6	-0.84380901	2.36177802	-0.18644443	1	-4.03600883	-3.16812205	-1.25474453
6	-2.78230906	1.52987790	-1.53194451				

TS__tBuMMI--ET-rad_MMI__si

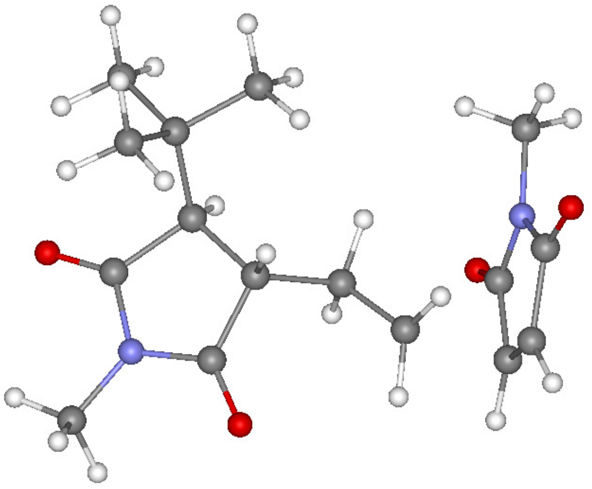

Zero-point vibrational energy 986475.1 (Joules/Mol)

235.77320 (Kcal/Mol)

Zero-point correction= 0.375729 (Hartree/Particle)

Thermal correction to Energy= 0.399564

Thermal correction to Enthalpy= 0.400508

Thermal correction to Gibbs Free Energy= 0.318101

Sum of electronic and zero-point Energies= -1033.549703

Sum of electronic and thermal Energies= -1033.525868

Sum of electronic and thermal Enthalpies= -1033.524924

Sum of electronic and thermal Free Energies= -1033.607331

cartesian

6	-3.91306448	-0.65327334	-1.46663332	6	3.14063549	1.46162677	1.20896673
6	-3.81626463	-1.93367338	-0.72663331	1	0.98483562	-0.32047334	1.60656667
6	-3.78356457	-1.66737342	0.59436667	1	-1.08896446	0.26652664	0.25876665
6	-3.95846462	-0.18947335	0.78076667	1	-0.85126442	-1.21467328	-0.65803331
7	-3.98756456	0.34952664	-0.49663335	1	2.85973549	3.51962662	-0.56503332
8	-3.93816423	-0.46157336	-2.65643334	1	3.39603567	2.09662676	-1.47333336
8	-4.05526447	0.43042666	1.80986667	1	1.76723564	2.73182678	-1.70443344
1	-3.77286434	-2.88767338	-1.23043323	1	3.43433571	2.42332673	1.63586664
1	-3.86126423	-2.35457325	1.42256665	1	2.82653570	0.82472664	2.04066658
6	-1.41506445	-1.51187325	1.37566662	1	4.03223562	1.01892662	0.75936669

6	-0.69506443	-0.75107336	0.32316667	1	1.14763558	3.35152674	1.19336677
6	1.51733553	-1.98277330	0.43266666	1	0.02873558	2.52712679	0.11296666
6	0.82693553	-0.63977337	0.57216668	1	0.43693560	1.81992674	1.68256664
6	1.56043553	0.29642665	-0.40333334	1	-1.71376443	-1.00527334	2.28636670
6	2.69343567	-0.55547333	-0.96503335	1	-1.27976441	-2.58367324	1.43306661
7	2.58143568	-1.83187342	-0.43353334	6	3.46833563	-2.91887331	-0.77933335
8	1.22243559	-3.02497339	0.96266669	1	4.18713570	-2.54087329	-1.50223327
8	3.54993534	-0.23297334	-1.74873328	1	2.90103579	-3.74377322	-1.20973325
6	2.03033543	1.66072655	0.17416665	1	3.98683548	-3.27947330	0.10896666
1	0.90373564	0.51422668	-1.25523329	6	-4.15386438	1.75012660	-0.79143333
1	-3.33636427	2.11022663	-1.41673326	1	-4.15876436	2.28742671	0.15476665
6	2.54893541	2.54912663	-0.96123332	1	-5.09456444	1.92692661	-1.31433344
6	0.84073555	2.36852670	0.82816666				

tBuMMI--ET-rad--MMI__re

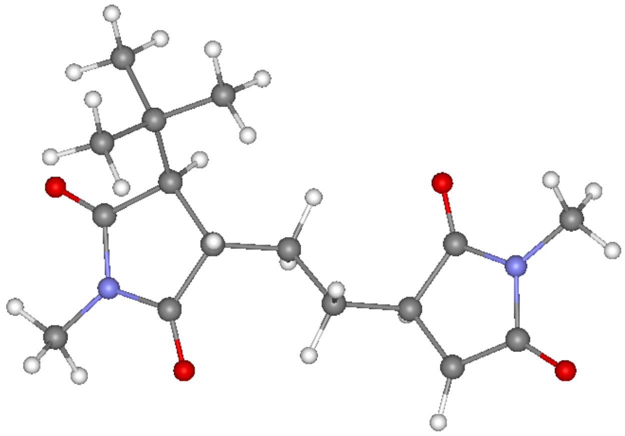

Zero-point vibrational energy	998198.2 (Joules/Mol)
	238.57509 (Kcal/Mol)
Zero-point correction=	0.380194 (Hartree/Particle)
Thermal correction to Energy=	0.403412
Thermal correction to Enthalpy=	0.404356
Thermal correction to Gibbs Free Energy=	0.324695
Sum of electronic and zero-point Energies=	-1033.605264
Sum of electronic and thermal Energies=	-1033.582046
Sum of electronic and thermal Enthalpies=	-1033.581102
Sum of electronic and thermal Free Energies=	-1033.660763

cartesian

6	3.52113771	0.33056444	0.70959556	1	-0.72266221	-0.25113556	-1.22870445
6	2.93013787	-1.07473552	0.59369558	1	0.68503785	0.57546443	0.75399554
6	4.03853798	-1.83963561	-0.02610443	1	0.33293781	-0.94343555	1.54469562

6	5.18783760	-0.98573554	-0.23870443	1	-3.66836214	3.32276440	-0.00220443
7	4.80983734	0.28896445	0.22559558	1	-4.27606201	1.86256456	0.79539555
8	2.98173785	1.32256436	1.13689554	1	-2.88896227	2.72466445	1.46319556
8	6.27103806	-1.24883556	-0.71030444	1	-3.36786222	2.12466455	-2.20240450
1	2.73753786	-1.43493557	1.61529553	1	-2.49136209	0.60366446	-2.29360437
1	4.02373791	-2.88453555	-0.29920441	1	-4.05596256	0.68006444	-1.46910441
6	1.60503781	-1.12523556	-0.18640442	1	-1.47286224	3.35476446	-1.11560440
6	0.44763780	-0.47183555	0.56079555	1	-0.65116221	2.69126439	0.29429558
6	-1.44046223	-1.95923567	-0.23560442	1	-0.47336221	1.91856456	-1.28410447
6	-0.88746214	-0.54713559	-0.18720444	1	1.74423778	-0.63683558	-1.15740442
6	-2.00616217	0.29956442	0.44549555	1	1.36403775	-2.16803551	-0.39850444
6	-3.15096211	-0.68083555	0.66439557	6	5.68023777	1.43796444	0.18509558
7	-2.73396230	-1.93573546	0.24379557	1	6.62683773	1.11736441	-0.24420442
8	-0.88386214	-2.96153545	-0.61390442	1	5.84133816	1.82866454	1.19019556
8	-4.24096203	-0.46433556	1.13109553	1	5.24233818	2.22636461	-0.42780447
6	-2.42586207	1.58016443	-0.32980442	6	-3.55906224	-3.11843538	0.32929558
1	-1.69056225	0.61986446	1.44619560	1	-4.49886227	-2.83183551	0.79509556
6	-3.37576222	2.41566443	0.53359556	1	-3.06046224	-3.88133550	0.92659557
6	-1.18176222	2.42356443	-0.62300444	1	-3.74486232	-3.52253556	-0.66590446
6	-3.12156224	1.21816444	-1.64450443				

tBuMMI--ET-rad--MMI__si

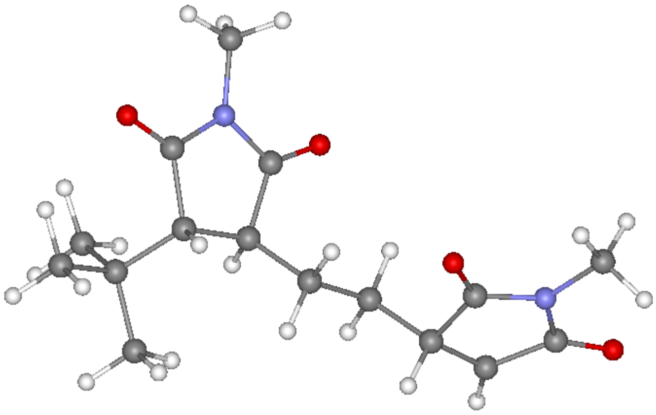

Zero-point vibrational energy	998121.2 (Joules/Mol)
	238.55669 (Kcal/Mol)
Zero-point correction=	0.380164 (Hartree/Particle)
Thermal correction to Energy=	0.403410
Thermal correction to Enthalpy=	0.404354
Thermal correction to Gibbs Free Energy=	0.324312
Sum of electronic and zero-point Energies=	-1033.604486
Sum of electronic and thermal Energies=	-1033.581241
Sum of electronic and thermal Enthalpies=	-1033.580297

Sum of electronic and thermal Free Energies= -1033.660338

cartesian

6	5.26747990	-0.29276225	0.58606446	1	-0.93351990	-0.55296230	1.36416447
6	4.14888000	-1.13586223	0.95066446	1	0.35098007	-1.42146218	-0.65123558
6	3.00957990	-0.91136217	0.02896444	1	0.74358010	0.23153776	-1.06613553
6	3.55807996	0.15033776	-0.92713559	1	-4.78942013	-2.14756227	-0.98943555
7	4.84728003	0.44283777	-0.53513557	1	-4.63641977	-0.42426226	-1.37053549
8	6.36308002	-0.19236225	1.09146452	1	-3.60582018	-1.60436225	-2.18033552
8	2.98517990	0.65663779	-1.86073554	1	-4.42651987	-1.72746217	1.47206450
1	4.17318010	-1.81326222	1.79166448	1	-3.04661989	-0.79756224	2.04046440
1	2.80428004	-1.81386232	-0.56553555	1	-4.34422016	-0.00136225	1.13766444
6	1.70598006	-0.48116222	0.72626442	1	-3.00902009	-3.36276221	0.19766445
6	0.51488006	-0.42616227	-0.22393556	1	-1.77641988	-2.80296230	-0.92933559
6	-0.75321990	1.48023784	0.86026442	1	-1.56591988	-2.56696224	0.81116444
6	-0.79031998	0.02093776	0.44246444	1	1.49988008	-1.19886220	1.52826452
6	-2.02291989	-0.10166225	-0.47173557	1	1.84818006	0.48643777	1.21006453
6	-2.63351989	1.29413784	-0.48113558	6	-2.10491991	3.52653790	0.49676442
7	-1.83981991	2.12003779	0.29966444	1	-3.00011992	3.77593780	-0.06753555
8	0.06048006	2.03033781	1.56106448	1	-1.26171994	4.12023783	0.14436445
8	-3.62181997	1.67103779	-1.05913556	1	-2.25941992	3.73603773	1.55516446
6	-3.03911996	-1.22186220	-0.11313555	6	5.69138002	1.40793777	-1.19493556
1	-1.68871999	-0.28636226	-1.50023556	1	5.14728022	1.79263783	-2.05433559
6	-4.08122015	-1.34966218	-1.22863555	1	6.61938000	0.93623781	-1.51863551
6	-2.29791999	-2.55686212	-0.00013556	1	5.93677998	2.22513771	-0.51573557
6	-3.74711990	-0.91346216	1.20846450				

TS__tBuMMI--ET-rad_ET

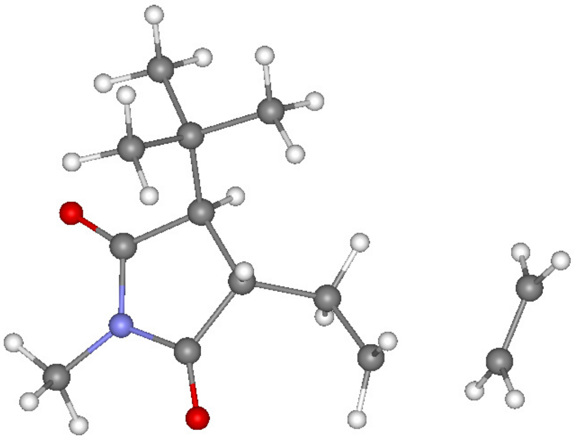

Zero-point vibrational energy 867462.1 (Joules/Mol)

207.32841 (Kcal/Mol)

Zero-point correction= 0.330399 (Hartree/Particle)

Thermal correction to Energy=	0.349331
Thermal correction to Enthalpy=	0.350276
Thermal correction to Gibbs Free Energy=	0.281527
Sum of electronic and zero-point Energies=	-713.427058
Sum of electronic and thermal Energies=	-713.408126
Sum of electronic and thermal Enthalpies=	-713.407182
Sum of electronic and thermal Free Energies=	-713.475930

cartesian

1	-5.04094744	-1.53062105	0.13204738	6	0.37295264	-2.43172097	-0.56235266
6	-4.65464735	-0.78502095	0.81824738	6	2.34485269	-1.18232095	-1.46105266
6	-4.60134745	0.52247894	0.48614737	1	-0.13364737	0.18697898	-1.38385260
1	-5.10454750	0.89317894	-0.39915264	1	-1.72104740	-0.62582099	0.42594737
1	-4.26534748	-1.14862096	1.76274741	1	-1.52144742	0.91947901	1.22944736
1	-4.32774734	1.27097893	1.22124732	1	2.81595254	-3.16682100	0.35424736
1	-2.71334743	2.07687902	-0.66095263	1	3.28415251	-1.63232100	1.10594738
6	-2.58984733	1.00187898	-0.61875266	1	1.87755263	-2.51522088	1.69914734
6	-1.51934743	0.43487898	0.24624738	1	2.69425249	-2.10312104	-1.93415260
6	0.38145262	1.97757900	-0.40985262	1	1.75335264	-0.64282107	-2.20615244
6	-0.09814738	0.54107898	-0.34905264	1	3.22715259	-0.58152103	-1.22835267
6	0.98115265	-0.21132103	0.44924736	1	0.75465262	-3.36822104	-0.97645259
6	2.04785275	0.83937895	0.73344737	1	-0.22374736	-2.68372107	0.31854737
7	1.61565256	2.04747891	0.21084738	1	-0.29604733	-1.99712110	-1.30895269
8	-0.17734738	2.92797899	-0.89695263	1	-2.81954741	0.47557896	-1.54005265
8	3.09195256	0.70157897	1.32114732	6	2.36255264	3.27827907	0.32604736
6	1.53875256	-1.50762105	-0.20105264	1	3.27125263	3.06237912	0.88264740
1	0.57235265	-0.49242100	1.42824733	1	1.77085257	4.02907896	0.84954739
6	2.43675256	-2.24272108	0.79904735	1	2.61265254	3.66197896	-0.66305262

tBuMMI--ET-rad--ET

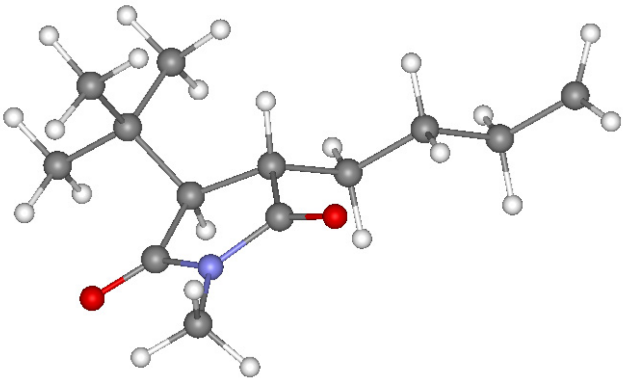

Zero-point vibrational energy	876390.4 (Joules/Mol)
	209.46233 (Kcal/Mol)

Zero-point correction=	0.333799 (Hartree/Particle)
Thermal correction to Energy=	0.352716
Thermal correction to Enthalpy=	0.353660
Thermal correction to Gibbs Free Energy=	0.285308
Sum of electronic and zero-point Energies=	-713.476351
Sum of electronic and thermal Energies=	-713.457434
Sum of electronic and thermal Enthalpies=	-713.456490
Sum of electronic and thermal Free Energies=	-713.524842

cartesian

1	-5.71097374	0.86739218	-0.19334473	6	0.90892631	-2.48210788	-0.32244471
6	-5.28577375	-0.11580783	-0.02994473	6	2.41352630	-0.89500785	-1.53684473
6	-4.00087404	-0.27100787	0.69885528	1	-0.29147366	-0.11450785	-1.17444468
1	-4.00677395	0.35429215	1.60085523	1	-1.37067366	-1.10970783	0.92795527
1	-5.74447393	-0.94950783	-0.54704469	1	-1.49607372	0.52079213	1.54935527
1	-3.88587356	-1.30530787	1.04215527	1	3.54972649	-2.58230782	0.28215528
1	-2.86727357	1.15039217	-0.48144472	1	3.73712635	-0.93150783	0.89835531
6	-2.77427363	0.11649215	-0.14464474	1	2.65662646	-2.06290793	1.71265531
6	-1.46877360	-0.06300783	0.62095529	1	2.90142632	-1.74820781	-2.01394463
6	-0.08437367	1.81519210	-0.36804470	1	1.62702632	-0.55390787	-2.21594477
6	-0.21117365	0.31559217	-0.17034474	1	3.15762639	-0.09960786	-1.45274472
6	1.10862637	-0.11170784	0.49685529	1	1.44432640	-3.34010792	-0.73664474
6	1.93192625	1.16749215	0.57665527	1	0.49482635	-2.79100776	0.64135528
7	1.16912627	2.20489216	0.06525527	1	0.07422633	-2.26750779	-0.99434471
8	-0.90157372	2.57919216	-0.81804472	1	-2.75177360	-0.50060785	-1.05044472
8	3.04642630	1.31159210	1.01425529	6	1.62162638	3.57539201	0.01305527
6	1.86552644	-1.29690790	-0.16524473	1	2.61662650	3.61099219	0.45005527
1	0.90672636	-0.40030786	1.53605533	1	0.94172633	4.21669245	0.57365531
6	3.02392650	-1.73730779	0.73485529	1	1.65452623	3.92349219	-1.01944470

S16. MMI – IB reaction profile

TS__tBuMMI-rad_IB

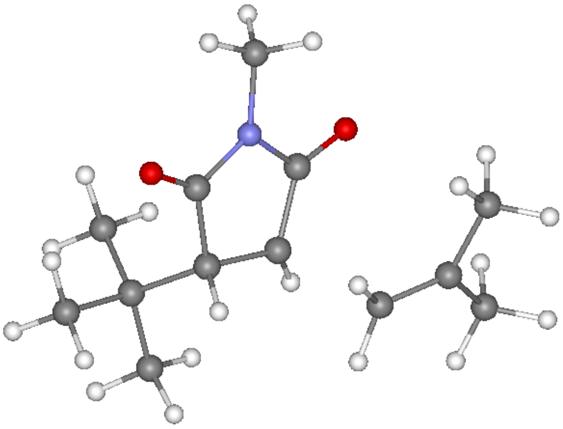

Zero-point vibrational energy	868029.1 (Joules/Mol)
	207.46394 (Kcal/Mol)
Zero-point correction=	0.330615 (Hartree/Particle)
Thermal correction to Energy=	0.349743
Thermal correction to Enthalpy=	0.350687
Thermal correction to Gibbs Free Energy=	0.281763
Sum of electronic and zero-point Energies=	-713.453638
Sum of electronic and thermal Energies=	-713.434510
Sum of electronic and thermal Enthalpies=	-713.433566
Sum of electronic and thermal Free Energies=	-713.502489

cartesian

6	-3.03821063	-0.64696312	0.54961050	1	1.38448942	-2.44306302	-1.12548947
6	-1.82271051	-0.83296311	1.13831055	1	3.58238935	-0.54456311	-1.88398945
6	-0.65321052	1.00703692	-0.81238949	1	1.88828945	-0.14886314	-2.17188931
6	-0.20091054	-0.29426312	-0.35318947	1	2.93438935	0.98773688	-1.30878949
6	1.03578949	-0.12426314	0.47641051	1	4.40618944	-1.00196314	0.47481051
6	1.11128950	1.39603686	0.64581054	1	3.67108941	0.48183686	1.10541058
7	0.12878945	1.95953691	-0.14248948	1	3.26878953	-1.07616317	1.82301056
8	-1.53981054	1.29433692	-1.58908951	6	-3.62551045	-1.67026317	-0.36558947
8	1.87978947	2.02443695	1.33401048	1	-3.63861060	-1.29186308	-1.39408946
6	2.34118938	-0.74276310	-0.11838948	1	-4.66451073	-1.89136314	-0.10118948
1	0.91928947	-0.55396312	1.47971046	1	-3.06501055	-2.60626316	-0.35498947
6	3.48808956	-0.56746310	0.87971050	6	-3.78761053	0.63753688	0.68281054
6	2.12108946	-2.24056315	-0.34498948	1	-4.81381035	0.45653689	1.02061057
6	2.70088959	-0.07126313	-1.44438946	1	-3.85371041	1.14533687	-0.28438950
1	-0.39321056	-1.15716314	-0.97238946	1	-3.31311059	1.31883693	1.39031053

1	-1.37971056	-1.82226324	1.17811048	6	-0.07691056	3.37963676	-0.27758947
1	-1.46441054	-0.12546313	1.87911057	1	0.70268947	3.88383698	0.28911051
1	3.05648947	-2.71306324	-0.65518945	1	-1.05621052	3.66443682	0.10981052
1	1.79118943	-2.73866320	0.57131052	1	-0.02501055	3.66833687	-1.32748950

TS__tBuMMI-rad_to_IB_transfer

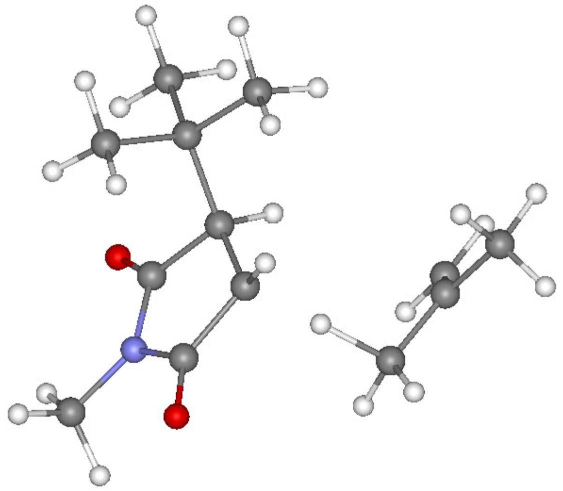

Zero-point vibrational energy	856529.8 (Joules/Mol)
	204.71553 (Kcal/Mol)
Zero-point correction=	0.326235 (Hartree/Particle)
Thermal correction to Energy=	0.345232
Thermal correction to Enthalpy=	0.346177
Thermal correction to Gibbs Free Energy=	0.277240
Sum of electronic and zero-point Energies=	-713.441600
Sum of electronic and thermal Energies=	-713.422602
Sum of electronic and thermal Enthalpies=	-713.421658
Sum of electronic and thermal Free Energies=	-713.490595

cartesian

6	-2.14796591	-1.84932375	0.09927629	1	-0.12796591	-0.31072369	1.89717638
6	-3.01746607	-0.81442368	-0.44392371	1	-3.68666601	0.23357630	-2.16862369
6	-3.83396578	-0.02832370	0.54147625	1	-2.46926594	-1.12202370	-2.47402358
6	-3.05636597	-0.55002367	-1.76372361	1	0.27433410	3.37567639	1.31337631
1	-1.04196596	-1.27492368	0.53077626	1	-0.98846585	2.40707636	0.55387628
1	-3.19486594	0.44477630	1.29337633	1	-0.20036592	1.84567642	2.03557634
1	-4.52426577	-0.68152368	1.08317637	1	2.66813397	2.51657629	1.37137628
6	1.10463405	-1.80232370	0.90297627	1	2.19623399	0.91047633	1.92177629
6	0.15013409	-0.68092370	0.91677630	1	3.13523412	1.10157633	0.43407631
6	0.51093411	0.26197630	-0.20492372	1	1.64243400	3.51477623	-0.71732372
6	1.53883410	-0.54462373	-1.00002372	1	2.01753402	2.07577634	-1.67962372

7	1.83743405	-1.68642366	-0.27992371	1	0.36053407	2.66417623	-1.58412361
8	1.24233401	-2.70282364	1.69727635	1	-1.75056601	-2.56382370	-0.61912376
8	2.02743411	-0.26882368	-2.06872368	1	-4.41886616	0.75277627	0.05517629
6	1.03593409	1.66537631	0.22857629	1	-2.48026586	-2.32792377	1.01917636
1	-0.34616590	0.43407631	-0.86882371	6	2.80723405	-2.66782379	-0.69992375
6	1.28313410	2.52537632	-1.01322365	1	3.15243411	-2.38882375	-1.69292367
6	-0.03286590	2.35247636	1.08267629	1	2.35283399	-3.65822363	-0.72362375
6	2.32993412	1.53387630	1.03337634	1	3.65063405	-2.69222379	-0.00872371

tBuMMI--IB-rad

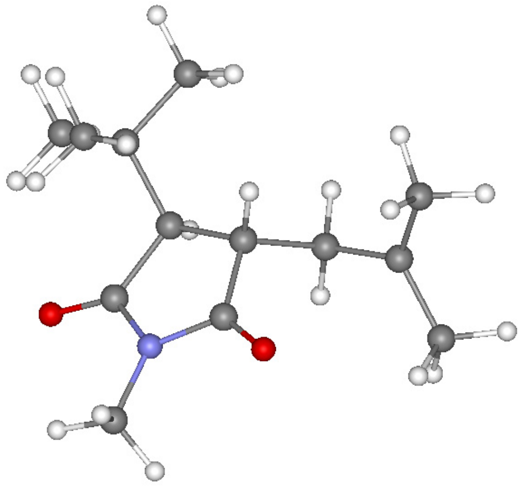

Zero-point vibrational energy	876375.5 (Joules/Mol)
	209.45877 (Kcal/Mol)
Zero-point correction=	0.333794 (Hartree/Particle)
Thermal correction to Energy=	0.352708
Thermal correction to Enthalpy=	0.353652
Thermal correction to Gibbs Free Energy=	0.285688
Sum of electronic and zero-point Energies=	-713.488487
Sum of electronic and thermal Energies=	-713.469573
Sum of electronic and thermal Enthalpies=	-713.468629
Sum of electronic and thermal Free Energies=	-713.536593

cartesian

6	-2.88273430	-0.58573413	0.43280786	1	3.13936567	-1.31543422	1.74700785
6	-1.49003410	-0.52223414	0.96250790	1	3.02376604	-1.21953416	-1.99739206
6	-0.68213415	1.43726587	-0.43279213	1	1.44426584	-0.45983416	-2.14669204
6	-0.41533414	0.00626588	-0.02019213	1	2.82456589	0.46986586	-1.54529214
6	1.00386584	0.01256588	0.57310790	1	2.19546604	-3.06773424	-0.49559215
6	1.42866588	1.47526598	0.52310789	1	1.20226586	-2.69973421	0.91250789
7	0.41136587	2.20326591	-0.07329214	1	0.56096584	-2.45893431	-0.71709210

8	-1.66213417	1.87986588	-0.97879213	6	-3.15623426	-1.42033410	-0.76959211
8	2.45476580	1.96616578	0.92400789	1	-3.02883434	-0.84573418	-1.69959211
6	2.03136587	-0.94853407	-0.09019212	1	-4.18523407	-1.79213428	-0.77599210
1	0.95806587	-0.25463414	1.63580787	1	-2.49043417	-2.28703427	-0.83269209
6	3.32616568	-0.96463412	0.72780788	6	-3.91753435	0.38626587	0.87870789
6	1.45636582	-2.36773419	-0.09789212	1	-4.91623449	-0.06313413	0.87970787
6	2.34126568	-0.51003414	-1.52319217	1	-3.96283436	1.25796580	0.21090788
1	-0.46593416	-0.59263414	-0.93319213	1	-3.72043419	0.76356590	1.88560784
1	-1.15823412	-1.52883410	1.25470781	6	0.47616586	3.62816572	-0.29829213
1	-1.45553410	0.08496587	1.87360787	1	1.42486584	3.98246574	0.09780787
1	4.04966593	-1.64573407	0.27150786	1	-0.35063413	4.12696600	0.20710787
1	3.77536583	0.02466586	0.79410791	1	0.41186586	3.84476590	-1.36469221

TS__tBuMMI--IB-rad_MMI__re

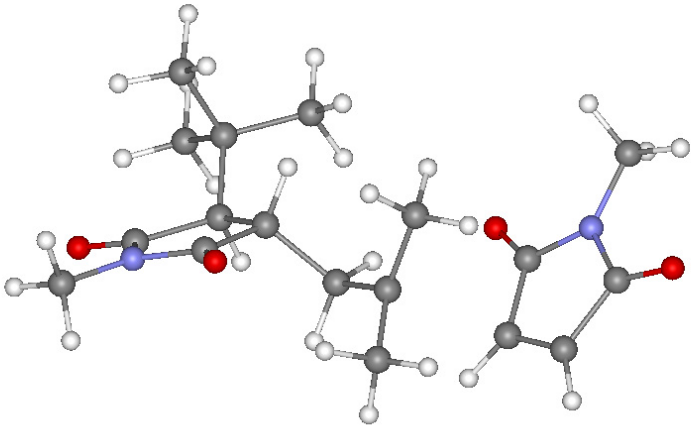

Zero-point vibrational energy	1138197.8 (Joules/Mol)
	272.03581 (Kcal/Mol)
Zero-point correction=	0.433517 (Hartree/Particle)
Thermal correction to Energy=	0.459673
Thermal correction to Enthalpy=	0.460617
Thermal correction to Gibbs Free Energy=	0.375914
Sum of electronic and zero-point Energies=	-1112.130952
Sum of electronic and thermal Energies=	-1112.104796
Sum of electronic and thermal Enthalpies=	-1112.103852
Sum of electronic and thermal Free Energies=	-1112.188555

cartesian

6	3.15619040	0.73377651	0.80623138	6	-3.02780962	1.72107649	-1.46266854
6	3.12219024	-0.63022351	1.41473138	1	-0.79030973	-0.00412349	-1.08046854
6	4.25879049	-1.27792346	1.04193139	1	0.55419028	0.35227650	0.97273135
6	4.99879026	-0.43122351	0.09043138	1	-0.12830974	-1.09912348	1.69323146

7	4.26929045	0.76457649	-0.00846861	1	-3.42620969	3.71107650	0.35893136
8	2.38079023	1.65167654	0.96253139	1	-4.23470974	2.26167655	0.97743136
8	6.03029013	-0.65592349	-0.49466860	1	-2.79880977	2.88877654	1.78773141
1	2.48169041	-0.85582352	2.25373149	1	-3.14030957	2.69707656	-1.94056869
1	4.62389040	-2.24412346	1.35523140	1	-2.43690968	1.09887648	-2.14116859
6	1.41929030	-2.88672352	0.50003141	1	-4.02560949	1.28267646	-1.38256860
6	1.26129031	-1.45672357	0.10773139	1	-1.18860972	3.60437655	-0.63956863
6	1.69099033	-1.10242343	-1.27446854	1	-0.50670975	2.70217657	0.71183139
6	0.16819027	-0.64662349	0.74073136	1	-0.34390974	2.09167647	-0.93116862
1	1.02909029	-1.59502351	-1.99896860	1	2.39829040	-3.27592349	0.21203139
1	2.70309043	-1.45932353	-1.48136854	1	1.28369033	-3.04042363	1.57353139
6	-1.83930969	-1.70322347	-0.42696863	1	0.66079026	-3.48722363	-0.01576862
6	-1.09660971	-0.42032349	-0.11776862	1	1.65309036	-0.02802350	-1.46626854
6	-2.12930965	0.48977649	0.57093138	6	4.66649055	1.89457655	-0.80806863
6	-3.38890958	-0.36242348	0.65253139	1	5.63219023	1.65817654	-1.25026858
7	-3.12820959	-1.58492351	0.04583139	1	4.75309038	2.78847647	-0.18986861
8	-1.41460967	-2.68352342	-0.99076861	1	3.94259024	2.08747649	-1.60156858
8	-4.44940948	-0.08092350	1.14993143	6	-4.10630989	-2.64172363	-0.06946862
6	-2.36700964	1.87817645	-0.09086861	1	-5.01900959	-2.30012345	0.41273141
1	-1.82030964	0.67897648	1.60573149	1	-3.74160957	-3.54632354	0.41673136
6	-3.26550961	2.72897649	0.81153136	1	-4.29900980	-2.86472344	-1.11886859
6	-1.02490973	2.59877658	-0.24406861				

TS__tBuMMI--IB-rad_MMI__si

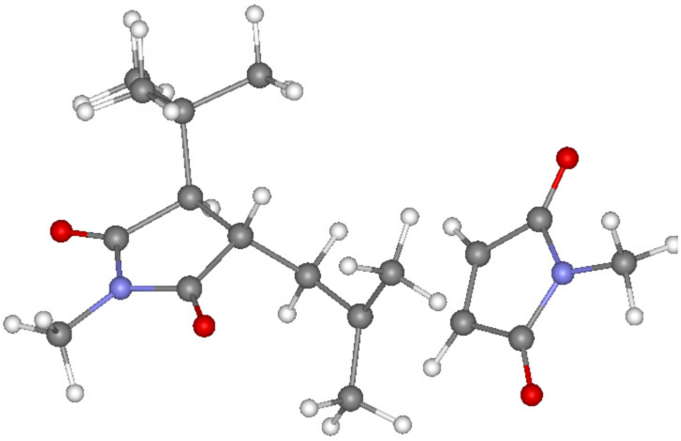

Zero-point vibrational energy	1136584.8 (Joules/Mol)
	271.65028 (Kcal/Mol)
Zero-point correction=	0.432902 (Hartree/Particle)
Thermal correction to Energy=	0.459401
Thermal correction to Enthalpy=	0.460345
Thermal correction to Gibbs Free Energy=	0.373334
Sum of electronic and zero-point Energies=	-1112.128897

Sum of electronic and thermal Energies= -1112.102398
Sum of electronic and thermal Enthalpies= -1112.101454
Sum of electronic and thermal Free Energies= -1112.188465

cartesian

6	4.05246639	1.42207837	0.46105492	1	-3.84213305	3.58807850	0.21615490
6	3.05766678	0.95317841	1.44205487	1	-4.44163322	2.10717845	0.97995490
6	3.05976677	-0.40922159	1.45595491	1	-3.04833317	2.94807863	1.65595496
6	4.17996645	-0.86822158	0.57765490	1	-3.57703328	2.45087862	-2.01714516
7	4.68706656	0.27247840	-0.02204511	1	-2.68843317	0.94167840	-2.16834497
8	4.32426691	2.54857850	0.12115490	1	-4.24133348	0.97857845	-1.32004511
8	4.58426666	-1.99272156	0.39565492	1	-1.66333330	3.66247845	-0.91394508
1	2.47066665	1.63857841	2.03485489	1	-0.82643330	2.95377851	0.46335489
1	2.64586663	-1.05712152	2.21375489	1	-0.66213328	2.23827863	-1.14484513
6	1.29106677	-1.27302158	0.09465490	6	1.41596675	-2.70272136	0.49985492
6	0.19066672	-0.43832159	0.68365490	1	2.43616676	-3.06632137	0.36165488
6	-1.78363323	-1.62262154	-0.43074507	1	0.75426674	-3.31742144	-0.12154511
6	-1.10223329	-0.29782158	-0.15564509	1	1.12636673	-2.86192155	1.54165494
6	-2.16393328	0.55817842	0.55925488	6	1.73806667	-0.92092162	-1.28394508
6	-3.32003331	-0.40442157	0.79975492	1	2.67506671	-1.41502154	-1.54474509
7	-3.02263331	-1.60732162	0.17415491	1	1.85396671	0.15777843	-1.42124510
8	-1.35303330	-2.55502152	-1.06474507	1	0.98916674	-1.27032161	-2.00714517
8	-4.33683348	-0.20592159	1.41455495	6	-3.92343330	-2.73662138	0.14795490
6	-2.60273337	1.85877836	-0.17624511	1	-4.80483341	-2.46862149	0.72555494
1	-1.80053329	0.86037838	1.54785490	1	-3.44203329	-3.61192155	0.58335489
6	-3.54383326	2.66627860	0.72245491	1	-4.20583344	-2.96972156	-0.87874508
6	-1.36503327	2.71537852	-0.45784509	6	5.76606655	0.28517842	-0.97614503
6	-3.31363320	1.53037846	-1.49114513	1	6.15876675	-0.72732162	-1.04444504
1	-0.83443332	0.12227842	-1.12714505	1	6.55246687	0.96467841	-0.64754510
1	0.55666673	0.58497840	0.82215494	1	5.41646671	0.60667843	-1.95864511
1	-0.07373329	-0.81422162	1.67795491				

tBuMMI--IB-rad--MMI__re

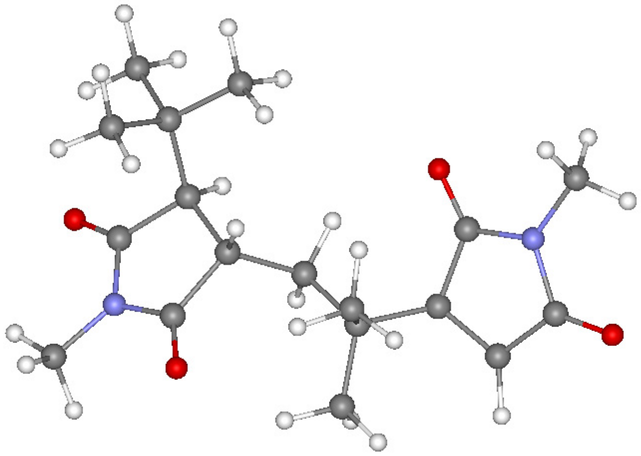

Zero-point vibrational energy	1146647.1 (Joules/Mol)
	274.05522 (Kcal/Mol)
Zero-point correction=	0.436735 (Hartree/Particle)
Thermal correction to Energy=	0.462491
Thermal correction to Enthalpy=	0.463435
Thermal correction to Gibbs Free Energy=	0.379674
Sum of electronic and zero-point Energies=	-1112.166798
Sum of electronic and thermal Energies=	-1112.141042
Sum of electronic and thermal Enthalpies=	-1112.140098
Sum of electronic and thermal Free Energies=	-1112.223858

cartesian

7	4.40846682	0.73953527	0.13908632	1	-4.30313301	2.12133527	1.01918638
6	3.09626675	0.72843528	0.55228633	1	-2.88433337	2.93903518	1.67088640
6	2.72196674	-0.71526474	0.91028631	1	-3.45763326	2.43703532	-1.99441373
6	3.99896669	-1.43346477	0.67988628	1	-2.60923338	0.90463525	-2.14411378
6	5.02036667	-0.52536476	0.20568632	1	-4.15453291	0.98573524	-1.28471363
1	4.20206690	-2.47586489	0.87088627	1	-1.51643324	3.61423516	-0.90231371
8	2.39846659	1.71373522	0.61078632	1	-0.66453332	2.87613511	0.45368633
1	2.46646667	-0.73616475	1.98018634	1	-0.54913330	2.16913533	-1.15501368
6	1.47136664	-1.26676476	0.13848631	6	1.37536669	-2.76976490	0.41328633
6	0.24076669	-0.51976472	0.71078628	1	2.22436666	-3.30776477	-0.01441368
6	-1.77113342	-1.64626479	-0.45641366	1	0.47496670	-3.18306470	-0.03681368
6	-1.02903342	-0.36046475	-0.14551368	1	1.35066676	-2.97726488	1.48778641
6	-2.06753325	0.52213526	0.57878631	6	1.64596677	-1.03426480	-1.36241364
6	-3.24393344	-0.40826476	0.83088630	1	2.58376670	-1.47176480	-1.71551371
7	-2.99603343	-1.60646474	0.17518631	1	1.65556669	0.02913526	-1.61621368
8	-1.40503335	-2.57236481	-1.14101362	1	0.83556670	-1.51276481	-1.91361368
8	-4.24293327	-0.19306473	1.46968639	8	6.17606688	-0.74356472	-0.08121369

6	-2.48313332	1.82993519	-0.15861368	6	-3.93823314	-2.70026469	0.12988631
1	-1.68733335	0.82033527	1.56158638	1	-4.82813311	-2.39066482	0.67258632
6	-3.39773345	2.66083527	0.74618632	1	-3.51053333	-3.58906484	0.59408629
6	-1.22993326	2.65863514	-0.45561370	1	-4.18943310	-2.93526483	-0.90411371
6	-3.21403337	1.51263523	-1.46541369	6	5.10096693	1.93603516	-0.27141368
1	-0.74703330	0.07303526	-1.10661364	1	6.14136696	1.66993523	-0.44391367
1	0.54826671	0.50053525	0.94548631	1	5.03186703	2.69673514	0.50608629
1	-0.03143331	-0.98446470	1.66678631	1	4.66616678	2.33563519	-1.18841362
1	-3.68453336	3.58493519	0.23738632				

tBuMMI--IB-rad--MMI__si

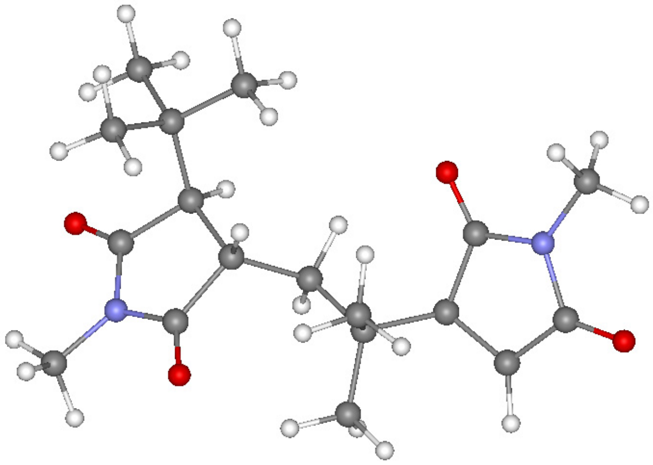

Zero-point vibrational energy	1146518.5 (Joules/Mol)
	274.02449 (Kcal/Mol)
Zero-point correction=	0.436686 (Hartree/Particle)
Thermal correction to Energy=	0.462481
Thermal correction to Enthalpy=	0.463425
Thermal correction to Gibbs Free Energy=	0.379172
Sum of electronic and zero-point Energies=	-1112.165550
Sum of electronic and thermal Energies=	-1112.139755
Sum of electronic and thermal Enthalpies=	-1112.138811
Sum of electronic and thermal Free Energies=	-1112.223064

cartesian

6	5.12050772	0.02793925	0.25336078	1	-4.30009222	-3.17486072	-0.27473924
6	4.01990795	-0.04786076	1.18966079	1	-4.43389225	-1.76906073	-1.34383917
6	2.76350808	-0.41466075	0.49106079	1	-3.07979202	-2.88416076	-1.51633930
6	3.23130798	-0.56636077	-0.96183926	1	-4.40489244	-1.72566080	1.79226065
7	4.58000755	-0.28096074	-1.00453925	1	-3.33539200	-0.34246075	1.98306084
8	6.28740788	0.28883925	0.44566077	1	-4.61339235	-0.31606072	0.75916076
8	2.56610799	-0.88356078	-1.91773915	1	-2.51819205	-3.34986067	1.40086079

1	4.15480804	0.11343925	2.24816084	1	-1.25529218	-2.99176073	0.22636077
1	2.42340803	-1.40576077	0.82636076	1	-1.37879217	-2.04926062	1.71826077
6	1.55770791	0.56893927	0.67876077	6	1.95990789	1.95013928	0.15416077
6	0.35870790	-0.01906075	-0.10813923	1	2.85530806	2.31543946	0.66526079
6	-1.50939214	1.74803925	0.19376075	1	1.16430795	2.67313933	0.32226077
6	-1.08319211	0.29823923	0.32906076	1	2.17360783	1.92253923	-0.91803926
6	-2.09899211	-0.49046075	-0.52503920	6	1.24640787	0.65753925	2.17336082
6	-2.96929193	0.57733923	-1.17083931	1	2.10660791	1.02183926	2.73826075
7	-2.55959201	1.81503928	-0.69673926	1	0.96160793	-0.31416076	2.58976078
8	-1.06009209	2.71343946	0.76466078	1	0.43310791	1.36283922	2.34606075
8	-3.86769223	0.43073922	-1.96053934	6	5.37570763	-0.32526076	-2.20623922
6	-2.92049193	-1.58166075	0.22046077	1	4.72460794	-0.62126076	-3.02543926
1	-1.57489216	-0.99176073	-1.34673929	1	6.18710756	-1.04506075	-2.09503913
6	-3.73499179	-2.39386058	-0.79073924	1	5.80840778	0.65463924	-2.40983915
6	-1.95779216	-2.53806067	0.93086082	6	-3.19089198	3.05973935	-1.06973922
6	-3.86619210	-0.94906080	1.24396086	1	-3.96489215	2.83083940	-1.79823923
1	-1.19739211	0.05203925	1.38746071	1	-2.45669198	3.73813939	-1.50343919
1	0.42940789	-1.11016071	-0.06783924	1	-3.63049197	3.53723931	-0.19403923
1	0.47320789	0.22403926	-1.16663933				

TS__tBuMMI--IB-rad_IB

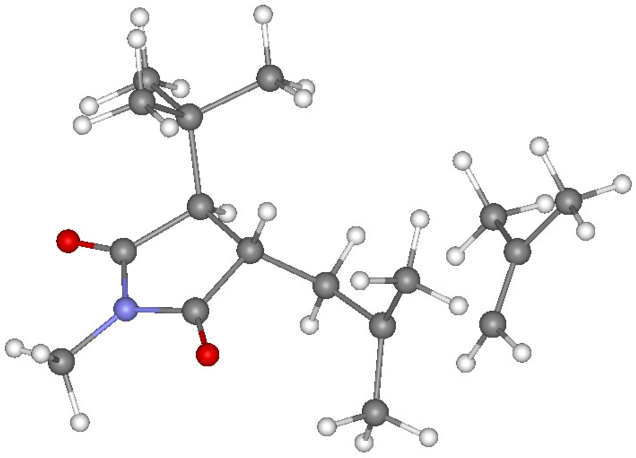

Zero-point vibrational energy	1165820.4 (Joules/Mol)
	278.63777 (Kcal/Mol)
Zero-point correction=	0.444038 (Hartree/Particle)
Thermal correction to Energy=	0.468545
Thermal correction to Enthalpy=	0.469489
Thermal correction to Gibbs Free Energy=	0.388619
Sum of electronic and zero-point Energies=	-870.581307
Sum of electronic and thermal Energies=	-870.556799
Sum of electronic and thermal Enthalpies=	-870.555855

Sum of electronic and thermal Free Energies= -870.636726

cartesian

6	4.44843245 1.01342607 -0.53363603			1	-3.16916800	3.65812588	-0.08493601
6	3.70253205	0.51082599	0.66266400	1	-3.83786798	2.32972598	0.87746400
6	3.43433189	-0.81637400	0.84126401	1	-2.31816792	3.08512592	1.35096407
1	3.97193217	-1.55367398	0.25426400	1	-3.21116805	2.27472591	-2.19603586
6	3.24123192	1.55312598	1.63346410	1	-2.48566794	0.67342597	-2.24453592
1	3.06913209	-1.16277397	1.80316401	1	-3.94766808	0.95912594	-1.28953600
6	1.60823214	-2.97757411	0.31476399	1	-1.10756791	3.39112592	-1.38863611
6	1.55103207	-1.55217397	-0.14193600	1	-0.22066790	2.74772596	-0.00913601
6	0.52403212	-0.64607400	0.47866401	1	-0.26586789	1.85402596	-1.53353596
1	0.80983210	-3.56987405	-0.14483601	1	2.55833197	-3.44507408	0.03856400
6	-1.64466786	-1.78177392	-0.28733599	6	1.84723222	-1.32177401	-1.59113598
6	-0.83946788	-0.50087398	-0.24103600	1	5.32763243	1.59602606	-0.23333600
6	-1.75676787	0.52172595	0.45496398	1	3.83403182	1.68712604	-1.14393592
6	-2.96626806	-0.29157400	0.89626402	1	2.77293205	-1.81847394	-1.89383602
7	-2.81866789	-1.58287394	0.41496399	1	1.94773209	-0.25807402	-1.82383609
8	-1.36166787	-2.81947398	-0.83383602	1	2.69193196	1.12102604	2.47156405
8	-3.91176796	0.07462598	1.54916406	1	4.09363222	2.10712600	2.04576397
6	-2.13846803	1.77742600	-0.38093600	1	1.49733210	-3.06317401	1.39956403
1	-1.27836788	0.88392597	1.37236404	1	1.04913211	-1.73167396	-2.22503591
6	-2.91696811	2.76502585	0.49346399	1	2.59873199	2.30482602	1.15796399
6	-0.85776794	2.47132587	-0.85383600	1	4.78993225	0.20052598	-1.17603612
6	-2.99056792	1.39112604	-1.59223604	6	-3.80276799	-2.62227416	0.60606402
1	-0.64626789	-0.22277401	-1.27963591	1	-4.61276770	-2.20437407	1.19916391
1	0.93633211	0.36832595	0.51936400	1	-3.35836792	-3.47117400	1.12546396
1	0.34143206	-0.94217402	1.51836395	1	-4.18166780	-2.96457410	-0.35693601

TS__tBuMMI--IB-rad_IB_transfer

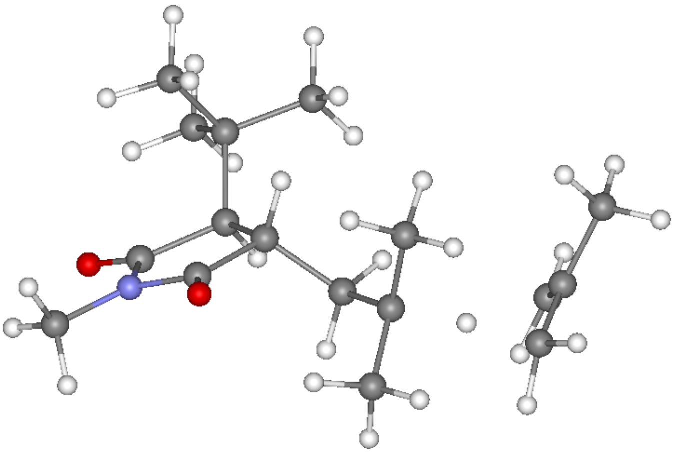

Zero-point vibrational energy	1154894.4 (Joules/Mol)
	276.02639 (Kcal/Mol)
Zero-point correction=	0.439876 (Hartree/Particle)
Thermal correction to Energy=	0.464512
Thermal correction to Enthalpy=	0.465457
Thermal correction to Gibbs Free Energy=	0.383336
Sum of electronic and zero-point Energies=	-870.572986
Sum of electronic and thermal Energies=	-870.548349
Sum of electronic and thermal Enthalpies=	-870.547405
Sum of electronic and thermal Free Energies=	-870.629526

cartesian

6	1.43705010	-3.07812595	0.37986404	1	-3.08544993	2.45167398	-2.16633582
6	1.44525003	-1.66912591	-0.15723597	1	-2.48854995	0.79837406	-2.21133590
6	1.59825015	-1.57632589	-1.65413594	1	-3.91085005	1.20447409	-1.23953605
6	0.46985006	-0.68942595	0.46686405	1	-0.89664996	3.41157413	-1.39073598
1	0.55605006	-3.62682605	0.03466402	1	-0.03704995	2.70627403	-0.02323598
1	2.49615002	-2.10112596	-1.99103606	1	-0.17674994	1.81497407	-1.54223597
1	1.67105007	-0.54012597	-1.99723589	1	2.32075000	-3.62892604	0.04446402
6	-1.77614999	-1.71242595	-0.22233598	6	3.78605008	-0.74582601	0.85146403
6	-0.89644992	-0.47962597	-0.21973597	6	3.88134980	0.70477402	0.74216402
6	-1.73545003	0.60837400	0.47736400	6	4.38205004	1.25347400	-0.56543601
6	-2.99515009	-0.11582597	0.93246400	6	3.51504993	1.52537405	1.74496400
7	-2.93245006	-1.42352593	0.47586399	1	2.63085008	-1.17052591	0.31666404
8	-1.55725002	-2.78152585	-0.73783600	1	3.78034997	0.89197409	-1.40433598
8	-3.91405010	0.32187402	1.57886398	1	5.40945005	0.92727411	-0.75443602
6	-2.03125000	1.88507414	-0.36123598	1	3.56695008	2.60397410	1.64586401
1	-1.22534990	0.93827403	1.38976395	1	3.16945004	1.13747406	2.69756413
6	-2.72134995	2.93227410	0.51826400	1	3.70045018	-1.13592601	1.86436403
6	-0.70844996	2.47757411	-0.85523599	1	0.74475008	-2.04002595	-2.16203594
6	-2.92715001	1.55797410	-1.55813599	1	1.43335009	-3.09072590	1.47346401
1	-0.71884990	-0.23102596	-1.26873600	1	4.50115013	-1.31232595	0.25526404
1	0.94285005	0.29997402	0.49116403	1	4.36675024	2.34387398	-0.58233601
1	0.29975009	-0.96042597	1.51506400	6	-3.97784996	-2.39562583	0.69446403
1	-2.91774988	3.83777404	-0.06243598	1	-4.74704981	-1.92262590	1.30026400
1	-3.66494989	2.56687403	0.91966403	1	-3.57754993	-3.26792598	1.21066403
1	-2.08664989	3.21337414	1.36366403	1	-4.39854956	-2.71902585	-0.25783598

tBuMMI--IB-rad--IB

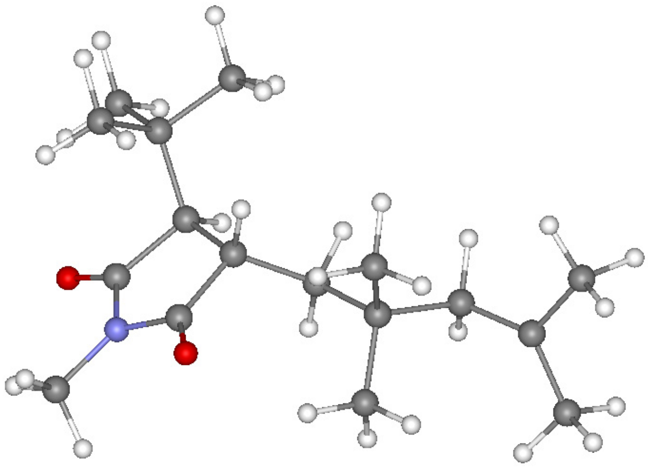

Zero-point vibrational energy	1174802.8 (Joules/Mol)
	280.78461 (Kcal/Mol)
Zero-point correction=	0.447459 (Hartree/Particle)
Thermal correction to Energy=	0.471710
Thermal correction to Enthalpy=	0.472654
Thermal correction to Gibbs Free Energy=	0.391976
Sum of electronic and zero-point Energies=	-870.616047
Sum of electronic and thermal Energies=	-870.591796
Sum of electronic and thermal Enthalpies=	-870.590851
Sum of electronic and thermal Free Energies=	-870.671530

cartesian

6	-5.06587648	0.73642194	1.22040999	1	4.59652376	-2.78427792	0.49140999
6	-4.22997618	-0.19407804	0.40920997	1	4.74972343	-1.15107810	1.15881002
6	-2.79227614	-0.40967804	0.75601000	1	3.60752392	-2.28847790	1.86690998
1	-2.63197613	-0.16657804	1.81561005	1	4.14252377	-1.87697804	-1.81788993
6	-4.94217634	-1.15407813	-0.48189002	1	2.86842394	-0.69657803	-2.09469008
1	-2.54787612	-1.47517812	0.64760995	1	4.32742357	-0.23777805	-1.20538998
6	-2.04697609	1.88762188	0.03840999	1	2.58312392	-3.51507807	-0.69119000
6	-1.73687601	0.39352196	-0.06719001	1	1.52812386	-2.98837805	0.61811000
6	-0.37137616	0.05132194	0.57211000	1	1.23742390	-2.43787789	-1.03638995
1	-1.33497620	2.48412204	-0.52919000	1	-3.04487610	2.09622192	-0.35499004
6	1.31202388	1.60392189	-0.62519002	6	-1.78997612	-0.03297804	-1.53319001
6	0.93472385	0.18712194	-0.23469000	1	-5.60137653	0.20262195	2.02280998
6	2.13952374	-0.31827801	0.58760995	1	-5.83757639	1.22582185	0.61660999
6	2.97902393	0.92662197	0.83200997	1	-2.79147601	0.12442195	-1.93928993
7	2.44482398	1.96122193	0.08030999	1	-1.54507613	-1.09347808	-1.65758991
8	0.76672387	2.33232212	-1.41798997	1	-4.25917625	-1.71127808	-1.12548995
8	3.95092392	1.04292190	1.53611004	1	-5.51247644	-1.89757812	0.09870999

6	2.95612383	-1.48207808	-0.04889001	1	-2.02447605	2.22362208	1.08030999
1	1.80622387	-0.66737807	1.57071006	1	-1.09687614	0.55582196	-2.13689017
6	4.04392385	-1.94607818	0.92430997	1	-5.67317629	-0.65047807	-1.12428999
6	2.01662374	-2.66397786	-0.30499002	1	-4.47257614	1.51632190	1.70100999
6	3.60422397	-1.04187810	-1.36328995	6	3.03682399	3.27582192	-0.00299001
1	0.82632381	-0.37037802	-1.16648996	1	3.91162395	3.28502202	0.64280999
1	-0.40167618	-1.00277805	0.87210995	1	2.32362390	4.03222179	0.32410997
1	-0.26927614	0.61602193	1.50700998	1	3.32632399	3.49362206	-1.03109002
